# A simple and convenient one-pot synthesis of substituted isoindolin-1-ones via lithiation, substitution and cyclization of *N'*-benzyl-*N,N*-dimethylureas

**DOI:** 10.3762/bjoc.7.142

**Published:** 2011-09-06

**Authors:** Keith Smith, Gamal A El-Hiti, Amany S Hegazy, Benson Kariuki

**Affiliations:** 1 School of Chemistry, Cardiff University, Main Building, Park Place, Cardiff CF10 3AT, UK, Fax: +44(0)2920870600; Tel: +44(0)2920870600; 2 Permanent address: Chemistry Department, Faculty of Science, Tanta University, Tanta 31527, Egypt

**Keywords:** *N'*-benzyl-*N,N*-dimethylureas, isoindolin-1-ones, directed lithiation, electrophiles, substitution, synthesis

## Abstract

Lithiation of *N'*-benzyl-*N,N*-dimethylurea and its substituted derivatives with *t-*BuLi (3.3 equiv) in anhydrous THF at 0 °C followed by reaction with various electrophiles afforded a range of 3-substituted isoindolin-1-ones in high yields.

## Introduction

In recent years there has been a great deal of interest in compounds possessing an isoindolinone ring system since it represents the core unit of numerous naturally occurring substances [1–8]. Also, some members that possess this moiety have shown interesting biological properties [9–15].

Several traditional methods are available for the synthesis of isoindolinones [16–25], based on the use of Grignard reagents [26], Diels–Alder reactions [27], Wittig reagents [28], reduction processes [29–30], rearrangement processes [31] and photochemical reactions [32–33]. However, such methods generally require multiple reaction steps and are unsatisfactory both in yield and generality. In recent years several new approaches have been developed for the synthesis of substituted isoindolines, of which the most generally useful involve palladium-catalysed reactions [34–42] or lithiation procedures [43–54].

**Scheme 1:**
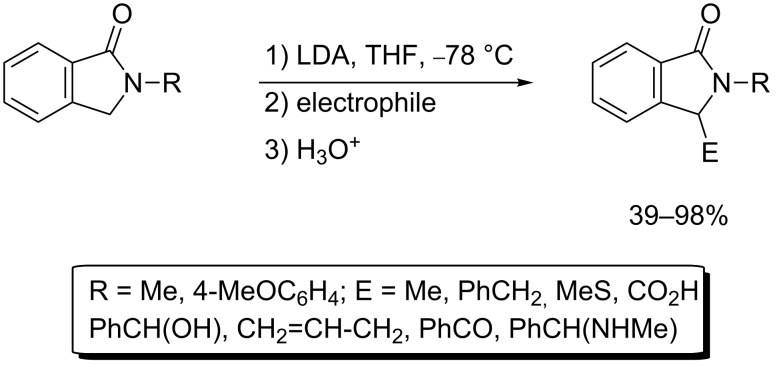
Lithiation and substitution of isoindolin-1-ones [43].

**Scheme 2:**
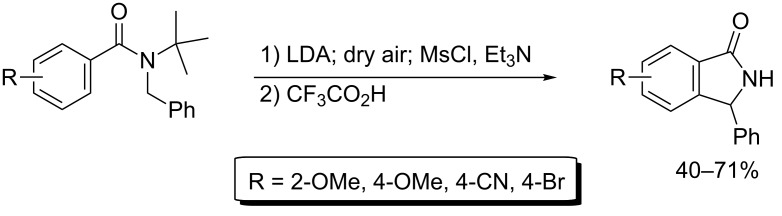
Lithiation and cyclization of *N*-*tert*-butyl-*N-*benzylbenzamides [45].

In particular, among the various lithiation methods two useful approaches to the synthesis of isoindolin-1-ones have been reported (Scheme 1 and Scheme 2) [43,45]. One method simply involves lithiation of a preformed isoindolin-1-one ring system at the 3-position followed by treatment with an electrophile (Scheme 1) [43]. While this approach is straightforward, clearly, its general utility depends on the availability of appropriately substituted analogues of the isoindolin-1-one ring system.

The other, potentially more useful approach involves generation of the heterocyclic ring system during the lithiation step. For example, lithiation of *N*-*tert*-butyl-*N*-benzylbenzamides gives intermediates that cyclise to form de-aromatised species. Oxidation to re-aromatise the benzenoid system, followed by treatment with trifluoroacetic acid to remove the *tert*-butyl group, gives the corresponding isoindolin-1-ones (Scheme 2) [45]. However, this approach gives more modest yields, requires an additional step to remove the *tert*-butyl group, and also involves incorporating the eventual C-3 substituent into the starting material, which limits the generality. Moreover, the reaction works well only with 3-aryl substituents.

Clayden has improved the yield of isoindolin-1-ones by using 2-methoxybenzamides as the starting materials; in this case the methoxy group acts as a leaving group, which avoids the need for an oxidation step [45]. However, this approach still requires an additional step to remove the *tert*-butyl group and incorporation of the 3-substituent into the starting material.

**Scheme 3:**
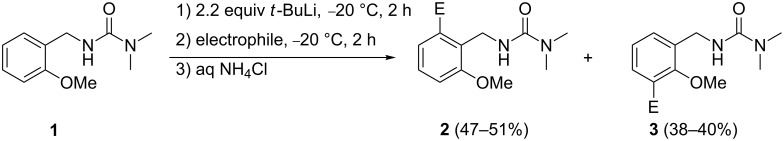
Lithiation and substitution of **1** at −20 °C [69].

**Figure 1:**
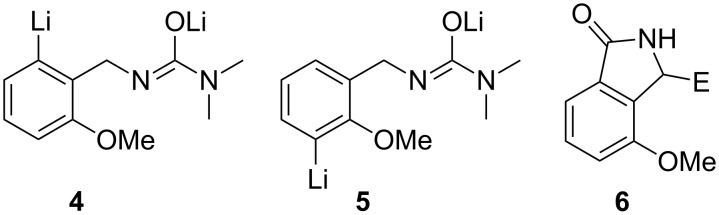
Structures of **4**–**6**.

As a result of our own interest in the use of lithium reagents in organic synthesis [55–67], we have had occasion to investigate lithiation of various aromatic compounds containing substituted amino groups. Factors that influence the site(s) of lithiation of such compounds can be quite subtle. For example, lithiation of *N*,*N*-dimethyl-*N'*-(substituted phenyl)ureas can occur predominantly on the *N*,*N*-dimethyl group (e.g., for the unsubstituted, 4-methyl or 4-methoxy-compounds with *t*-BuLi at −20 °C) or on the ring next to the urea group (e.g., for the 4-chloro-, 4-fluoro- or 4-trifluoromethyl compounds with either *n*-BuLi or *t*-BuLi at 0 °C) [59]. However, a recent report indicates that replacement of the dimethylamino group by a more hindered dialkylamino group in the unsubstituted phenyl derivative results in lithiation on the ring [68]. We have also developed procedures for the lithiation of various substituted benzylamines [69–71]. Similar subtleties over the site(s) of lithiation are also observed for these compounds. The site(s) of lithiation depend on the substituents at nitrogen, the nature and positions of substituents on the aryl ring and/or on the nature of the lithium reagent. For example, lithiation of *N*-benzylpivalamide with *t*-BuLi gives a mixture of ring (2-position) and side-chain lithiated species, whereas for *N'*-benzyl-*N,N-*dimethylurea side-chain lithiation does not occur [69]. Of particular relevance for the present study was the observation that lithiation of *N'*-(2-methoxybenzyl)-*N,N*-dimethylurea (**1**) with two equivalents of *t*-BuLi in THF at −20 °C for 2 h followed by reactions with a range of electrophiles gave mixtures of products (Scheme 3) involving ring substitution both next to the urea-containing group (*o*-substitution; 47–51% yields) and next to the methoxy group (*o*'-substitution; 38–40% yields) [69]. Formation of **2** and **3** presumably involved lithium intermediates **4** and **5**, respectively (Figure 1).

We found that when the lithiation reaction was carried out at 0 °C rather than at −20 °C, followed by reaction with an electrophile, it produced lower yields of the corresponding substituted products **2** and **3**, along with some residual **1** and a small amount of a new product of structure **6** (Figure 1). By increasing the amount of lithiating agent and extending the period of the lithiation reaction, we were able to increase the yield of **6**, and in a preliminary communication we reported that directed lithiation of various *N'*-benzyl-*N,N*-dimethylureas with *t*-BuLi (3.3 equiv) in anhydrous THF at 0 °C followed by reaction with various electrophiles afforded the corresponding 3-substituted isoindolin-1-ones in high yields [72]. We now report full details of that work, extend the scope of the reaction and examine the diastereoselectivity of the reaction with prochiral electrophiles.

## Results and Discussion

**Scheme 4:**
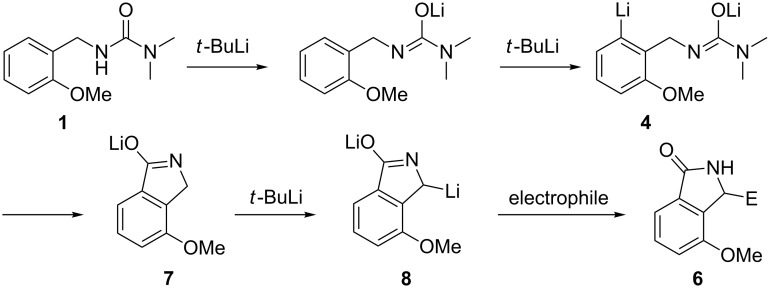
A possible mechanism for the formation of **6**.

As noted above, when lithiation of **1** was carried out at 0 °C rather than at −20 °C prior to reaction with an electrophile, lower yields of **2** and **3** along with some residual **1** and a small amount of **6** were obtained. A possible mechanism for the formation of **6** is shown in Scheme 4. According to this scheme, compounds of the general structure **6** would arise by cyclization of **4** to give **7**, followed by further lithiation to give **8**, which on reaction with an electrophile would give **6**. It appeared likely that the yield of **6** could be increased by use of a larger quantity of *t*-BuLi and an extended reaction time; therefore, we investigated this possibility.

Indeed, lithiation of **1** with *t*-BuLi (3.3 equiv) in anhydrous THF at 0 °C for 6 h, followed by treatment with iodomethane, gave **9** (i.e., **6**, where E = Me) in 79% yield (Table 1). While an increased yield of **6** (E = Me, i.e., **9**, produced from **7**) and the disappearance of **2** (produced from **4**) were expected, the disappearance of **3** (produced from **5**) was a surprise. It would appear that at 0 °C, not only did **4** cyclize to give **7**, but **5** was also in equilibrium with **4**, allowing its eventual conversion into **7** and then **8**.

**Table 1:** Synthesis of 3-substituted 4-methoxyisoindolin-1-ones **9**–**18**.

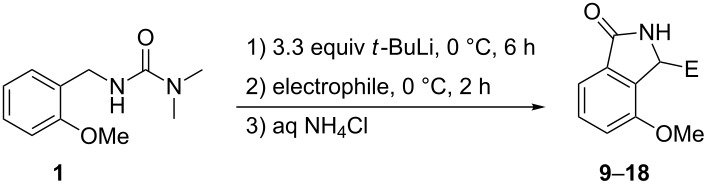			

Product	Electrophile	E	Yield (%)^a^

**9**	MeI	Me	79
**10**	EtI	Et	84
**11**	BuBr	Bu	72
**12**	H_2_O	H	82
**13**	Ph_2_CO	Ph_2_C(OH)	81
**14**	(CH_2_)_5_CO	(CH_2_)_5_C(OH)	78
**15**	BuCOMe	BuC(OH)Me	78
**16**	PhCOMe	PhC(OH)Me	80
**17**	4-MeOC_6_H_4_CHO	4-MeOC_6_H_4_CH(OH)	81
**18**	PhCHO	PhCH(OH)	80

^a^Yield of material isolated by washing or triturating the crude product with diethyl ether.

This fortuitous finding appeared to offer potential as a general synthetic method and the same lithiation procedure was therefore used with a range of other electrophiles including other haloalkanes, water, symmetrical and unsymmetrical ketones, and aldehydes. After work-up of the reaction mixtures, the crude products were triturated and/or washed with diethyl ether to give products **10**–**18** in high yields (Table 1).

**Figure 2:**
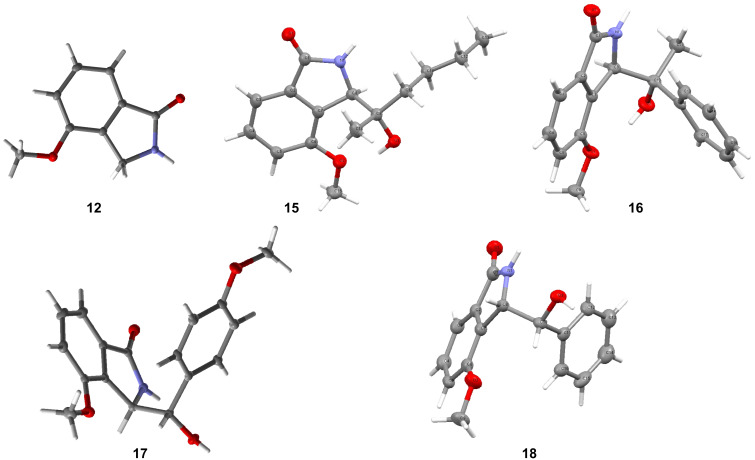
X-Ray crystal structures of crystallized compounds **12** and **15**–**18**.

From the results recorded in Table 1, it appeared that the reaction was a general process for production of 3-substituted 4-methoxyisoindolin-1-ones **9–18** in high yields from reactions with a variety of electrophiles. The identity of the ring system was confirmed by the crystallization of **12** and subjecting it to X-ray crystallography (Figure 2). All the products were analysed by standard spectroscopic methods and showed, for example, the expected molecular ions in their mass spectra. Compounds **10**–**14** were readily identified by their NMR spectra, which showed all the expected signals. In the ^1^H NMR spectra of compounds **10** and **11**, the expected diastereotopicity of the two hydrogens of the CH_2_ groups closest to the ring was evident. For compound **13** the two phenyl groups appeared as separated signals in the ^13^C NMR spectrum, verifying that they were also diastereotopic, and similarly, the two sides of the cyclohexane ring in compound **14** also appeared as separated signals. The NMR spectra of products **15**–**18** were relatively complex. Therefore, in order to get further information about these products, they were each crystallized from ethyl acetate. The ^1^H NMR spectra of the crystallized materials showed what appeared to be a single component in each case. The crystalline products were therefore subjected to X-ray crystallography. The structures found are illustrated in Figure 2.

The X-ray crystal structures of the isolated crystals of products **15**, **17** and **18** showed that they were α-(R*)-3-(S*)-diastereoisomers, while that of **16** showed that it was the α-(R*)-3-(R*)-diastereoisomer (Figure 2). With this information in hand, the NMR spectra of the initial products obtained by trituration or washing of the original crude products were re-examined. These spectra showed the presence of the crystallized product and a second component, the identifiable resonances of which were consistent with the other diastereoisomer. The two diastereoisomers were present in nearly equal proportions (between 47:53 and 42:58), with the α-(R*)-3-(R*)-diastereoisomer being the somewhat predominant diastereoisomer in all cases.

We have shown previously that lithiation of *N'*-benzyl-*N,N*-dimethylurea and *N'*-(4-substituted benzyl)-*N,N*-dimethylureas with *t*-BuLi (two equiv) in THF at −78 °C for 4 h followed by reactions with a variety of electrophiles gave high yields of products involving substitution at the 2-position [69]. Therefore, it was of interest to investigate further the scope of the process represented in Table 1 with other ring-substituted *N'*-benzyl-*N,N*-dimethylureas **19** (R = H, 4-OMe, 4-Me; Table 2). Each substrate was lithiated according to the standard procedure with *t*-BuLi (3.3 equiv) in anhydrous THF at 0 °C for 6 h, and then treated with various electrophiles. After work-up, as described above, the substituted isoindolin-1-ones **20–40** were obtained in high yields (Table 2).

**Table 2:** Synthesis of various substituted isoindolin-1-ones **20**–**40**.

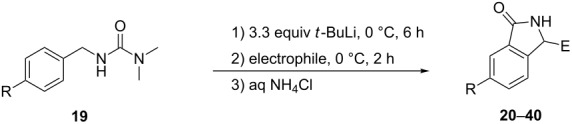				

Product	R	Electrophile	E	Yield (%)^a^

**20**	H	H_2_O	H	71
**21**	H	MeI	Me	75
**22**	H	EtI	Et	77
**23**	H	BuBr	Bu	76^b^
**24**	H	Ph_2_CO	Ph_2_C(OH)	74
**25**^c^	H	PhCHO	PhCH(OH)	73
**26**^d^	H	4-MeOC_6_H_4_CHO	4-MeOC_6_H_4_CH(OH)	78
**27**	OMe	H_2_O	H	70
**28**	OMe	MeI	Me	76
**29**	OMe	EtI	Et	78
**30**	OMe	BuBr	Bu	77
**31**	OMe	Ph_2_CO	Ph_2_C(OH)	72
**32**^c^	OMe	4-MeOC_6_H_4_CHO	4-MeOC_6_H_4_CH(OH)	75
**33**	Me	H_2_O	H	75
**34**	Me	MeI	Me	78
**35**	Me	EtI	Et	75
**36**	Me	(CH_2_)_5_C=O	(CH_2_)_5_C(OH)	72
**37**	Me	Ph_2_CO	Ph_2_C(OH)	85
**38**^e^	Me	MeCOBu	MeC(OH)Bu	77
**39**^e^	Me	PhCHO	PhCH(OH)	79
**40**^d^	Me	4-MeOC_6_H_4_CHO	4-MeOC_6_H_4_CH(OH)	83

^a^Yield of product isolated by trituration or washing of the initial reaction mixture with diethyl ether. ^b^Compound **41** (Figure 3) was obtained in 5% yield. ^c^The ^1^H NMR spectrum of the solid after crystallization showed the structure to be the α-(R*)-3-(R*)-isomer. ^d^The ^1^H NMR spectrum of the solid after crystallization showed the structure to be the α-(R*)-3-(S*)-isomer. ^e^X-Ray crystallography showed the product isolated by crystallization to be the α-(R*)-3-(R*)-isomer (Figure 4).

**Figure 3:**
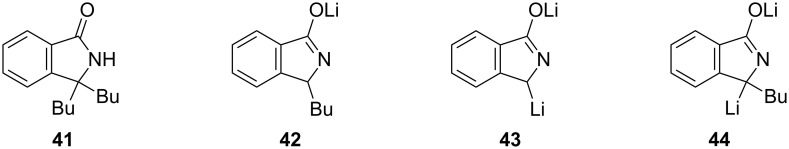
Structures of compounds **41**–**44**.

**Figure 4:**
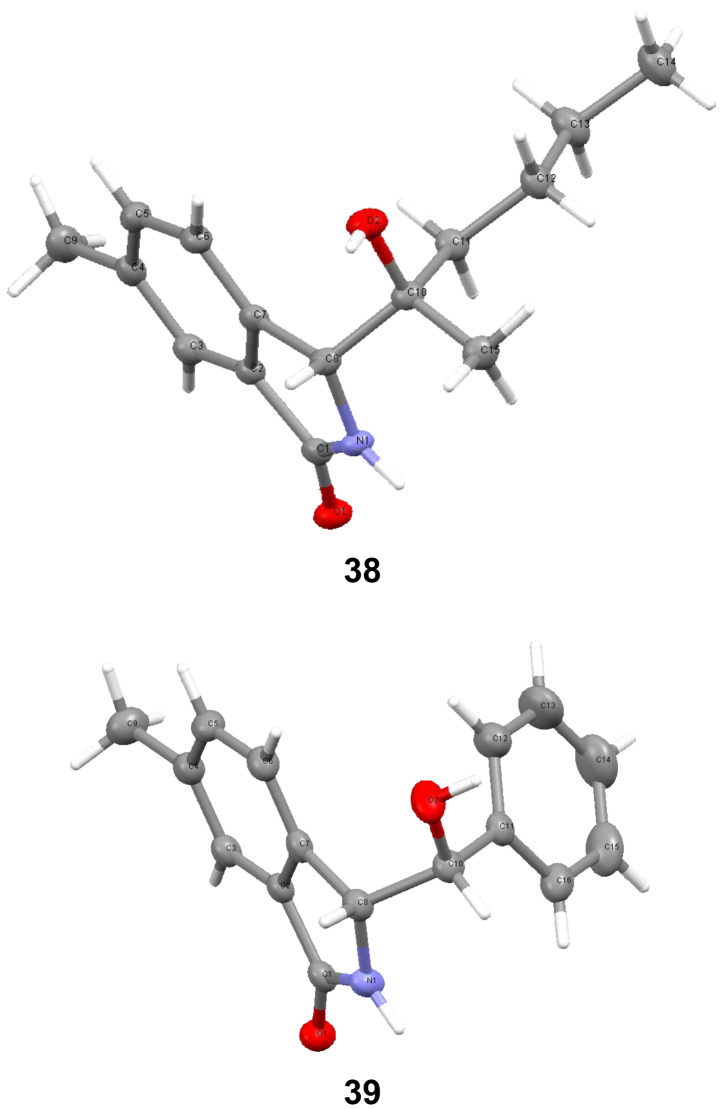
X-ray crystal structures of compounds **38** and **39**.

The ^1^H NMR spectra of compounds **22–24**, **29**–**31** and **35–38** showed diastereotopicity features similar to those for compounds reported in Table 1. The NMR spectra of **25**, **26**, **32**, and **38–40** showed the presence of two racemic diastereoisomers in unequal proportions (up to ca. 38:62). However, the NMR spectra of the products after crystallization from ethyl acetate all exhibited one set of signals, indicating that the isolated crystalline product in each case was a single racemic diastereoisomer. X-ray crystallography of the isolated crystals of compounds **38** and **39** showed them to be the α-(R*)-3-(R*)-diastereoisomers (Figure 4). The ^1^H NMR spectra of the isolated crystalline products **26**, **32** and **40** indicated that the crystals were α-(R*)-3-(S*)-diastereoisomers, while the ^1^H NMR spectrum of the crystallized product **25** showed it to be α-(R*)-3-(R*)-isomer. With the help of the NMR spectra of the crystallized diastereoisomers, re-examination of the spectra of the materials obtained by simple trituration or washing of the original crude product allowed calculation of the proportions of the two diastereoisomers and in all cases the major diastereoisomer was again the α-(R*)-3-(R*)-isomer, as was the case when 2-methoxybenzyl-*N,N*-dimethylurea was the starting material.

Compound **41** (Figure 3) was obtained as a side product in 5% yield when 1-bromobutane was used as the electrophile. Compound **41** arose due to further lithiation at position 3 of **42**, produced in situ from **43**, to generate the lithium intermediate **44** (Figure 3). Reaction of the latter with a further equivalent of 1-bromobutane affords **41**.

## Conclusion

We have developed a novel, simple, efficient, general and high yielding procedure for synthesis of isoindolin-1-ones in a one-step reaction. It allows easy addition of a range of substituents to the initial benzene ring and incorporation of a range of substituents derived from the electrophiles used. Isolation of the pure products is also extremely easy, involving simple trituration and/or washing of the crude product after work up, except in the cases where two diastereoisomers are produced. In such cases the α-(R*)-3-(R*)-isomer is somewhat predominant (ratios of ca. 47:53 to 38:62); crystallization of these mixtures produces one pure diastereoisomer in all cases. The method promises to be a very useful new approach for synthesis of substituted isoindolin-1-ones.

## Experimental

### General information

Melting point determinations were performed by the open capillary method with a Gallenkamp melting point apparatus and are uncorrected. ^1^H and ^13^C NMR spectra were recorded on a Bruker AV400 or AV500 spectrometer at 400 or 500 MHz for ^1^H and 100 or 125 MHz for ^13^C measurements. Chemical shifts δ are reported in parts per million (ppm) relative to TMS and coupling constants *J* are in Hz and have been rounded to the nearest whole number. ^13^C multiplicities were revealed by DEPT signals. Assignments of signals are based on integration values, coupling patterns and expected chemical shift values and have not been rigorously confirmed. Signals with similar characteristics might be interchanged. Low-resolution mass spectra (see Supporting Information File 1) were recorded on a Quattro II spectrometer, electron impact (EI) at 70 eV and chemical ionization (CI) at 50 eV by the use of NH_3_ as ionization gas. Atmospheric pressure chemical ionization (APCI) mass spectra were measured on a Waters LCT Premier XE instrument. Electrospray (ES) analyses were performed on a ZQ4000 spectrometer in positive and negative ionisation modes. Accurate mass data were obtained on a MAT900 instrument. IR spectra (see Supporting Information File 1) were recorded on a Perkin Elmer Spectrum One FT-IR spectrometer or a Perkin Elmer 1600 series FT-IR spectrometer. Microanalyses for representative compounds were performed by Warwick analytical service at the University of Warwick. The X-ray single-crystal diffraction data were collected on a Nonius Kappa CCD diffractometer using graphite-monochromated Mo-K_α_, (λ = 0.71073 Å) radiation. Crystal and structure refinement data are shown in the Supporting Information File 1. The structures were solved by direct methods using SHELXS-96 [73] and refined with all data on F^2^ full-matrix least squares using SHELXL-97 [74]. Non-hydrogen atoms were generally refined anisotropically. Hydrogen atom positions were located from difference Fourier maps and a riding model with atomic displacement parameters 1.2 times (1.5 times for methyl groups) those of the atom to which they are bonded were used for subsequent refinements. Full crystallographic data have been deposited with the CCDC, reference numbers 737411 (compound **12**), 762624 (compound **15**), 766180 (compound **16**), 737415 (compound **17**), 762623 (compound **18**), 766182 (compound **38**) and 766181 (compound **39**), and can be obtained free of charge via http://www.ccdc.cam.ac.uk/data_request/cif. Alkyl lithiums were obtained from Aldrich Chemical Company and were estimated prior to use by the method of Watson and Eastham [75]. Other reagents and starting materials were obtained from Aldrich Chemical Company and used without further purification. THF was distilled from sodium benzophenone ketyl.

**General procedure for the synthesis of 3-substituted isoindolin-1-ones 9–18 and 20–40.** A solution of *t*-BuLi in heptane (3.9 mL, 1.7 M, 6.6 mmol) was added to a cold (0 °C), stirred solution of *N'*-(substituted benzyl)-*N,N*-dimethylurea (**1** or **19**; 2.0 mmol) in anhydrous THF (20 mL) under a N_2_ atmosphere. Formation of the monolithium reagent was observed as a yellow solution and the dilithium reagent was observed as a reddish orange solution, after which the colour changed to deep red. The mixture was stirred at 0 °C for 6 h after which an electrophile (2.2 mmol), in anhydrous THF (8 mL) if solid, otherwise neat, was added. The mixture was stirred for 2 h at 0 °C then the cooling bath was removed and the mixture allowed to warm to room temperature. It was then diluted with Et_2_O (10 mL) and quenched with aq sat. NH_4_Cl (10 mL). The organic layer was separated, washed with H_2_O (2 × 10 mL), dried (MgSO_4_), and evaporated under reduced pressure. The residue obtained was triturated with diethyl ether (20 mL) to give a white solid which was filtered and washed with diethyl ether (20 mL) to give the pure product. The solid obtained was recrystallized from ethyl acetate. Some of the products were previously reported and in these cases the melting point and the spectral data were in agreement with reported values. Of those compounds that had not been reported previously, representative examples were subjected to microanalysis, and in all cases gave analyses consistent with the assigned structures. For other compounds, therefore, proof of purity was established by a combination of tlc (single spot), sharp melting point and clean NMR spectra. Elemental composition of a molecular ion or pseudo molecular ion confirmed the formula.

## Supporting Information

File 1 Characterization data of all compounds given in the article and NMR spectra and X-ray information for representative compounds. CCDC 737411, 737415, 762623, 762624, 766180, 766181 and 766182.
