# Supplementary material for: A simple and convenient one-pot synthesis of substituted isoindolin-1-ones via lithiation, substitution and cyclization of N'-benzyl-N,N-dimethylureas
Source: Beilstein J Org Chem. 2011 Sep 6;7:1219–27. doi: 10.3762/bjoc.7.142 (PMC3182431; doi:10.3762/bjoc.7.142)
Supplement: File 1 — Characterization data of all compounds given in the article and NMR spectra and X-ray information for representative compounds. CCDC 737411, 737415, 762623, 762624, 766180, 766181 and 766182. [file Beilstein_J_Org_Chem-07-1219-s001.pdf]

Supporting Information

for

A simple and convenient one-pot synthesis of substituted isoindolin-1-ones via lithiation, substitution and cyclization of *N*'-benzyl-*N*,*N*-dimethylureas

Keith Smith[*], Gamal A. El-Hiti*[1,2], Amany S. Hegazy[1] and Benson Kariuki[1]

Address: [1]School of Chemistry, Cardiff University, Main Building, Park Place, Cardiff CF10 3AT, UK, Fax: +44(0)2920870600; Tel: +44(0)2920870600 and [2]Permanent address: Chemistry Department, Faculty of Science, Tanta University, Tanta 31527, Egypt

Email: Keith Smith - smithk13@cardiff.ac.uk; Gamal A. El-Hiti - el-hitiga@cardiff.ac.uk

*Corresponding author

Characterization data of all compounds given in the article and NMR spectra and X-ray information for representative compounds. CCDC 737411, 737415, 762623, 762624, 766180, 766181 and 766182.

Table of contents

Characterization data for products	S03–S20
NMR spectra of compound **9**	S23–S30
NMR spectra of compound **10**	S31–S37
NMR spectra of compound **12**	S38–S43
NMR spectra of compound **13**	S44–S50
NMR spectra of compound **14**	S51–S57
NMR spectra of compound **15a**	S58–S63
NMR spectra of compound **16a**	S64–S71
NMR spectra of compound **17a**	S72–S76
NMR spectra of compound **20**	S77–S82
NMR spectra of compound **26a**	S83–S92
NMR spectra of compound **28**	S93–S100
NMR spectra of compound **29**	S101–S109
NMR spectra of compound **30**	S110–S118
NMR spectra of compound **34**	S119–S126
NMR spectra of compound **35**	S127–S135
NMR spectra of compound **38a**	S136–S146
NMR spectra of compound **39a**	S147–S153
NMR spectra of compound **40a**	S154–S164
NMR spectra of compound **41**	S165–S173
Crystal and structure refinement data of compounds **12** and **15**	S174
Crystal and structure refinement data of compounds **16-18**	S175
Crystal and structure refinement data of compounds **38** and **39**	S176

Characterization Data for Products

Note: When the multiplicity of a proton NMR signal appears simpler than expected (for example if a signal expected to be a double doublet appears as a triplet because the two coupling constants are very similar), the observed apparent multiplicity is recorded without comment in the data reported below.

4-Methoxy-3-methylisoindolin-1-one (9). Yield: 0.28 g (1.58 mmol, 79%); mp 153–154 °C; IR (FT) ν/cm^{-1}: 3071, 2971, 1680, 1602, 1493, 1267, 1057; ^{1}H NMR (400 MHz, $CDCl_3$) δ (ppm): 7.26 (br s, exch., 1H, NH), 7.47 (t, *J* = 8 Hz, 1H, H-6), 7.24 (d, *J* = 8 Hz, 1H, H-7), 7.15 (d, *J* = 8 Hz, 1H, H-5), 4.73 (q, *J* = 7 Hz, 1H, H-3), 3.92 (s, 3H, OCH_3), 1.55 (d, *J* = 7 Hz, 3H, CH_3); ^{13}C NMR (100 MHz, $CDCl_3$) δ (ppm): 171.2 (s, C-1), 155.3 (s, C-4), 136.8 (s, C-7a), 133.9 (s, C-3a), 130.1 (d, C-6), 116.0 (d, C-7), 113.6 (d, C-5), 55.8 (q, OCH_3), 52.0 (d, C-3), 19.3 (q, CH_3); MS (EI) *m/z*: 177 ($[M]^+$, 24%), 162 (100), 146 (10), 119 (8), 105 (9), 91 (11), 84 (8), 49 (15); MS (CI) *m/z*: 195 ($[M + NH_4]^+$, 30%), 178 ($[MH]^+$, 100), 162 (8), 94 (11), 52 (50), 44 (41); HRMS CI: Calc for $C_{10}H_{12}NO_2$ $[MH^+]$: 178.0863; Found 178.0864.

3-Ethyl-4-methoxyisoindolin-1-one (10). Yield: 0.32 g (1.68 mmol, 84%); mp 150–152 °C; IR (FT) ν/cm^{-1}: 3303, 2922, 1679, 1602, 1493, 1271, 1049.^{1}H NMR (400 MHz, DMSO-d_6) δ (ppm): 7.72 (s, exch., 1H, NH), 7.44 (t, *J* = 8 Hz, 1H, H-6), 7.23 (d, *J* = 8 Hz, 1H, H-7), 7.18 (d, *J* = 8 Hz, 1H, H-5), 4.57 (dd, *J* = 3, 7 Hz, 1H, H-3), 3.87 (s, 3H, OCH_3), 2.08 (ddq, *J* = 3, 14, 7 Hz, 1H, 1H of CH_2), 1.59 (d quintet, *J* = 14, 7 Hz, 1 H, 1 H of CH_2), 0.73 (t, *J* = 7 Hz, 3 H, CH_3); ^{13}C NMR (100 MHz, DMSO-d_6) δ (ppm): 169.7 (s, C-1), 155.1 (s, C-4), 134.8 (s, C-7a), 134.4 (s, C-3a), 130.1 (d, C-6), 115.0 (d, C-7), 113.8 (d, C-5), 56.1 (d, C-3), 55.9 (q, OCH_3), 25.0 (t, CH_2), 9.2 (q, CH_3); MS (EI) *m/z*: 191 ($[M]^+$, 7%), 162 (100), 149 (5), 132 (6); MS (CI) *m/z*: 209 ($[M + NH_4]^+$, 9%), 192 ($[MH]^+$, 100), 176 (12), 162 (28), 94 (11), 59 (33), 44 (40); HRMS (CI): Calc for $C_{11}H_{14}NO_2$ $[MH^+]$:192.1019; Found 192.1018.

3-Butyl-4-methoxyisoindolin-1-one (11). Yield: 0.32 g (1.45 mmol, 72%); mp 130–131 °C (a known compound but no mp reported [76]); IR (FT) ν/cm^{-1}: 3314, 2955, 1678, 1600, 1490, 1276, 1053; ^{1}H NMR (400 MHz, $CDCl_3$) δ (ppm): 7.57 (s, exch., 1H, NH), 7.47–7.41 (m, 2H, H-6 and H-7), 7.22 (dd, *J* = 2, 8 Hz, 1H, H-5), 4.69 (dd, *J* = 3, 7 Hz, 1H, H-3), 3.91 (s, 3H, OCH_3), 2.20 (m, 1H, 1H of $CH_2CH_2CH_2CH_3$), 1.65 (m, 1H, 1H of $CH_2CH_2CH_2CH_3$), 1.39–1.22 (m, 4H, $CH_2CH_2CH_3$), 0.88 (t, *J* = 7 Hz, 3 H, CH_3); ^{13}C NMR (100 MHz, $CDCl_3$) δ (ppm): 171.5 (s, C-1), 155.3 (s, C-4), 135.6 (s, C-7a), 134.2 (s, C-3a), 130.0 (d, C-6), 116.1 (d, C-7), 113.5 (d, C-5), 56.4 (d, C-3), 55.8 (q, OCH_3), 32.4 (t, $CH_2CH_2CH_2CH_3$), 27.9 (t, $CH_2CH_2CH_3$), 23.0 (t, CH_2CH_3), 14.3 (q, CH_3); MS (EI) *m*/*z*: 219 ($[M]^+$, 4%), 188 (3), 162 (100), 148 (4), 132 (5), 119 (4), 91 (4), 77 (6); MS (CI) *m*/*z*: 237 ($[M + NH_4]^+$, 8%), 220 ($[MH]^+$, 100), 204 (9), 190 (11), 164 (18), 162 (17), 86 (17), 72 (38), 58 (41), 44 (39); HRMS (CI): Calc for $C_{13}H_{18}NO_2$ [MH^+]: 220.1332; Found 220.1332.

4-Methoxyisoindolin-1-one (12). Yield; 0.27 g (1.65 mmol, 82%); mp 190–192 °C (Lit [77] >200 °C); IR (FT) ν/cm^{-1}: 3286, 2870, 1682, 1585, 1440, 1266, 1052; ^{1}H NMR (400 MHz, DMSO-d_6) δ (ppm): 8.60 (s, exch., 1H, NH), 7.46 (t, *J* = 8 Hz, 1H, H-6), 7.26 (d, *J* = 8 Hz, 1H, H-7), 7.19 (d, *J* = 8 Hz, 1H, H-5), 4.29 (s, 2H, H-3), 3.88 (s, 3H, OCH_3); ^{13}C NMR (100 MHz, DMSO-d_6) δ (ppm): 170.3 (s, C-1), 154.9 (s, C-4), 134.6 (s, C-3a), 131.8 (s, C-7a), 129.9 (d, C-6), 115.2 (d, C-7), 113.5 (d, C-5), 55.8 (q, OCH_3), 43.0 (t, C-3); MS (EI) *m*/*z*: 163 ($[M]^+$, 100%), 162 (42), 135 (12), 134 (22), 132 (44), 119 (22), 104 (18), 92 (15), 77 (26); MS (CI) *m*/*z*: 327 ($[2 M + 1]^+$, 6%), 181 ($[M + NH_4]^+$, 14) 164 ($[MH]^+$, 100), 134 (10); HRMS (CI): Calc for $C_9H_{10}NO_2$ [MH^+]: 164.0706; Found: 164.0707.

3-(Hydroxydiphenylmethyl)-4-methoxyisoindolin-1-one (13). Yield: 0.56 g (1.62 mmol, 81%); mp 206–208 °C; IR (FT) ν/cm^{-1}: 3301, 2981, 1690, 1599, 1491, 1267, 1038; ^{1}H NMR (400 MHz,

DMSO-d_6) δ (ppm): 7.89 (s, exch., 1H, NH), 7.52–7.18 (m, 11H, H-6 and 2 Ph), 7.10 (d, *J* = 8 Hz, 1H, H-7), 6.94 (d, *J* = 8 Hz, 1H, H-5), 5.84 (s, exch., 1H, OH), 5.76 (s, 1H, H-3), 3.19 (s, 3H, OCH_3); ^{13}C NMR (100 MHz, DMSO-d_6) δ (ppm): 169.7 (s, C-1), 155.0 (s, C-4), 145.8, 144.4 (2 s, C-1 of 2 Ph), 136.2 (s, C-7a), 131.9 (s, C-3a), 130.2 (s, C-6), 128.0, 127.4 (2 d, C-3/C-5 of 2 Ph), 127.1, 126.9 (2 d, C-2/C-6 of 2 Ph), 127.0, 126.8 (2 d, C-4 of 2 Ph), 115.0 (d, C-7), 113.9 (d, C-5), 79.7 (s, C-OH), 64.3 (d, C-3), 55.5 (q, OCH_3); MS (EI) *m*/*z*: 182 (22%), 163 (12), 105 (100), 77 (89), 51 (33); MS (CI) *m*/*z*: 346 ($[MH]^+$, 100%); MS (ES^+) *m*/*z*: 691 ($[2\ MH + 1]^+$, 12%), 346 ($[MH]^+$, 31), 164 (100); HRMS (ES^+): Calc for $C_{22}H_{20}NO_3$ [MH^+]: 346.1438; Found: 346.1440.

3-(1-Hydroxycyclohexyl)-4-methoxyisoindolin-1-one (14). Yield: 0.41 g (1.57 mmol, 78%); mp 206–207 °C; IR (FT) ν/cm^{-1}: 3301, 2952, 1690, 1590, 1470, 1240, 1047; ^{1}H NMR (400 MHz, DMSO-d_6) δ (ppm): 8.45 (s, exch., 1H, NH), 7.43 (t, *J* = 8 Hz, 1H, H-6), 7.25 (d, *J* = 8 Hz, 1H, H-7), 7.22 (d, *J* = 8 Hz, 1H, H-5), 4.46 (s, 1H, H-3), 4.32 (s, exch., 1H, OH), 3.88 (s, 3H, OCH_3), 1.54–0.96 (m, 10H, cyclohexyl group); ^{13}C NMR (100 MHz, DMSO-d_6) δ (ppm): 169.8 (s, C-1), 155.0 (s, C-4), 135.9 (s, C-7a), 132.0 (s, C-3a), 130.2 (d, C-6), 115.5 (d, C-7), 114.6 (d, C-5), 73.5 (s, C-1 of cyclohexyl group), 65.5 (d, C-3), 56.1 (q, OCH_3), 38.8, 33.7 (2 t, C-2/C-6 of cyclohexyl group), 25.8 (t, C-4 of cyclohexyl group), 21.5, 21.4 (2 t, C-3/C-5 of cyclohexyl group); MS (EI) *m*/*z*: 262 ($[MH]^+$, 11%), 262 ($[M]^+$, 2), 244 (32), 243 (100), 214 (33), 212 (21), 188 (48), 177 (19), 176 (34), 175 (55); MS (CI) *m*/*z*: 279 ($[M + NH_4]^+$, 2%), 262 ($[MH]^+$, 29), 244 (4), 181 (35), 164 (100), 134 (12), 116 (88), 98 (12), 55 (13); HRMS (CI): Calc for $C_{15}H_{20}NO_3$ [MH^+]: 262.1438; Found: 262.1435; Anal. Calc for $C_{15}H_{19}NO_3$: C, 68.94; H, 7.33; N, 5.36. Found: C, 69.13; H, 7.34; N, 5.48.

3-(1-Hydroxy-1-methylpentyl)-4-methoxyisoindolin-1-one (15). Yield: 0.41 g (1.56 mmol, 78%); Product **15** was a mixture of two diastereoisomers (**15a** and **15b**) in which many individual NMR signals could be identified, indicating that **15a**:**15b** = 47:53. IR (FT) ν/cm^{-1}: 3490, 3073, 1682,

1593, 1263, 1046; MS (EI) *m*/*z*: 163 (100%), 148 (10), 132 (13), 119 (11), 58 (15), 43 (18); MS (CI) *m*/*z*: 264 (30%) $[MH]^+$, 181 (7), 164 (18), 118 (100); HRMS (CI): Calc for $C_{15}H_{22}NO_3$ $[MH^+]$: 264.1600; Found: 264.1596; Crystallization of the crude product provided crystals of **15a**, mp 186–187 °C. Compound **15a** (α-(*R**)-3-(*S**)- isomer): ^{1}H NMR (400 MHz, $CDCl_3$) δ (ppm): 7.83 (s, exch., 1H, NH), 7.54 (dd, *J* = 1, 8 Hz, 1H, H-7), 7.49 (t, *J* = 8 Hz, 1H, H-6), 7.12 (dd, *J* = 1, 8 Hz, 1H, H-5), 4.72 (s, exch., 1H, OH), 4.08 (s, 1H, H-3), 3.99 (s, 3H, OCH_3), 1.34 (s, 3H, CH_3C-OH), 1.39–1.33 (m, 2H, $CH_2CH_2CH_2CH_3$), 1.29–0.94 (m, 4H, $CH_2CH_2CH_3$), 0.79 (t, *J* = 7 Hz, 3H, CH_2CH_3); ^{13}C NMR (100 MHz, $CDCl_3$) δ (ppm): 171.1 (s, C-1), 154.3 (s, C-4), 135.5 (s, C-7a), 132.5 (s, C-3a), 130.60 (d, C-6), 117.5 (d, C-7), 114.2 (d, C-5), 74.7 (s, C-OH), 66.9 (d, C-3), 56.49 (q, OCH_3), 36.8 (t, $CH_2CH_2CH_2CH_3$), 25.6 (t, $CH_2CH_2CH_3$), 24.8 (q, CH_3C-OH), 23.60 (t, CH_2CH_3), 14.4 (q, CH_2CH_3); Compound **15b** (α-(*R**)-3-(*R**)- isomer): ^{1}H NMR (400 MHz, $CDCl_3$) δ (ppm): 7.56 (s, exch., 1H, NH), 7.45 (dd, *J* = 1, 8 Hz, 1H, H-7), 7.41 (t, *J* = 8 Hz, 1H, H-6), 7.05 (dd, *J* = 1, 8 Hz, 1H, H-5), 4.66 (s, exch., 1H, OH), 4.36 (s, 1H, H-3), 3.92 (s, 3H, OCH_3), 1.59–1.33 (m, 6H, $CH_2CH_2CH_2CH_3$), 0.90 (t, *J* = 7 Hz, 3H, CH_2CH_3), 0.73 (s, 3H, CH_3C-OH); ^{13}C NMR (100 MHz, $CDCl_3$) δ (ppm): 170.9 (s, C-1), 154.1 (s, C-4), 135.4 (s, C-7a), 132.9 (s, C-3a), 130.57 (d, C-6), 117.6 (d, C-7), 114.3 (d, C-5), 74.3 (s, C-OH), 64.7 (d, C-3), 56.52 (q, OCH_3), 40.5 (t, $CH_2CH_2CH_2CH_3$), 25.7 (t, $CH_2CH_2CH_3$), 23.62 (q, CH_3C-OH), 23.2 (t, CH_2CH_3), 14.5 (q, CH_2CH_3).

3-(1-Hydroxy-1-phenylethyl)-4-methoxyisoindolin-1-one (16). Yield: 0.43 g (1.61 mmol, 80%); Product **16** was a mixture of two diastereoisomers (**16a** and **16b**) in which many individual NMR signals could be identified, indicating that **16a**:**16b** = 56:44. IR (FT) ν/cm^{-1}: 3373, 3010, 1687, 1594, 1488, 1367, 1263, 1044; MS (EI) *m*/*z*: 265 (100%), 238 (22), 187 (35); MS (CI) *m*/*z*: 301 ($[M + NH_4]^+$, 3%), 284 ($[MH]^+$, 100), 268 (12); HRMS (CI): Calc for $C_{17}H_{18}NO_3$ $[MH]^+$: 284.1281; Found: 284.1281; Crystallization of the crude product provided crystals of **16a**, mp 235–236 °C. Compound **16a** (α-(*R**)-3-(*R**)- isomer): Anal. Calc for $C_{17}H_{17}NO_3$: C, 72.07; H, 6.05; N, 4.94.

Found: C, 71.98; H, 6.03; N, 4.94; ^{1}H NMR (400 MHz, DMSO-d_6) δ (ppm): 8.61 (s, exch., 1H, NH), 7.32 (t, *J* = 8 Hz, 1H, H-6), 7.26 (d, *J* = 7 Hz, 2H, H-2/H-6 of Ph), 7.15 (t, *J* = 7 Hz, 2H, H-3/H-5 of Ph), 7.11–7.05 (m, 3H, H-5, H-7 and H-4 of Ph), 5.48 (s, exch., 1H, OH), 4.83 (s, 1H, H-3), 3.67 (s, 3H, OCH_3), 1.51 (s, 3H, CH_3); ^{13}C NMR (100 MHz, DMSO-d_6) δ (ppm): 169.8 (s, C-1), 154.7 (s, C-4), 154.2 (s, C-1 of Ph), 135.5 (s, C-7a), 131.9 (s, C-3a), 130.2 (d, C-6), 127.5 (d, C-3/C-5 of Ph), 126.8 (d, C-4 of Ph), 125.6 (d, C-2/C-6 of Ph), 115.3 (d, C-7), 114.2 (d, C-5), 76.0 (s, C-OH), 65.7 (d, C-3), 56.1 (q, OCH_3), 26.6 (q, CH_3); Compound **16b** (α-(*R**)-3-(*S**)- isomer): ^{1}H NMR (400 MHz, $CDCl_3$) δ (ppm): 8.29 (s, exch., 1H, NH), 7.34 (t, *J* = 8 Hz, 1H, H-6), 7.18 (d, *J* = 7 Hz, 2H, H-2/H-6 of Ph), 7.12 (t, *J* = 7 Hz, 2H, H-3/H-5 of Ph), 7.01–6.88 (m, 3H, H-5, H-7 and H-4 of Ph), 5.43 (s, exch., 1H, OH), 4.77 (s, 1H, H-3), 3.72 (s, 3H, OCH_3), 1.74 (s, 3H, CH_3); ^{13}C NMR (100 MHz, DMSO-d_6) δ (ppm): 169.0 (s, C-1), 155.4 (s, C-4), 154.8 (s, C-1 of Ph), 137.1 (s, C-7a), 131.1 (s, C-3a), 129.5 (d, C-6), 128.5 (d, C-3/C-5 of Ph), 128.1 (d, C-4 of Ph), 126.1 (d, C-2/C-6 of Ph), 115.9 (d, C-7), 114.5 (d, C-5), 76.0 (s, C-OH), 65.3 (d, C-3), 55.3 (q, OCH_3), 28.3 (q, CH_3).

3-(Hydroxy(4-methoxyphenyl)methyl)-4-methoxyisoindolin-1-one (17). Yield: 0.49 g (1.63 mmol, 81%); Product **17** was a mixture of two diastereoisomers (**17a** and **17b**) in which many individual NMR signals could be identified, indicating that **17a**:**17b** = 42:58. IR (FT) ν/cm^{-1}: 3304, 2872, 1677, 1604, 1512, 1273, 1048; MS (EI) *m*/*z*: 163 (46%), 136 (54), 135 (100), 119 (8), 107 (10), 92 (11), 77 (15); MS (CI) *m*/*z*: 300 ([MH]$^{+}$, 100%), 284 (8), 237 (24); HRMS (CI): Calc for $C_{17}H_{18}NO_4$ [MH^{+}]: 300.1230; Found: 300.1231; Crystallization of the crude product provided crystals of **17a**, mp 199–201 °C. Compound **17a** (α-(*R**)-3-(*S**)- isomer): ^{1}H NMR (400 MHz, DMSO-d_6) δ (ppm): 8.77 (s, exch., 1H, NH), 7.31 (t, *J* = 8 Hz, 1H, H-6), 7.16 (d, *J* = 8 Hz, 1H, H-5), 6.93 (d, *J* = 8 Hz, 1H, H-7), 6.89 (d, *J* = 9 Hz, 2H, H-2/H-6 of 4-methoxyphenyl group), 6.58 (d, *J* = 9 Hz, 2H, H-3/H-5 of 4-methoxyphenyl group), 5.73 (d, *J* = 3 Hz, exch., 1H, OH), 5.39 (t, *J* = 3 Hz, 1H, C*H*OH), 4.89 (d, *J* = 3 Hz, 1H, H-3), 3.97 (s, 3H, OCH_3), 3.60 (s, 3H, OCH_3); ^{13}C NMR

(100 MHz, DMSO-d_6) δ (ppm): 169.7 (s, C-1), 158.4 (s, C-4 of 4-methoxyphenyl), 155.0 (s, C-4), 134.9 (s, C-1 of 4-methoxyphenyl), 131.6 (s, C-7a), 131.5 (s, C-3a), 130.1 (d, C-6), 128.3 (d, C-2/C-6 of 4-methoxyphenyl), 114.9 (d, C-7), 113.6 (d, C-5), 112.5 (d, C-3/C-5 of 4-methoxyphenyl), 71.6 (d, CHOH), 61.5 (d, C-3), 56.0 (q, OCH_3), 55.1 (q, OCH_3); Compound **17b** (α-(*R**)-3-(*R**)-isomer): ^{1}H NMR (400 MHz, DMSO-d_6) δ (ppm): 8.01 (s, exch., 1H, NH), 7.31 (t, *J* = 8 Hz, 1H, H-6), 7.20 (d, *J* = 8 Hz, 1H, H-5), 6.90 (d, *J* = 8 Hz, 1H, H-7), 6.89 (d, *J* = 9 Hz, 2H, H-2/H-6 of 4-methoxyphenyl group), 6.58 (d, *J* = 9 Hz, 2H, H-3/H-5 of 4-methoxyphenyl group), 5.18 (dd, *J* = 3, 6 Hz, 1H, C*H*OH), 5.12 (d, *J* = 6 Hz, 1H, H-3), 4.76 (d, *J* = 3 Hz, exch., 1H, OH), 3.85 (s, 3H, OCH_3), 3.75 (s, 3H, OCH_3); ^{13}C NMR (100 MHz, DMSO-d_6) δ (ppm): 170.4 (s, C-1), 158.7 (s, C-4 of 4-methoxyphenyl), 155.2 (s, C-4), 135.7 (s, C-1 of 4-methoxyphenyl), 135.4 (s, C-7a), 133.1 (s, C-3a), 130.2 (d, C-6), 127.7 (d, C-2/C-6 of 4-methoxyphenyl), 115.0 (d, C-7), 113.8 (d, C-5), 112.6 (d, C-3/C-5 of 4-methoxyphenyl), 70.6 (d, CHOH), 62.0 (d, C-3), 56.0 (q, OCH_3), 55.5 (q, OCH_3).

3-(Hydroxy(phenyl)methyl)-4-methoxyisoindolin-1-one (18). Yield: 0.43 g (1.60 mmol, 80%); Product **18** was a mixture of two diastereoisomers (**18a** and **18b**) in which many individual NMR signals could be identified, indicating that **18a**:**18b** = 44:56. IR (FT) ν/cm^{-1}: 3201, 3070, 1672, 1603, 1492, 1269, 1053; MS (EI) *m*/*z*: 163 (22%), 132 (5), 116 (15), 105 (14), 86 (24), 84 (30), 51 (56), 49 (100); MS (CI) *m*/*z*: 270 ([MH]$^+$, 15%) 181 (7), 164 (100), 148 (8), 134 (7), 124 (14), 105 (23), 94 (13), 78 (12), 58 (28), 44 (27); HRMS (CI): Calc for $C_{16}H_{16}NO_3$ [MH$^+$]: 270.1125; Found: 270.1125; Crystallization of the crude product provided crystals of **18a**, mp 213–214 °C. Compound **18a** (α-(*R**)-3-(*S**)- isomer): ^{1}H NMR (400 MHz, DMSO-d_6) δ (ppm): 8.79 (s, exch., 1H, NH), 7.31 (t, *J* = 8 Hz, 1H, H-6), 7.16 (d, *J* = 8 Hz, 1H, H-7), 7.03–6.97 (m, 5H, Ph), 6.91 (d, *J* = 8 Hz, 1H, H-5), 5.82 (d, *J* = 3 Hz, exch., 1H, OH), 5.43 (t, 1H, *J* = 3 Hz, C*H*OH), 4.92 (d, *J* = 3 Hz, 1H, H-3), 3.98 (s, 3H, OCH_3); ^{13}C NMR (100 MHz, DMSO-d_6) δ (ppm): 175.0 (s, C-1), 160.3 (s, C-4), 144.8 (s, C-1 of Ph), 140.1 (s, C-7a), 136.8 (s, C-3a), 135.4 (d, C-6), 132.6 (d, C-4 of Ph), 132.5 (d, C-2/C-6 of Ph), 132.4 (d, C-3/C-5 of Ph), 120.1 (d, C-7), 118.8 (d, C-5), 77.2 (d, CHOH),

66.6 (d, C-3), 61.2 (q, OCH_3); Compound **18b** (α-(*R**)-3-((*R**)- isomer): ^{1}H NMR (400 MHz, DMSO-d_6) δ (ppm): 7.79 (s, exch., 1H, NH), 7.45-7.33 (m, 7H, H-6, H-7 and Ph), 7.17 (d, *J* = 8 Hz, 1H, H-5), 5.65 (d, *J* = 3 Hz, exch., 1H, OH), 5.22 (dd, 1H, *J* = 3, 6 Hz, C*H*OH), 4.96 (d, *J* = 6 Hz, 1H, H-3), 3.86 (s, 3H, OCH_3); ^{13}C NMR (100 MHz, DMSO-d_6) δ (ppm): 170.8 (s, C-1), 155.3 (s, C-4), 143.4 (s, C-1 of Ph), 139.7 (s, C-7a), 135.7 (s, C-3a), 130.1 (d, C-6), 128.4 (d, C-2/C-6 of Ph), 127.4 (d, C-4 of Ph), 126.6 (d, C-3/C-5 of Ph), 115.0 (d, C-7), 113.9 (d, C-5), 71.0 (d, CHOH), 61.8 (d, C-3), 56.0 (q, OCH_3).

Isoindolin-1-one (20). Yield: 0.19 g (1.43 mmol, 71%); mp 153–154 °C (Lit [78] 149–151 °C); IR (FT) ν/cm^{-1}: 3288, 2964, 1676, 1570, 1458, 1272, 1052; ^{1}H NMR (500 MHz, $CDCl_3$) δ (ppm): 7.82 (d, *J* = 8 Hz, 1H, H-7), 7.66 (br, exch., 1H, NH), 7.49 (dt, *J* = 2, 8 Hz, 1H, H-5), 7.43–7.40 (m, 2H, H-4 and H-6), 4.41 (s, 2H, H-3); ^{13}C NMR (125 MHz, $CDCl_3$) δ (ppm): 172.0 (s, C-1), 143.7 (s, C-3a), 132.2 (s, C-7a), 131.8 (d, C-5), 128.0 (d, C-4), 123.8 (d, C-7), 123.2 (d, C-6), 45.7 (t, C-3); MS (APCI) *m*/*z*: 134 ($[MH]^+$, 100%); HRMS (APCI): Calc for C_8H_8NO $[MH]^+$: 134.0606; Found: 134.0604.

3-Methylisoindolin-1-one (21). Yield: 0.22 g (1.50 mmol, 75%); mp 117–118 °C (Lit [79] 115–116 °C); IR (FT) ν/cm^{-1}: 3176, 2934, 1678, 1602, 1457, 1265, 1043; ^{1}H NMR (500 MHz, $CDCl_3$) δ (ppm): 8.35 (br, exch., 1H, NH), 7.77 (d, *J* = 8 Hz, 1H, H-7), 7.49 (dt, *J* = 2, 8 Hz, 1H, H-5), 7.38–7.34 (m, 2H, H-4 and H-6), 4.63 (q, *J* = 7 Hz, 1H, H-3), 1.43 (d, *J* = 7 Hz, 3H, CH_3); ^{13}C NMR (125 MHz, $CDCl_3$) δ (ppm): 171.3 (s, C-1), 149.0 (s, C-3a), 131.8 (s, C-7a), 131.7 (d, C-5), 128.0 (d, C-4), 123.6 (d, C-7), 122.2 (d, C-6), 52.8 (d, C-3), 20.2 (q, CH_3); MS (ES^+) *m*/*z*: 295 ($[2 M]^+$, 9%), 189 ($[M + MeCNH]^+$, 100), 148 ($[MH]^+$, 34); HRMS (ES^+): Calc for $C_9H_{10}NO$ $[MH]^+$: 148.0762; Found: 148.0762.

3-Ethylisoindolin-1-one (22). Yield: 0.25 g (1.55 mmol, 77%); mp 103–105 °C (Lit [79] 104–105 °C); IR (FT) ν/cm^{-1}: 3312, 2945, 1677, 1589, 1472, 1267, 1043; ^{1}H NMR (500 MHz, $CDCl_3$) δ (ppm): 8.46 (br, exch., 1H, NH), 7.76 (d, *J* = 8 Hz, 1H, H-7), 7.46 (dt, *J* = 2, 8 Hz, 1H, H-5), 7.35 (t, *J* = 8 Hz, 1H, H-6), 7.21 (d, *J* = 8 Hz, 1H, H-4), 4.52 (dd, *J* = 5, 7 Hz, 1H, H-3), 1.94 (ddq, *J* = 5, 14, 7 Hz, 1H, 1H of CH_2), 1.62 (d quintet, *J* = 14, 7 Hz, 1H, 1H of CH_2), 0.87 (t, *J* = 7 Hz, 3H, CH_3); ^{13}C NMR (125 MHz, $CDCl_3$) δ (ppm): 171.6 (s, C-1), 147.6 (s, C-3a), 132.3 (s, C-7a), 131.7 (d, C-5), 128.5 (d, C-4), 123.6 (d, C-7), 122.4 (d, C-6), 58.2 (d, C-3), 27.3 (t, CH_2), 9.5 (q, CH_3); MS (APCI) *m/z*: 203 ([M + MeCNH]$^+$, 37%), 162 ([MH]$^+$, 100); HRMS (APCI): Calc for $C_{10}H_{12}NO$ [MH]$^+$: 162.0913; found: 162.0919.

3-Butylisoindolin-1-one (23). Yield: 0.29 g (1.53 mmol, 76%); mp 88–89 °C (Lit [79] 88–89 °C); IR (FT) ν/cm^{-1}: 3312, 2960, 1677, 1596, 1475, 1272, 1045; ^{1}H NMR (500 MHz, $CDCl_3$) δ (ppm): 7.77 (d, *J* = 8 Hz, 1H, H-7), 7.66 (br, exch., 1H, NH), 7.49 (dt, *J* = 2, 8 Hz, 1H, H-5), 7.40–7.36 (m, 2H, H-4 and H-6), 4.55 (dd, *J* = 4, 7 Hz, 1H, H-3), 1.88 (m, 1H, 1H of $CH_2CH_2CH_2CH_3$), 1.59 (m, 1H, 1H of $CH_2CH_2CH_2CH_3$), 1.30–1.23 (m, 4H, $CH_2CH_2CH_3$), 0.82 (t, *J* = 7 Hz, 3H, CH_3); ^{13}C NMR (125 MHz, $CDCl_3$) δ (ppm): 171.2 (s, C-1), 147.8 (s, C-3a), 132.0 (s, C-7a), 131.7 (d, C-5), 128.0 (d, C-4), 123.7 (d, C-7), 122.4 (d, C-6), 57.0 (d, C-3), 34.3 (t, $CH_2CH_2CH_2CH_3$), 27.6 (t, $CH_2CH_2CH_3$), 22.6 (t, CH_2CH_3), 13.9 (q, CH_3); MS (ES$^+$) *m/z*: 189 ([M]$^+$, 25%), 177 (56), 132 (100), 104 (23), 72 (32); HRMS (ES$^+$): Calc for $C_{12}H_{15}NO$ [M]$^+$: 189.1154; Found: 189.1154.

3-(Hydroxydiphenylmethyl)isoindolin-1-one (24). Yield: 0.47 g (1.49 mmol, 74%); mp 189–191 °C; IR (FT) ν/cm^{-1}: 3202, 2990, 1678, 1590, 1491, 1252, 1025; ^{1}H NMR (500 MHz, DMSO-*d*$_6$) δ (ppm): 8.20 (br, exch., 1H, NH), 7.60 (d, *J* = 8 Hz, 1H, H-7), 7.52 (t, *J* = 8 Hz, 1H, H-5), 7.35 (d, *J* = 8 Hz, 1H, H-4), 7.30–7.00 (m, 11H, H-6 and 2 Ph), 6.44 (d, *J* = 6 Hz, 1H, H-3), 6.19 (s, exch., 1H, OH); ^{13}C NMR (125 MHz, DMSO-*d*$_6$) δ (ppm): 170.3 (s, C-1), 145.2, 145.1 (2 s, C-1 of 2 Ph), 141.2 (s, C-3a), 134.2 (s, C-7a), 130.9 (d, C-5), 129.6 (d, C-4), 128.3, 128.2 (2 d, C-3/C-5 of

2 Ph), 127.4, 127.3 (2 d, C-2/C-6 of Ph), 126.4, 126.3 (2 d, C-4 of 2 Ph), 124.6 (d, C-7), 122.8 (d, C-6), 79.1 (s, C-OH), 67.5 (d, C-3); MS (ES^+) *m*/*z*: 316 ($[MH]^+$, 3%), 315 ($[M]^+$, 2), 297 (37), 268 (10), 182 (100), 133 (95), 105 (97); HRMS (ES^+): Calc for $C_{21}H_{18}NO_2$ $[MH]^+$: 316.1338; Found: 316.1338.

3-(Hydroxy(phenyl)methyl)isoindolin-1-one (25). Yield: 0.35 g (1.46 mmol, 73%); Product **25** was a mixture of two diastereoisomers (**25a** and **25b**) in which many individual NMR signals could be identified, indicating that **25a**:**25b** = 58:42. IR (FT) ν/cm^{-1}: 3237, 2957, 1669, 1599, 1512, 1050; MS (EI) *m*/*z*: 239 ($[M]^+$, 4%), 221 (100), 220 (51), 193 (33), 165 (89), 149 (86); MS (CI) *m*/*z*: 257 ($[M + NH_4]^+$, 12%), 240 ($[MH]^+$, 100), 224 (24); HRMS (CI): Calc for $C_{15}H_{14}NO_2$ $[MH]^+$: 240.1019; Found: 240.1018. Crystallization of the crude product provided crystals of **25a**, mp 174–175 °C. Compound **25a** (α-(*R**)-3-(*R**)- isomer): ^1H NMR (500 MHz, DMSO-d_6) δ (ppm): 8.69 (br, exch., 1H, NH), 7.48 (dd, *J* = 1, 8 Hz, 1H, H-7), 7.31 (dt, *J* = 1, 8 Hz, 1H, H-5), 7.37 (t, *J* = 8 Hz, 1H, H-6), 7.21 (br, 5H, Ph), 7.07 (d, *J* = 8 Hz, 1H, H-4), 5.92 (d, *J* = 4 Hz, exch., 1H, OH), 4.81 (d, *J* = 6 Hz, 1H, H-3), 4.73 (dd, *J* = 4, 6 Hz, 1H, C*H*OH); ^{13}C NMR (125 MHz, DMSO-d_6) δ (ppm): 169.5 (s, C-1), 144.5 (s, C-1 of Ph), 141.1 (s, C-3a), 133.6 (s, C-7a), 131.0 (d, C-5), 128.3 (d, C-4), 127.8 (d, C-3/C-5 of Ph), 127.7 (d, C-2/C-6 of Ph), 127.6 (d, C-4 of Ph), 124.7 (d, C-7), 122.8 (d, C-6), 74.8 (d, CHOH), 61.9 (d, C-3); Compound **25b** (α-(*R**)-3-(*S**)- isomer): ^1H NMR (500 MHz, DMSO-d_6) δ (ppm): 8.38 (br, exch., 1H, NH), 7.72 (dd, *J* = 1, 8 Hz, 1H, H-7), 7.41 (dt, *J* = 1, 8 Hz, 1H, H-5), 7.30 (t, *J* = 8 Hz, 1H, H-6), 7.21-6.88 (m, 6H, H-4 and Ph), 5.60 (d, *J* = 4 Hz, exch., 1H, OH), 4.83 (t, *J* = 4 Hz, 1H, C*H*OH), 4.62 (d, *J* = 4 Hz, 1H, H-3); ^{13}C NMR (125 MHz, DMSO-d_6) δ (ppm): 169.2 (s, C-1), 144.1 (s, C-1 of Ph), 140.7 (s, C-3a), 134.2 (s, C-7a), 133.2 (d, C-5), 130.6 (d, C-4), 129.4 (d, C-3/C-5 of Ph), 129.1 (d, C-2/C-6 of Ph), 127.0 (d, C-4 of Ph), 124.0 (d, C-7), 122.4 (d, C-6), 74.5 (d, CHOH), 61.5 (d, C-3).

3-(Hydroxy(4-methoxyphenyl)methyl)isoindolin-1-one (26). Yield: 0.42 g (1.56 mmol, 78%); Product **26** was a mixture of two diastereoisomers (**26a** and **26b**) in which many individual NMR signals could be identified, indicating that **26a**:**26b** = 45:55. IR (FT) ν/cm^{-1}: 3301, 2886, 1678, 1602, 1510, 1271, 1042; MS (ES$^+$) *m/z*: 561 ([2 M + Na]$^+$, 12%), 539 ([2 M + 1]$^+$, 100), 311 ([M + MeCNNa]$^+$, 17), 270 ([M + MeCNH]$^+$, 17), 270 ([MH]$^+$, 100), 252 (58), 175 (6); HRMS (ES$^+$): Calc for $C_{16}H_{16}NO_3$ [MH]$^+$: 270.1130; Found: 270.1121. Crystallization of the crude product provided crystals of **26a**, mp 196–198 °C. Compound **26a** (α-(*R**)-3-(*S**)- isomer): ^{1}H NMR (500 MHz, DMSO-d_6) δ (ppm): 8.55 (br, exch., 1H, NH), 7.51–7.37 (m, 4H, H-4, H-5, H-6 and H-7), 7.13 (d, *J* = 9 Hz, 2H, H-2/H-6 of 4-methoxyphenyl group), 6.77 (d, *J* = 9 Hz, 2H, H-3/H-5 of 4-methoxyphenyl group), 5.69 (d, *J* = 4 Hz, exch., 1H, OH), 4.95 (t, *J* = 4 Hz, 1H, C*H*OH), 4.83 (d, *J* = 4 Hz, 1H, H-3), 3.70 (s, 3H, OCH_3); ^{13}C NMR (125 MHz, DMSO-d_6) δ (ppm): 170.0 (s, C-1), 158.8 (s, C-4 of 4-methoxyphenyl), 145.1 (s, C-3a), 133.6 (s, C-1 of 4-methoxyphenyl), 133.1 (s, C-7a), 131.2 (d, C-5), 128.6 (d, C-2/C-6 of 4-methoxyphenyl), 128.3 (d, C-7), 124.5 (d, C-4), 122.9 (d, C-6), 113.3 (d, C-3/C-5 of 4-methoxyphenyl), 73.9 (d, CHOH), 62.5 (d, C-3), 55.4 (q, OCH_3); Compound **26b** (α-(*R**)-3-(*R**)- isomer): ^{1}H NMR (500 MHz, DMSO-d_6): *δ* = 8.67 (br, exch., 1 H, NH), 7.51–7.38 (m, 4 H, H-4, H-5, H-6 and H-7), 7.06 (d, *J* = 9 Hz, 2 H, H-2/H-6 of 4-methoxyphenyl group), 6.79 (d, *J* = 9 Hz, 2 H, H-3/H-5 of 4-methoxyphenyl group), 5.82 (d, *J* = 4 Hz, exch., 1 H, OH), 4.77 (d, *J* = 6 Hz, 1 H, H-3), 4.65 (dd, *J* = 4, 6 Hz, 1 H, C*H*OH), 3.68 (s, 3 H, OCH_3); ^{13}C NMR (125 MHz, DMSO-d_6): *δ* = 170.3 (s, C-1), 159.3 (s, C-4 of 4-methoxyphenyl), 145.0 (s, C-3a), 133.7 (s, C-1 of 4-methoxyphenyl), 133.4 (s, C-7a), 131.1 (d, C-5), 128.9 (d, C-2/C-6 of 4-methoxyphenyl), 128.4 (d, C-7), 124.8 (d, C-4), 122.9 (d, C-6), 113.4 (d, C-3/C-5 of 4-methoxyphenyl), 74.7 (d, CHOH), 62.1 (d, C-3), 55.4 (q, OCH_3).

6-Methoxyisoindolin-1-one (27). Yield: 0.23 g (1.41 mmol, 70%); mp 189–190 °C (a known compound but its mp was not reported [80,81]); IR (FT) ν/cm^{-1}: 3294, 2976, 1678, 1577, 1472, 1265, 1049; ^{1}H NMR (500 MHz, $CDCl_3$) δ (ppm): 8.21 (br, exch., 1H, NH), 7.38 (d, *J* = 2 Hz, 1H,

H-7), 7.35 (d, J = 8 Hz, 1H, H-4), 7.14 (dd, J = 2, 8 Hz, 1H, H-5), 4.42 (s, 2H, H-3), 3.87 (s, 3H, OCH_3); ^{13}C NMR (125 MHz, $CDCl_3$) δ (ppm): 172.2 (s, C-1), 160.0 (s, C-6), 135.9 (s, C-3a), 133.5 (s, C-7a), 124.0 (d, C-4), 120.3 (d, C-5), 106.3 (d, C-7), 55.7 (q, OCH_3), 45.4 (t, C-3); MS (ES^+) m/z: 205 ([M + MeCNH]$^+$, 100%), 164 ([MH]$^+$, 68); HRMS (ES^+): Calc for $C_9H_{10}NO_2$ [MH]$^+$:164.0706; Found: 164.0709.

6-Methoxy-3-methylisoindolin-1-one (28). Yield: 0.27 g (1.52 mmol, 76%); mp 160–161 °C; IR (FT) ν/cm^{-1}: 3165, 2979, 1679, 1600, 1487, 1261, 1046; ^{1}H NMR (500 MHz, $CDCl_3$) δ (ppm): 8.21 (br, exch., 1H, NH), 7.33 (d, J = 2 Hz, 1H, H-7), 7.31 (d, J = 8 Hz, 1H, H-4), 7.13 (dd, J = 2, 8 Hz, 1H, H-5), 4.66 (q, J = 7 Hz, 1H, H-3), 3.86 (s, 3H, OCH_3), 1.48 (d, J = 7 Hz, 3H, CH_3); ^{13}C NMR (125 MHz, $CDCl_3$) δ (ppm): 171.2 (s, C-1), 160.0 (s, C-6), 141.3 (s, C-3a), 133.0 (s, C-7a), 123.1 (d, C-4), 120.2 (d, C-7), 106.3 (d, C-5), 55.7 (q, OCH_3), 52.4 (d, C-3), 20.4 (q, CH_3); MS (ES^+) m/z: 219 (96%), 178 ([MH]$^+$, 100); HRMS (ES^+): Calc for $C_{10}H_{12}NO_2$ [MH]$^+$: 178.0863; Found: 178.0862.

3-Ethyl-6-methoxyisoindolin-1-one (29). Yield: 0.30 g (1.57 mmol, 78%); mp 137–138 °C; IR (FT) ν/cm^{-1}: 3300, 2942, 1678, 1598, 1475, 1270, 1045; ^{1}H NMR (500 MHz, $CDCl_3$) δ (ppm): 8.16 (br, exch., 1H, NH), 7.33 (d, J = 8 Hz, 1H, H-4), 7.31 (s, 1H, H-7), 7.12 (dd, J = 2, 8 Hz, 1H, H-5), 4.56 (dd, J = 5, 7 Hz, 1H, H-3), 3.87 (s, 3H, OCH_3), 1.96 (ddq, J = 5, 14, 7 Hz, 1H, 1H of CH_2), 1.70 (d quintet, J = 14, 7 Hz, 1H, 1H of CH_2), 0.97 (t, J = 7 Hz, 3H, CH_3); ^{13}C NMR (125 MHz, $CDCl_3$) δ (ppm): 171.4 (s, C-1), 160.0 (s, C-6), 139.8 (s, C-3a), 133.6 (s, C-7a), 123.2 (d, C-4), 120.1 (d, C-5), 106.3 (d, C-7), 57.8 (d, C-3), 55.7 (q, OCH_3), 27.5 (t, CH_2), 9.5 (q, CH_3); MS (EI) m/z: 191 ([M]$^+$, 21%), 162 (100), 147 (13), 134 (15), 119 (16); HRMS (EI): Calc for $C_{11}H_{13}NO_2$ [M]$^+$: 191.0946; Found: 191.0943.

3-Butyl-6-methoxyisoindolin-1-one (30). Yield: 0.34 g (1.55 mmol, 77%); mp 145–147 °C; IR (FT) ν/cm^{-1}: 3305, 2967, 1680, 1597, 1477, 1277, 1051; ^{1}H NMR (500 MHz, DMSO-d_6) δ (ppm): 8.71 (br, exch., 1H, NH), 7.44 (d, *J* = 8 Hz, 1H, H-4), 7.15–7.03 (m, 2H, H-5 and H-7), 4.48 (dd, *J* = 3, 7 Hz, 1H, H-3), 3.81 (s, 3H, OCH_3), 1.84 (m, 1H, 1H of C$H_2$$CH_2CH_2CH_3$), 1.48 (m, 1H, 1H of C$H_2$$CH_2CH_2CH_3$), 1.31–1.21 (m, 4H, C$H_2CH_2$$CH_3$), 0.84 (t, *J* = 7 Hz, 3H, CH_3); ^{13}C NMR (125 MHz, DMSO-d_6) δ (ppm): 169.6 (s, C-1), 159.5 (s, C-6), 140.4 (s, C-3a), 134.3 (s, C-7a), 124.3 (d, C-4), 119.4 (d, C-5), 106.5 (d, C-7), 56.0 (d, C-3), 55.9 (q, OCH_3), 34.4 (t, *C*$H_2CH_2CH_2CH_3$), 27.3 (t, *C*$H_2CH_2CH_3$), 22.6 (t, *C*H_2CH_3), 14.3 (q, CH_3); MS (ES^+) *m*/*z*: 261 (100%), 220 ($[MH]^+$, 46), 204 (9), 164 (21); HRMS (ES^+): Calc for $C_{13}H_{18}NO_2$ $[MH]^+$: 220.1338; Found: 220.1347.

3-(Hydroxydiphenylmethyl)-6-methoxyisoindolin-1-one (31). Yield: 0.50 g (1.45 mmol, 72%); mp 159–160 °C; IR (FT) ν/cm^{-1}: 3205, 2994, 1677, 1594, 1493, 1255, 1027; ^{1}H NMR (500 MHz, $CDCl_3$) δ (ppm): 8.12 (br, exch., 1H, NH), 7.71–7.16 (m, 10H, 2 Ph), 6.93 (d, *J* = 2 Hz, 1H, H-7), 6.75 (s, exch., 1H, OH), 6.69 (dd, *J* = 2, 8 Hz, 1H, H-5), 5.78 (d, *J* = 8 Hz, 1H, H-4), 5.40 (s, 1H, H-3), 3.68 (s, 3H, OCH_3); ^{13}C NMR (125 MHz, $CDCl_3$) δ (ppm): 172.0 (s, C-1), 160.0 (s, C-6), 146.5, 144.7 (2 s, C-1 of 2 Ph), 136.4 (s, C-3a), 135.1 (s, C-7a), 128.6, 128.5 (2 d, C-3/C-5 of 2 Ph), 127.4, 127.4 (2 d, C-4 of 2 Ph), 126.3, 126.2 (2 d, C-2/C-6 of Ph), 124.6 (d, C-4), 119.2 (d, C-5), 106.6 (d, C-7), 78.4 (s, C-OH), 64.4 (d, C-3), 55.7 (q, OCH_3); MS (EI) *m*/*z*: 346 ($[M + 1]^+$, 33%), 345 ($[M]^+$, 10), 328 (45), 327 (100); MS (CI) *m*/*z*: 346 ($[MH]^+$, 100%), HRMS (CI): Calc for $C_{22}H_{20}NO_3$ $[MH]^+$: 346.1438; Found: 346.1443.

3-(Hydroxy(4-methoxyphenyl)methyl)-6-methoxyisoindolin-1-one (32). Yield: 0.45 g (1.50 mmol, 75%); Product **32** was a mixture of two diastereoisomers (**32a** and **32b**) in which many individual NMR signals could be identified, indicating that **32a**:**32b** = 62:38. IR (FT) ν/cm^{-1}: 3377, 2824, 1694, 1612, 1511, 1492, 1247, 1036; MS (EI) *m*/*z*: 163 (47%), 136 (52), 135 (100), 119 (5),

107 (20), 92 (26), 77 (53); MS (CI) *m/z*: 300 ($[MH]^+$, 100%), 181 (44); HRMS (CI): Calc for $C_{17}H_{18}NO_4$ $[MH^+]$: 300.1230; Found: 300.1225; Crystallization of the crude product provided crystals of **32a**, mp 172–175 °C. Compound **32a** (α-(*R**)-3-(*R**)- isomer): ^{1}H NMR (400 MHz, DMSO-d_6) δ (ppm): 8.69 (s, exch., 1H, NH), 7.12 (d, *J* = 9 Hz, 2H, H-2/H-6 of 4-methoxyphenyl group), 7.01-6.98 (m, 2H, H-5 and H-7), 6.86 (d, *J* = 8 Hz, 1H, H-4), 6.79 (d, *J* = 9 Hz, 2H, H-3/H-5 of 4-methoxyphenyl group), 5.79 (d, *J* = 4 Hz, exch., 1H, OH), 4.68 (d, *J* = 6 Hz, 1H, H-3), 4.56 (dd, *J* = 4, 6 Hz, 1H, C*H*OH), 3.75 (s, 3H, OCH_3), 3.71 (s, 3H, OCH_3); ^{13}C NMR (100 MHz, DMSO-d_6) δ (ppm): 169.4 (s, C-1), 159.8 (s, C-4 of 4-methoxyphenyl), 158.9 (s, C-6), 136.6 (s, C-1 of 4-methoxyphenyl), 135.1 (s, C-7a), 133.2 (s, C-3a), 128.6 (d, C-2/C-6 of 4-methoxyphenyl), 125.6 (d, C-4), 118.6 (d, C-7), 113.2 (d, C-3/C-5 of 4-methoxyphenyl), 106.1 (d, C-5), 74.9 (d, CHOH), 61.6 (d, C-3), 55.7 (q, OCH_3), 55.3 (q, OCH_3); Compound **32b** (α-(*R**)-3-(*S**)- isomer): ^{1}H NMR (400 MHz, DMSO-d_6) δ (ppm): 8.53 (s, exch., 1H, NH), 7.31 (d, *J* = 8 Hz, 1H, H-4), 7.05 (dd, *J* = 2, 8 Hz, 1H, H-5), 6.86 (d, *J* = 9 Hz, 2H, H-2/H-6 of 4-methoxyphenyl group), 6.80-6.76 (m, 3H, H-7 and H-3/H-5 of 4-methoxyphenyl group), 5.64 (d, *J* = 4 Hz, 1H, H-3), 4.90 (t, *J* = 4 Hz, 1H, C*H*OH), 4.74 (d, *J* = 4 Hz, exch., 1H, OH), 3.77 (s, 3H, OCH_3), 3.69 (s, 3H, OCH_3); ^{13}C NMR (100 MHz, DMSO-d_6) δ (ppm): 169.8 (s, C-1), 159.7 (s, C-4 of 4-methoxyphenyl), 158.7 (s, C-6), 137.1 (s, C-1 of 4-methoxyphenyl), 134.9 (s, C-7a), 133.0 (s, C-3a), 128.5 (d, C-2/C-6 of 4-methoxyphenyl), 125.3 (d, C-4), 118.7 (d, C-7), 113.1 (d, C-3/C-5 of 4-methoxyphenyl), 106.1 (d, C-5), 73.8 (d, CHOH), 61.9 (d, C-3), 55.7 (q, OCH_3), 55.2 (q, OCH_3).

6-Methylisoindolin-1-one (33). Yield: 0.22 g (1.50 mmol, 75%); mp 211–213 °C (Lit [82] 203–205 °C; Lit [83] 205 °C); IR (FT) ν/cm^{-1}: 3286, 2972, 1676, 1570, 1467, 1261, 1047; ^{1}H NMR (500 MHz, DMSO-d_6) δ (ppm): 8.51 (br, exch., 1H, NH), 7.48 (s, 1H, H-7), 7.44 (d, *J* = 8 Hz, 1H, H-5), 7.39 (d, *J* = 8 Hz, 1H, H-4), 4.32 (s, 2H, H-3), 2.39 (s, 3H, CH_3); ^{13}C NMR (125 MHz, DMSO-d_6) δ (ppm): 170.5 (s, C-1), 141.7 (s, C-3a), 137.6 (s, C-6), 133.2 (s, C-7a), 132.6 (d, C-5), 123.8 (d, C-7), 123.4 (d, C-4), 45.1 (t, C-3), 21.3 (q, CH_3); MS (ES^+) *m/z*: 295 ($[2\ M + 1]^+$, 41%).

189 ([M + MeCNH]$^+$, 92), 148 ([MH]$^+$, 100); HRMS (ES$^+$): Calc for $C_9H_{10}NO$ [MH]$^+$: 148.0755; Found: 148.0762; Anal. Calc for C_9H_9NO: C, 73.45; H, 6.16; N, 9.52. Found: C, 73.32; H, 6.14; N, 9.41.

3,6-Dimethylisoindolin-1-one (34). Yield: 0.25 g (1.55 mmol, 78%); mp 161–162 °C; IR (FT) ν/cm^{-1}: 3187, 2986, 1677, 1598, 1478, 1266, 1042; ^{1}H NMR (500 MHz, $CDCl_3$) δ (ppm): 8.59 (br, exch., 1H, NH), 7.45 (s, 1H, H-7), 7.44 (d, *J* = 8 Hz, 1H, H-5), 7.39 (d, *J* = 8 Hz, 1H, H-4), 4.56 (q, *J* = 6 Hz, 1H, H-3), 2.38 (s, 3H, CH_3), 1.33 (d, *J* = 6 Hz, 3H, CH_3CH); ^{13}C NMR (125 MHz, $CDCl_3$) δ (ppm): 169.4 (s, C-1), 146.9 (s, C-3a), 137.7 (s, C-6), 132.8 (d, C-5), 132.7 (s, C-7a), 123.3 (d, C-7), 122.9 (d, C-4), 51.9 (d, C-3), 21.3 (q, CH_3), 20.8 (q, CH_3); MS (ES$^+$) *m*/*z*: 323 ([2 M + 1]$^+$, 22%), 203 ([M + MeCNH]$^+$, 96), 162 ([MH]$^+$, 100); HRMS (ES$^+$): Calc for $C_{10}H_{12}NO$ [MH]$^+$: 162.0925; Found: 162.0925.

3-Ethyl-6-methylisoindolin-1-one (35). Yield: 0.26 g (1.49 mmol, 75%); mp 165-166 °C; IR (FT) ν/cm^{-1}: 3307, 2949, 1679, 1597, 1470, 1272, 1049; ^{1}H NMR (500 MHz, $CDCl_3$) δ (ppm): 8.27 (br, exch., 1H, NH), 7.58 (s, 1H, H-7), 7.28 (d, *J* = 8 Hz, 1H, H-5), 7.23 (d, *J* = 8 Hz, 1H, H-4), 4.49 (dd, *J* = 5, 7 Hz, 1H, H-3), 2.36 (s, 3H, CH_3), 1.91 (ddq, *J* = 5, 14, 7 Hz, 1H, 1H of CH_2), 1.62 (d quintet, *J* = 14, 7 Hz, 1H, 1H of CH_2), 0.89 (t, *J* = 7 Hz, 3H, CH_3CH_2); ^{13}C NMR (125 MHz, $CDCl_3$) δ (ppm): 171.7 (s, C-1), 144.8 (s, C-3a), 137.9 (s, C-6), 132.7 (d, C-5), 132.4 (s, C-7a), 123.8 (d, C-4), 122.1 (d, C-7), 58.0 (d, C-3), 27.4 (t, CH_2), 21.3 (q, CH_3), 9.5 (q, CH_3CH_2); MS (APCI) *m*/*z*: 175 ([M]$^+$, 11%), 146 (100), 118 (12); HRMS (APCI): Calc for $C_{11}H_{13}NO$ [M]$^+$: 175.0997; Found: 175.1000.

3-(1-Hydroxycyclohexyl)-6-methylisoindolin-1-one (36). Yield: 0.35 g (1.43 mmol, 72%); mp 235–237 °C; IR (FT) ν/cm^{-1}: 3307, 2943, 1681, 1596, 1472, 1246, 1043; ^{1}H NMR (500 MHz, DMSO-d_6) δ (ppm): 8.52 (br, exch., 1H, NH), 7.59 (d, *J* = 8 Hz, 1H, H-5), 7.41 (s, 1H, H-7), 7.35

(d, *J* = 8 Hz, 1H, H-4), 4.63 (s, exch., 1H, OH), 4.28 (s, 1H, H-3), 2.38 (s, 3H, CH_3), 1.61–0.93 (m, 10H, cyclohexyl); ^{13}C NMR (125 MHz, DMSO-d_6) δ (ppm): 170.5 (s, C-1), 142.8 (s, C-3a), 137.6 (s, C-6), 133.8 (s, C-7a), 132.2 (d, C-5), 125.4 (d, C-4), 123.0 (d, C-7), 72.6 (s, C-1 of cyclohexyl group), 65.7 (d, C-3), 34.9, 30.4 (2 t, C-2/C-6 of cyclohexyl group), 25.9 (t, C-4 of cyclohexyl group), 21.31, 21.1 (2 t, C-3/C-5 of cyclohexyl group), 21.27;(q, CH_3); MS (ES^+) *m*/*z*: 491 ($[2 M + 1]^+$, 10%), 287 (79), 246 ($[MH]^+$, 100), 228 (18); HRMS (ES^+): Calc for $C_{15}H_{20}NO_2$ $[MH]^+$: 264.1494; Found: 246.1498; Anal. Calc for $C_{15}H_{19}NO_2$: C, 73.44; H, 7.81; N 5.71. Found: C, 73.81; H, 7.85; N 5.80.

3-(Hydroxydiphenylmethyl)-6-methylisoindolin-1-one (37). Yield: 0.56 g (1.70 mmol, 85%); mp 236–264 °C; IR (FT) ν/cm^{-1}: 3278, 3000, 1678, 1591, 1493, 1251, 1040; ^{1}H NMR (500 MHz, DMSO-d_6) δ (ppm): 8.12 (br, exch., 1H, NH), 7.61–7.19 (m, 11H, 2 Ph and H-7), 7.11 (d, *J* = 8 Hz, 1H, H-5), 6.26 (d, *J* = 8 Hz, 1H, H-4), 5.81 (s, exch., 1H, OH), 5.72 (s, 1H, H-3), 2.33 (s, 3H, CH_3); ^{13}C NMR (125 MHz, DMSO-d_6) δ (ppm): 172.4 (s, C-1), 145.5, 145.2 (2 s, C-1 of 2 Ph), 142.4 (s, C-3a), 137.6 (s, C-6), 134.4 (s, C-7a), 131.8 (d, C-5), 128.3, 128.2 (2 d, C-3/C-5 of 2 Ph), 127.3, 127.2 (2 d, C-4 of 2 Ph), 127.1, 127.0 (2 d, C-2/C-6 of 2 Ph), 124.3 (d, C-4), 123.0 (d, C-7), 79.0 (s, C-OH), 63.4 (d, C-3), 21.2 (q, CH_3); MS (ES^+) *m*/*z*: 659 ($[2 M + 1]^+$, 12%), 371 ($[M + MeCNH]^+$, 71), 330 ($[MH]^+$, 100), 312 (36), 189 (8); HRMS (ES^+): Calc for $C_{22}H_{20}NO_2$ $[MH]^+$: 330.1494; Found: 330.1487.

3-(1-Hydroxy-1-methylpentyl)-6-methylisoindolin-1-one (38). Yield: 0.38 g (1.54 mmol, 77%); Product **38** was a mixture of two diastereoisomers (**38a** and **38b**) in which many individual NMR signals could be identified, indicating that **38a**:**38b** = 55:45. IR (FT) ν/cm^{-1}: 3467, 3042, 1680, 1590, 1269, 1041; MS (ES^+) *m*/*z*: 517 ($[2 M + Na]^+$, 9%), 495 ($[2 M + 1]^+$, 44), 289 ($[M + MeCNH]^+$, 32), 248 ($[MH]^+$, 100), 230 (41), 148 (9), 100 (8); HRMS (ES^+): Calc for $C_{15}H_{22}NO_2$ $[MH]^+$: 248.1651; Found: 248.1646. Crystallization of the crude product provided crystals of **38a**,

mp 88–89 °C. Compound **38a** (α-(*R**)-3-(*R**)- isomer):^{1}H NMR (500 MHz, DMSO-d_6) δ (ppm): 8.47 (br, exch., 1H, NH), 7.53 (d, *J* = 8 Hz, 1H, H-5), 7.42 (s, 1H, H-7), 7.35 (d, *J* = 8 Hz, 1H, H-4), 4.79 (s, exch., 1H, OH), 4.39 (br, 1H, H-3), 2.38 (s, 3H, CH_3), 1.33–1.09 (m, 6H, $C\mathit{H_2}C\mathit{H_2}C\mathit{H_2}CH_3$), 1.06 (s, 3H, $C\mathit{H_3}$C-OH), 0.79 (t, *J* = 7 Hz, 3H, $C\mathit{H_3}CH_2$); ^{13}C NMR (125 MHz, DMSO-d_6) δ (ppm): 170.3 (s, C-1), 142.8 (s, C-3a), 137.7 (s, C-6), 133.9 (s, C-7a), 132.4 (d, C-5), 125.0 (d, C-4), 123.1 (d, C-7), 73.6 (s, C-OH), 65.5 (d, C-3), 36.3 (t, $\mathit{C}H_2CH_2CH_2CH_3$), 25.4 (t, $\mathit{C}H_2CH_2CH_3$), 24.6 (q, CH_3), 23.3 (t, $\mathit{C}H_2CH_3$), 21.3 (q, CH_3), 14.5 (q, $CH_2\mathit{C}H_3$); Compound **38b** (α-(*R**)-3-(*S**)- isomer): ^{1}H NMR (500 MHz, DMSO-d_6) δ (ppm): 8.59 (br, exch., 1H, NH), 7.56 (d, *J* = 8 Hz, 1H, H-5), 7.46 (s, 1H, H-7), 7.39 (d, *J* = 8 Hz, 1H, H-4), 4.88 (s, exch., 1H, OH), 4.23 (br, 1H, H-3), 2.26 (s, 3H, CH_3), 1.33–1.09 (m, 6H, $C\mathit{H_2}C\mathit{H_2}C\mathit{H_2}CH_3$), 1.00 (s, 3H, $C\mathit{H_3}$C-OH), 0.89 (t, *J* = 7 Hz, 3H, $C\mathit{H_3}CH_2$); ^{13}C NMR (125 MHz, DMSO-d_6) δ (ppm): 170.8 (s, C-1), 142.7 (s, C-3a), 137.6 (s, C-6), 133.5 (s, C-7a), 132.5 (d, C-5), 124.9 (d, C-4), 122.8 (d, C-7), 73.8 (s, C-OH), 66.6 (d, C-3), 34.2 (t, $\mathit{C}H_2CH_2CH_2CH_3$), 25.3 (t, $\mathit{C}H_2CH_2CH_3$), 24.8 (q, CH_3), 23.6 (t, $\mathit{C}H_2CH_3$), 21.5 (q, CH_3), 14.4 (q, $CH_2\mathit{C}H_3$).

3-(Hydroxy(phenyl)methyl)-6-methylisoindolin-1-one (39). Yield: 0.40 g (1.58 mmol, 79%); Product **39** was a mixture of two diastereoisomers (**39a** and **39b**) in which many individual NMR signals could be identified, indicating that **39a**:**39b** = 54:48. IR (FT) ν/cm^{-1}: 3312, 2943, 1678, 1602, 1500, 1272, 1045; MS (APCI) *m*/*z*: 529 ([2 M + Na]$^+$, 61%), 507 ([2 M$^+$ + 1], 93), 317 (15), 295 (22), 254 ([MH]$^+$, 89), 236 (100), 117 (12), 100 (22); HRMS (APCI): Calc for $C_{16}H_{16}NO_2$ [MH]$^+$: 254.1181; Found: 254.1182. Crystallization of the crude product provided crystals of **39a**, mp 228–229 °C. Compound **39a** (α-(*R**)-3-(*R**)- isomer): ^{1}H NMR (500 MHz, DMSO-d_6) δ (ppm): 8.02 (br, exch., 1H, NH), 7.41 (d, *J* = 2 Hz, 1H, H-7), 7.31–7.22 (m, 5H, Ph), 7.03 (dd, *J* = 2, 8 Hz, 1H, H-5), 6.33 (d, *J* = 8 Hz, 1H, H-4), 5.62 (d, *J* = 4 Hz, exch., 1H, OH), 4.67 (d, *J* = 7 Hz, 1H, H-3), 4.39 (dd, *J* = 4, 7 Hz, 1H, C*H*OH), 2.32 (s, 3H, CH_3); ^{13}C NMR (125 MHz, $CDCl_3$) δ (ppm): 170.3 (s, C-1), 141.1 (s, C-3a), 140.9 (s, C-1 of Ph), 137.9 (s, C-6), 133.3 (s, C-7a), 131.9 (d, C-5),

128.2 (d, C-7), 128.1 (d, C-3/C-5 of Ph), 127.7 (d, C-2/C-6 of Ph), 124.1 (d, C-4 of Ph), 123.2 (d, C-4), 76.9 (d, CHOH), 62.7 (d, C-3), 21.3 (q, CH_3); Compound **39b** (α-(*R**)-3-(*S**)- isomer): ^{1}H NMR (500 MHz, DMSO-d_6) δ (ppm): 8.00 (br, exch., 1H, NH), 7.50 (d, *J* = 2 Hz, 1H, H-7), 7.31–7.22 (m, 5H, Ph), 7.07 (dd, *J* = 2, 8 Hz, 1H, H-5), 6.28 (d, *J* = 8 Hz, 1H, H-4), 5.52 (d, *J* = 4 Hz, exch., 1H, OH), 4.73 (d, *J* = 4 Hz, 1H, H-3), 4.29 (t, *J* = 4 Hz, 1H, C*H*OH), 2.26 (s, 3H, CH_3); ^{13}C NMR (125 MHz, $CDCl_3$) δ (ppm): 170.7 (s, C-1), 142.4 (s, C-3a), 142.1 (s, C-1 of Ph), 137.2 (s, C-6), 133.5 (s, C-7a), 132.5 (d, C-5), 128.6 (d, C-7), 128.0 (d, C-3/C-5 of Ph), 126.8 (d, C-2/C-6 of Ph), 125.7 (d, C-4 of Ph), 124.3 (d, C-4), 74.7 (d, CHOH), 61.4 (d, C-3), 21.2 (q, CH_3).

3-(Hydroxy(4-methoxyphenyl)methyl)-6-methylisoindolin-1-one (40). Yield: 0.47 g (1.66 mmol, 83%); Product **40** was a mixture of two diastereoisomers (**40a** and **40b**) in which many individual NMR signals could be identified, indicating that **40a**:**40b** = 42:58. IR (FT) ν/cm^{-1}: 3301, 2896, 1677, 1601, 1519, 1271, 1042; MS (ES$^+$) *m*/*z*: 589 ([2 M + Na]$^+$, 37%), 567 ([2 M + 1]$^+$, 64), 325 ([M + MeCNH]$^+$, 65), 284 ([MH]$^+$, 100), 266 (46); HRMS (ES$^+$): Calc for $C_{17}H_{18}NO_3$ [MH]$^+$: 284.1287; Found: 284.1290. Crystallization of the crude product provided crystals of **40a**, mp 211–213 °C.

Compound **40a** (α-(*R**)-3-(*S**)- isomer): ^{1}H NMR (500 MHz, DMSO-d_6) δ (ppm): 8.47 (br, exch., 1H, NH), 7.31–7.29 (m, 3H, H-4, H-5 and H-7), 7.12 (d, *J* = 9, 2H, H-2/H-6 of 4-methoxyphenyl), 6.76 (d, *J* = 9, 2H, H-3/H-5 of 4-methoxyphenyl), 5.65 (d, *J* = 4 Hz, exch., 1H, OH), 4.92 (t, *J* = 4 Hz, 1H, C*H*OH), 4.77 (d, *J* = 4 Hz, 1H, H-3), 3.69 (s, 3H, OCH_3), 2.33 (s, 3H, CH_3); ^{13}C NMR (125 MHz, DMSO-d_6) δ (ppm): 170.1 (s, C-1), 158.8 (s, C-4 of 4-methoxyphenyl), 142.3 (s, C-3a), 137.7 (s, C-6), 133.8 (s, C-7a), 133.2 (s, C-1 of 4-methoxyphenyl), 132.1 (d, C-5), 128.6 (d, C-2/C-6 of 4-methoxyphenyl), 124.2 (d, C-7), 123.1 (d, C-4), 113.3 (d, C-3/C-5 of 4-methoxyphenyl), 73.9 (d, CHOH), 62.3 (d, C-3), 55.4 (q, OCH_3), 21.3 (q, CH_3); Compound **40b** (α-(*R**)-3-(*R**)- isomer): ^{1}H NMR (500 MHz, DMSO-d_6) δ (ppm): 8.63 (br, exch., 1H, NH), 7.31–7.25 (m, 3H, H-4, H-5 and H-7), 7.13 (d, *J* = 9, 2H, H-2/H-6 of 4-methoxyphenyl), 6.79 (d, *J* = 9, 2H, H-3/H-5 of

4-methoxyphenyl), 5.79 (d, *J* = 4 Hz, exch., 1H, OH), 4.71 (d, *J* = 6 Hz, 1H, H-3), 4.58 (dd, *J* = 4, 6 Hz, 1H, C*H*OH), 3.70 (s, 3H, OCH_3), 2.32 (s, 3H, CH_3); ^{13}C NMR (125 MHz, DMSO-d_6) δ (ppm): 169.2 (s, C-1), 158.5 (s, C-4 of 4-methoxyphenyl), 141.3 (s, C-3a), 137.2 (s, C-6), 133.4 (s, C-7a), 132.8 (s, C-1 of 4-methoxyphenyl), 131.5 (d, C-5), 128.4 (d, C-2/C-6 of 4-methoxyphenyl), 122.6 (d, C-7), 122.5 (d, C-4), 112.4 (d, C-3/C-5 of 4-methoxyphenyl), 74.8 (d, CHOH), 61.3 (d, C-3), 55.5 (q, OCH_3), 21.1 (q, CH_3).

3,3-Dibutylisoindolin-1-one (41). This compound was obtained as a side product, along with compound **40**, when 1-bromobutane was used as the electrophile; Yield: 25 mg (0.10 mmol, 5%); mp 88–89 °C; IR (FT) ν/cm^{-1}: 3300, 2960, 1681, 1591, 1473, 1273, 1046; ^{1}H NMR (500 MHz, $CDCl_3$) δ (ppm): 7.74 (d, *J* = 8 Hz, 1H, H-7), 7.47 (t, *J* = 8 Hz, 1H, H-5), 7.36 (t, *J* = 8 Hz, 1H, H-6), 7.23 (d, *J* = 8 Hz, 1H, H-4), 7.16 (br s, exch., 1H, NH), 1.85–1.72 (m, 4H, 2 $CH_2CH_2CH_2CH_3$), 1.18–1.09 (m, 8H, 2 $CH_2CH_2CH_3$), 0.74 (t, *J* = 7 Hz, 6H, 2 CH_3); ^{13}C NMR (125 MHz, $CDCl_3$) δ (ppm): 170.6 (s, C-1), 150.8 (s, C-3a), 133.3 (s, C-7a), 131.8 (d, C-5), 127.8 (d, C-4), 123.7 (d, C-7), 121.2 (d, C-6), 65.1 (s, C-3), 39.0 (t, 2 $CH_2CH_2CH_2CH_3$), 25.6 (t, 2 $CH_2CH_2CH_3$), 22.8 (t, 2 CH_2CH_3), 13.8 (q, 2 CH_3); MS (ES$^+$) *m*/*z*: 513 ([2 M + Na]$^+$, 12%), 491 ([2 M + 1]$^+$, 3), 309 ([M + MeCNNa]$^+$, 24), 287 ([M + MeCNH]$^+$, 97), 246 ([MH]$^+$, 100); HRMS (ES$^+$): Calc for $C_{16}H_{24}NO$ [MH]$^+$: 246.1858; Found: 246.1858.

References related to the characterization data

76. Kiyoshi, S. H. *Jpn. Kokai Tokkyo Koho*, *Jp* 01026559, **1989**; *Chem. Abstr.*, **1990**, *112*, 35677.
77. Pellicciari, R.; Camaioni, E.; Costantino, G.; Formentini, L.; Sabbatini, P.; Venturoni, F.; Eren, G.; Bellocchi, D.; Chiarugi, A.; Moroni, F. *ChemMedChem* **2008**, *3*, 914-923.
78. Broadus, K. M.; Kass, S. R. *J. Org. Chem.* **2000**, *65*, 6566-6571.
79. Deniau, E.; Enders, D. *Tetrahedron* **2001**, *57*, 2581-2588.

80. Shen, Z.; Ramamoorthy, P. S.; Hatzenbuhler, N. T.; Evrard, D. A.; Childers, W.; Harrison, B. L.; Chlenov, M.; Hornby, G.; Smith, D. L.; Sullivan, K. M.; Schechter, L. E.; Andree, T. H. *Bioorg. Med. Chem. Lett*. **2010**, *20*, 222-227.

81. Powers, J. J.; Favor, D. A.; Rankin, T.; Sharma, R.; Pandit, C.; Jeganathan, A.; Maiti, S. N. *Tetrahedron Lett*. **2009**, *50*, 1267-1269.

82. Anderson, P. S.; Christy, M. E.; Colton, C. D.; Halczenko, W.; Ponticello, G. S.; Shepard, K. L. *J. Org. Chem*. 1979, **44**, 1519-1533.

83. de Diesbach, H. *Helv. Chim. Acta* **1940**, *23*, 1232-1252.

NMR Spectra for Some of the Synthesised Compounds

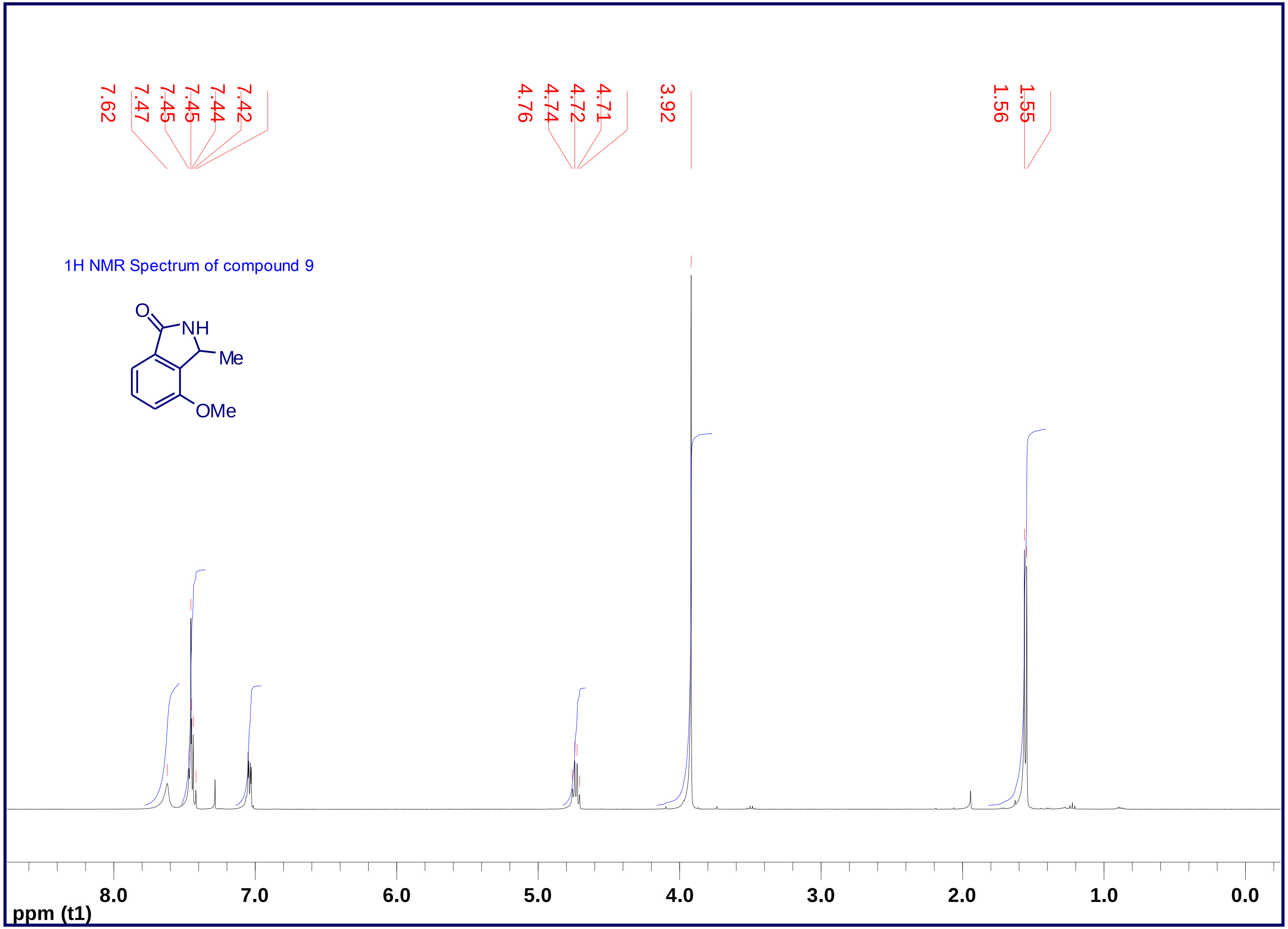
7.62
7.47
7.45
7.45
7.44
7.42
4.76
4.74
4.72
4.71
3.92
1.56
1.55
1H NMR Spectrum of compound 9
O
NH
Me
OMe
8.0
7.0
6.0
5.0
4.0
3.0
2.0
1.0
0.0
ppm (t1)

Expansion - 1H NMR Spectrum of compound 9

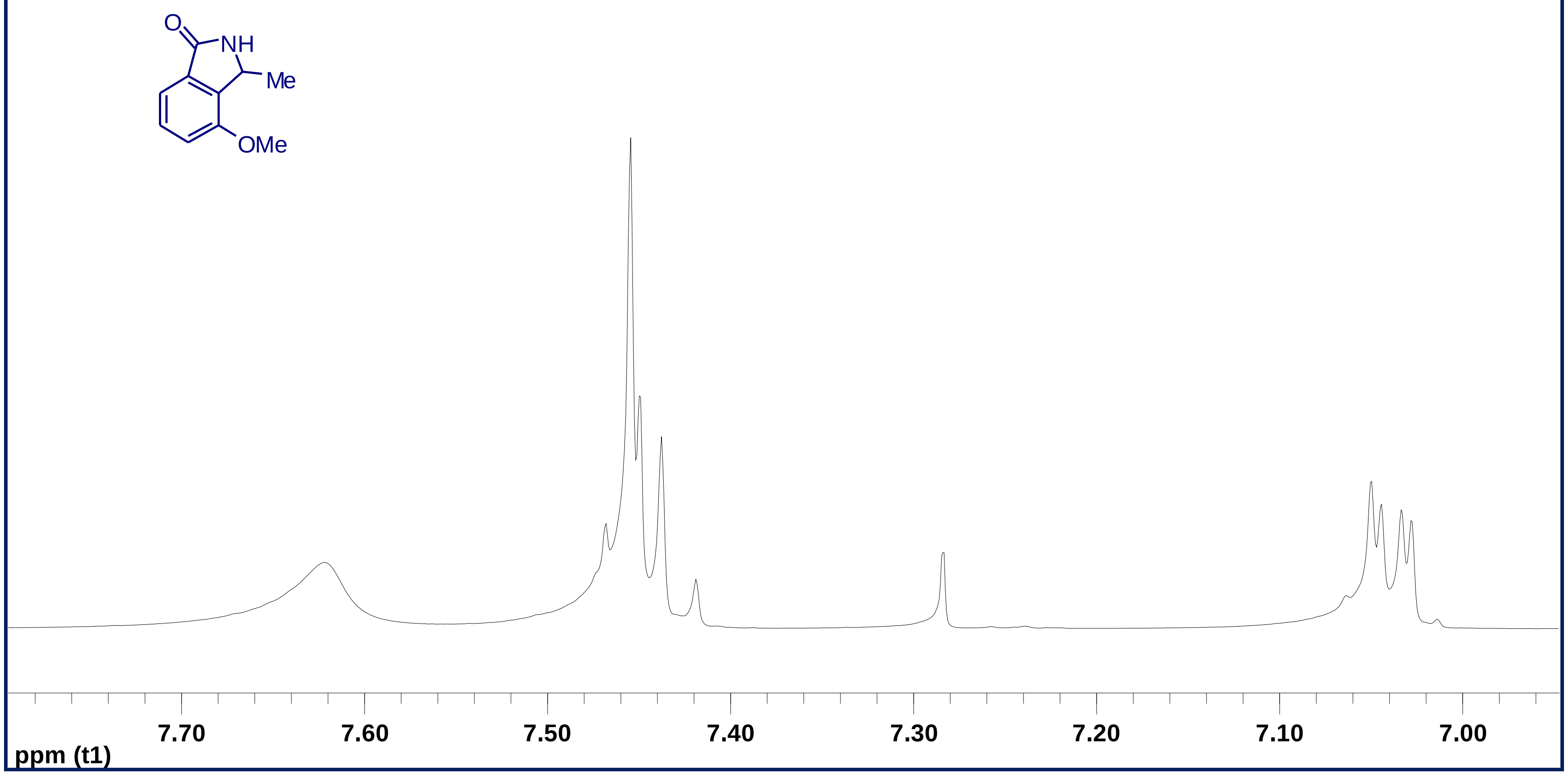

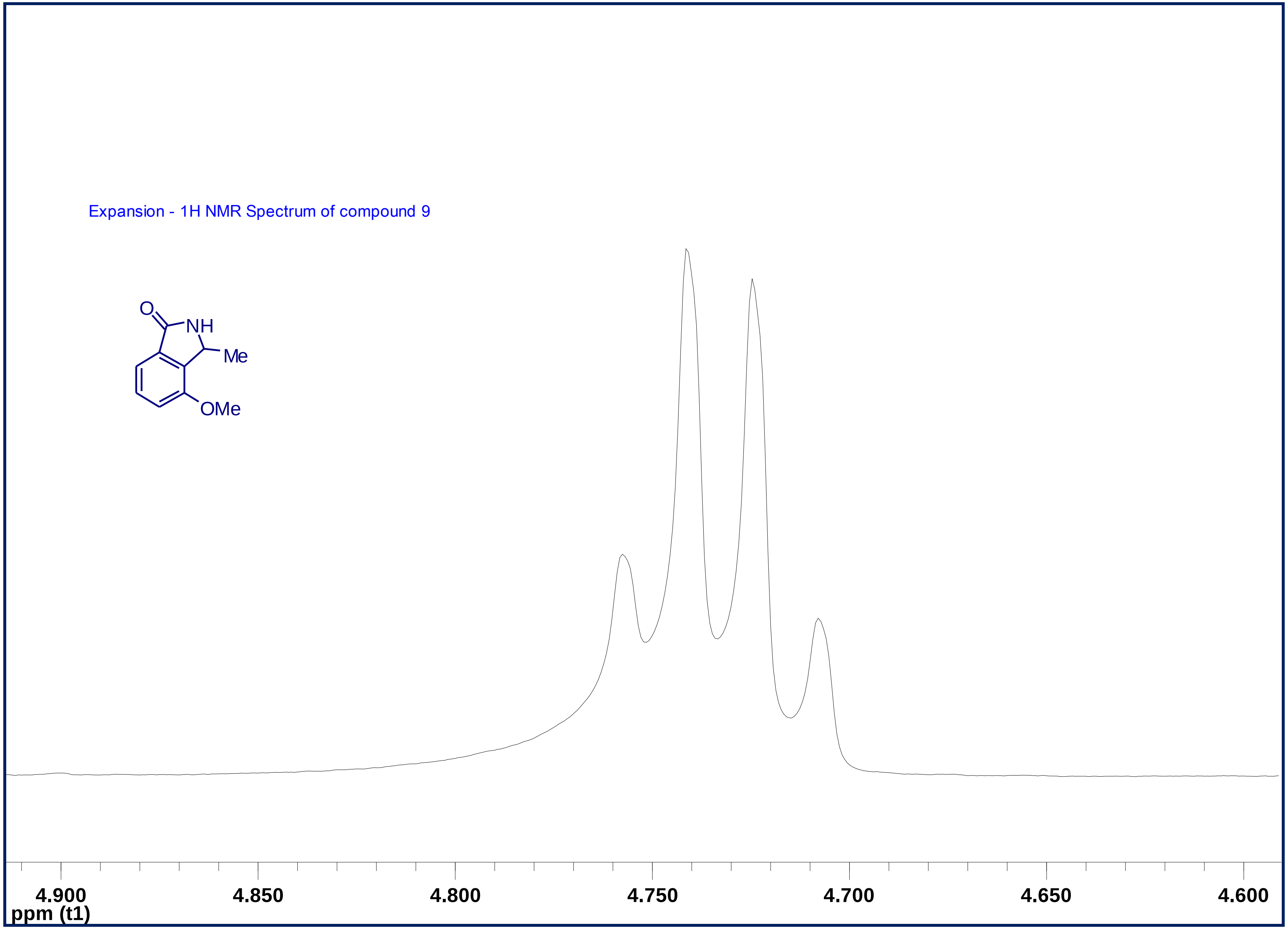
Expansion - 1H NMR Spectrum of compound 9
O
NH
Me
OMe
4.900
4.850
4.800
4.750
4.700
4.650
4.600
ppm (t1)

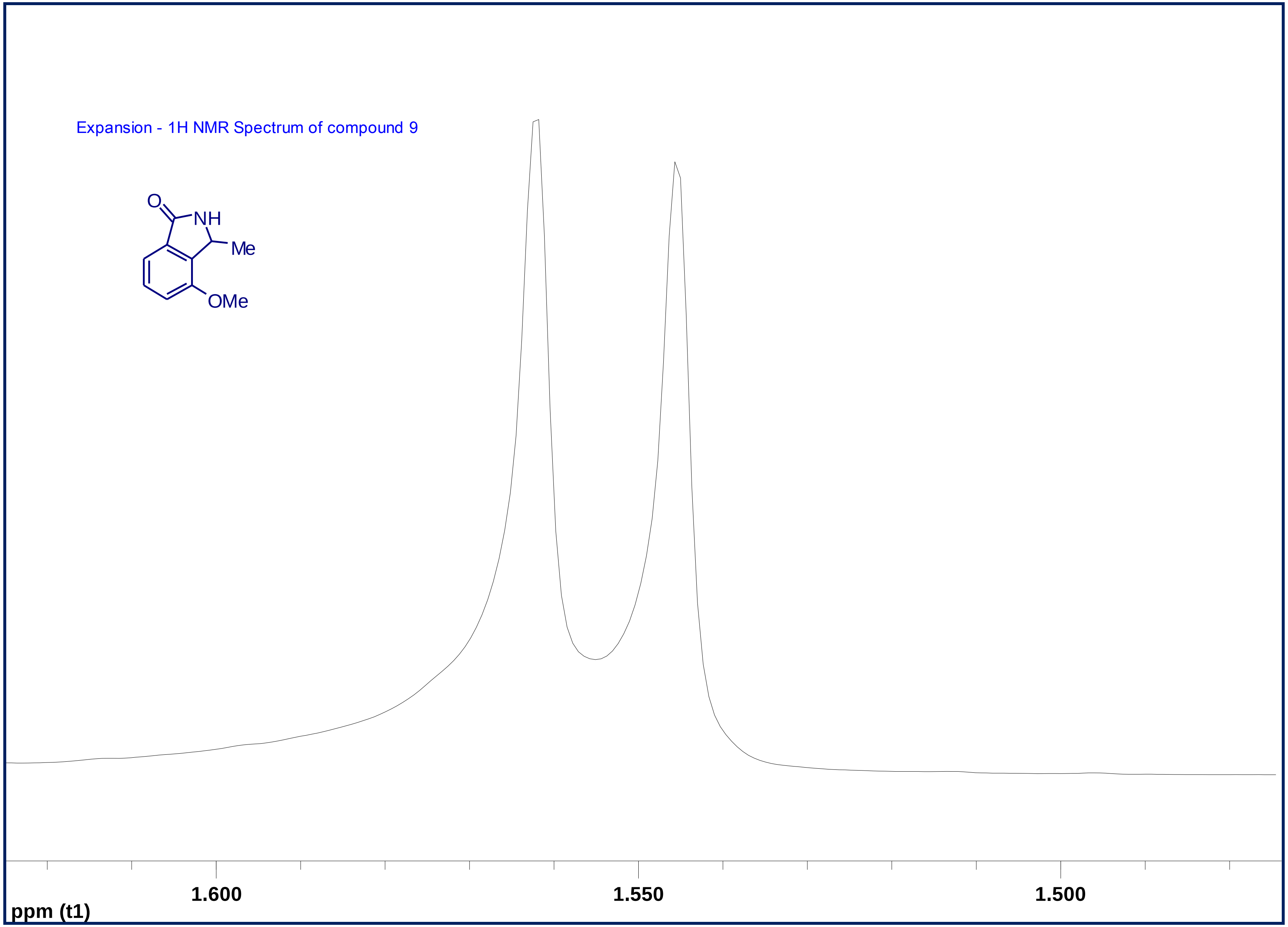

Expansion - 1H NMR Spectrum of compound 9
O
NH
Me
OMe
1.600
1.550
1.500
ppm (t1)

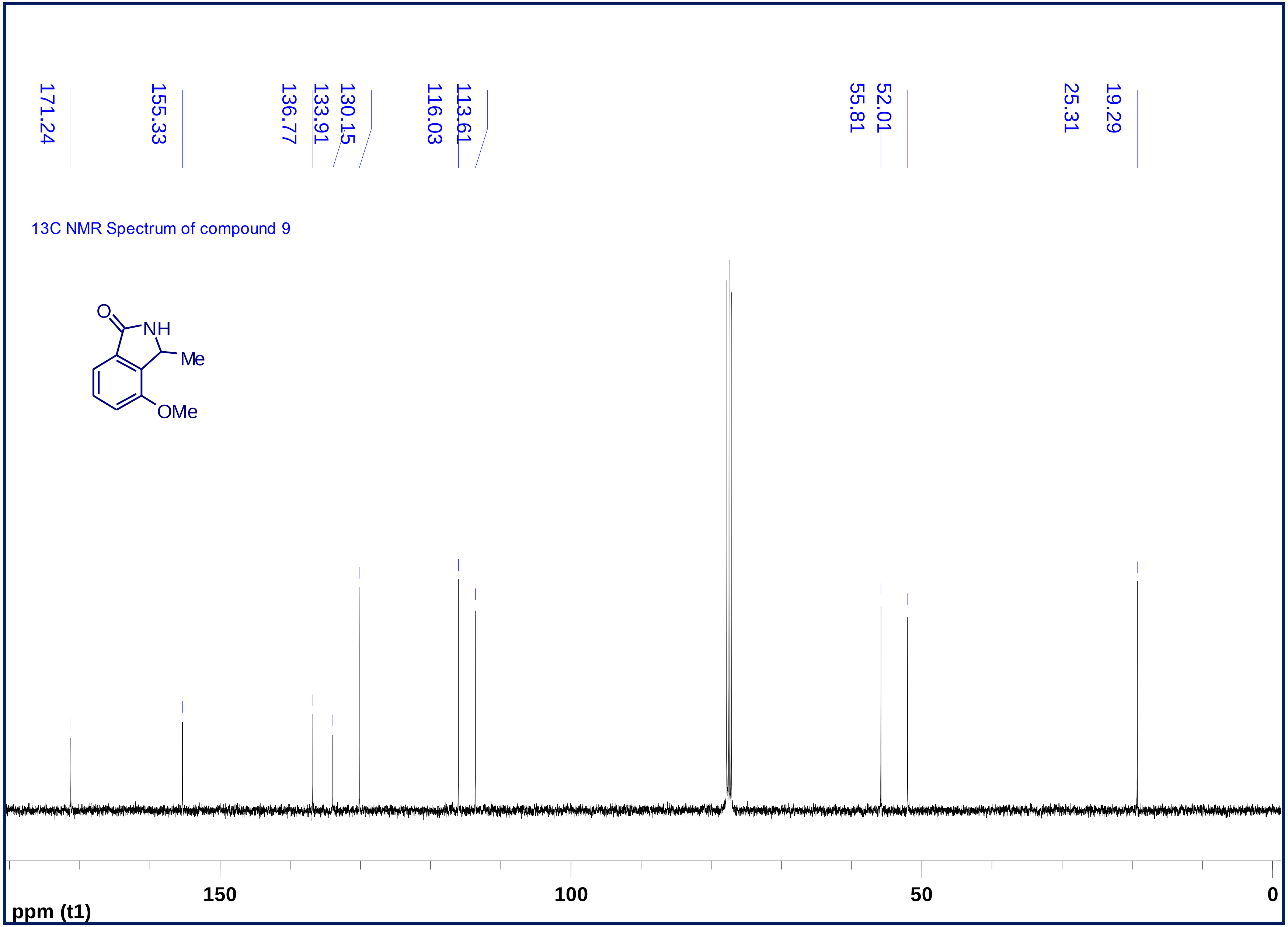
171.24
155.33
136.77
133.91
130.15
116.03
113.61
55.81
52.01
25.31
19.29
13C NMR Spectrum of compound 9
O
NH
Me
OMe
150
100
50
0
ppm (t1)

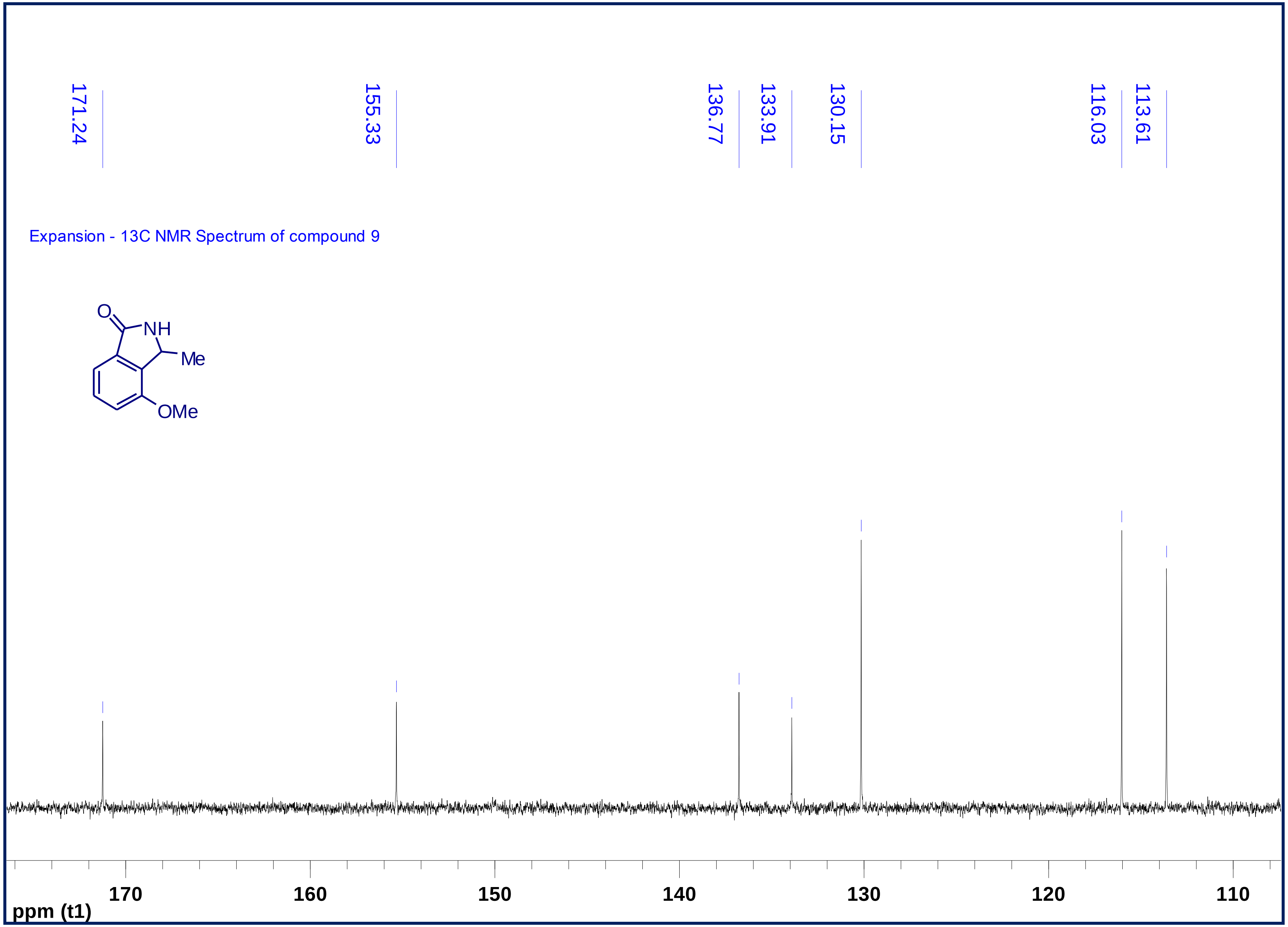

171.24
155.33
136.77
133.91
130.15
116.03
113.61
Expansion - 13C NMR Spectrum of compound 9
O
NH
Me
OMe
170
160
150
140
130
120
110
ppm (t1)

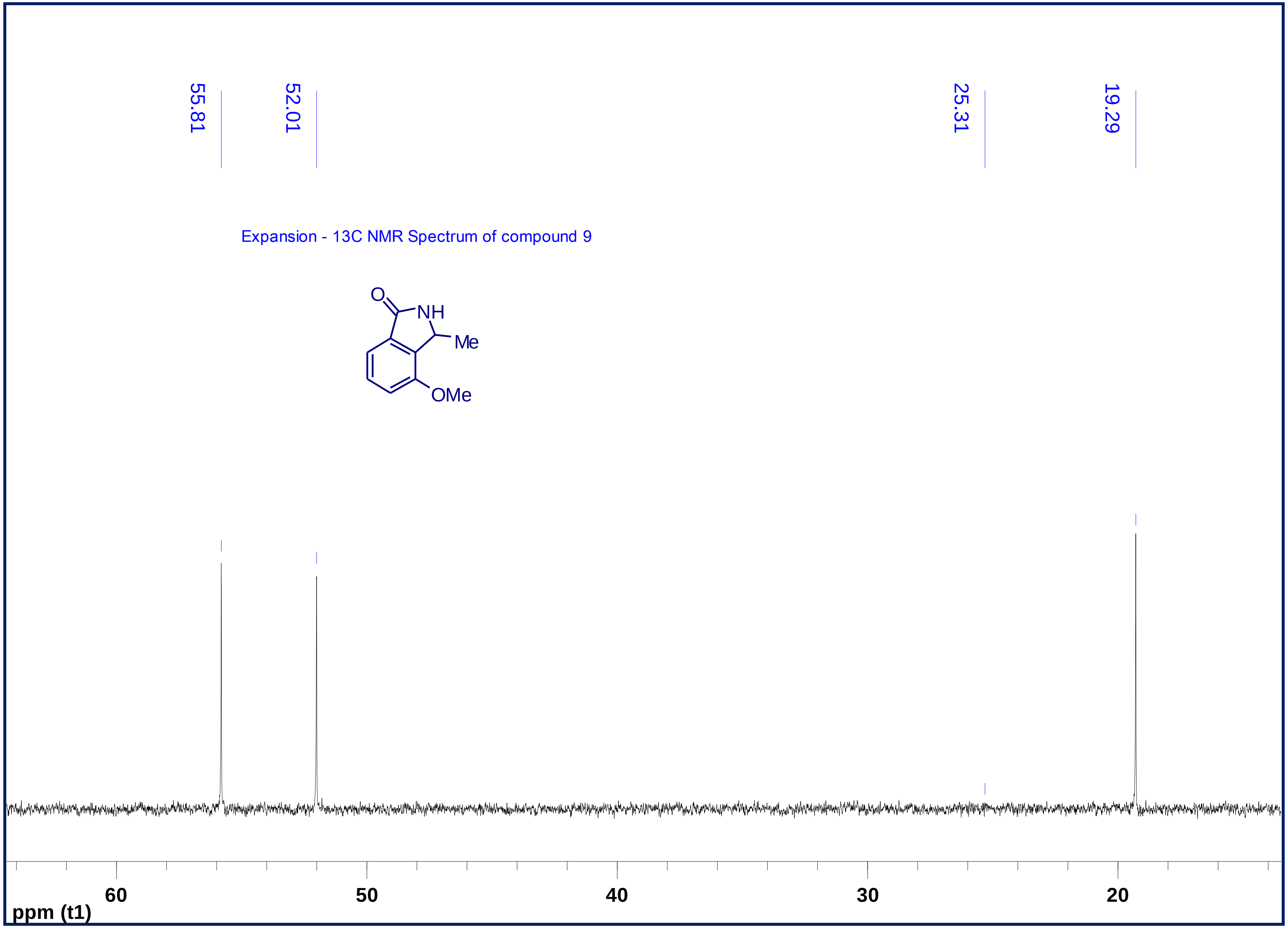

55.81
52.01
25.31
19.29
Expansion - 13C NMR Spectrum of compound 9
O
NH
Me
OMe
60
50
40
30
20
ppm (t1)

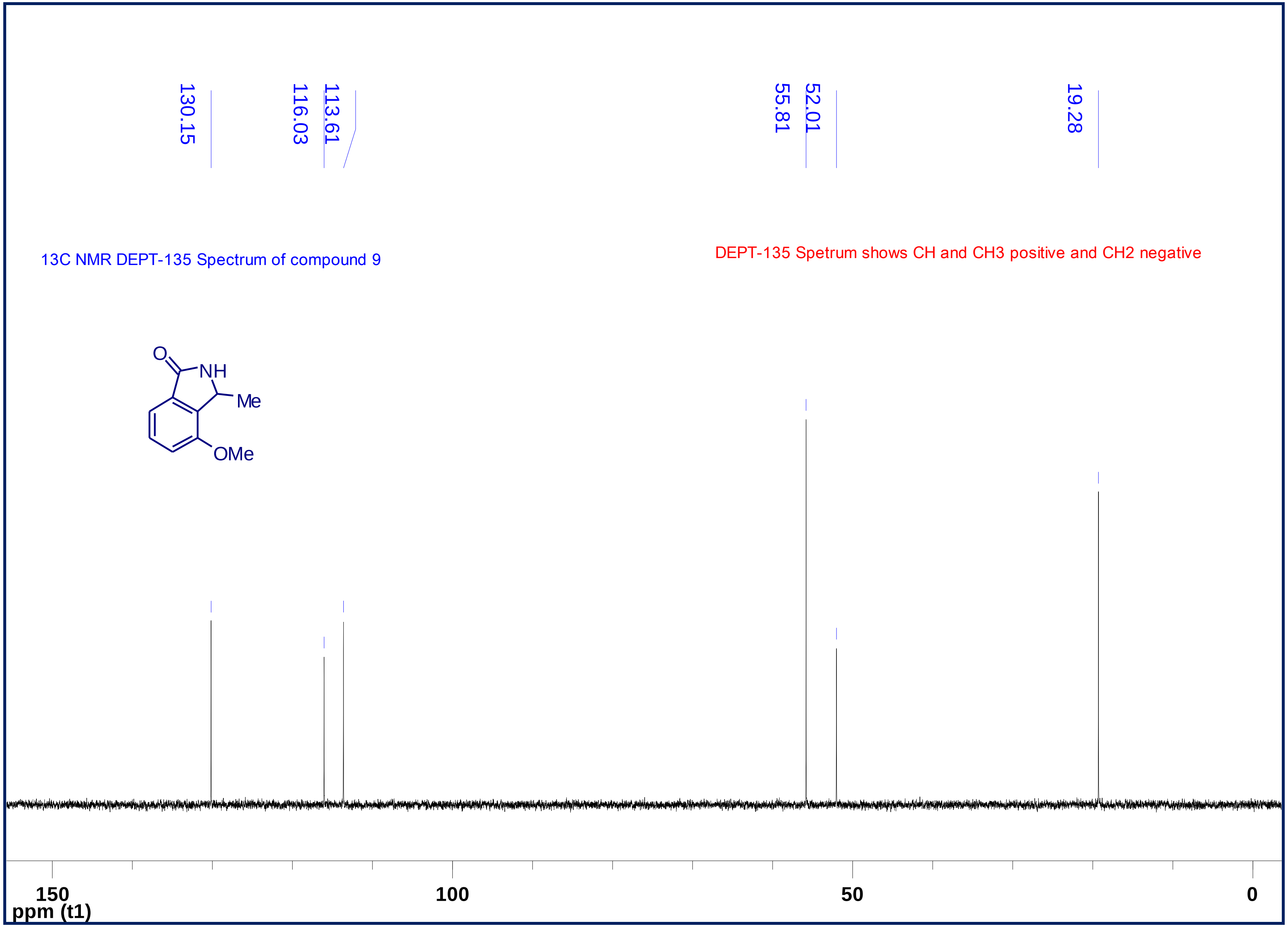
130.15
116.03
113.61
55.81
52.01
19.28
13C NMR DEPT-135 Spectrum of compound 9
DEPT-135 Spetrum shows CH and CH3 positive and CH2 negative
O
NH
Me
OMe
150
100
50
0
ppm (t1)

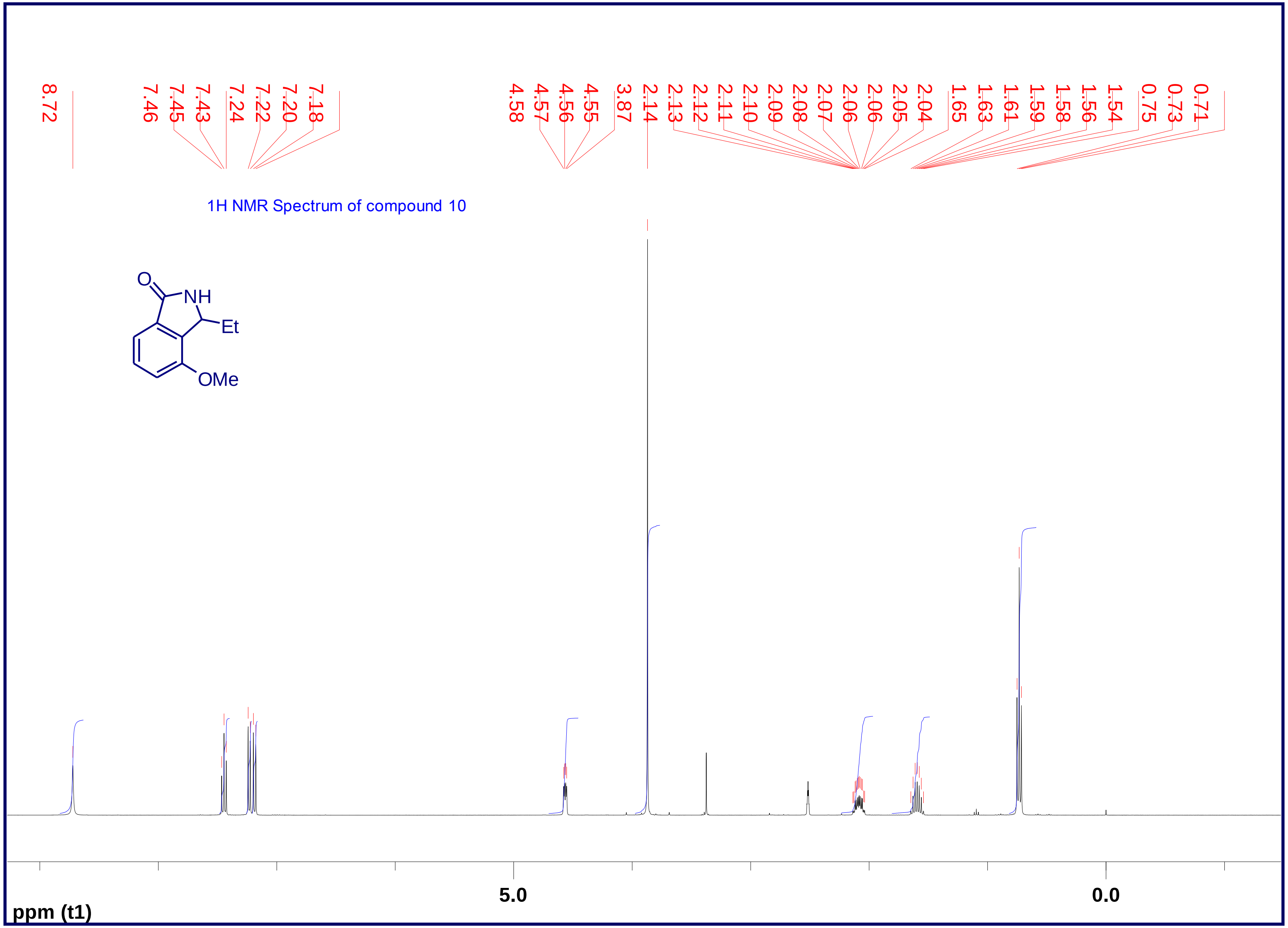

8.72
7.46
7.45
7.43
7.24
7.22
7.20
7.18
4.58
4.57
4.56
4.55
3.87
2.14
2.13
2.12
2.11
2.10
2.09
2.08
2.07
2.06
2.06
2.05
2.04
1.65
1.63
1.61
1.59
1.58
1.56
1.54
0.75
0.73
0.71
1H NMR Spectrum of compound 10
O
NH
Et
OMe
5.0
0.0
ppm (t1)

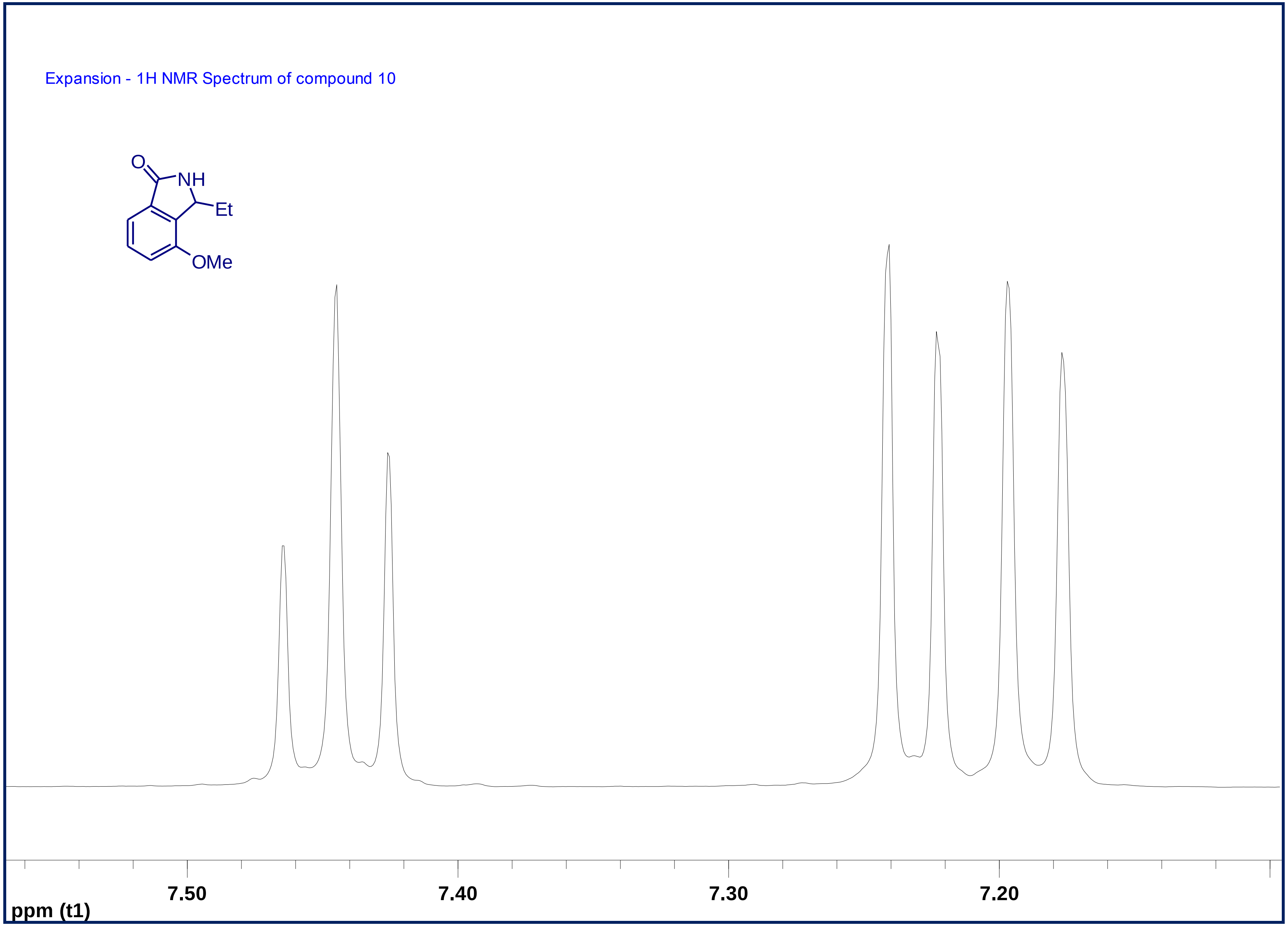
Expansion - 1H NMR Spectrum of compound 10
O
NH
Et
OMe
7.50
7.40
7.30
7.20
ppm (t1)

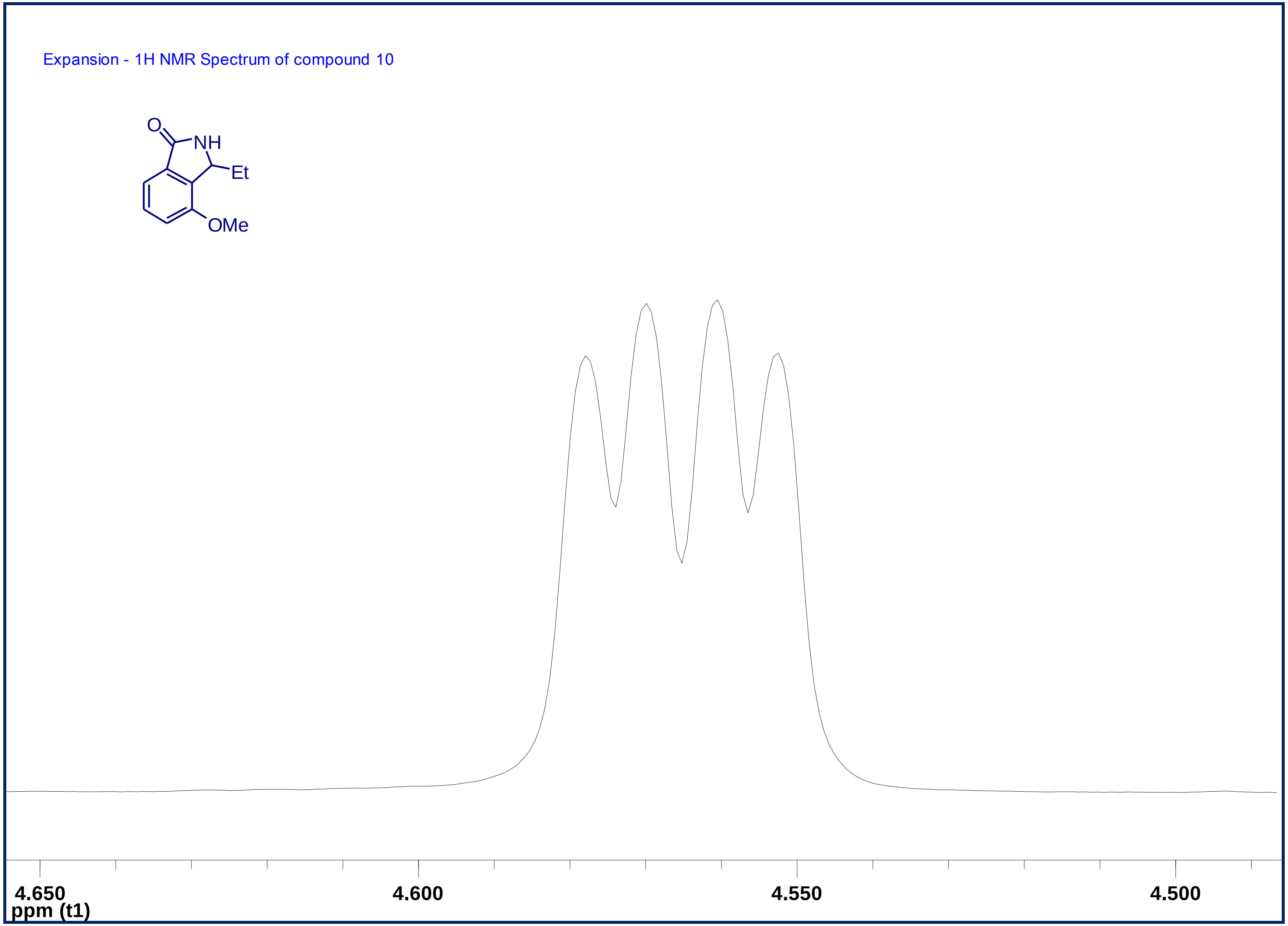

Expansion - 1H NMR Spectrum of compound 10
O
NH
Et
OMe
4.650
4.600
4.550
4.500
ppm (t1)

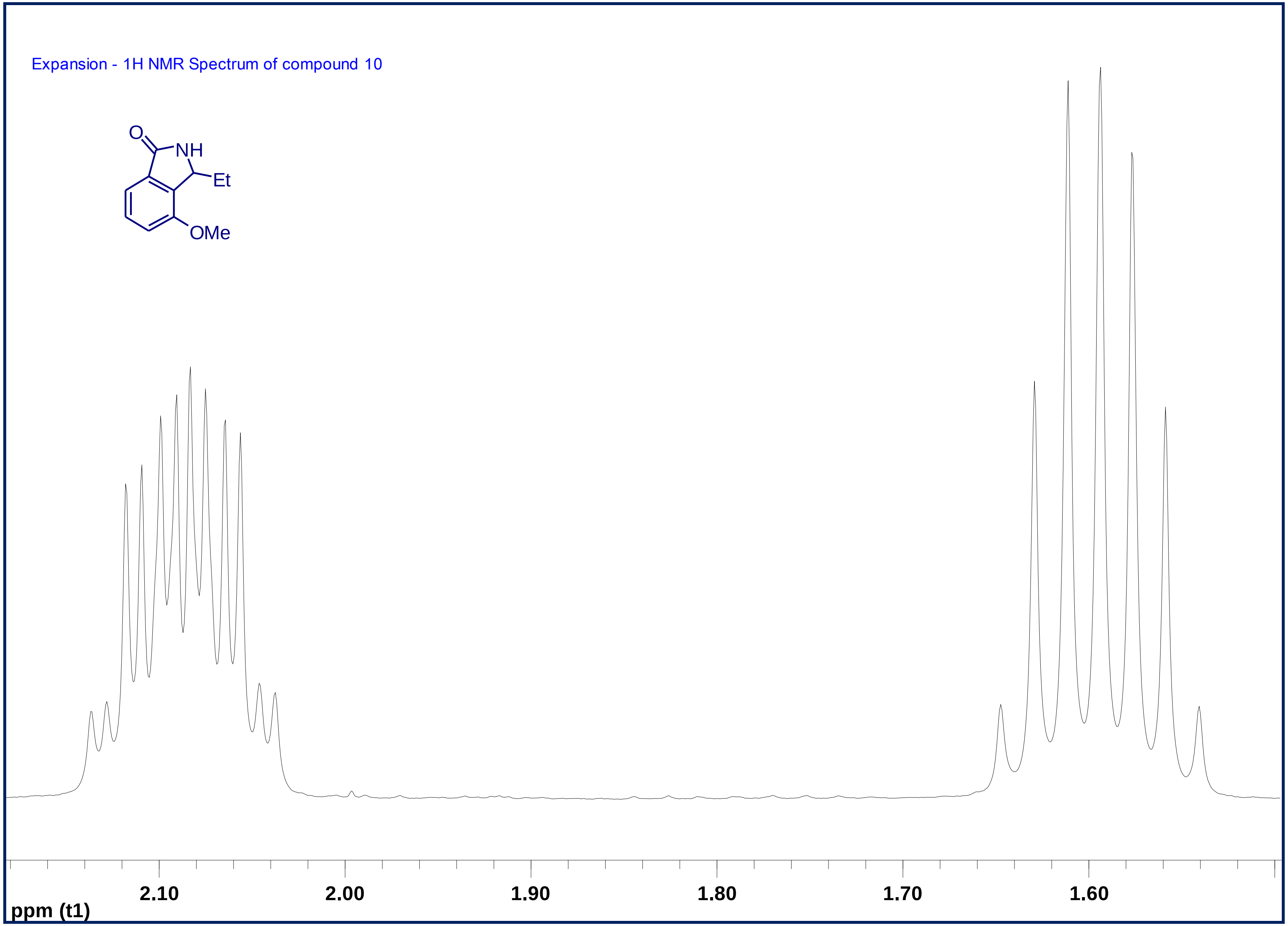

Expansion - 1H NMR Spectrum of compound 10
O
NH
Et
OMe
2.10
2.00
1.90
1.80
1.70
1.60
ppm (t1)

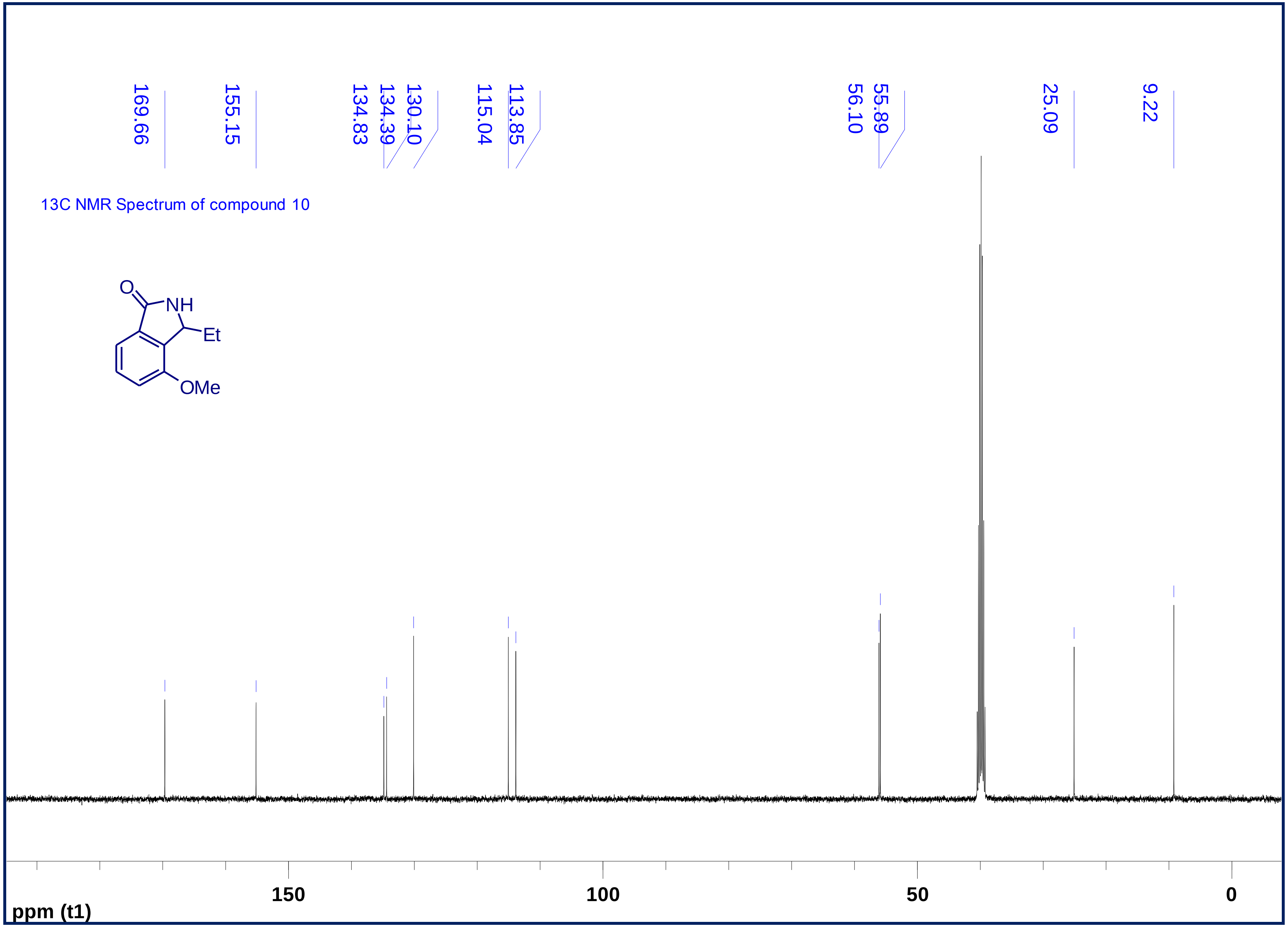
169.66
155.15
134.83
134.39
130.10
115.04
113.85
56.10
55.89
25.09
9.22
13C NMR Spectrum of compound 10
O
NH
Et
OMe
150
100
50
0
ppm (t1)

Expansion - 13C NMR Spectrum of compound 10

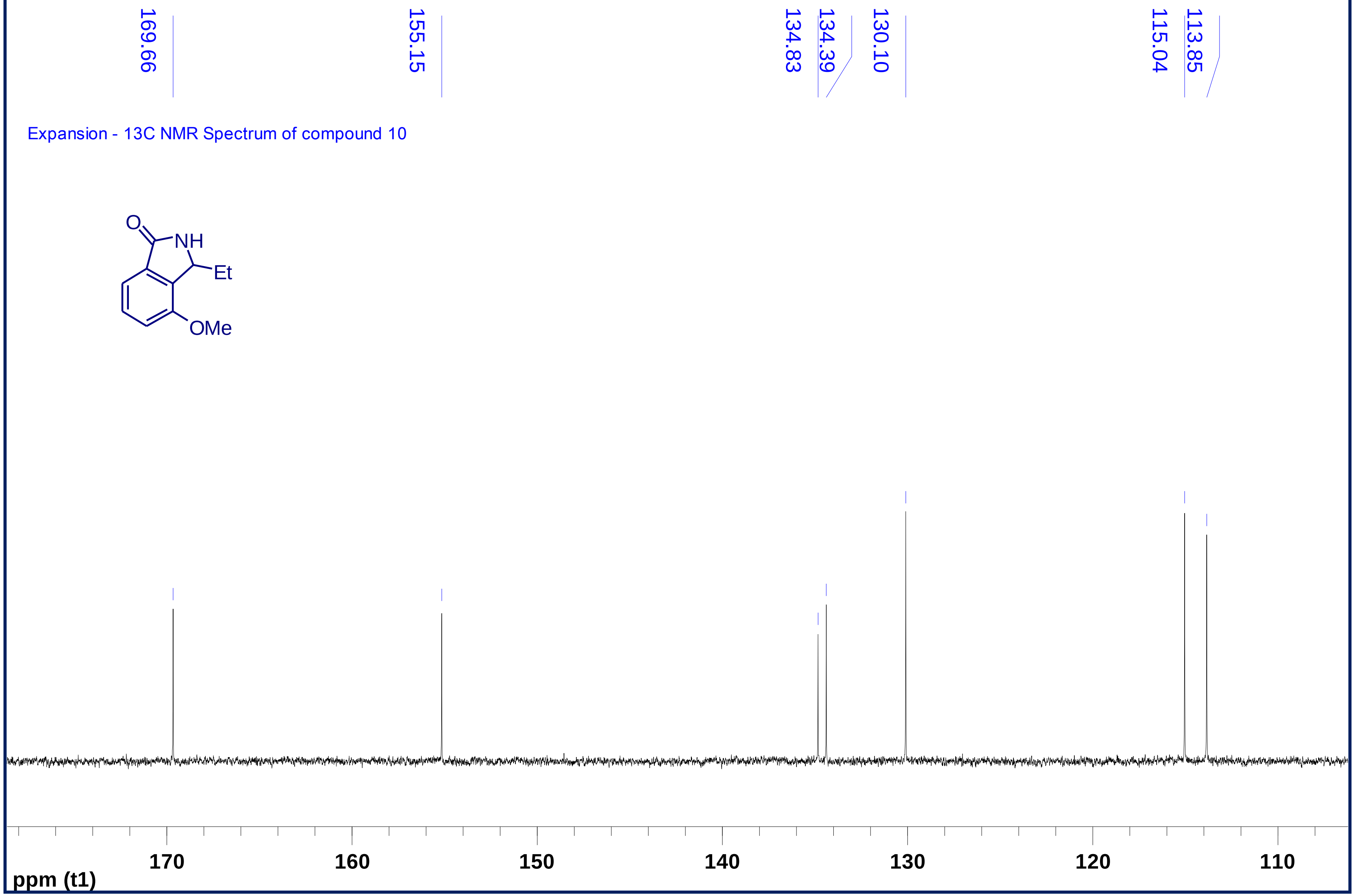

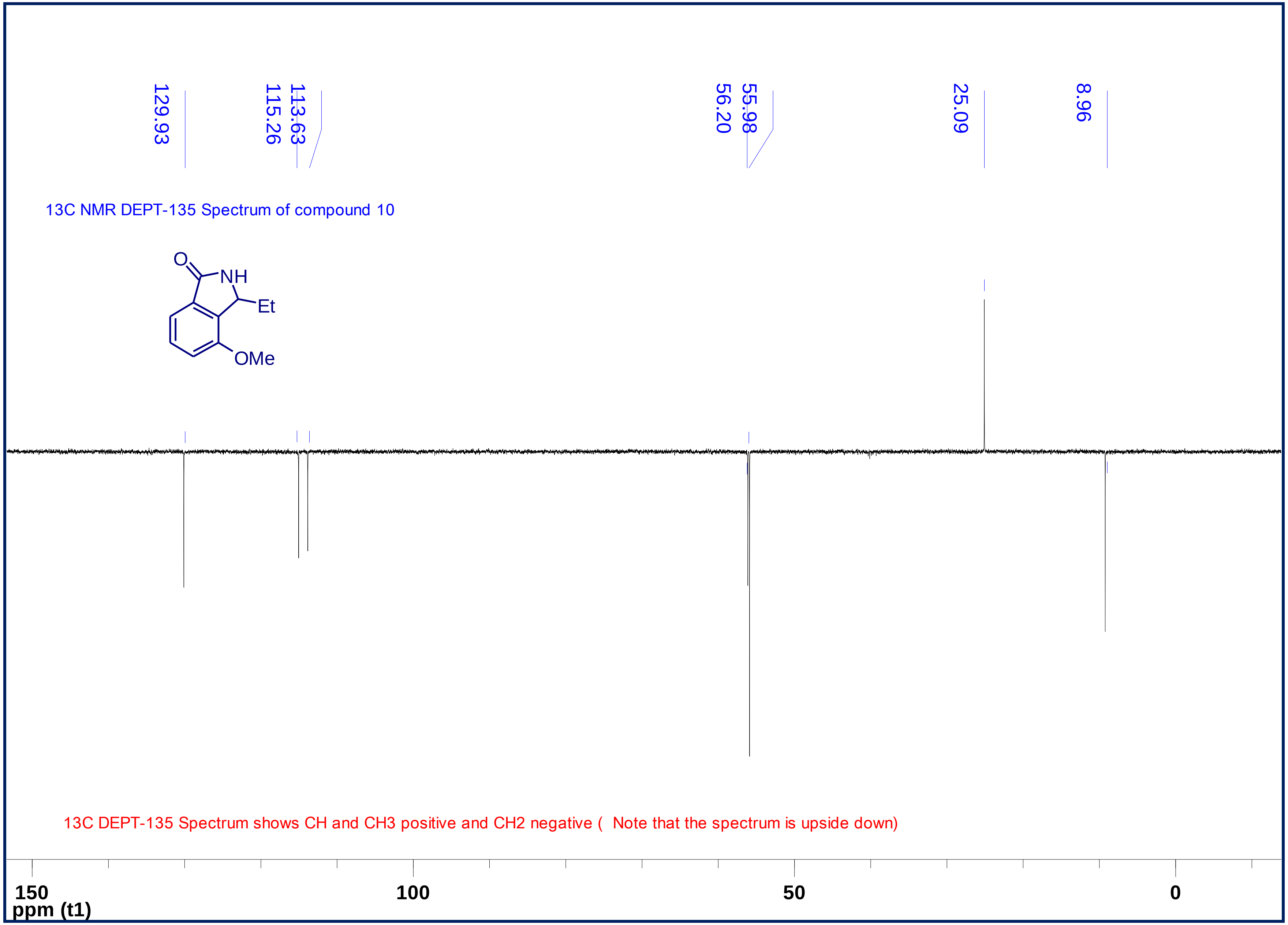

129.93
115.26
113.63
56.20
55.98
25.09
8.96
13C NMR DEPT-135 Spectrum of compound 10
O
NH
Et
OMe
13C DEPT-135 Spectrum shows CH and CH3 positive and CH2 negative (Note that the spectrum is upside down)
150
100
50
0
ppm (t1)

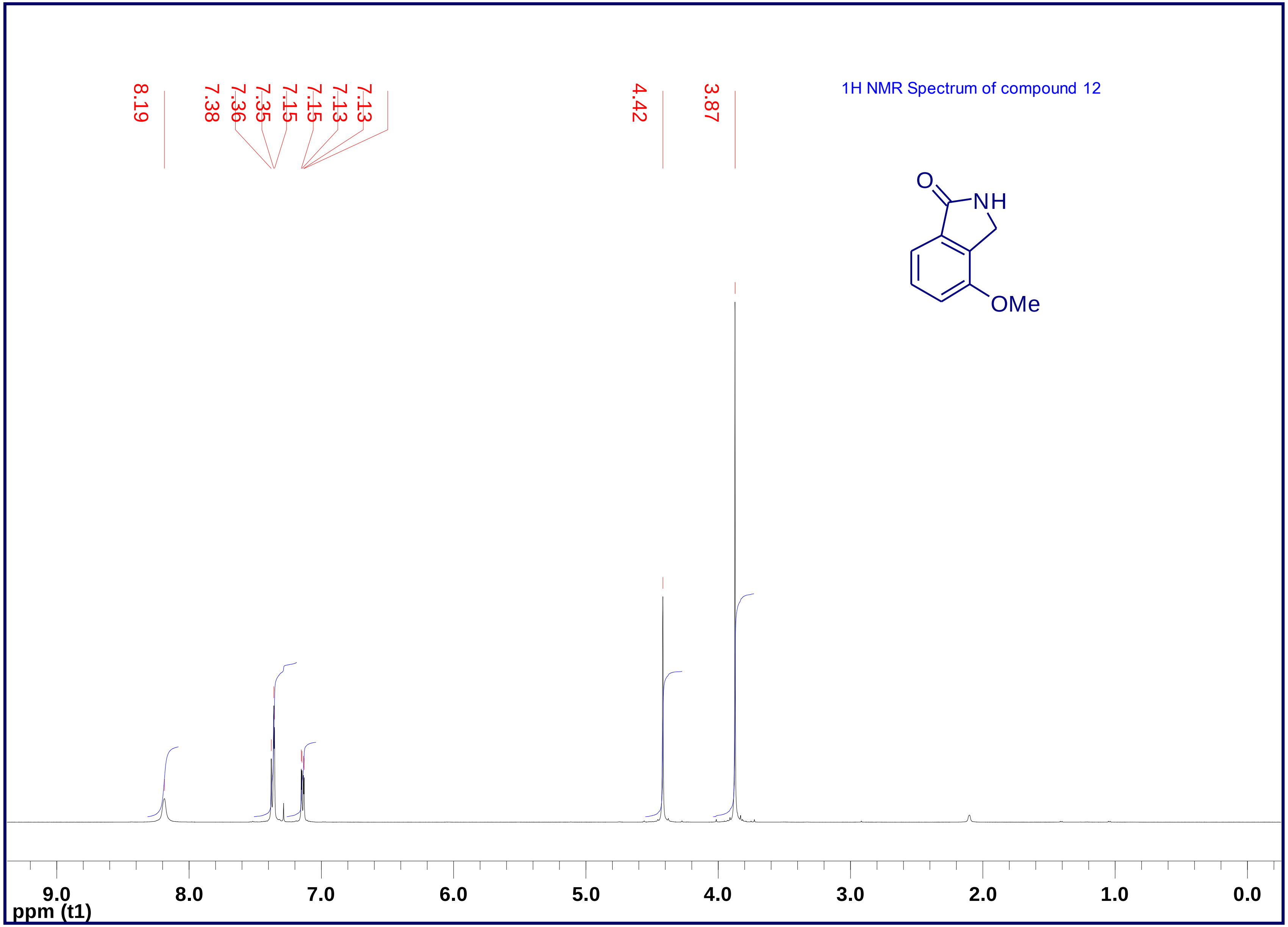
8.19
7.38
7.36
7.35
7.15
7.15
7.13
7.13
4.42
3.87
1H NMR Spectrum of compound 12
O
NH
OMe
9.0
8.0
7.0
6.0
5.0
4.0
3.0
2.0
1.0
0.0
ppm (t1)

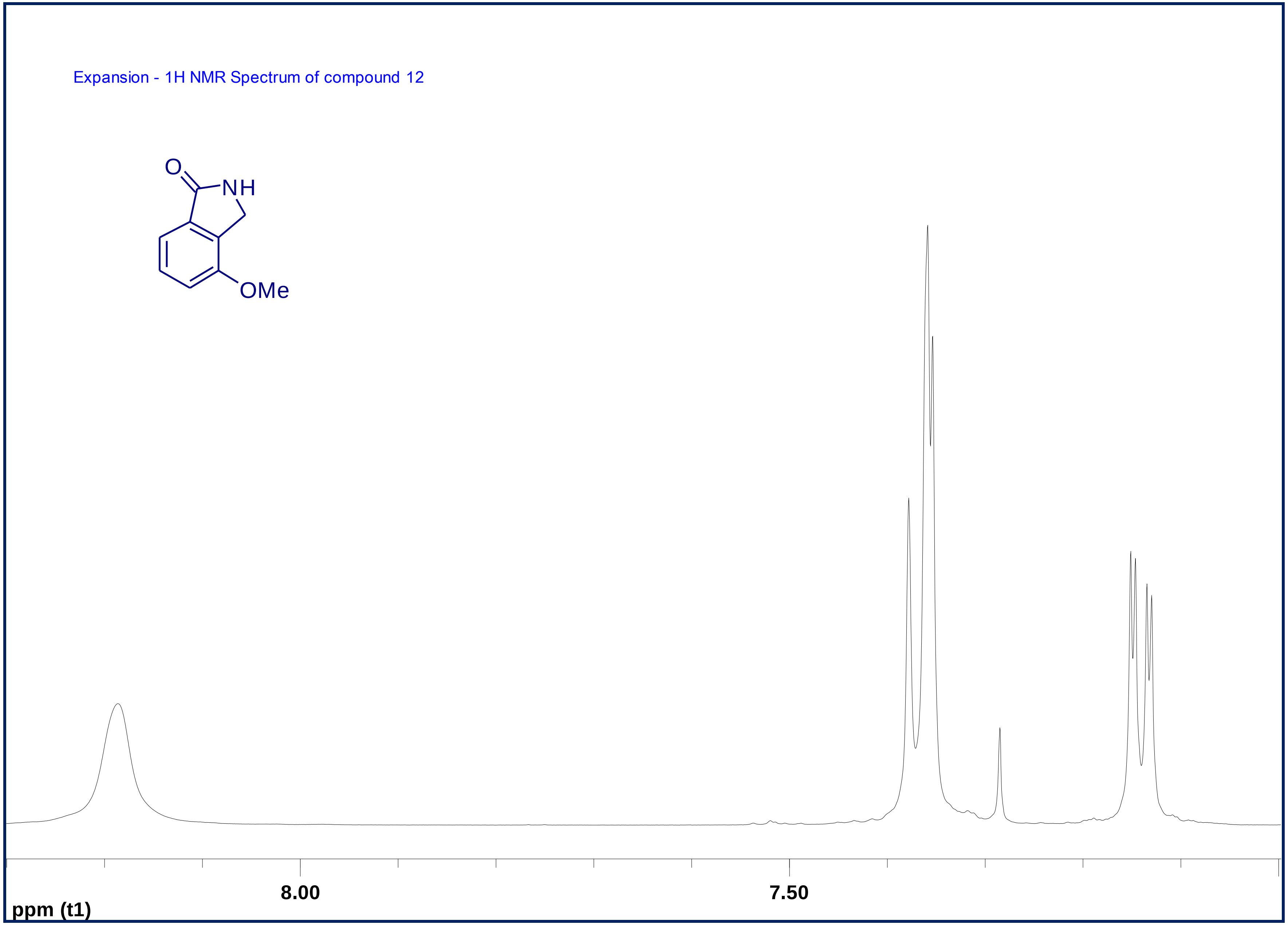
Expansion - 1H NMR Spectrum of compound 12
O
NH
OMe
8.00
7.50
ppm (t1)

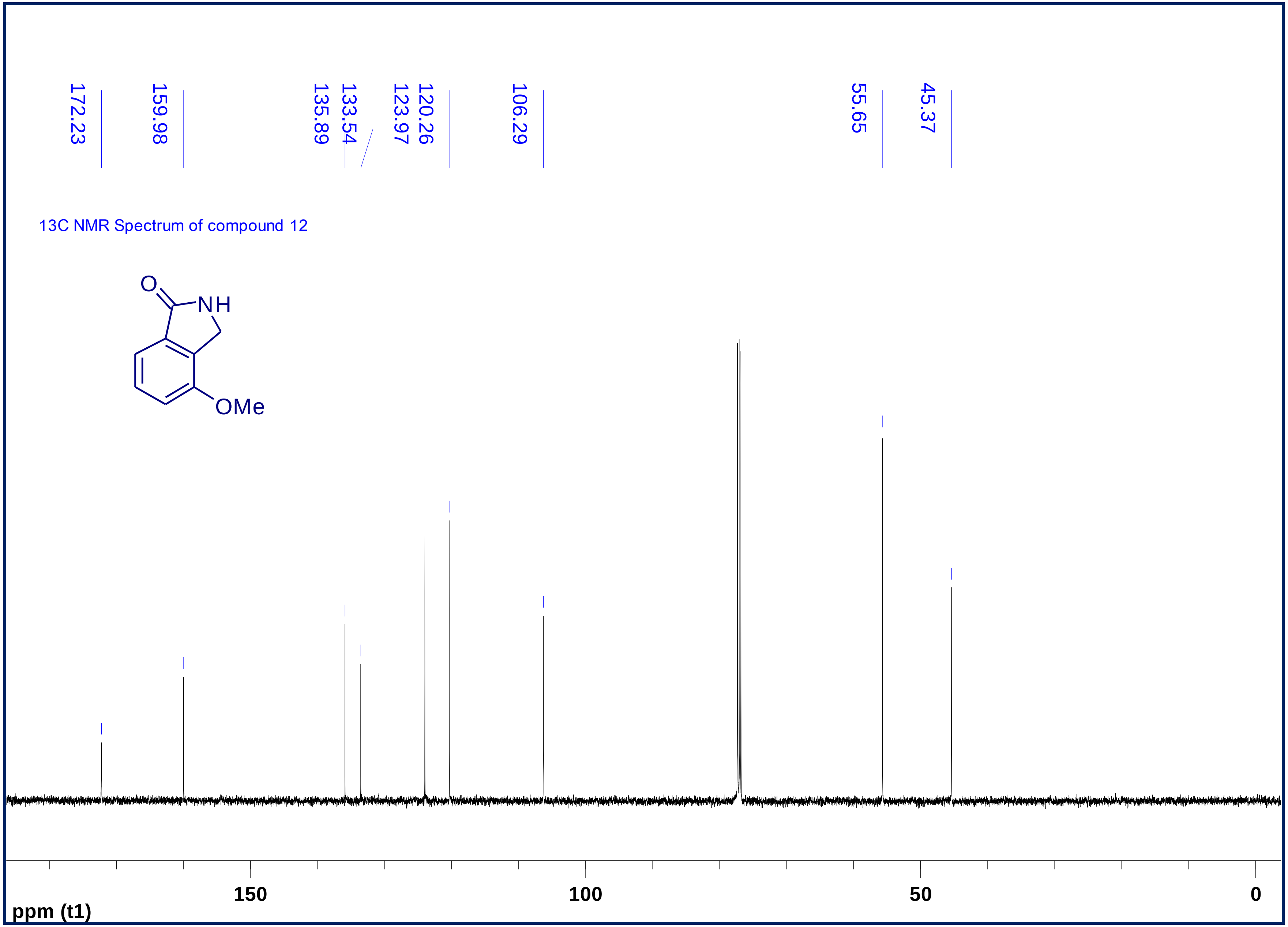

172.23
159.98
135.89
133.54
123.97
120.26
106.29
55.65
45.37
13C NMR Spectrum of compound 12
O
NH
OMe
150
100
50
0
ppm (t1)

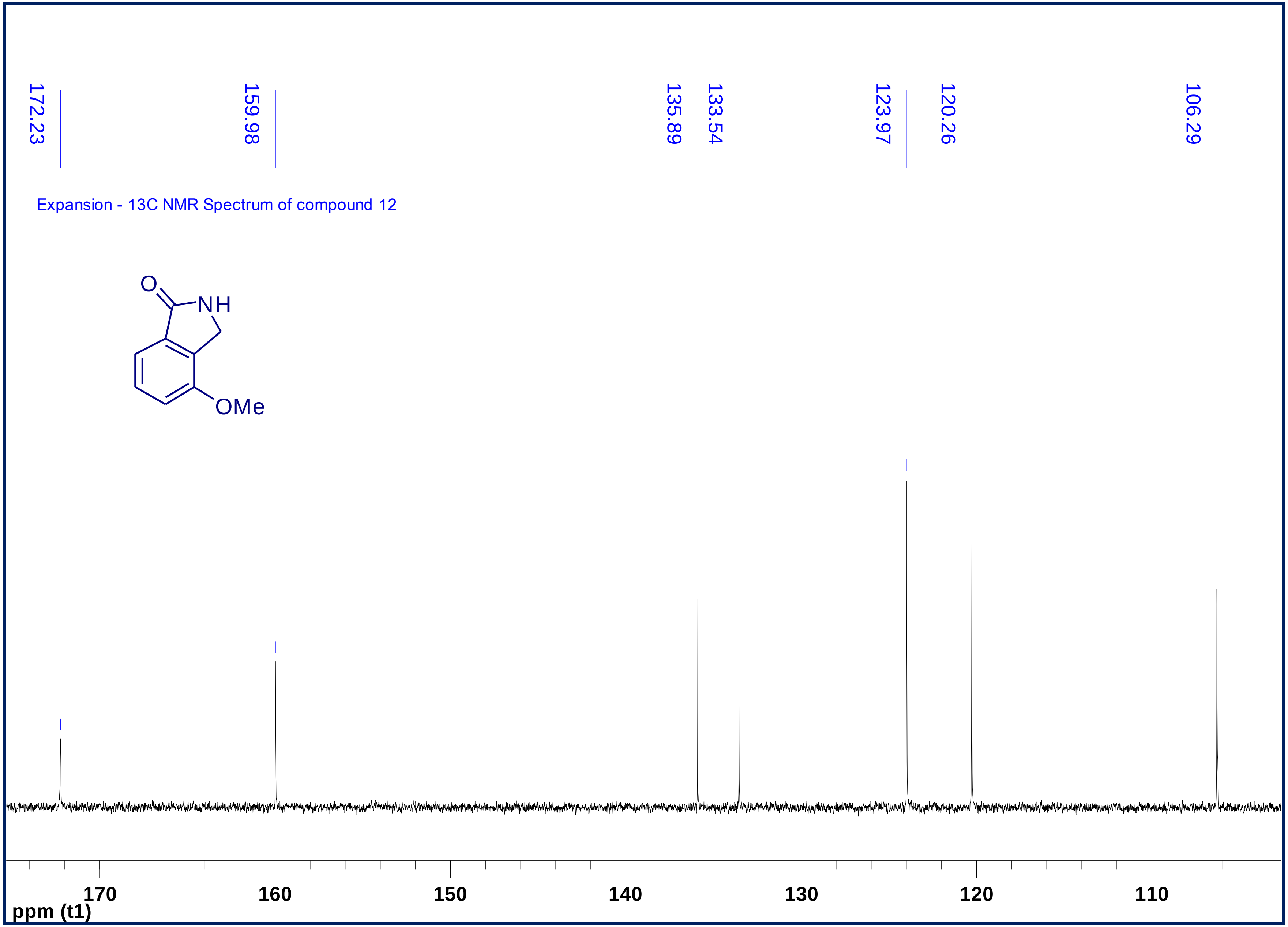

172.23
159.98
135.89
133.54
123.97
120.26
106.29
Expansion - 13C NMR Spectrum of compound 12
O
NH
OMe
170
160
150
140
130
120
110
ppm (t1)

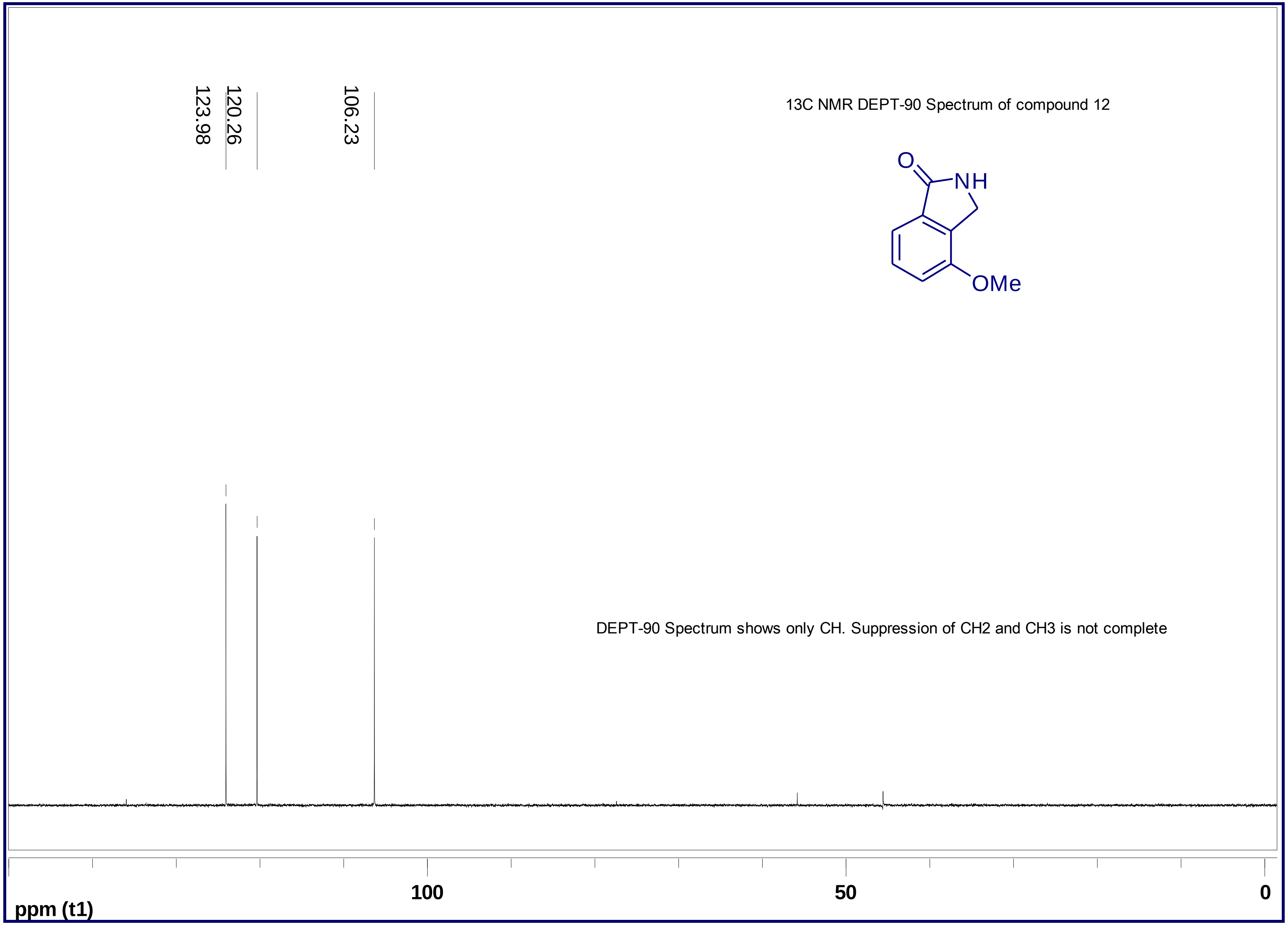

13C NMR DEPT-90 Spectrum of compound 12
O
NH
OMe
123.98
120.26
106.23
DEPT-90 Spectrum shows only CH. Suppression of CH2 and CH3 is not complete
100
50
0
ppm (t1)

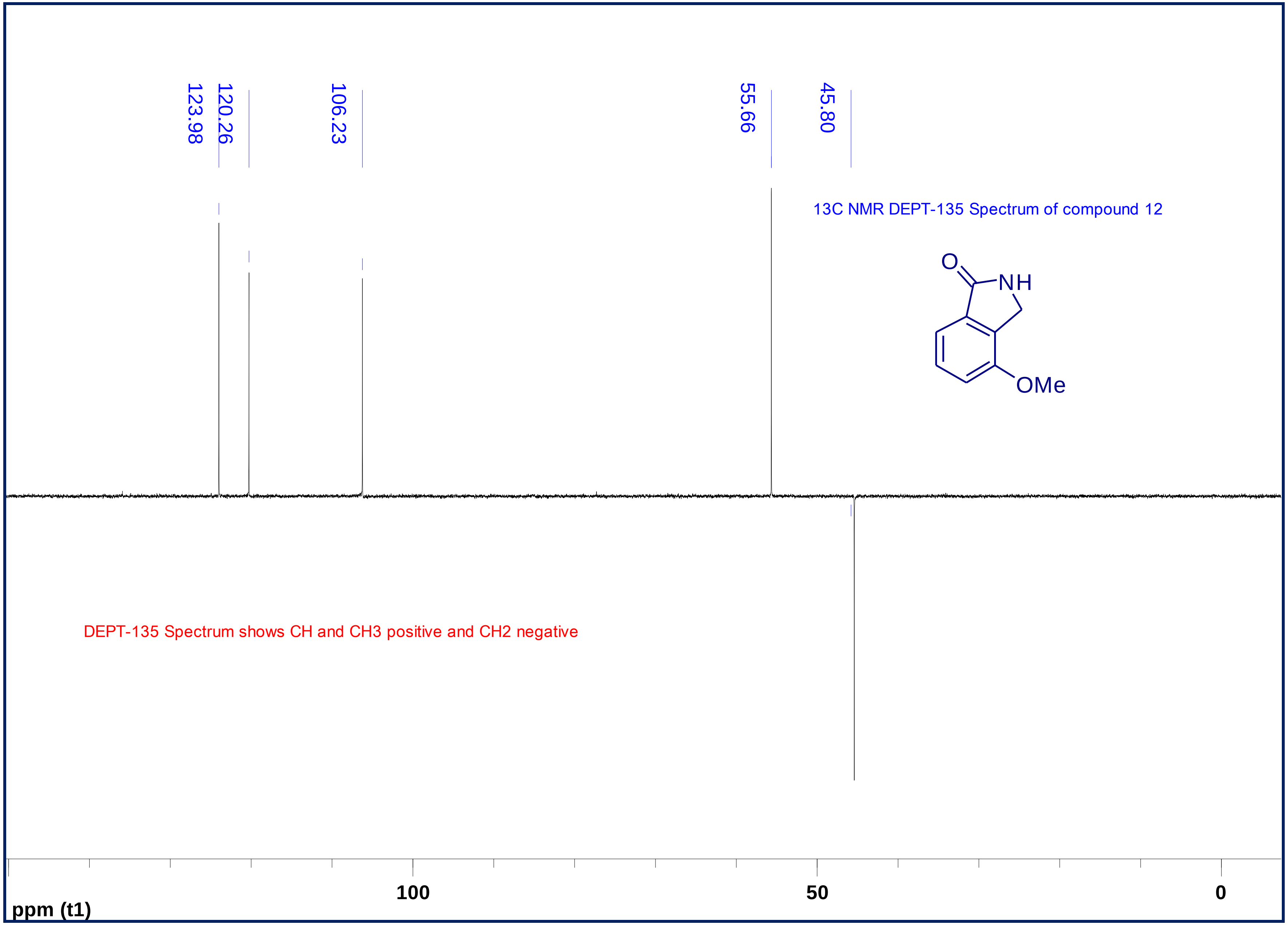
123.98
120.26
106.23
55.66
45.80
13C NMR DEPT-135 Spectrum of compound 12
O
NH
OMe
DEPT-135 Spectrum shows CH and CH3 positive and CH2 negative
100
50
0
ppm (t1)

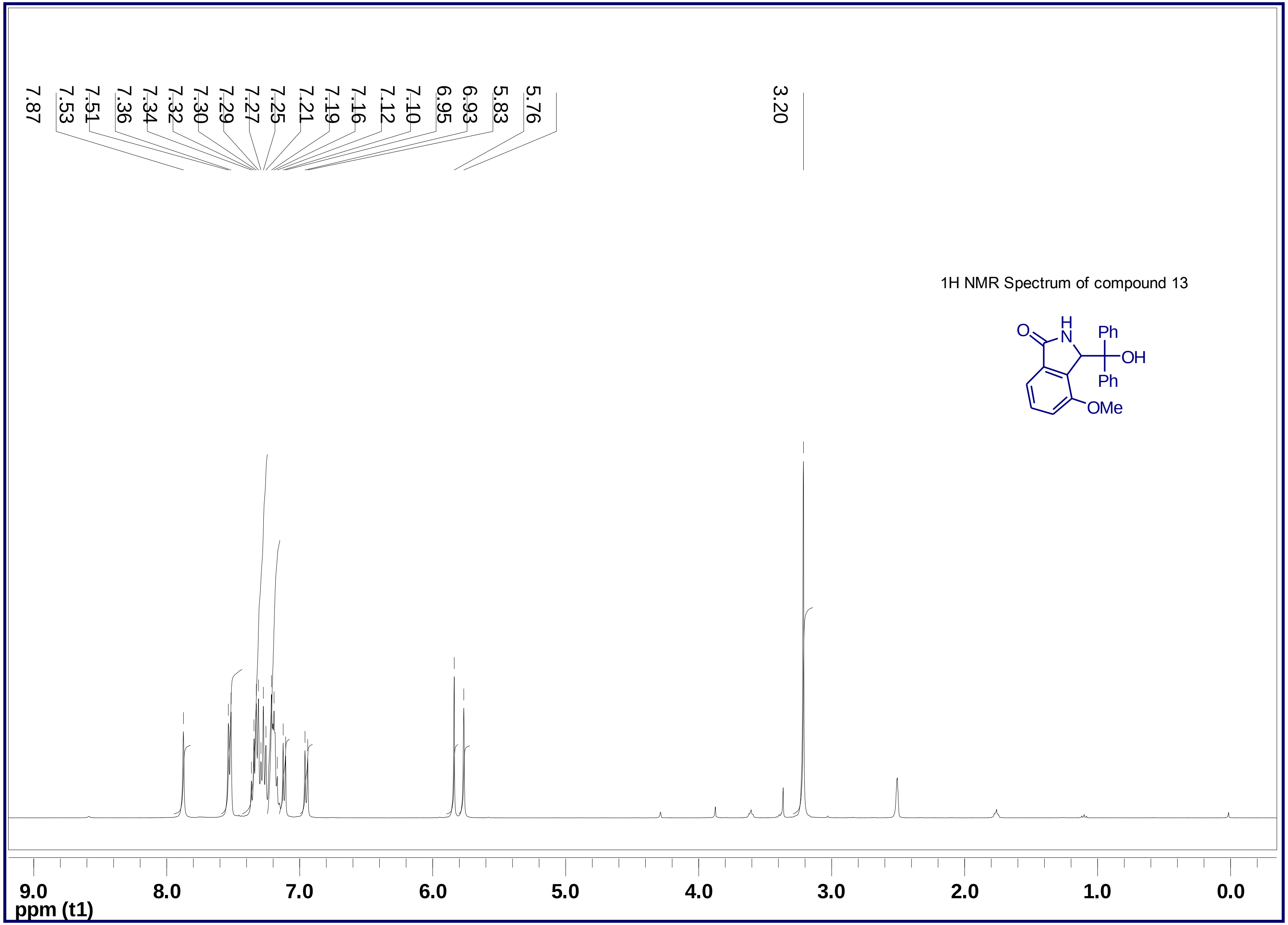

7.87
7.53
7.51
7.36
7.34
7.32
7.30
7.29
7.27
7.25
7.21
7.19
7.16
7.12
7.10
6.95
6.93
5.83
5.76
3.20
1H NMR Spectrum of compound 13
O
H
N
Ph
OH
Ph
OMe
9.0
8.0
7.0
6.0
5.0
4.0
3.0
2.0
1.0
0.0
ppm (t1)

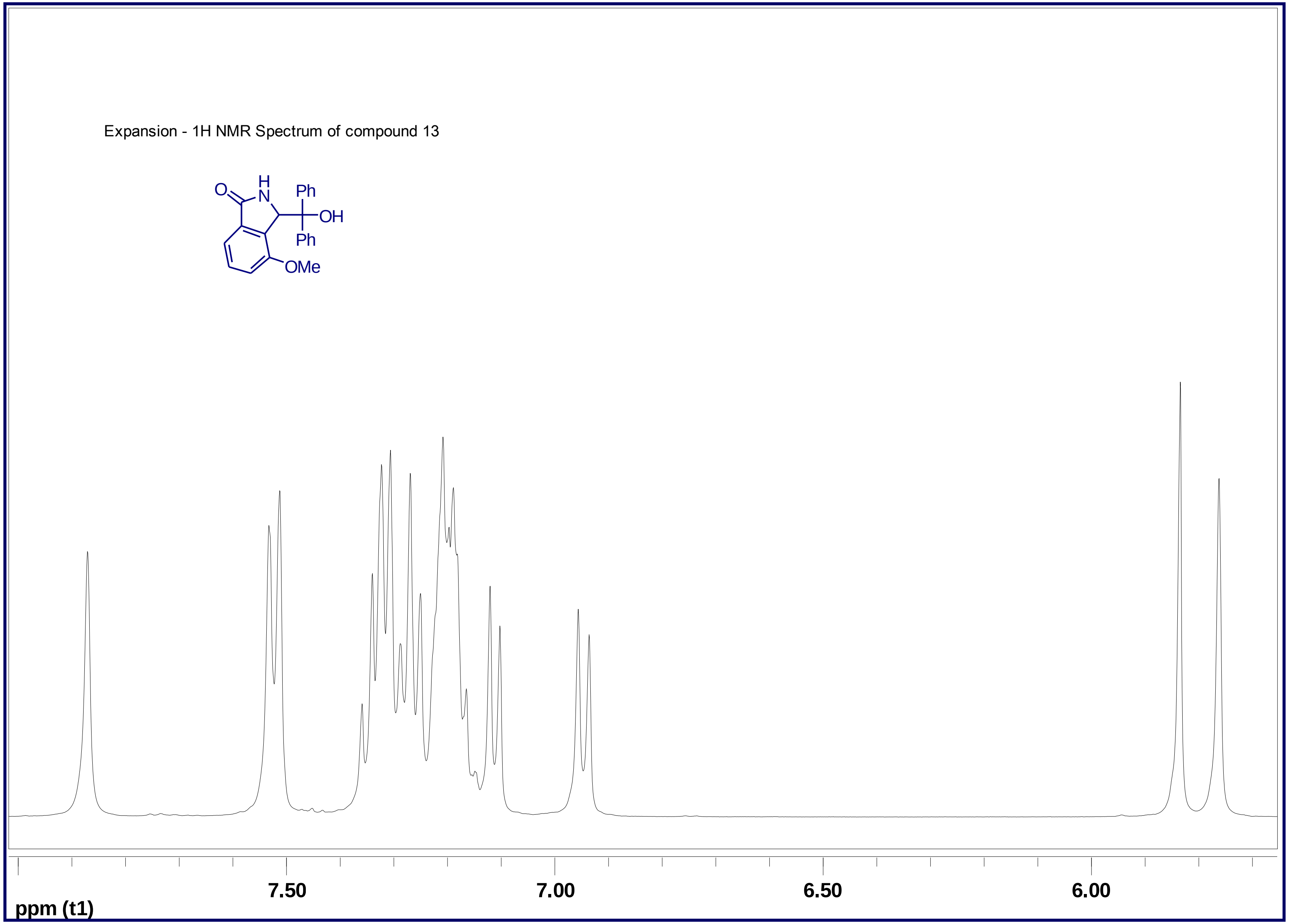

Expansion - 1H NMR Spectrum of compound 13
O
H
N
Ph
OH
Ph
OMe
7.50
7.00
6.50
6.00
ppm (t1)

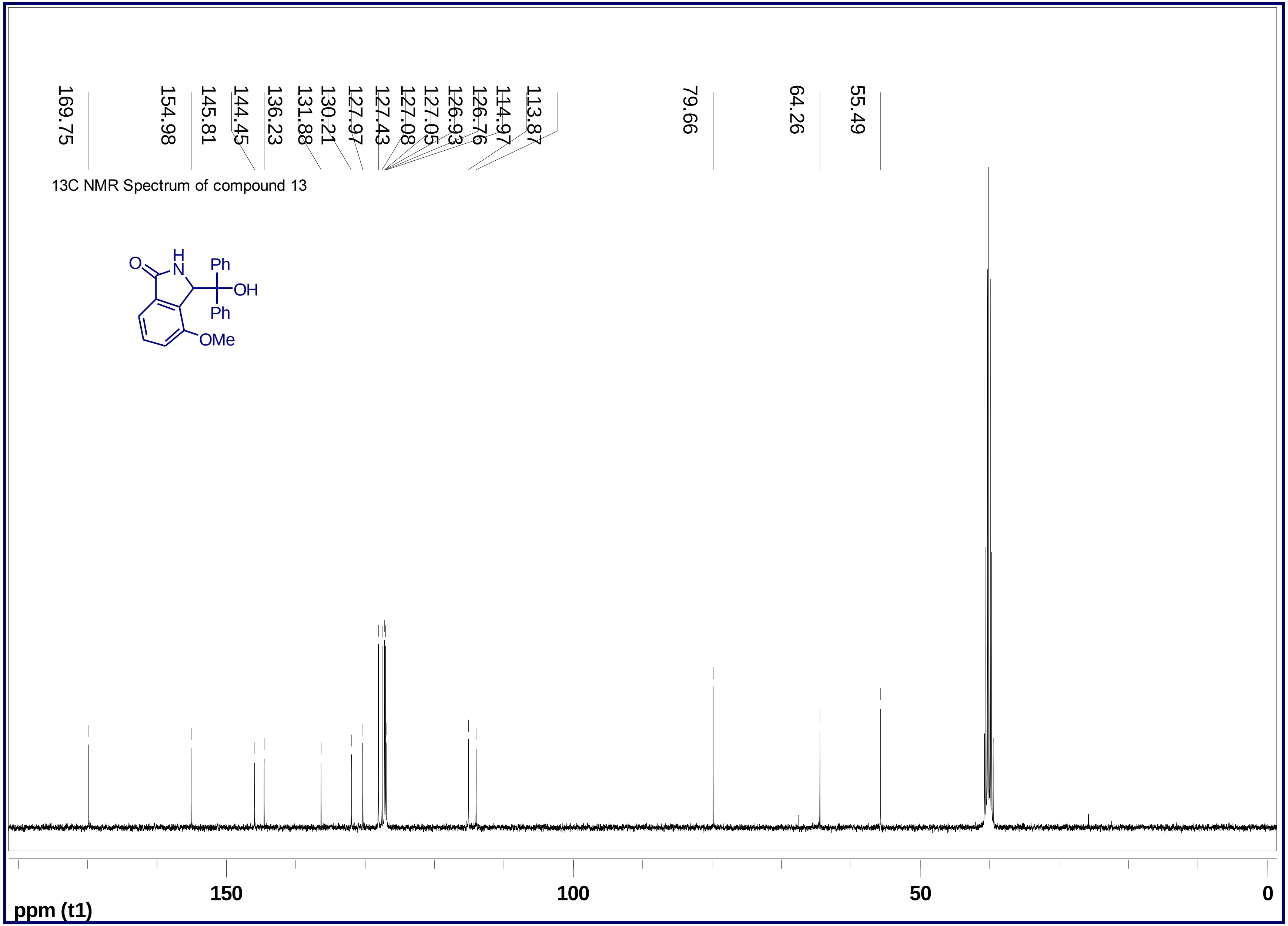

169.75
154.98
145.81
144.45
136.23
131.88
130.21
127.97
127.43
127.08
127.05
126.93
126.76
114.97
113.87
79.66
64.26
55.49
13C NMR Spectrum of compound 13
Ph
OH
Ph
OMe
150
100
50
0
ppm (t1)

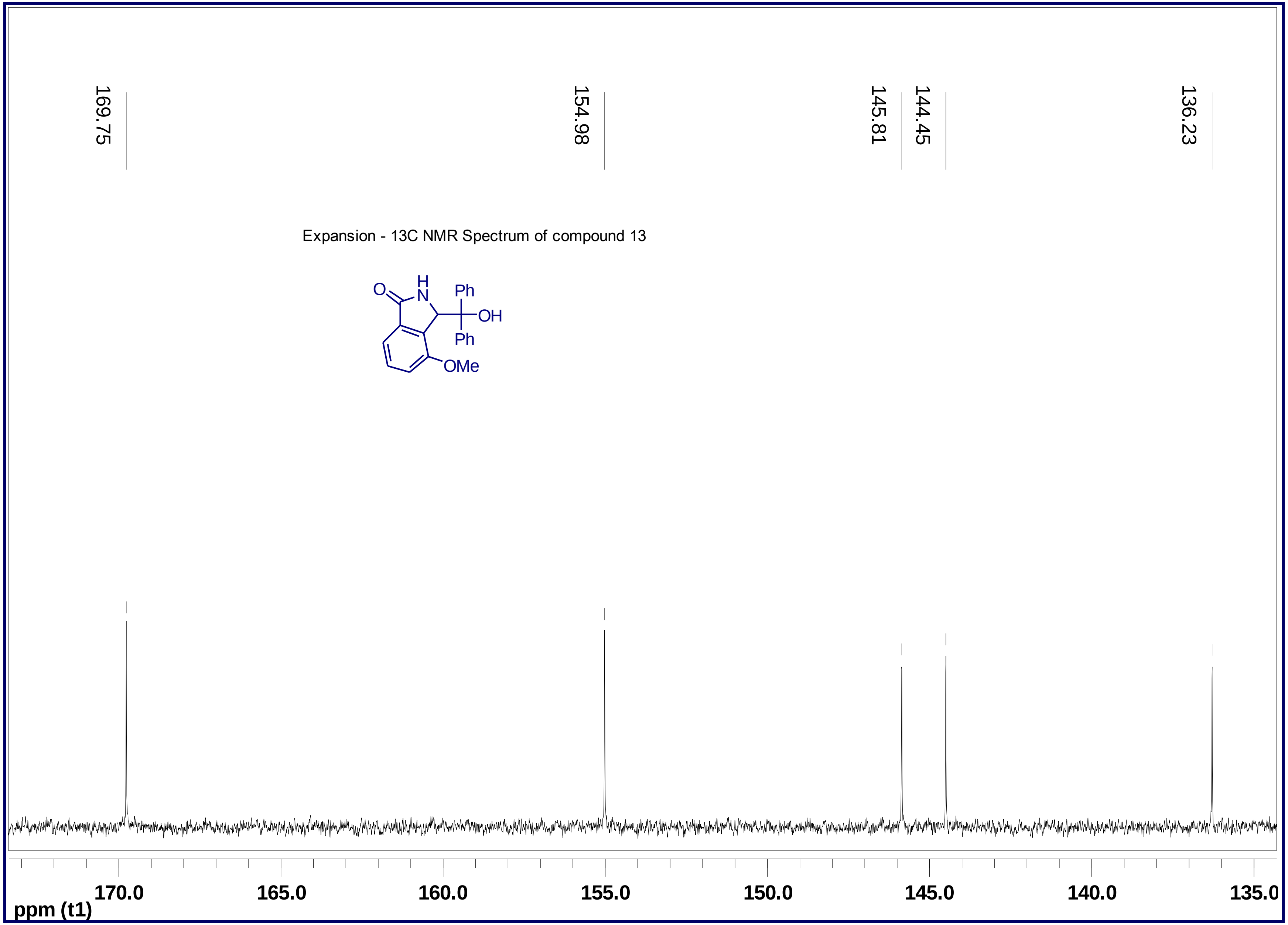
169.75
154.98
145.81
144.45
136.23
Expansion - 13C NMR Spectrum of compound 13
O
H
N
Ph
OH
Ph
OMe
170.0
165.0
160.0
155.0
150.0
145.0
140.0
135.0
ppm (t1)

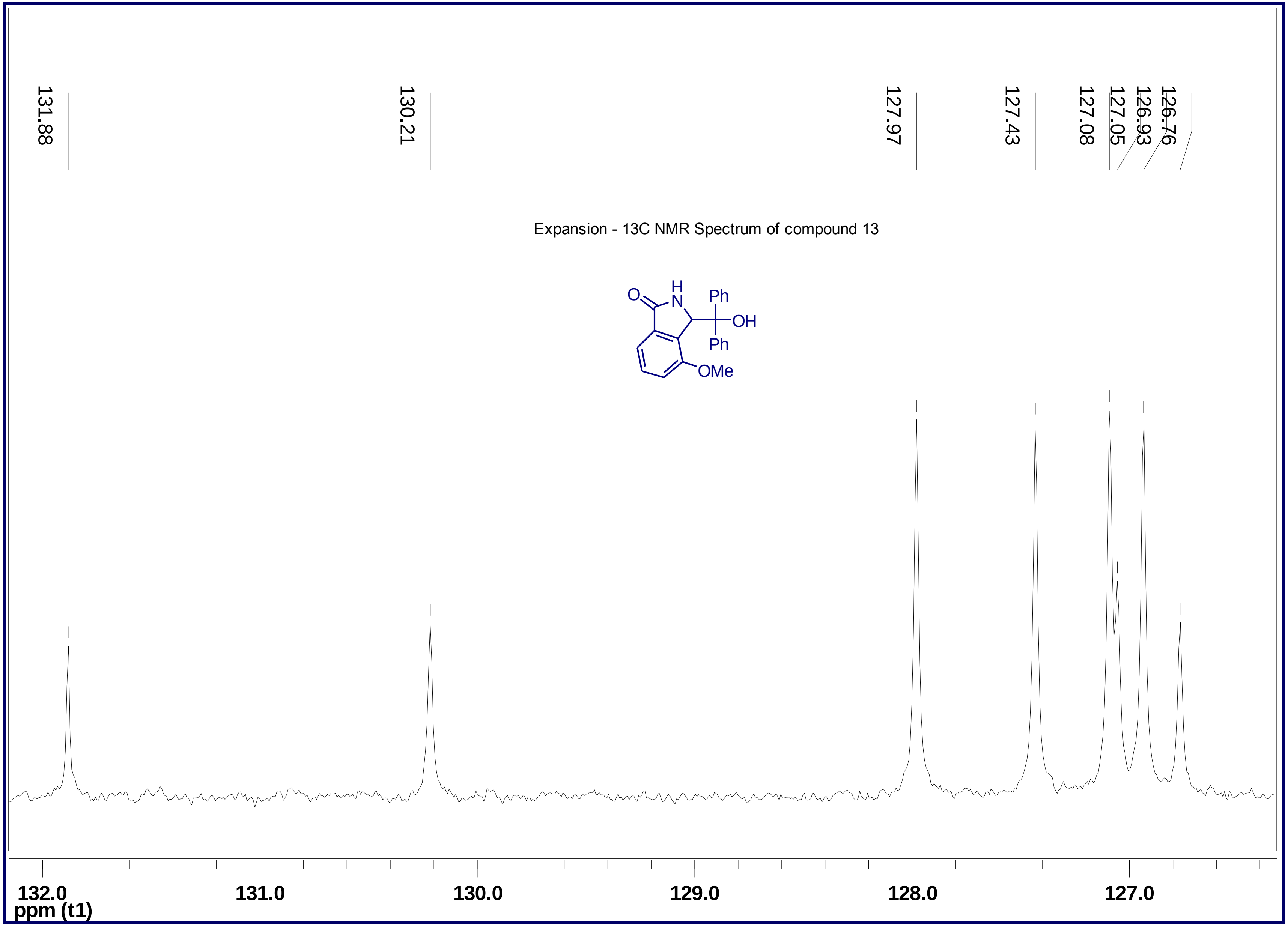
131.88
130.21
127.97
127.43
127.08
127.05
126.93
126.76
Expansion - 13C NMR Spectrum of compound 13
O
H
N
Ph
OH
Ph
OMe
132.0
131.0
130.0
129.0
128.0
127.0
ppm (t1)

Expansion - 13C NMR Spectrum of compound 13

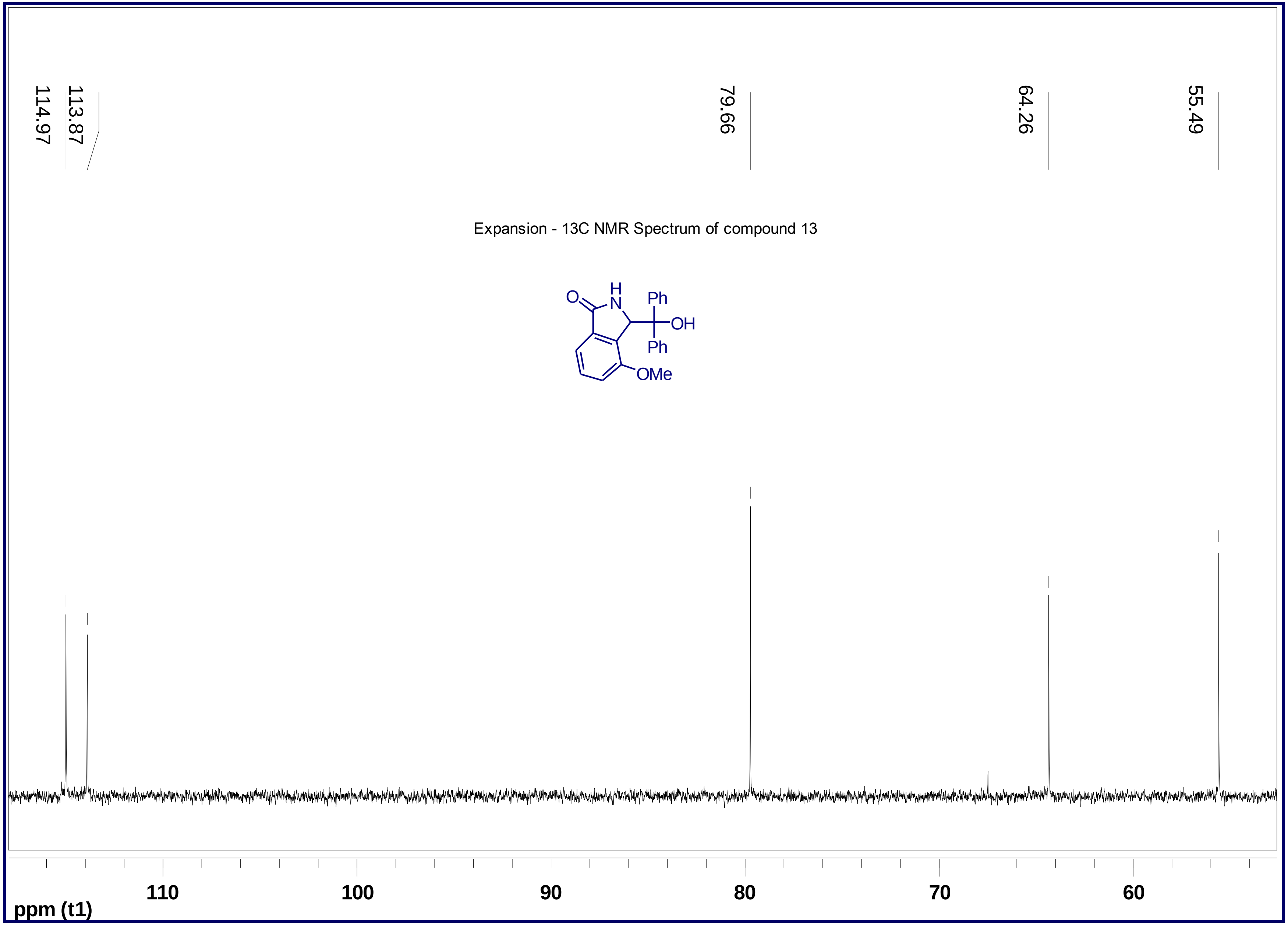

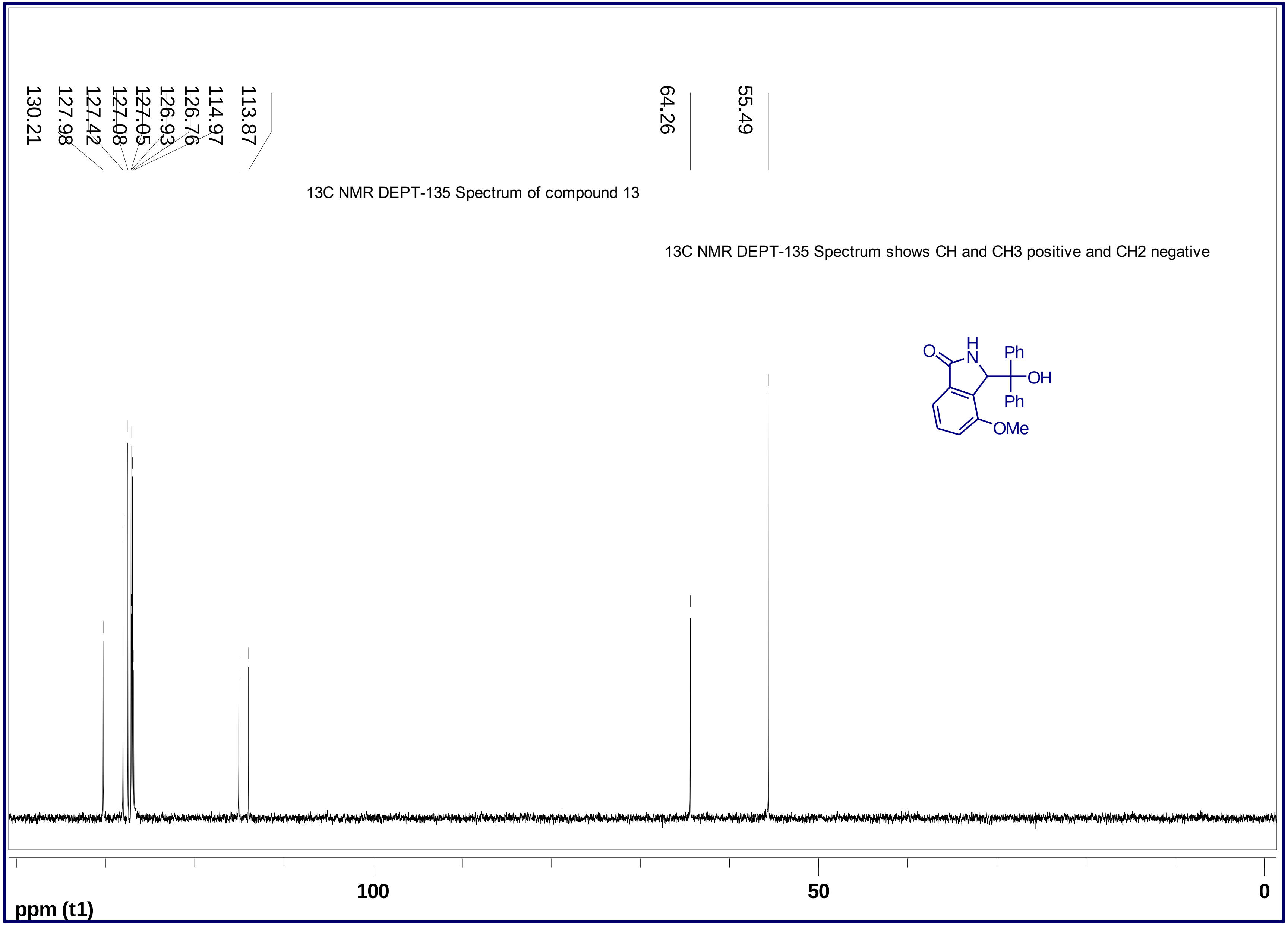
130.21
127.98
127.42
127.08
127.05
126.93
126.76
114.97
113.87
64.26
55.49
13C NMR DEPT-135 Spectrum of compound 13
13C NMR DEPT-135 Spectrum shows CH and CH3 positive and CH2 negative
O
H
N
Ph
OH
Ph
OMe
100
50
0
ppm (t1)

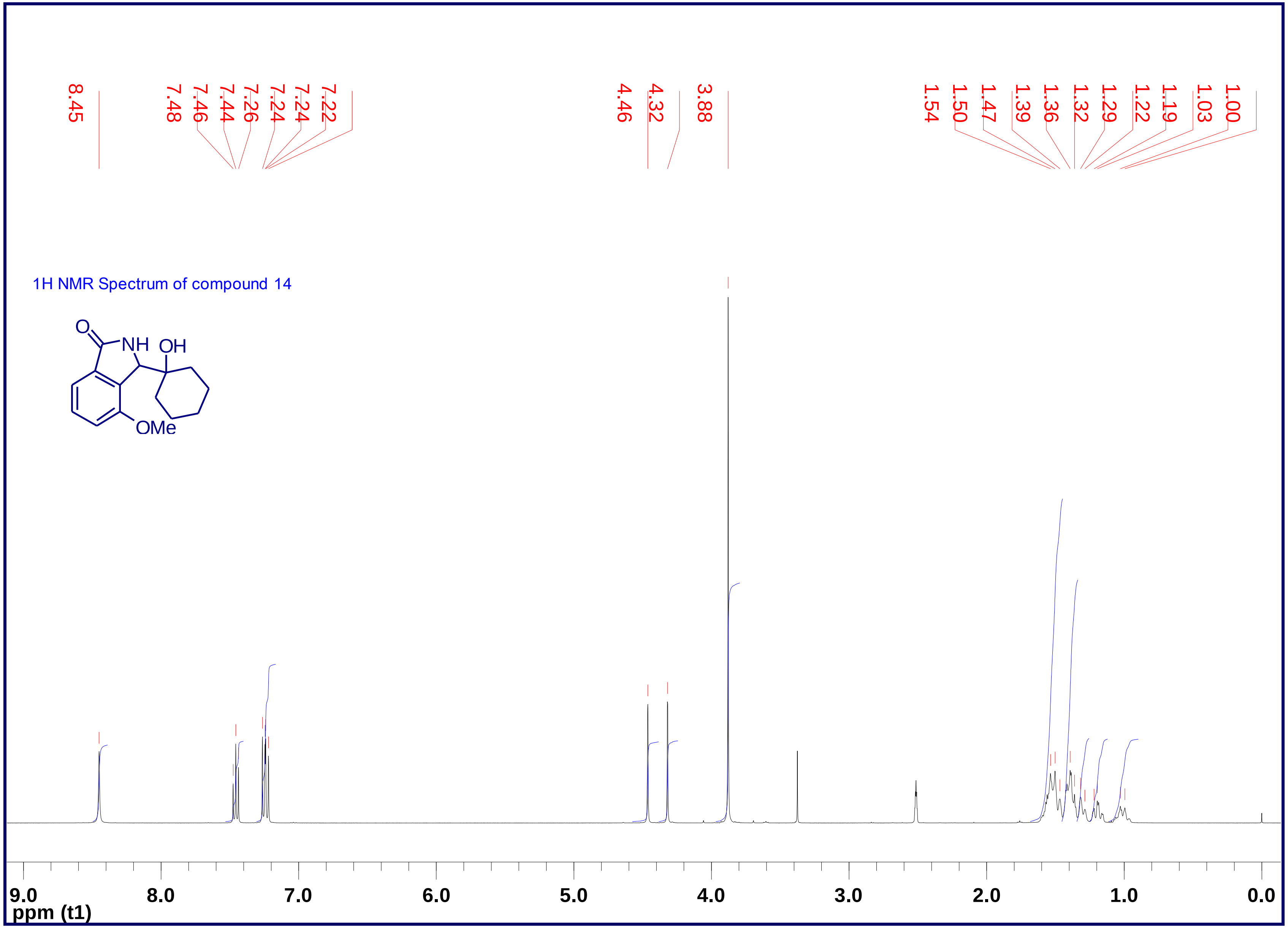

1H NMR Spectrum of compound 14
NH
OH
O
OMe
8.45
7.48
7.46
7.44
7.26
7.24
7.24
7.22
4.46
4.32
3.88
1.54
1.50
1.47
1.39
1.36
1.32
1.29
1.22
1.19
1.03
1.00
9.0
8.0
7.0
6.0
5.0
4.0
3.0
2.0
1.0
0.0
ppm (t1)

Expansion - 1H NMR Spectrum of compound 14

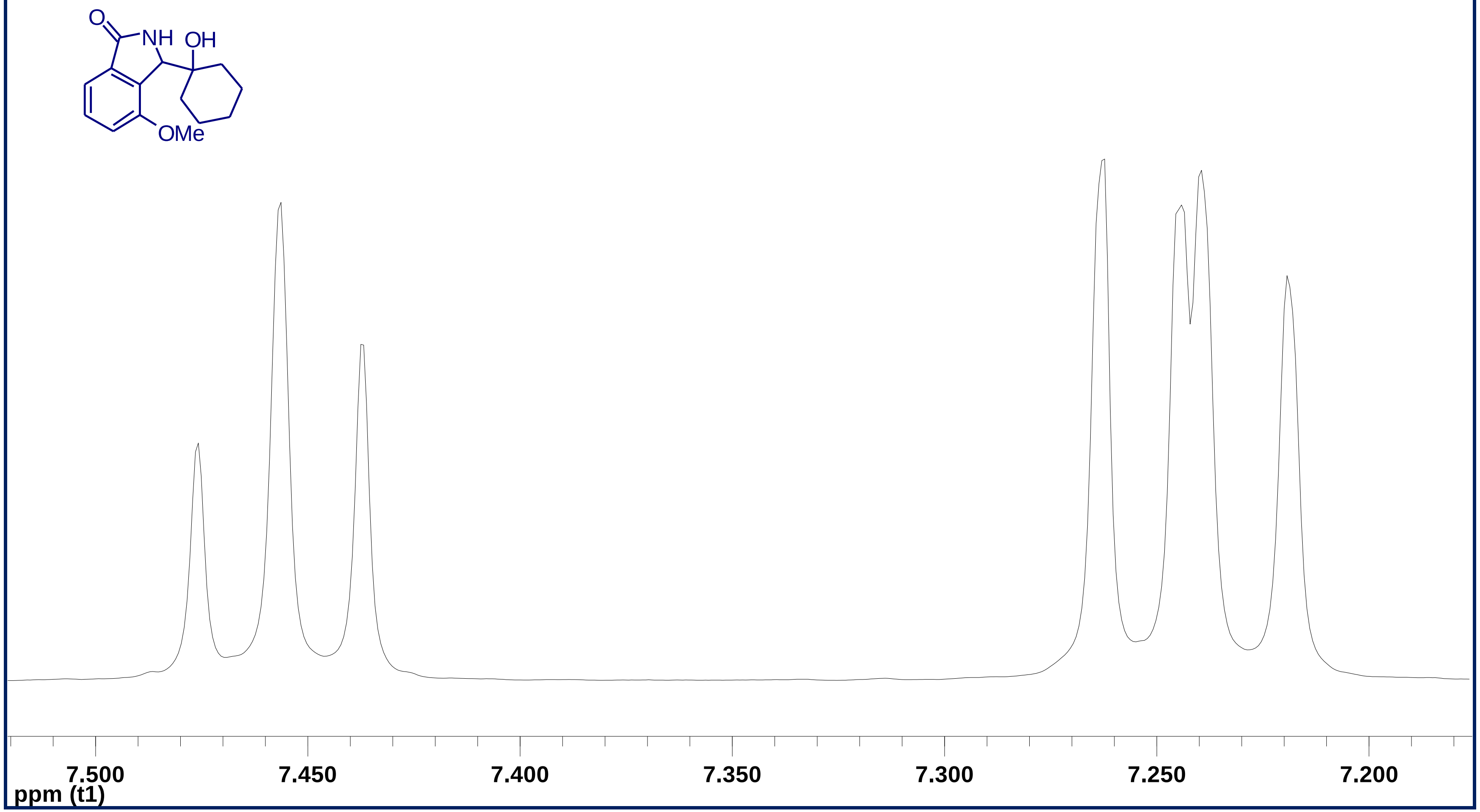

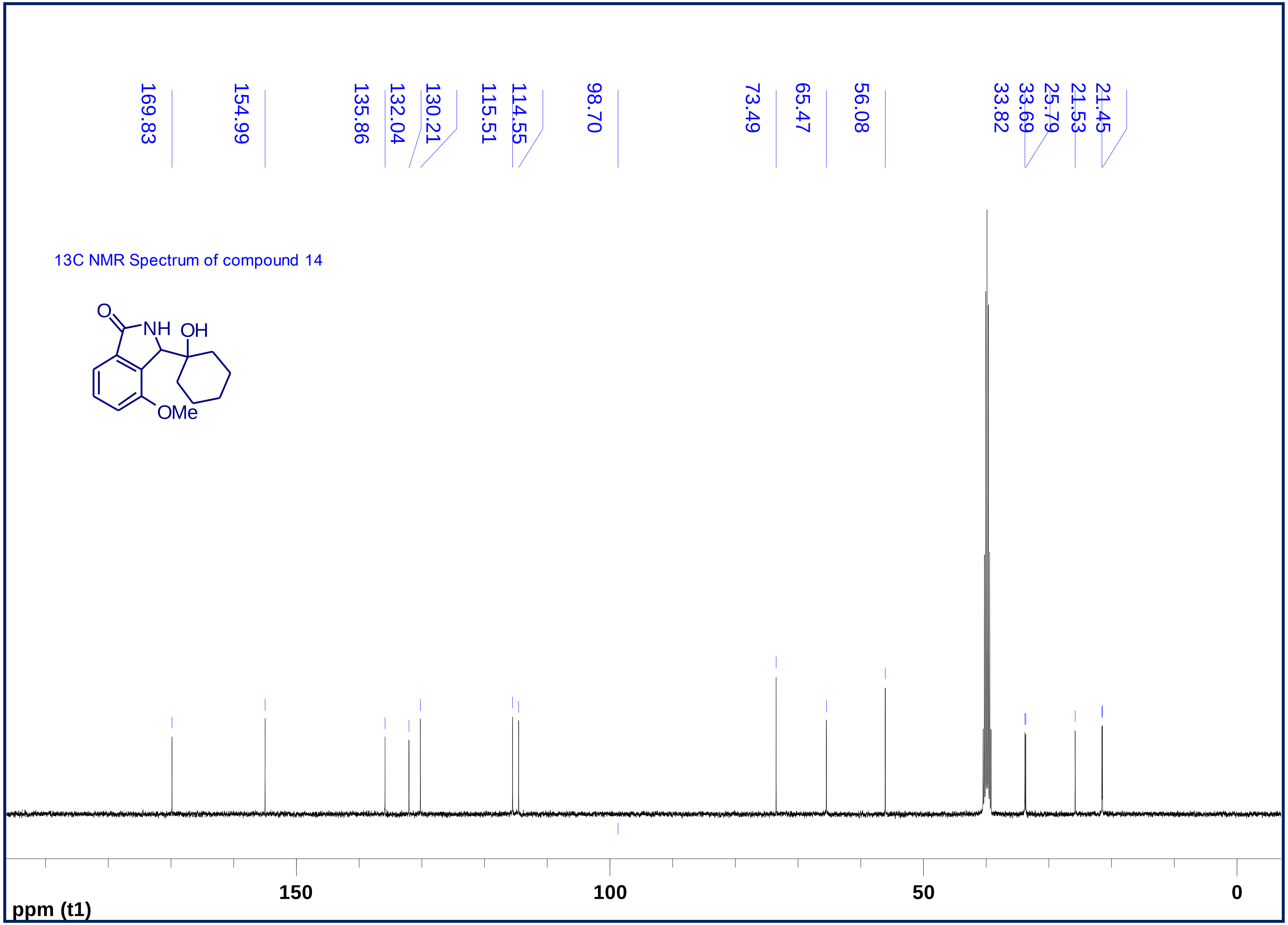
169.83
154.99
135.86
132.04
130.21
115.51
114.55
98.70
73.49
65.47
56.08
33.82
33.69
25.79
21.53
21.45
13C NMR Spectrum of compound 14
O
NH
OH
OMe
150
100
50
0
ppm (t1)

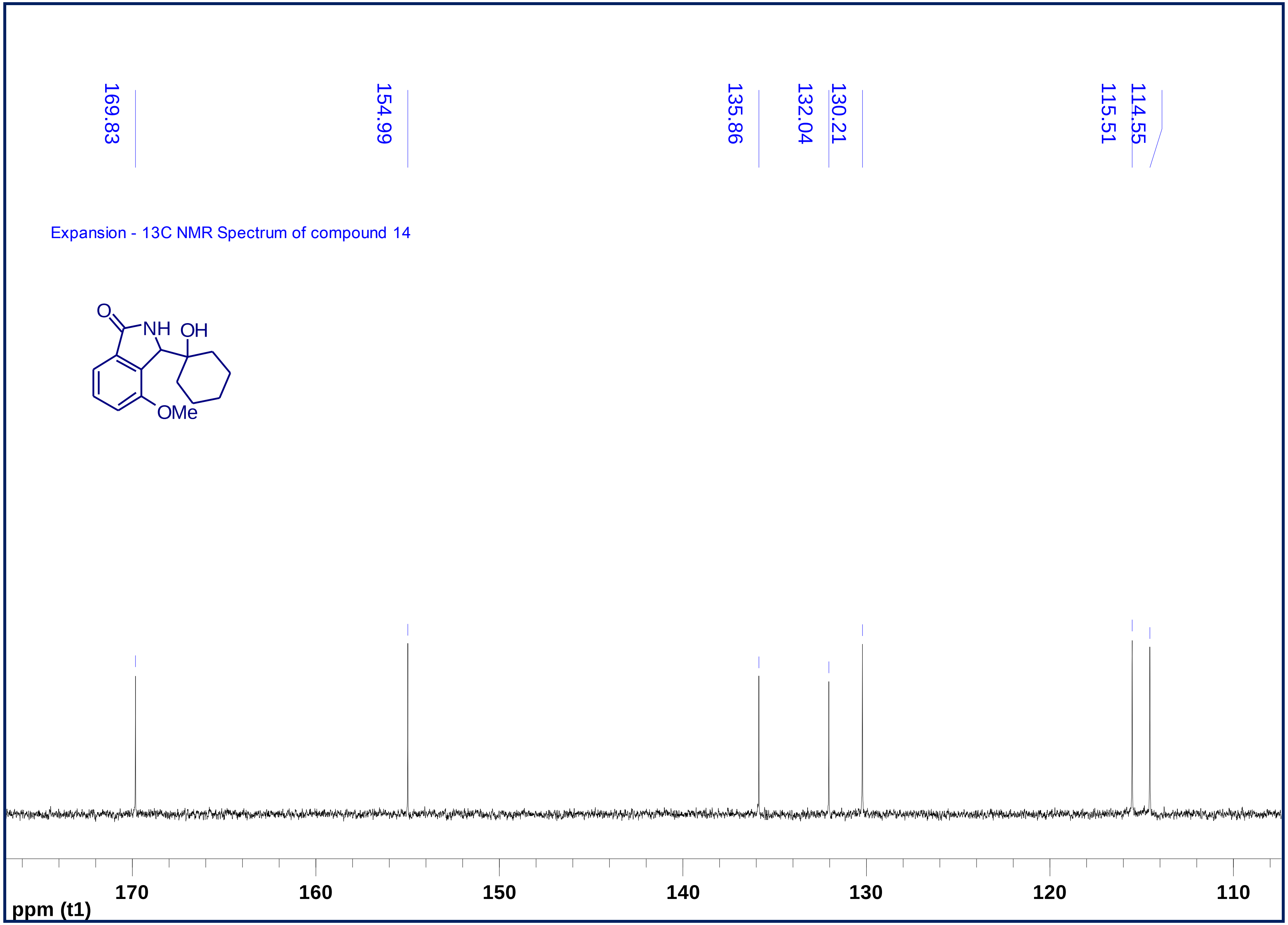

169.83
154.99
135.86
132.04
130.21
115.51
114.55
Expansion - 13C NMR Spectrum of compound 14
O
NH
OH
OMe
170
160
150
140
130
120
110
ppm (t1)

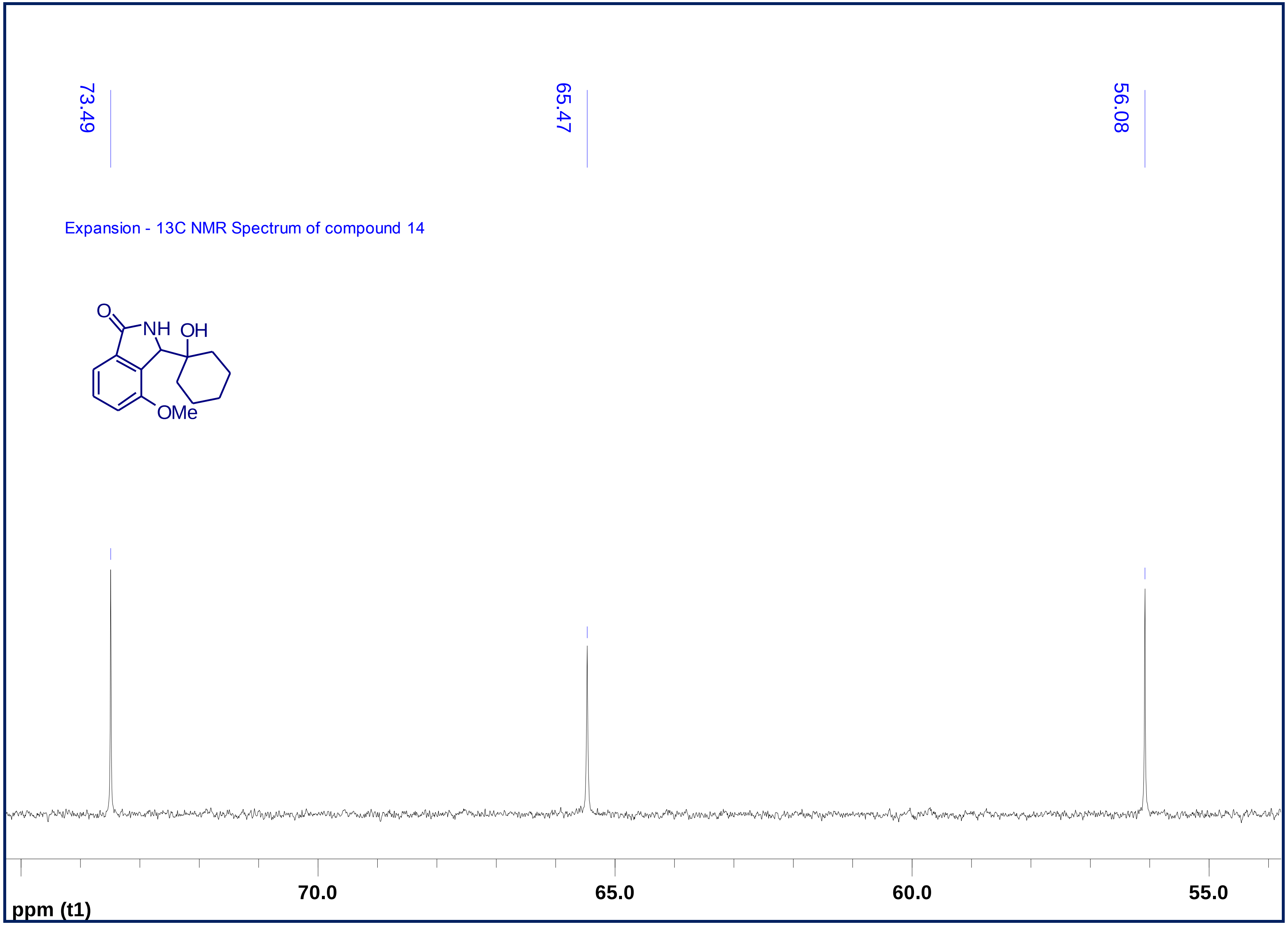
73.49
65.47
56.08
Expansion - 13C NMR Spectrum of compound 14
O
NH
OH
OMe
70.0
65.0
60.0
55.0
ppm (t1)

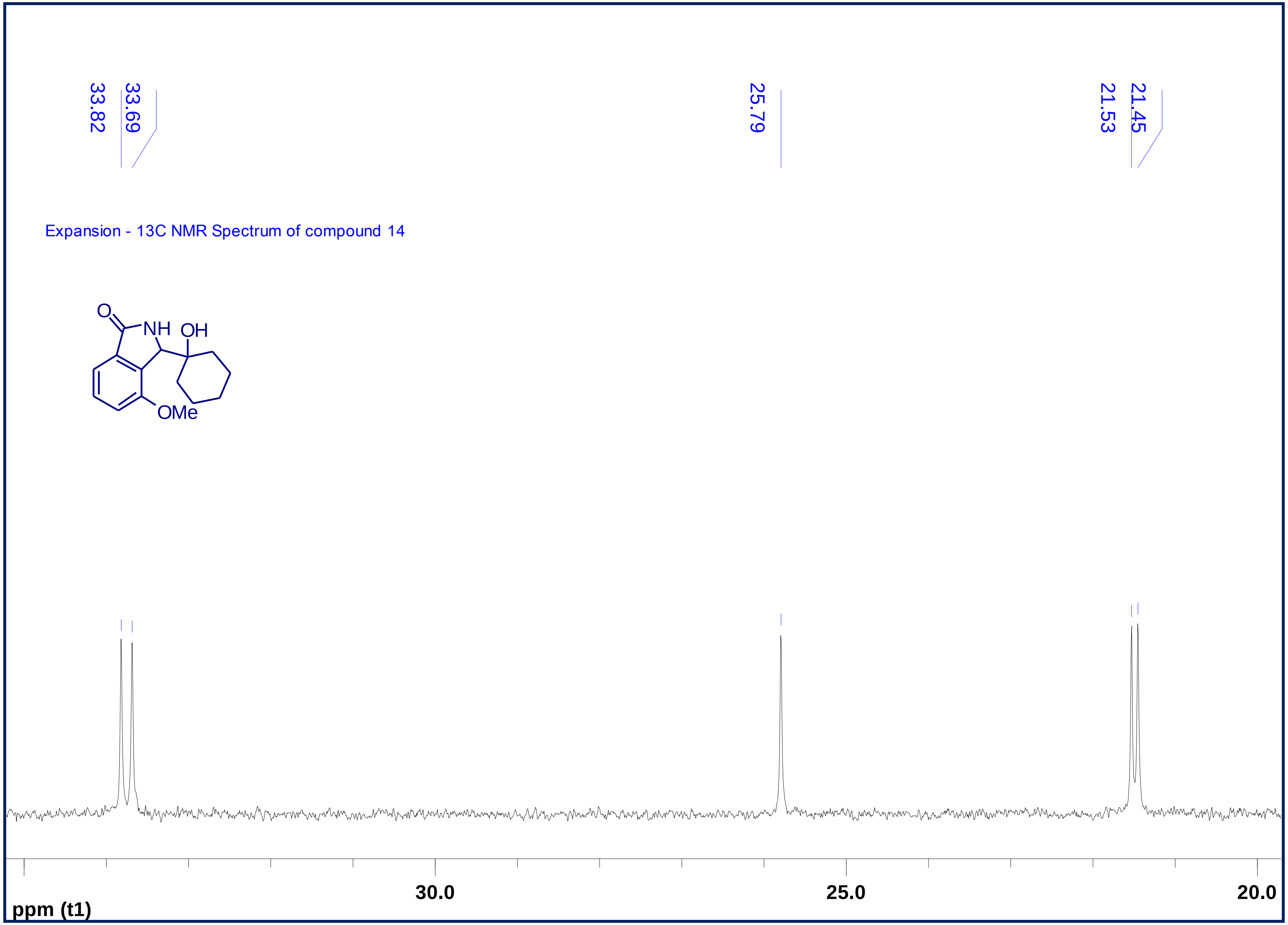
33.82
33.69
25.79
21.53
21.45
Expansion - 13C NMR Spectrum of compound 14
O
NH
OH
OMe
30.0
25.0
20.0
ppm (t1)

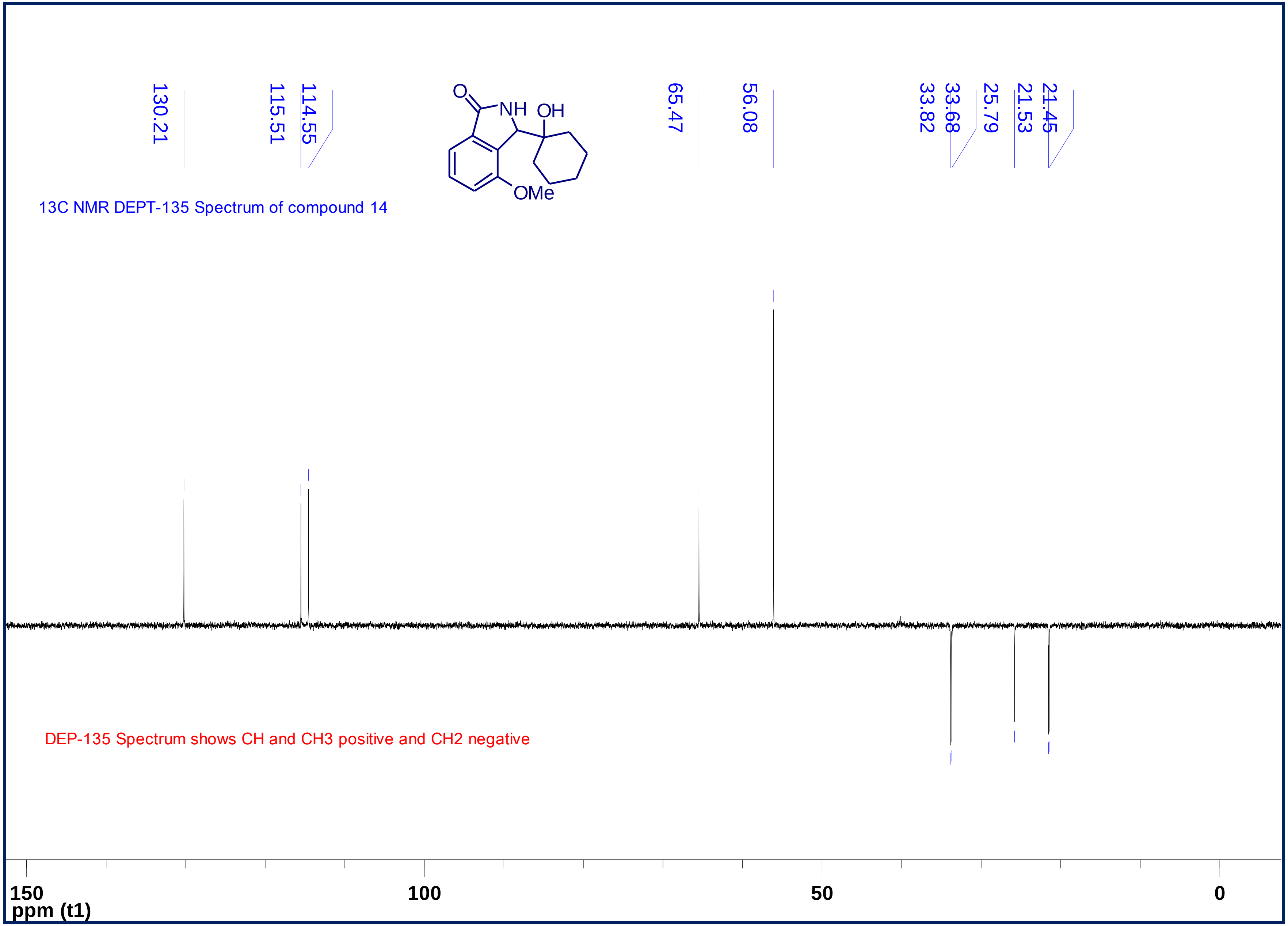
130.21
115.51
114.55
O
NH
OH
OMe
65.47
56.08
33.82
33.68
25.79
21.53
21.45
13C NMR DEPT-135 Spectrum of compound 14
DEP-135 Spectrum shows CH and CH3 positive and CH2 negative
150
100
50
0
ppm (t1)

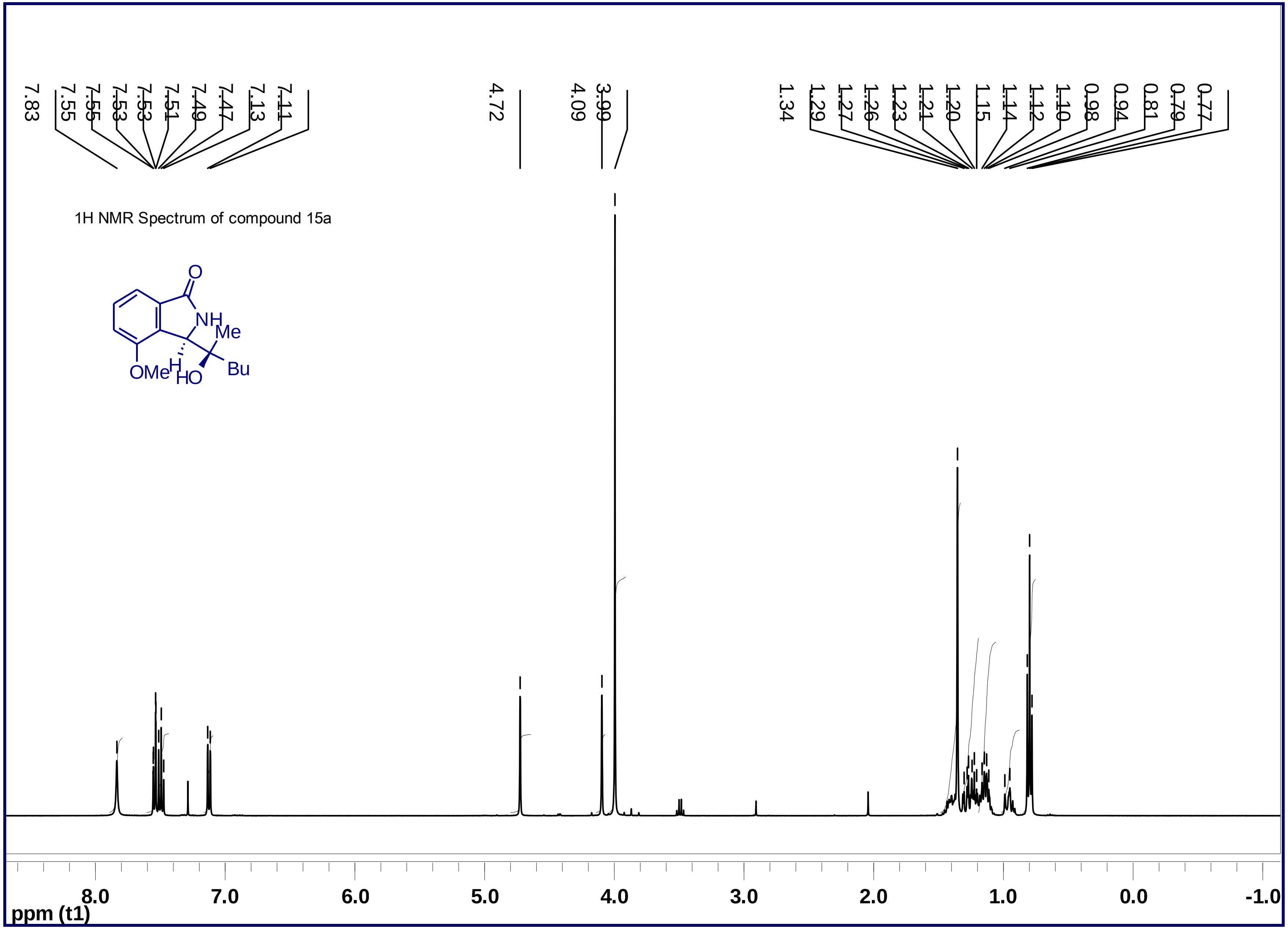
7.83
7.55
7.55
7.53
7.53
7.51
7.49
7.47
7.13
7.11
4.72
4.09
3.99
1.34
1.29
1.27
1.26
1.23
1.21
1.20
1.15
1.14
1.12
1.10
0.98
0.94
0.81
0.79
0.77
1H NMR Spectrum of compound 15a
O
NH
Me
OMe
H
HO
Bu
8.0
7.0
6.0
5.0
4.0
3.0
2.0
1.0
0.0
-1.0
ppm (t1)

Expansion - 1H NMR Spectrum of compound 15a

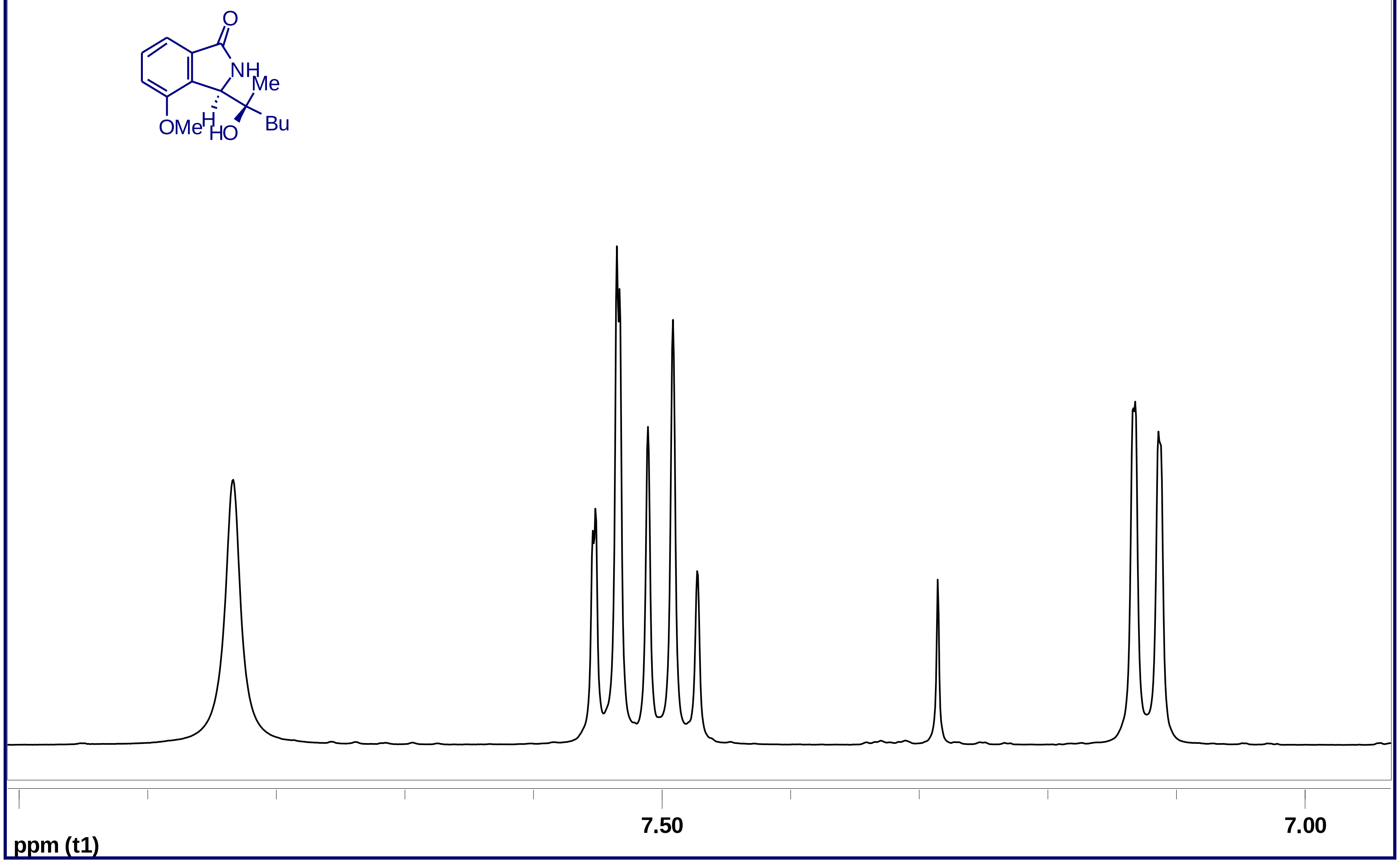

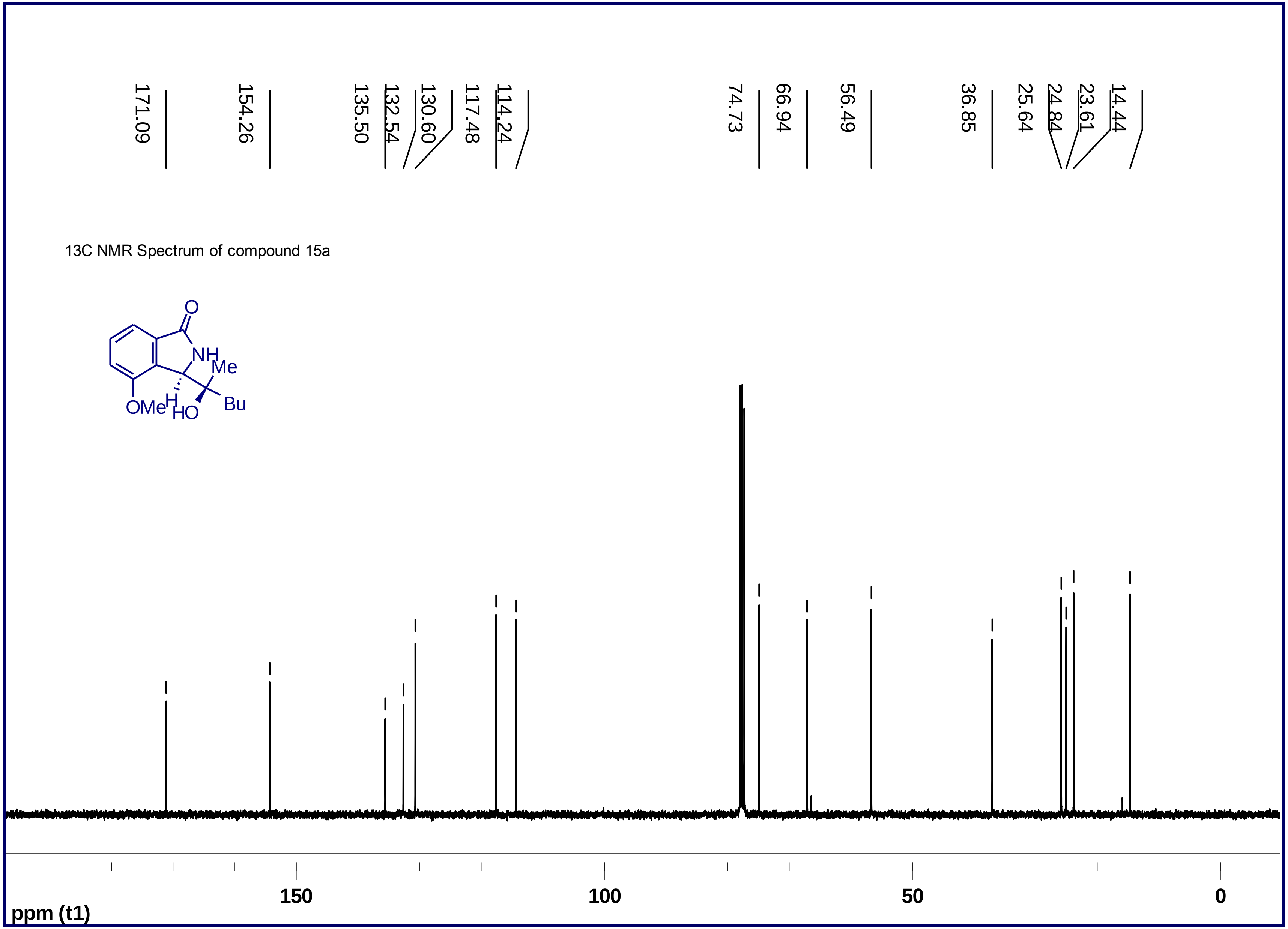
171.09
154.26
135.50
132.54
130.60
117.48
114.24
74.73
66.94
56.49
36.85
25.64
24.84
23.61
14.44
13C NMR Spectrum of compound 15a
O
NH
Me
OMe
H
HO
Bu
150
100
50
0
ppm (t1)

Expansion - 13C NMR Spectrum of compound 15a

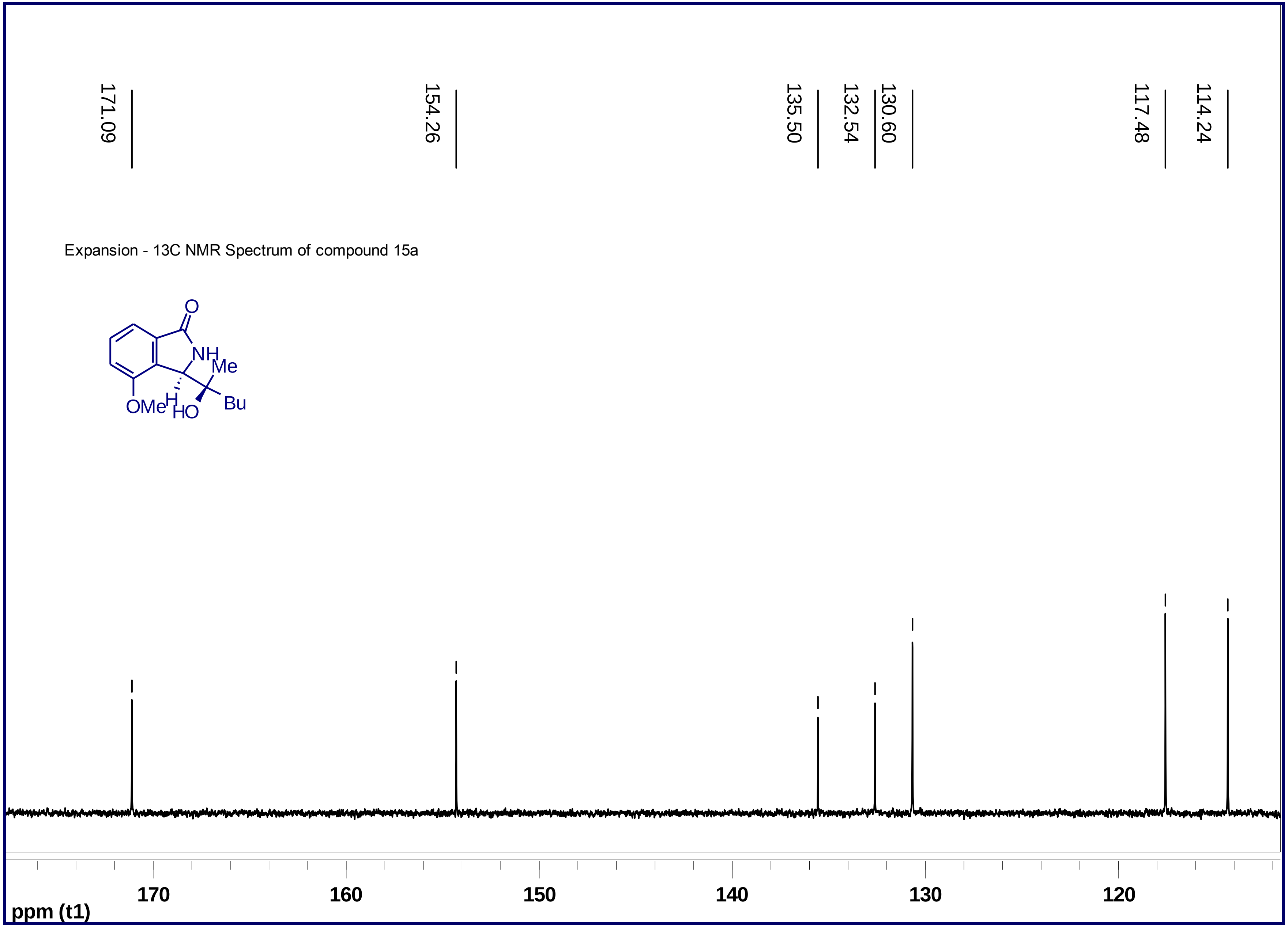

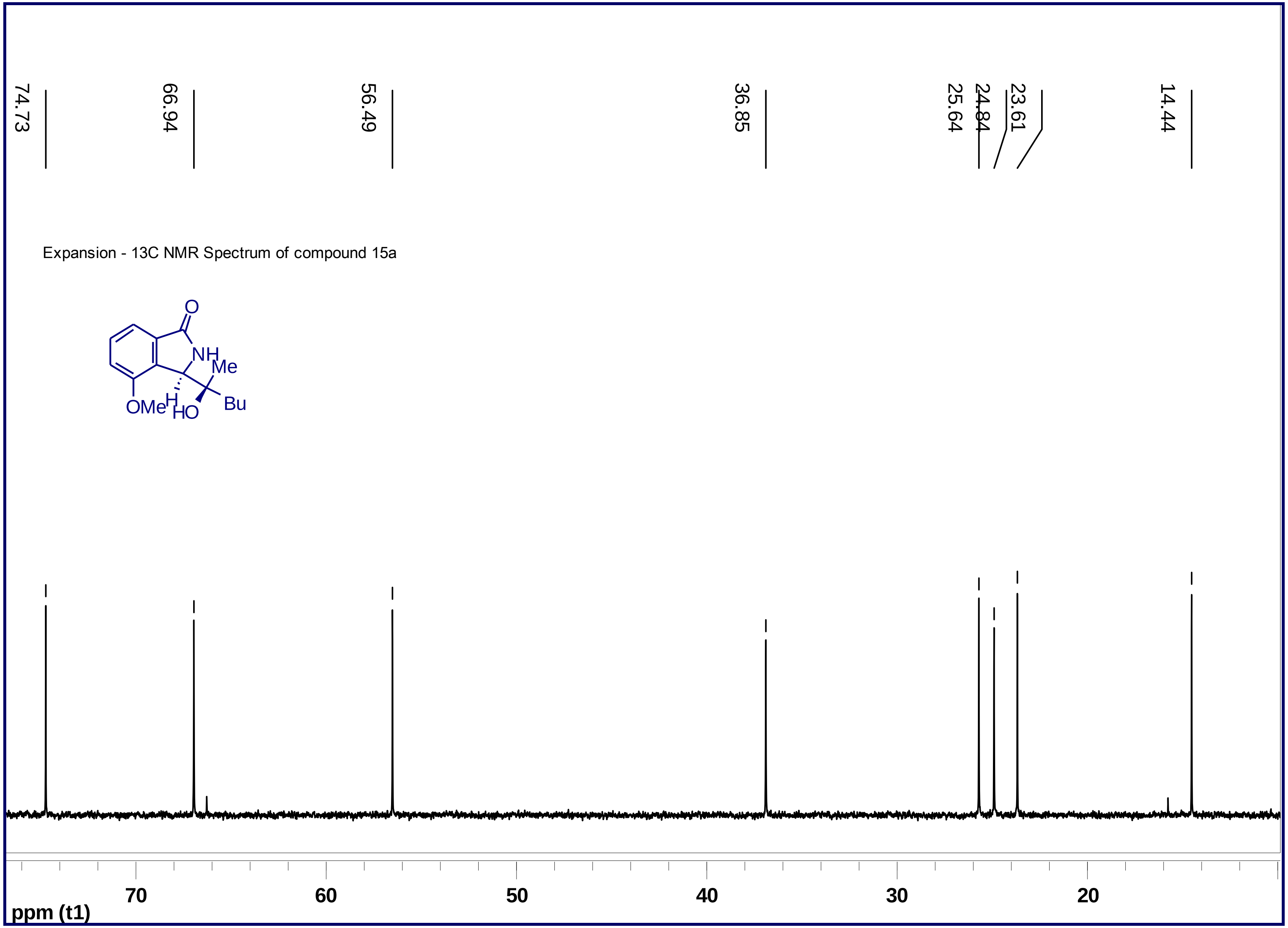
74.73
66.94
56.49
36.85
25.64
24.84
23.61
14.44
Expansion - 13C NMR Spectrum of compound 15a
O
NH
Me
Bu
OMe
H
HO
70
60
50
40
30
20
ppm (t1)

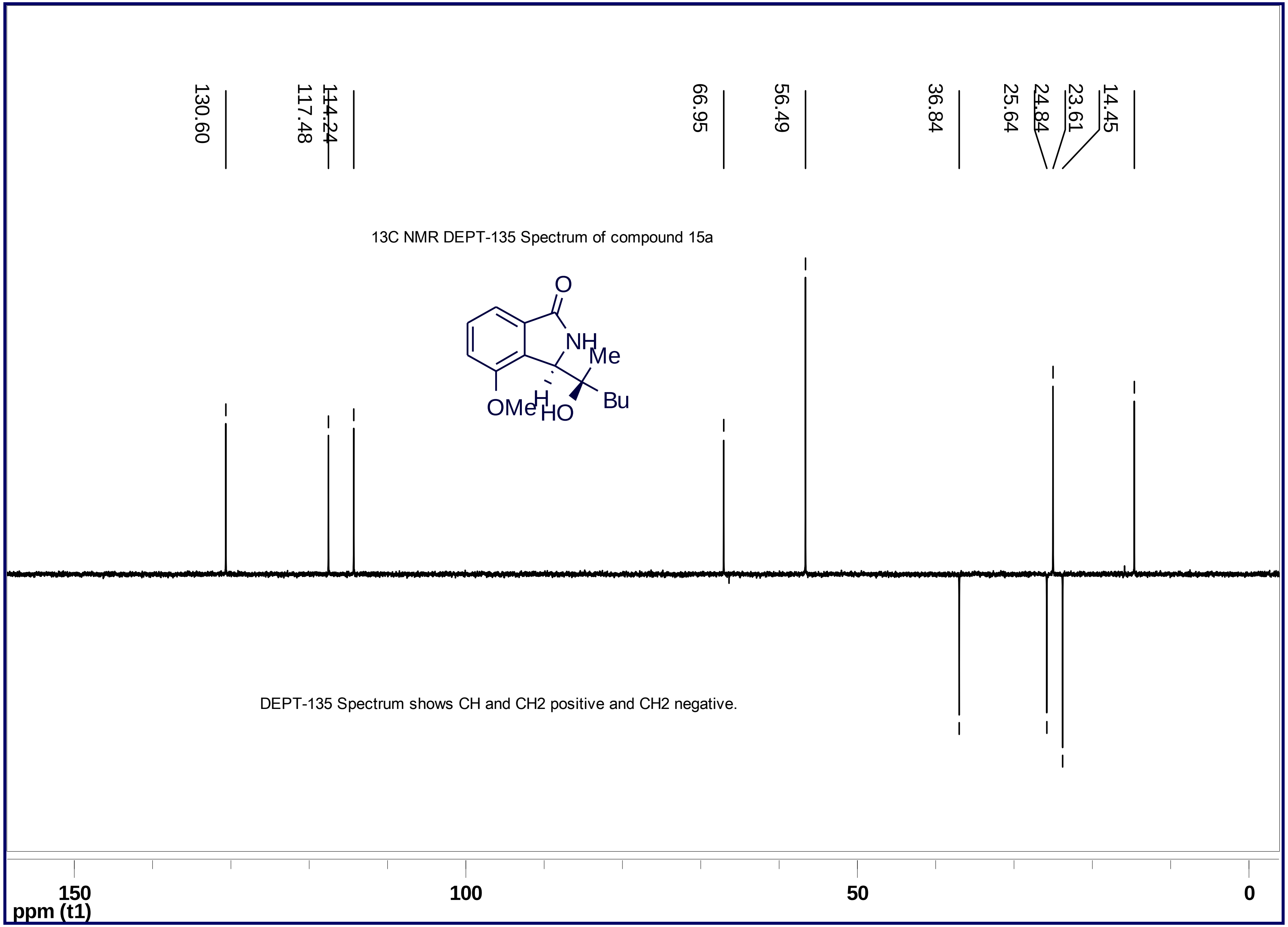

130.60
117.48
114.24
66.95
56.49
36.84
25.64
24.84
23.61
14.45
13C NMR DEPT-135 Spectrum of compound 15a
O
NH
Me
Bu
OMe
H
HO
DEPT-135 Spectrum shows CH and CH2 positive and CH2 negative.
150
100
50
0
ppm (t1)

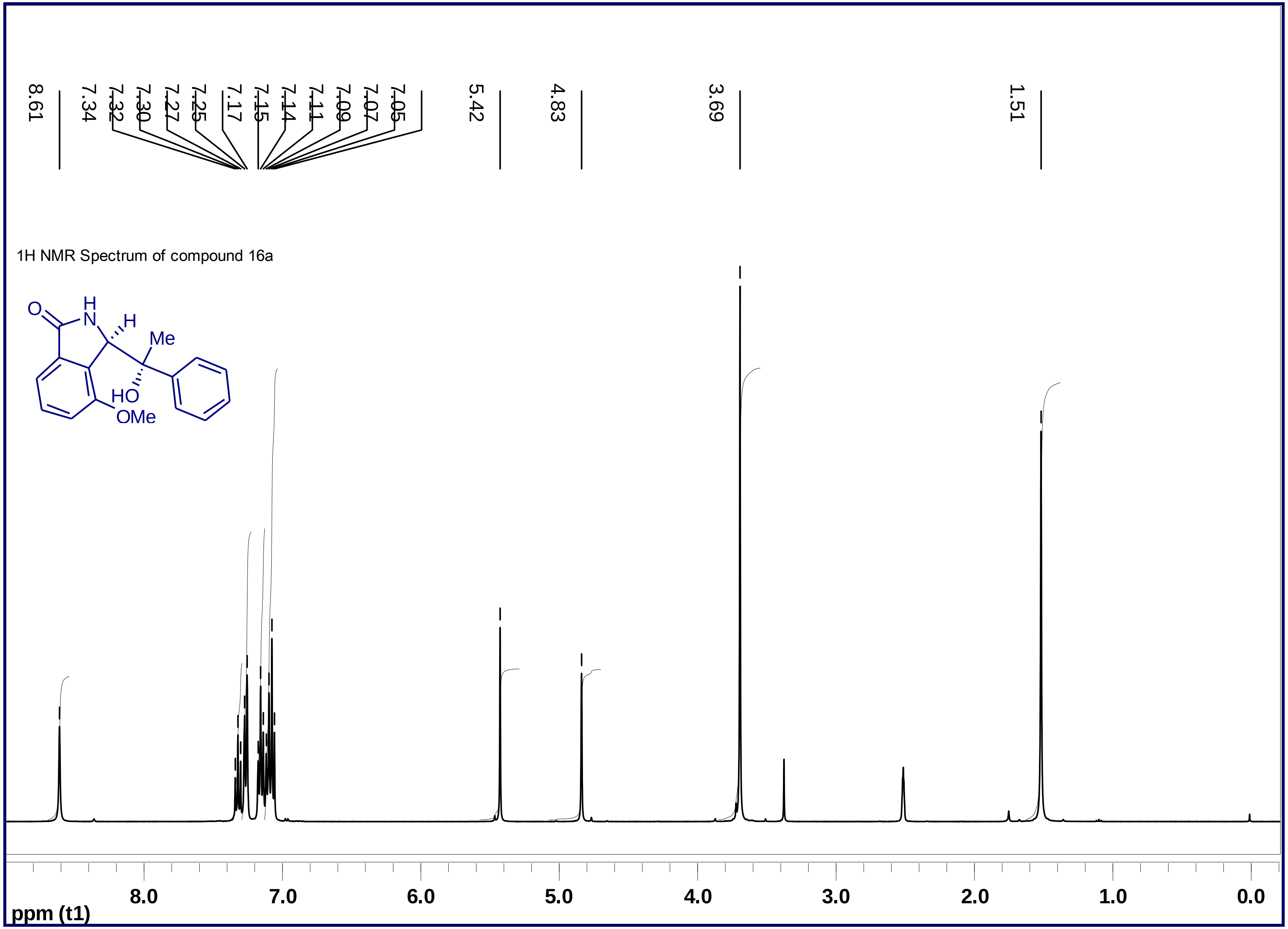
8.61
7.34
7.32
7.30
7.27
7.25
7.17
7.15
7.14
7.11
7.09
7.07
7.05
5.42
4.83
3.69
1.51
1H NMR Spectrum of compound 16a
O
H
N
H
Me
HO
OMe
8.0
7.0
6.0
5.0
4.0
3.0
2.0
1.0
0.0
ppm (t1)

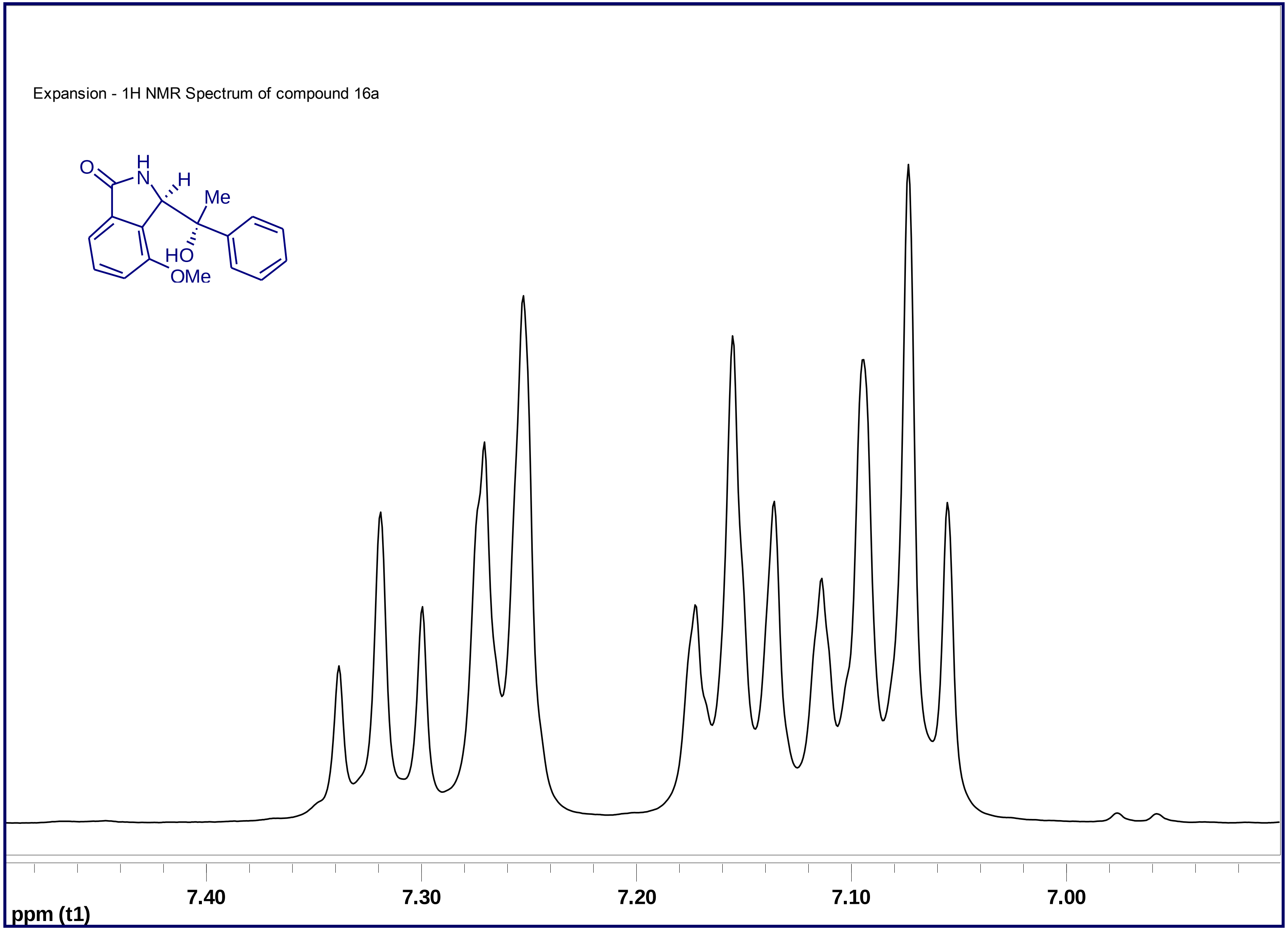
Expansion - 1H NMR Spectrum of compound 16a
O
H
N
H
Me
HO
OMe
7.40
7.30
7.20
7.10
7.00
ppm (t1)

Expansion - 1H NMR Spectrum of compound 16a

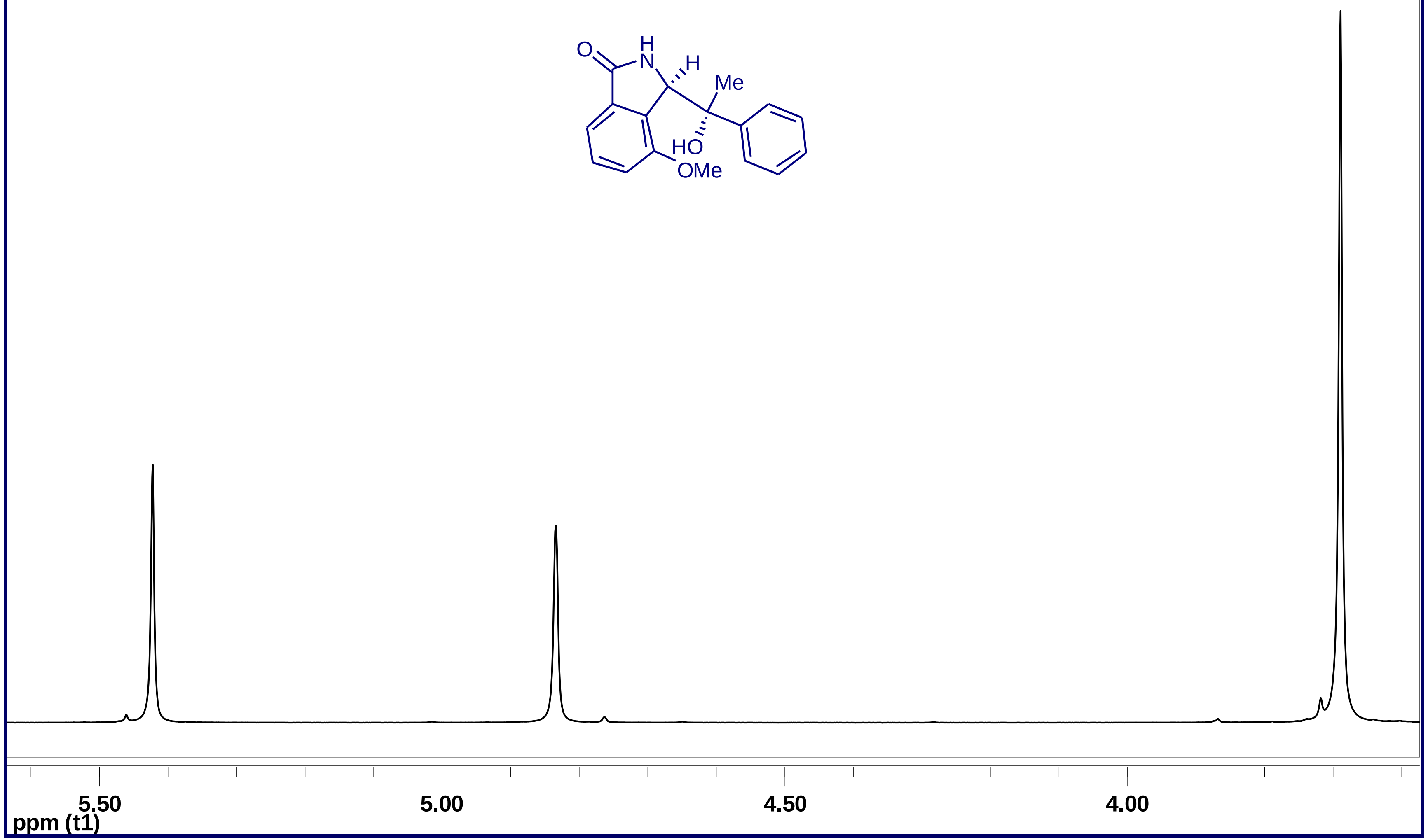

Expansion - 1H NMR Spectrum of compound 16a

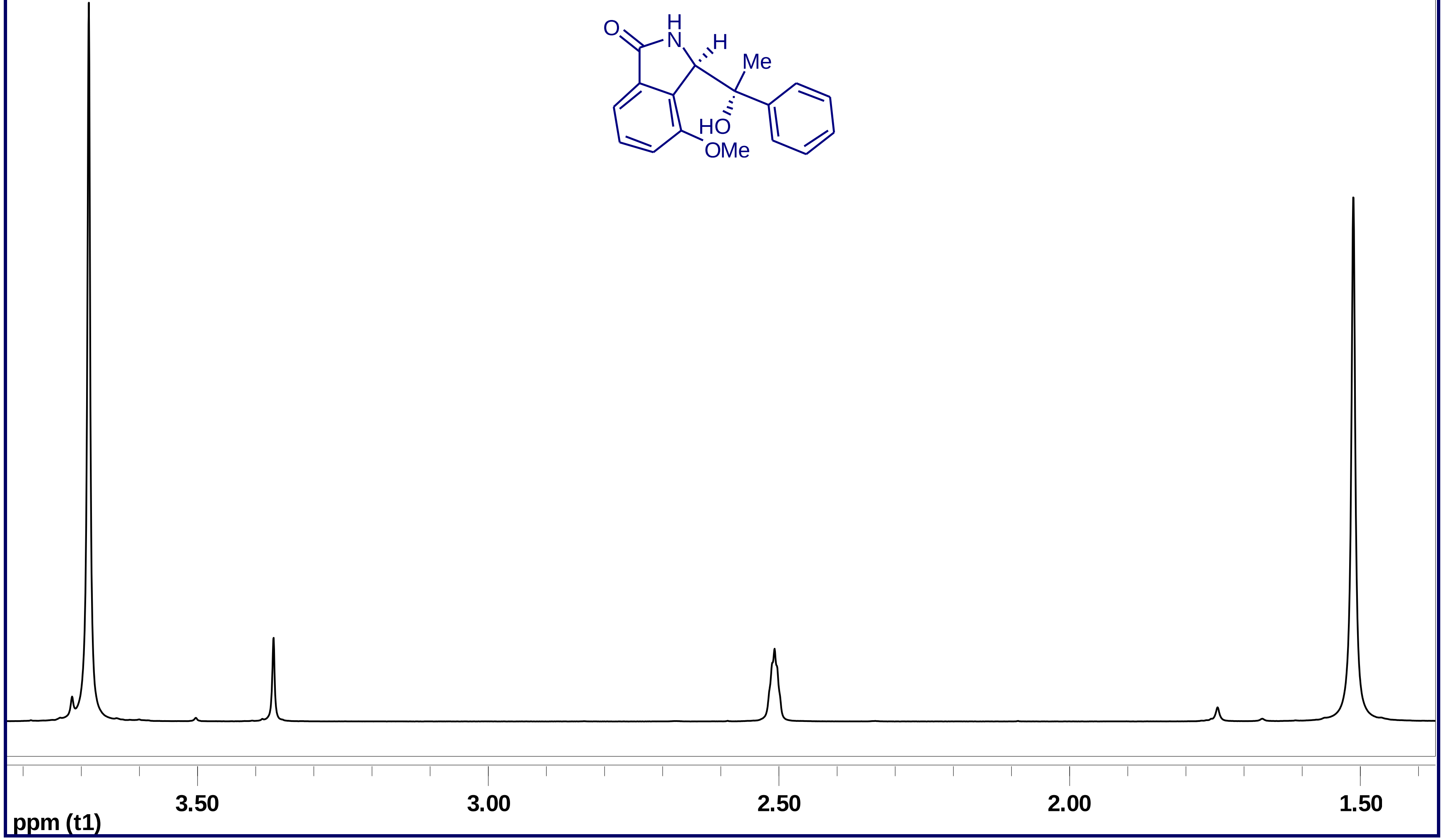

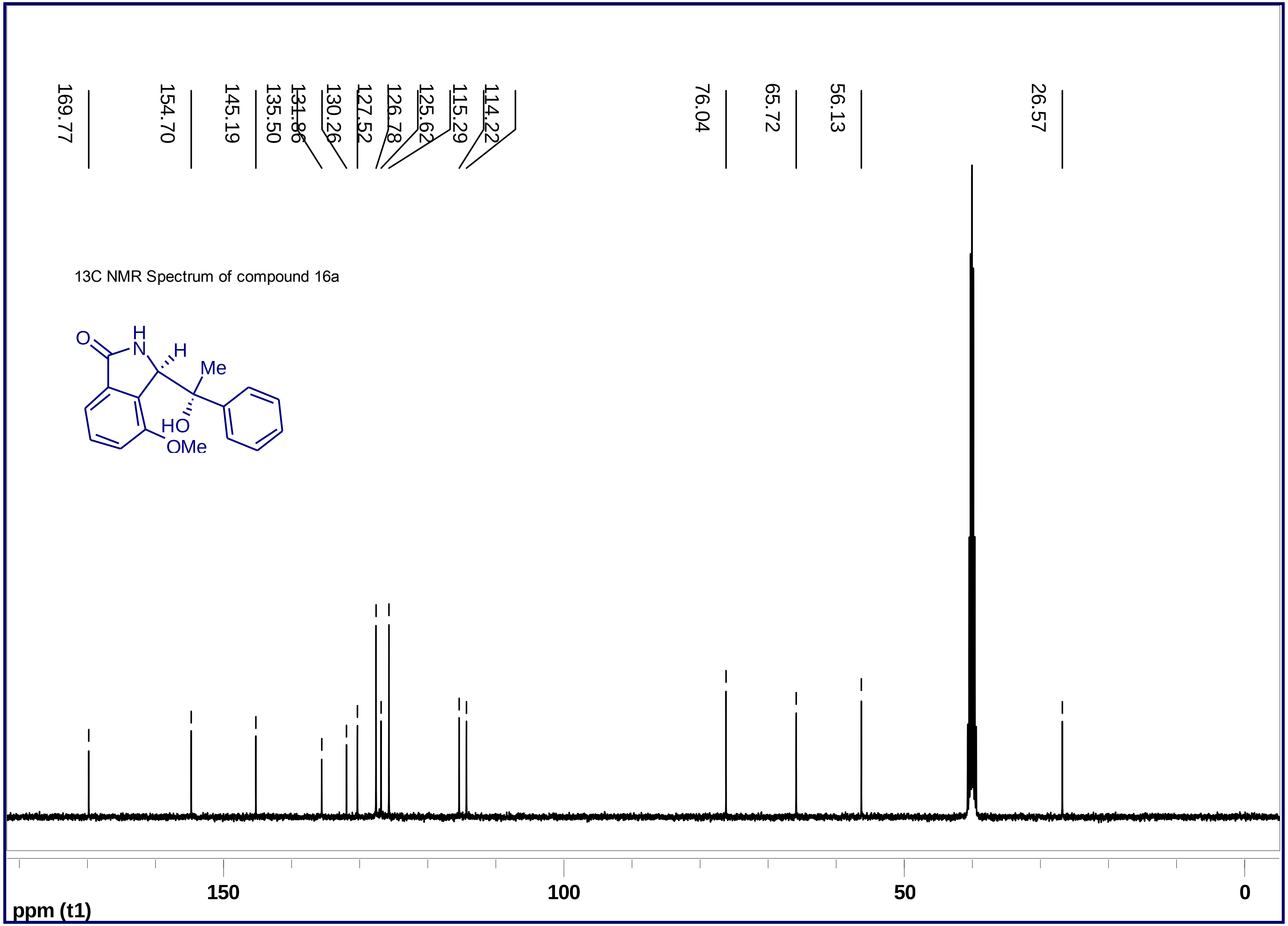
169.77
154.70
145.19
135.50
131.86
130.26
127.52
126.78
125.62
115.29
114.22
76.04
65.72
56.13
26.57
13C NMR Spectrum of compound 16a
O
H
N
H
Me
HO
OMe
150
100
50
0
ppm (t1)

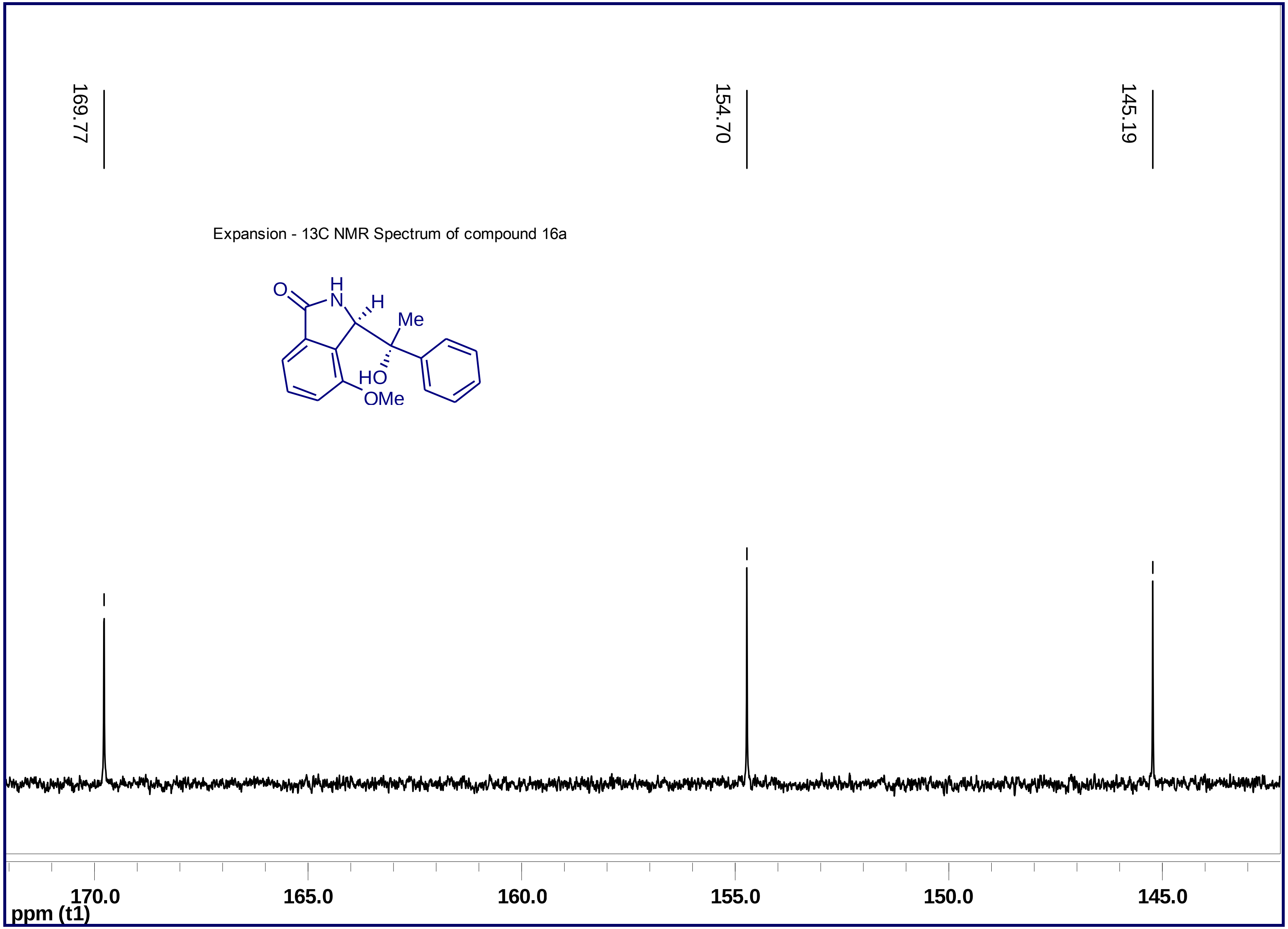
169.77
154.70
145.19
Expansion - 13C NMR Spectrum of compound 16a
O
H
N
H
Me
HO
OMe
170.0
165.0
160.0
155.0
150.0
145.0
ppm (t1)

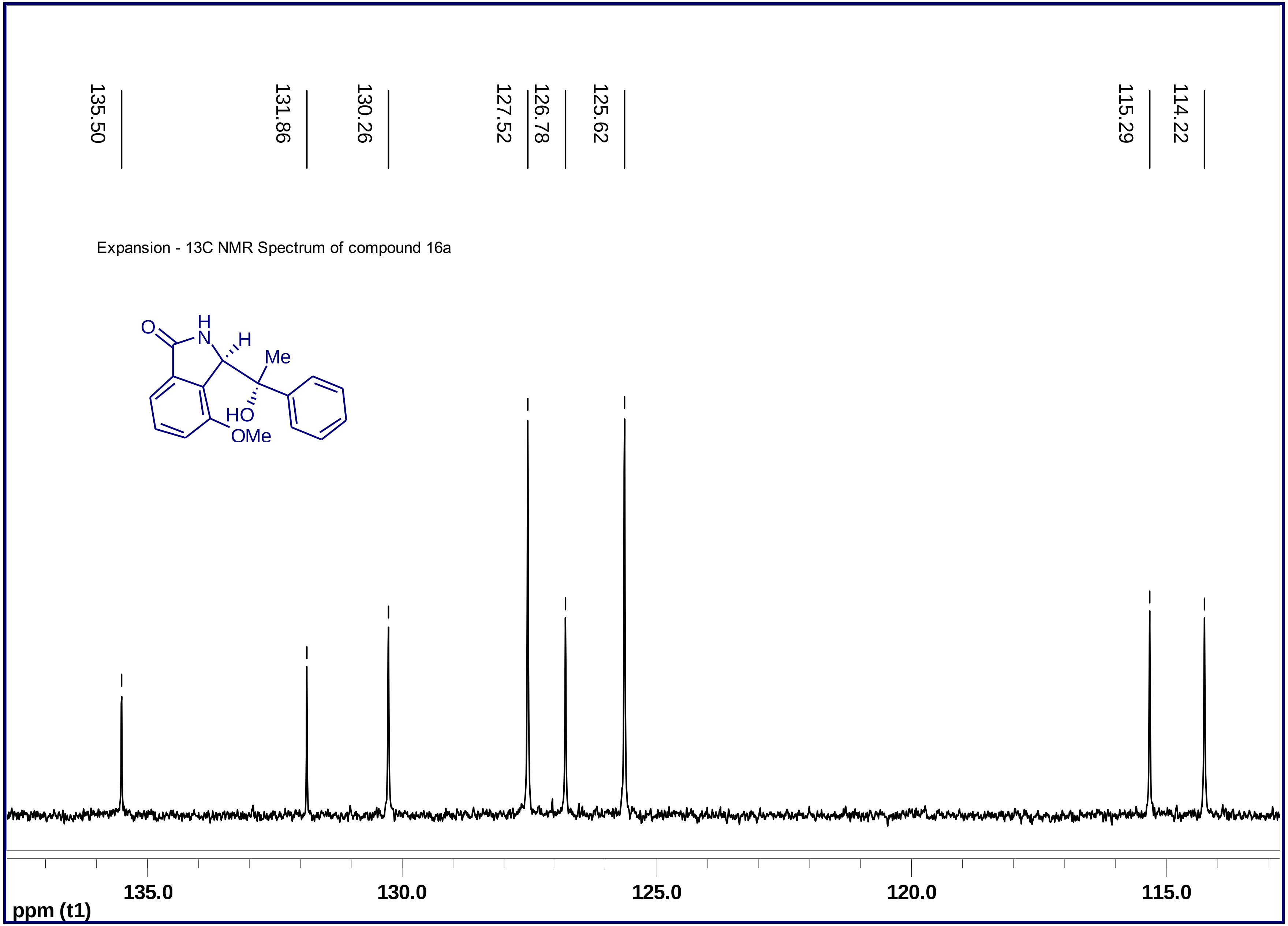
135.50
131.86
130.26
127.52
126.78
125.62
115.29
114.22
Expansion - 13C NMR Spectrum of compound 16a
O
H
N
H
Me
HO
OMe
135.0
130.0
125.0
120.0
115.0
ppm (t1)

Expansion - 13C NMR Spectrum of compound 16a

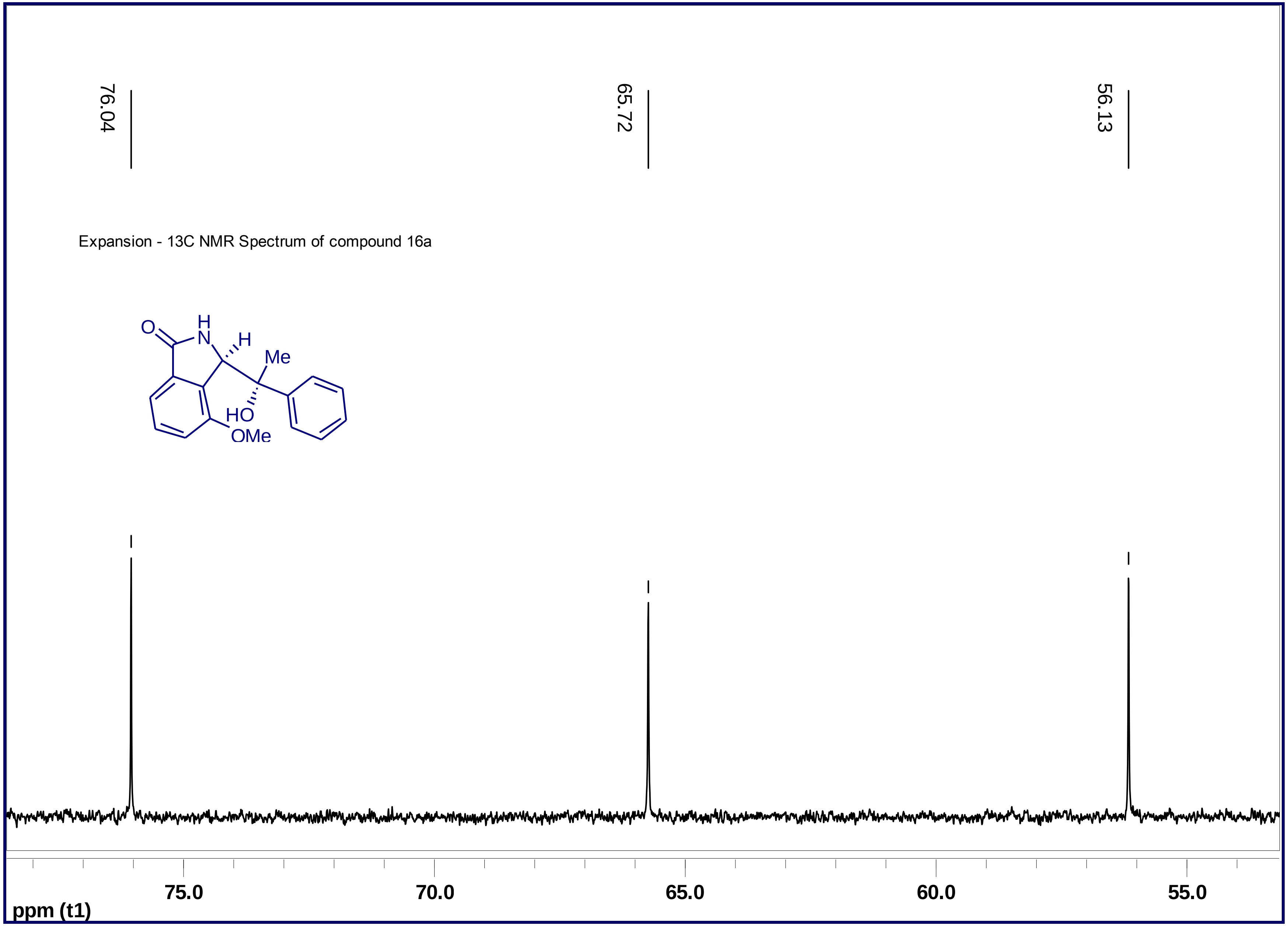

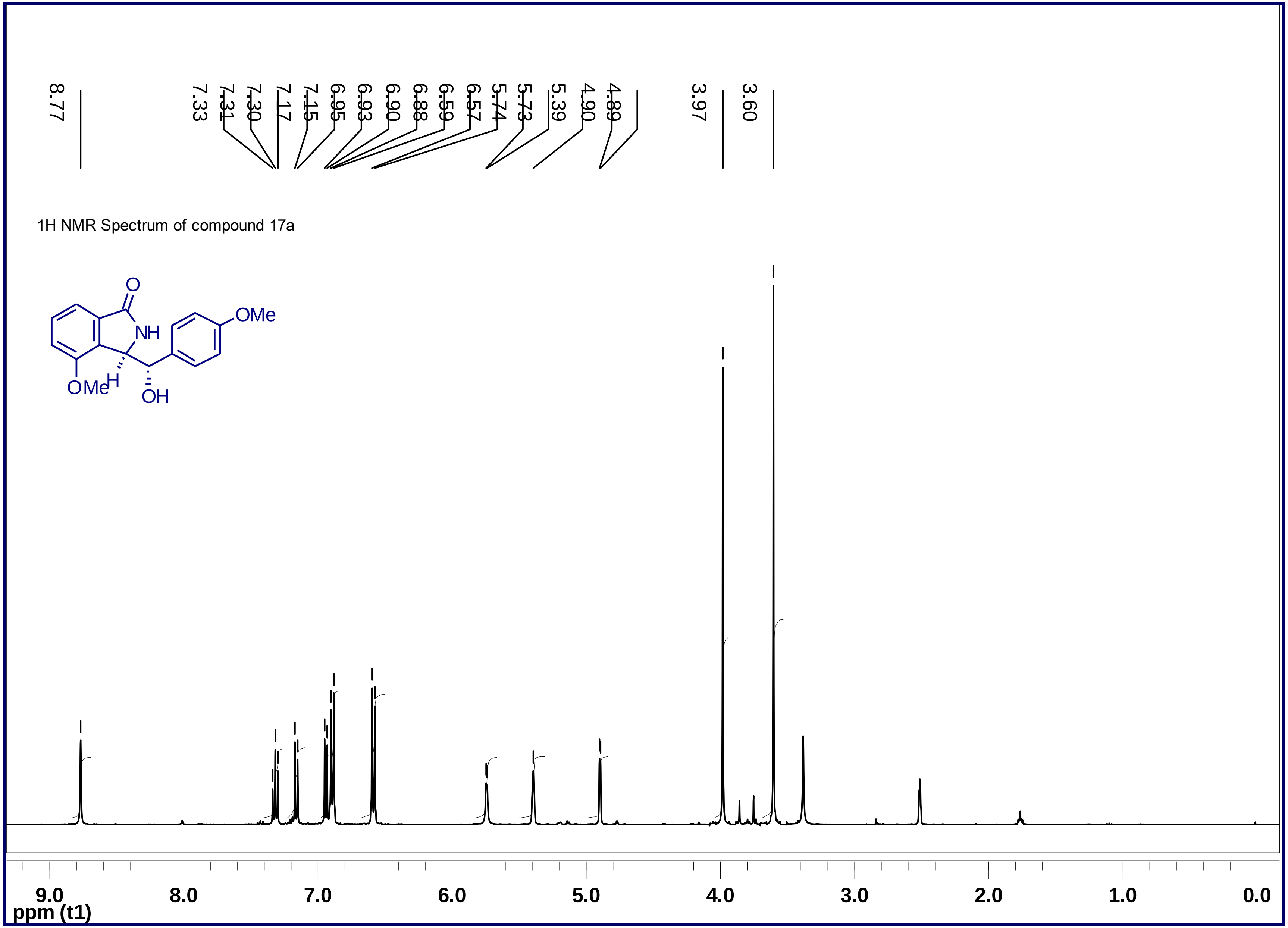

8.77
7.33
7.31
7.30
7.17
7.15
6.95
6.93
6.90
6.88
6.59
6.57
5.74
5.73
5.39
4.90
4.89
3.97
3.60
1H NMR Spectrum of compound 17a
O
NH
OMe
OMe
H
OH
9.0
8.0
7.0
6.0
5.0
4.0
3.0
2.0
1.0
0.0
ppm (t1)

Expansion - 1H NMR Spectrum of compound 17a

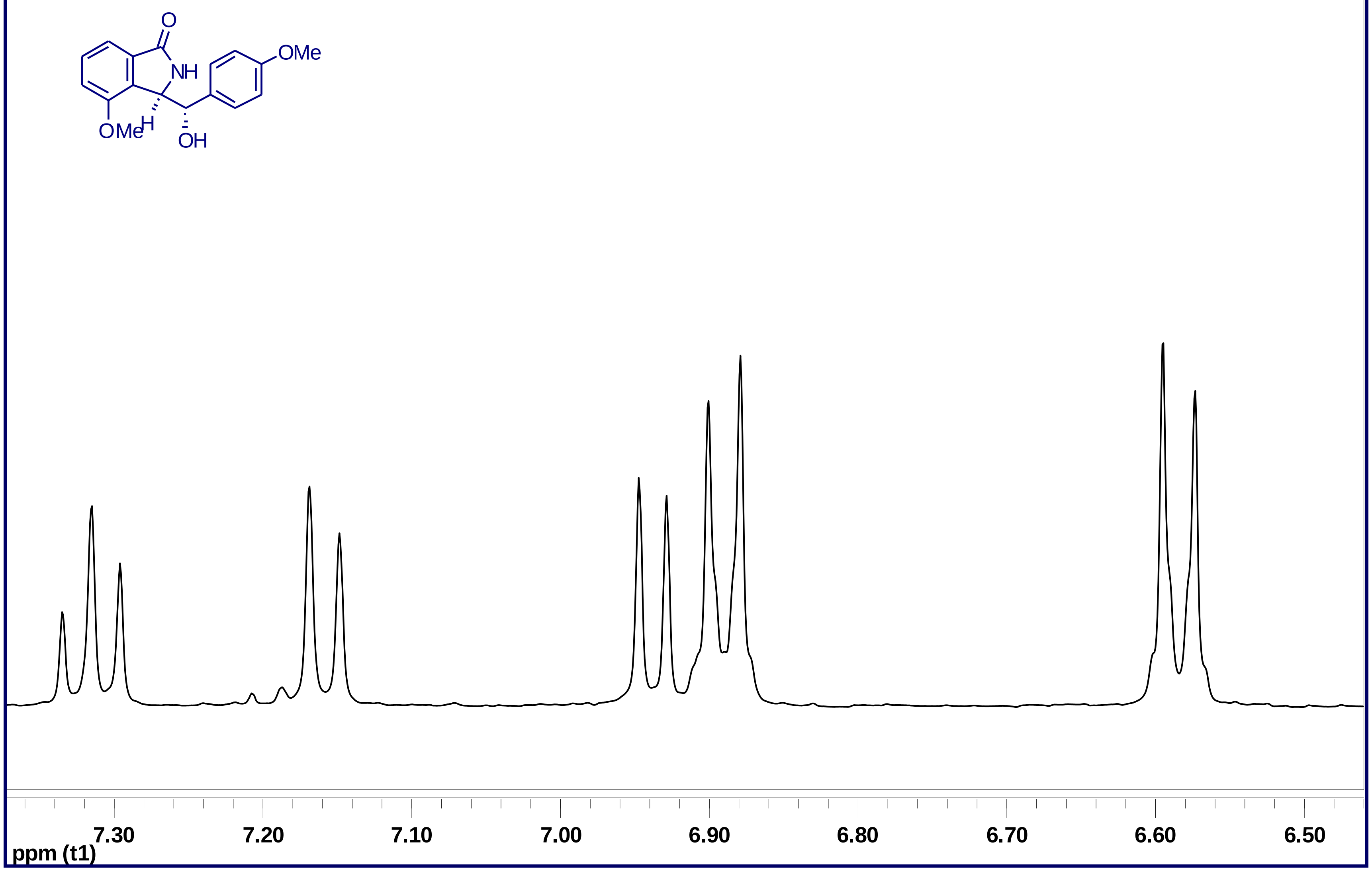

Expansion - 1H NMR Spectrum of compound 17a

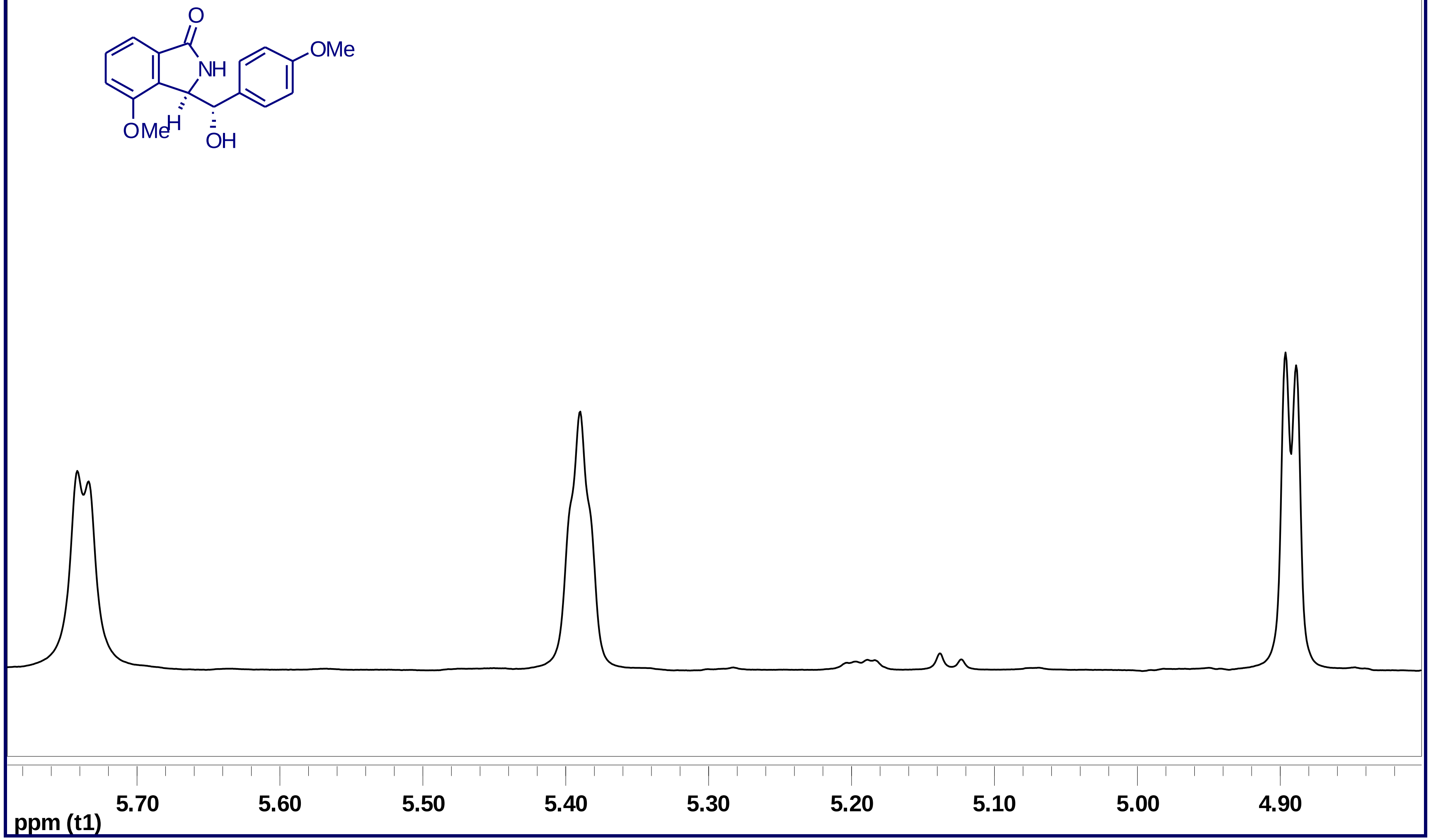

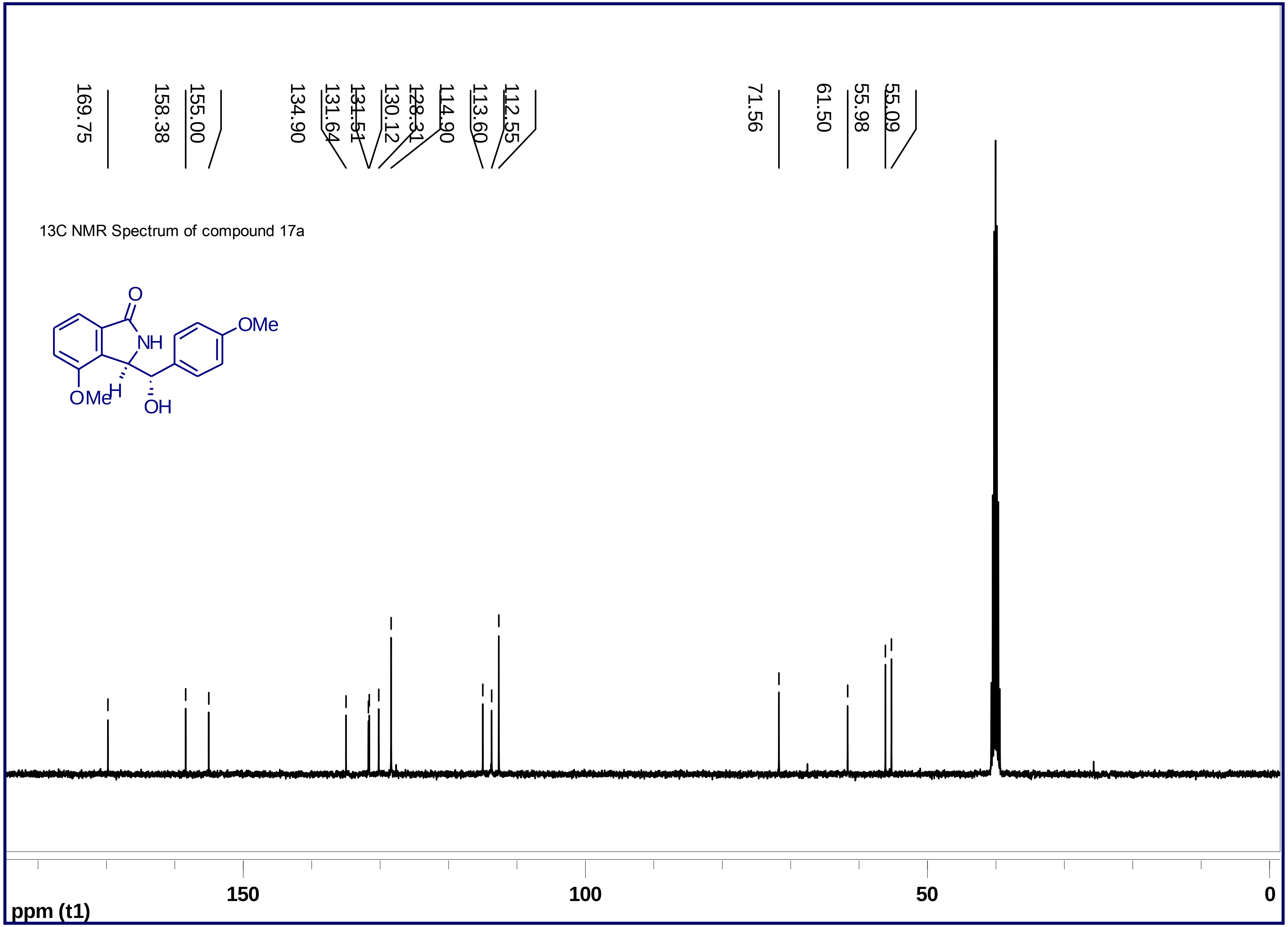

169.75
158.38
155.00
134.90
131.64
131.51
130.12
128.31
114.90
113.60
112.55
71.56
61.50
55.98
55.09
13C NMR Spectrum of compound 17a
O
NH
OMe
OMe
H
OH
150
100
50
0
ppm (t1)

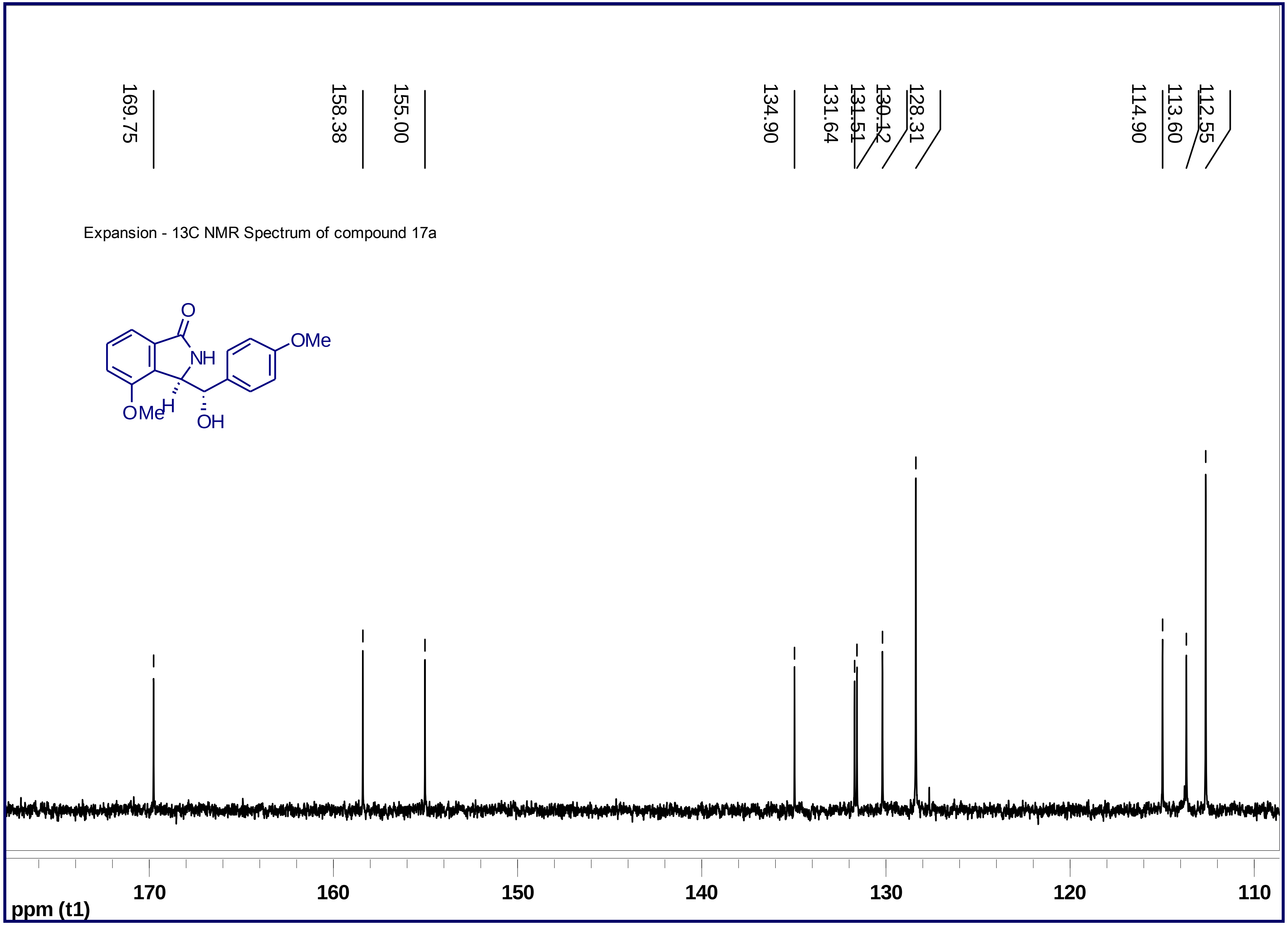

169.75
158.38
155.00
134.90
131.64
131.51
130.12
128.31
114.90
113.60
112.55
Expansion - 13C NMR Spectrum of compound 17a
O
NH
OMe
H
OMe
OH
170
160
150
140
130
120
110
ppm (t1)

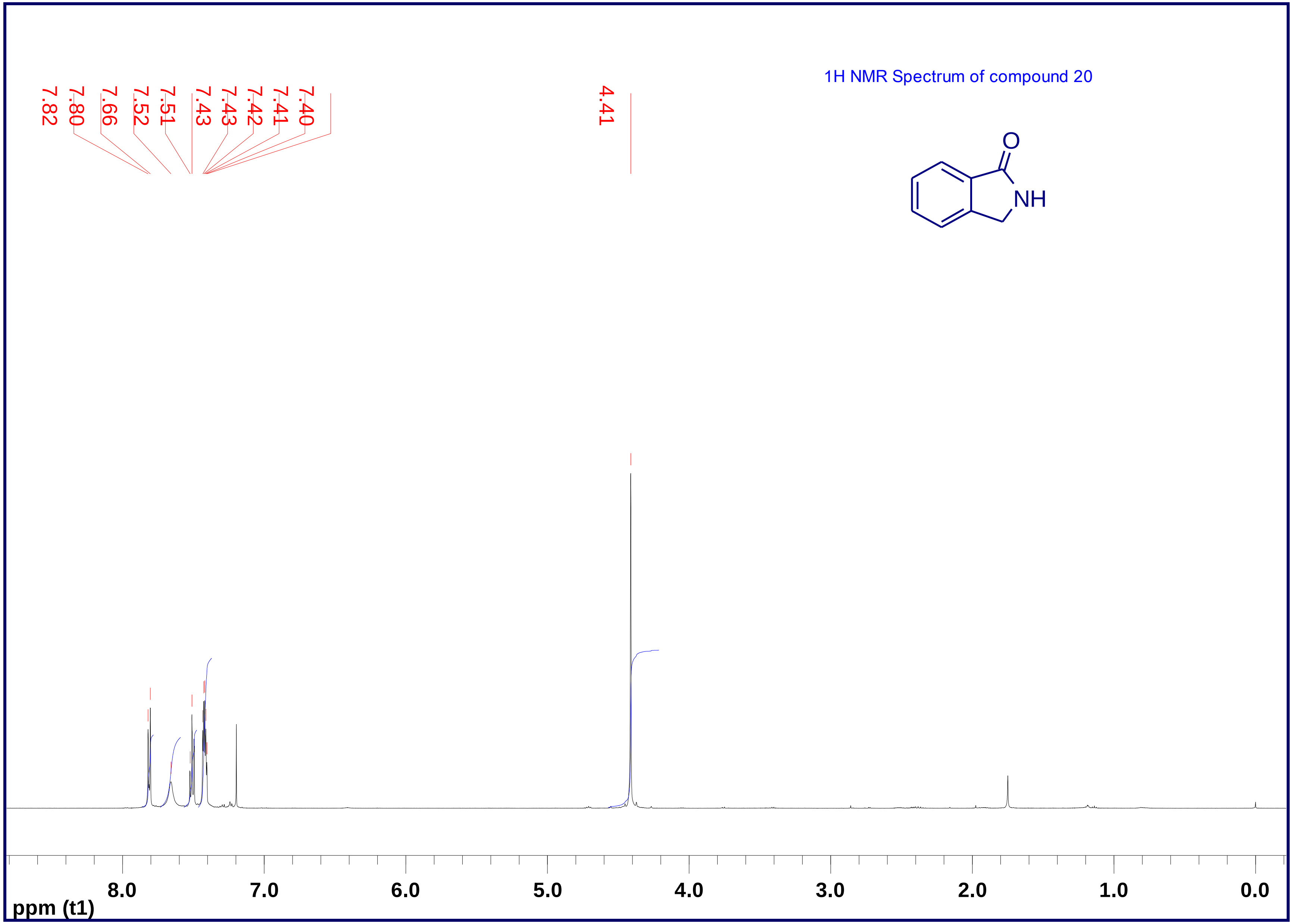
1H NMR Spectrum of compound 20
7.82
7.80
7.66
7.52
7.51
7.43
7.43
7.42
7.41
7.40
4.41
O
NH
8.0
7.0
6.0
5.0
4.0
3.0
2.0
1.0
0.0
ppm (t1)

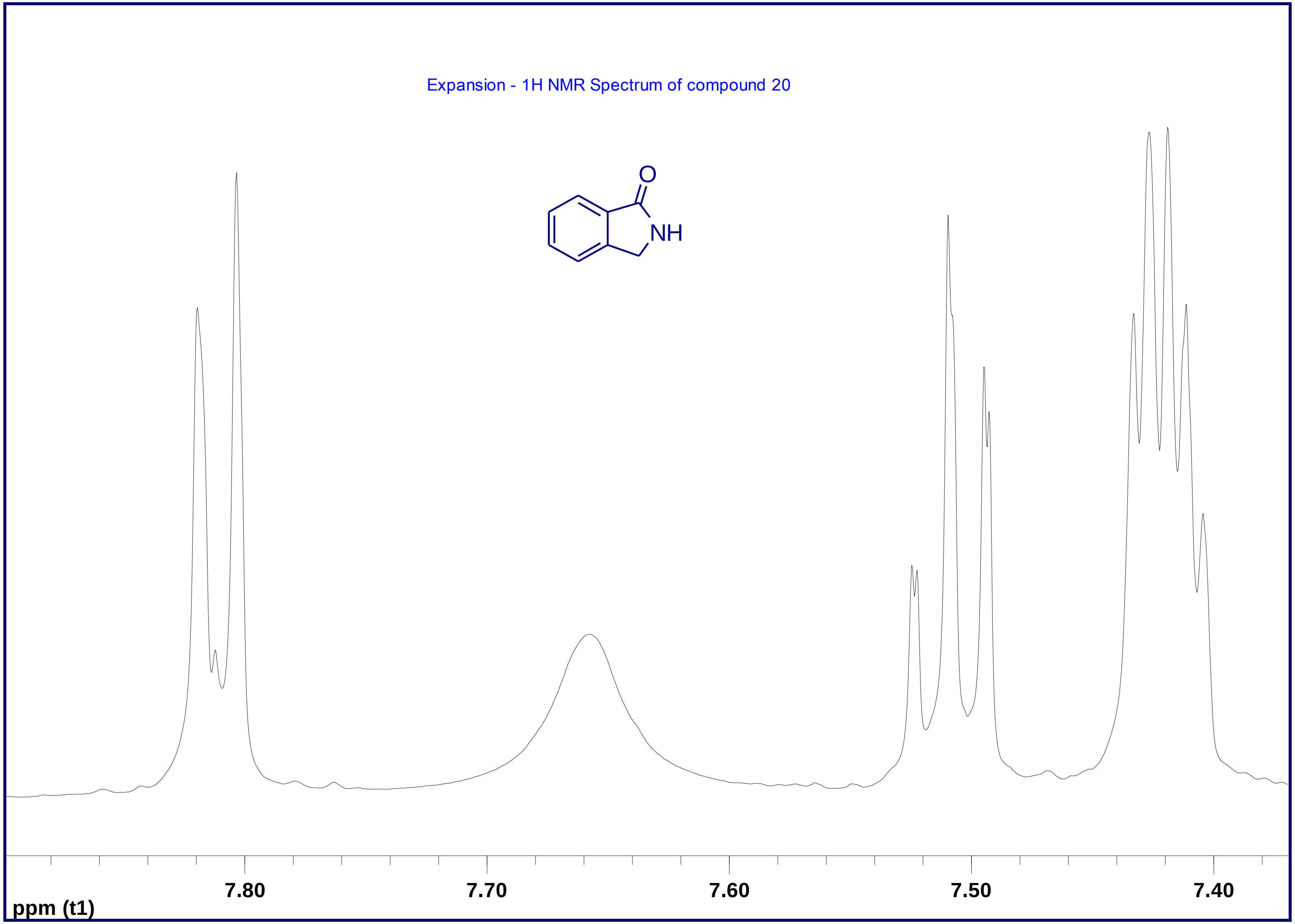
Expansion - 1H NMR Spectrum of compound 20
O
NH
7.80
7.70
7.60
7.50
7.40
ppm (t1)

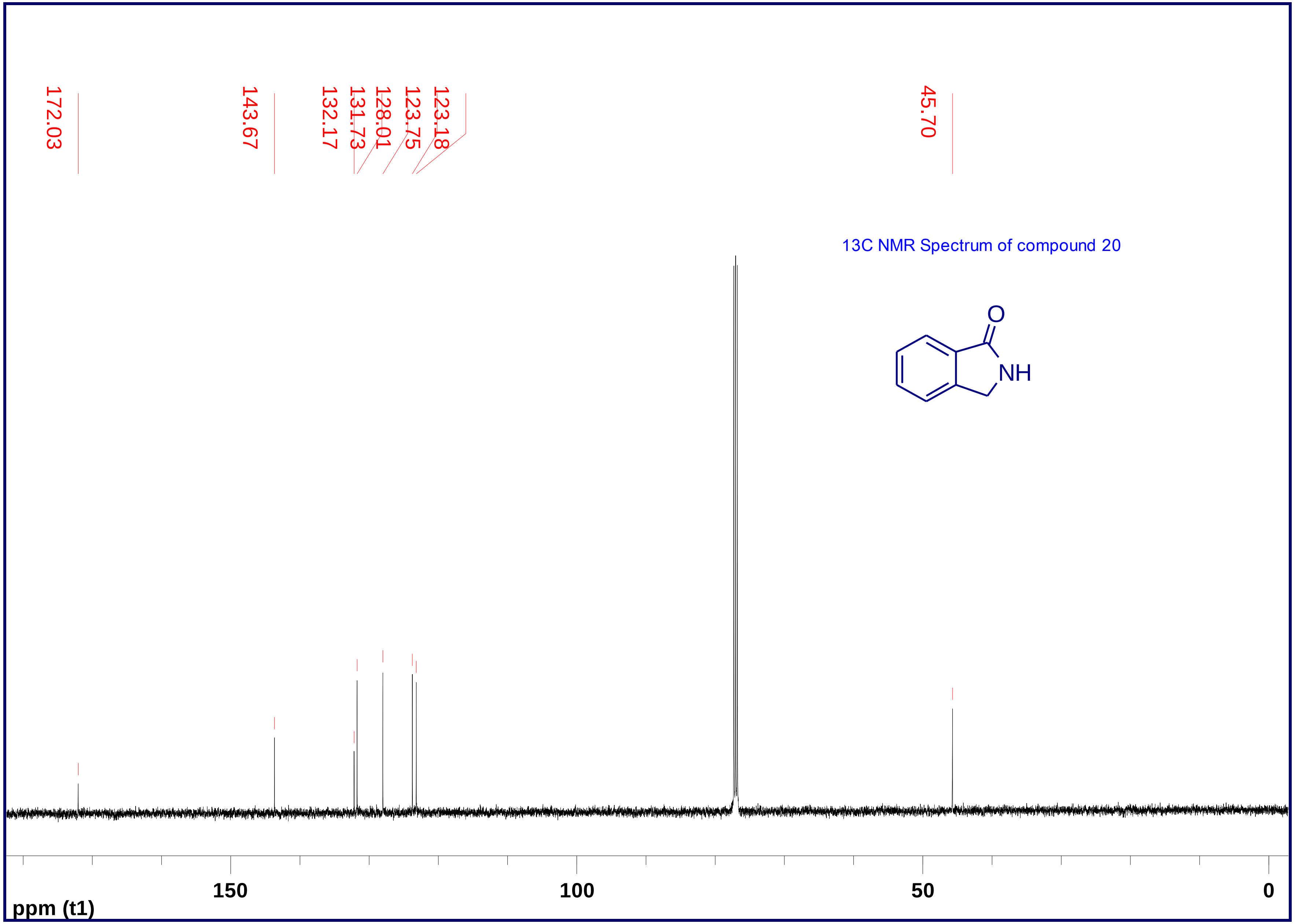
172.03
143.67
132.17
131.73
128.01
123.75
123.18
45.70
13C NMR Spectrum of compound 20
O
NH
150
100
50
0
ppm (t1)

Expansion - 13C NMR Spectrum of compound 20

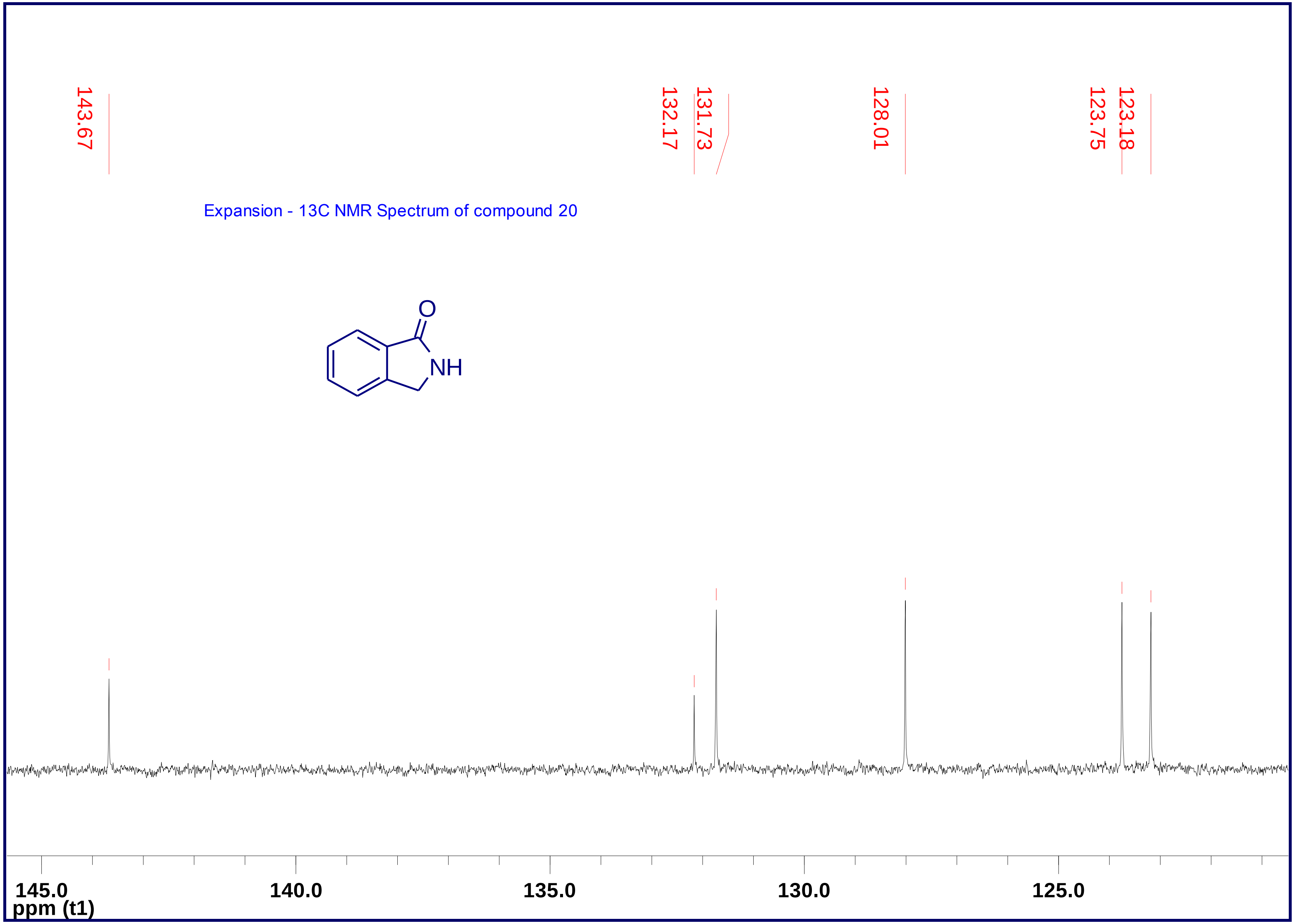

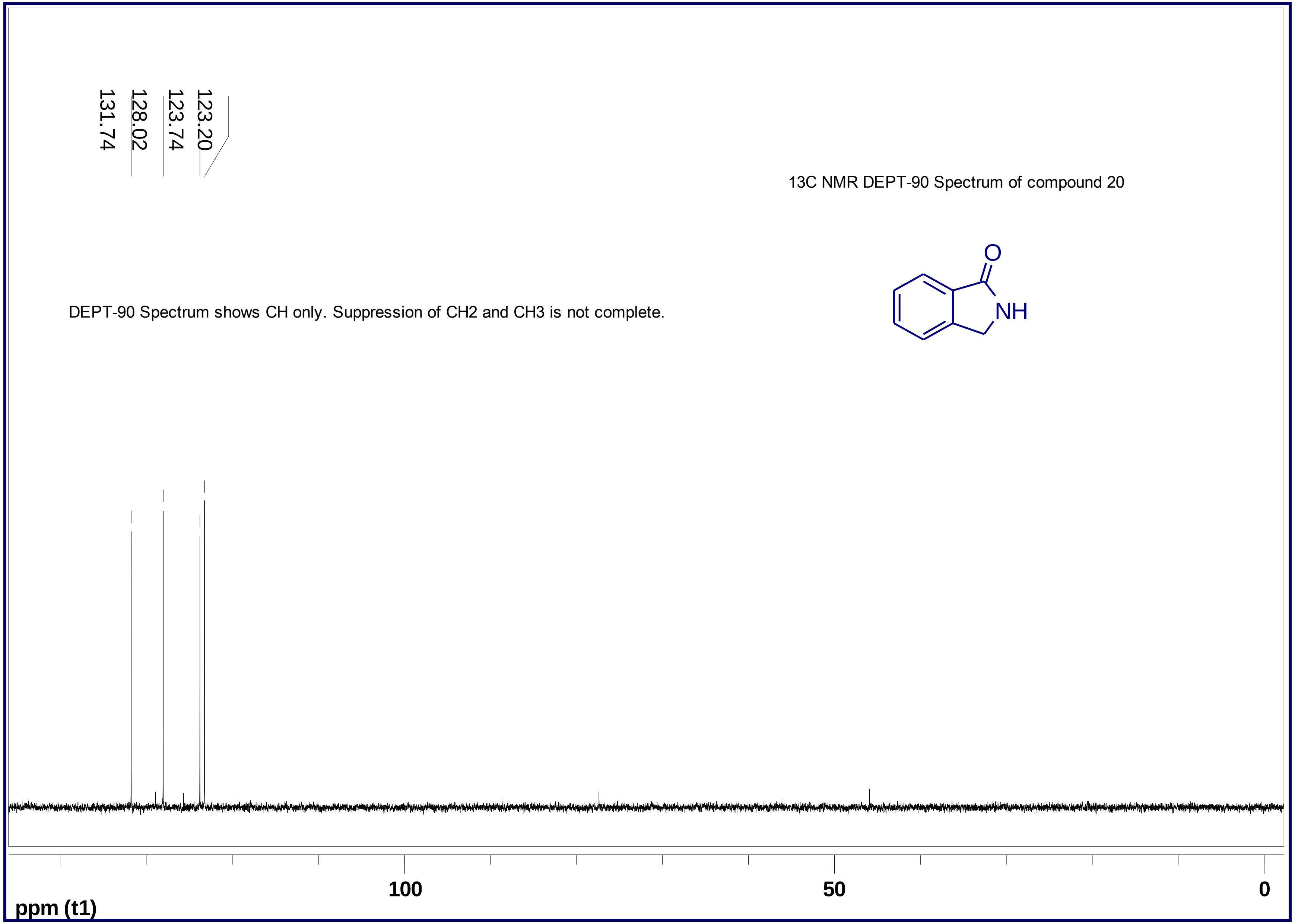
131.74
128.02
123.74
123.20
13C NMR DEPT-90 Spectrum of compound 20
O
NH
DEPT-90 Spectrum shows CH only. Suppression of CH2 and CH3 is not complete.
100
50
0
ppm (t1)

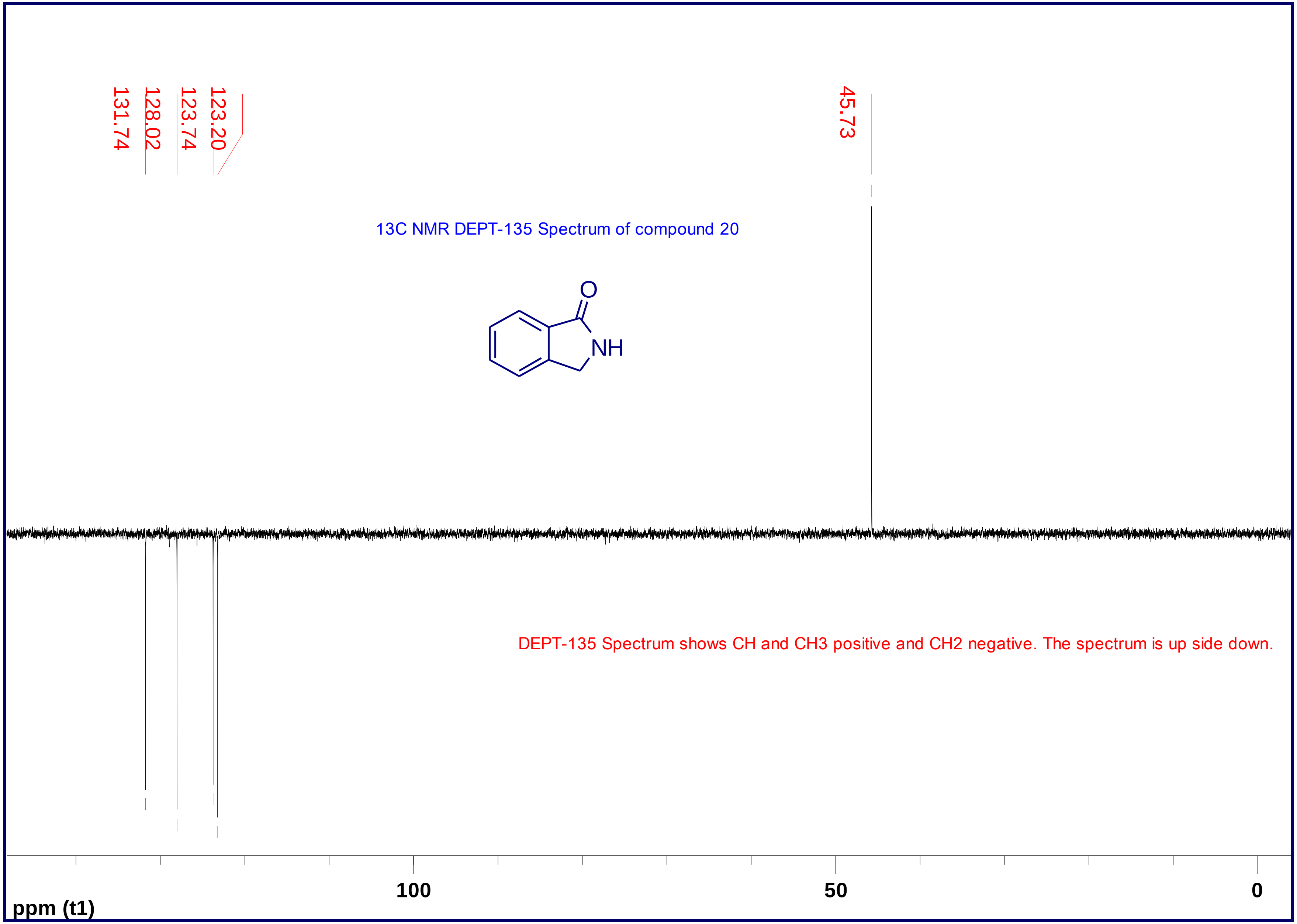
131.74
128.02
123.74
123.20
45.73
13C NMR DEPT-135 Spectrum of compound 20
O
NH
DEPT-135 Spectrum shows CH and CH3 positive and CH2 negative. The spectrum is up side down.
100
50
0
ppm (t1)

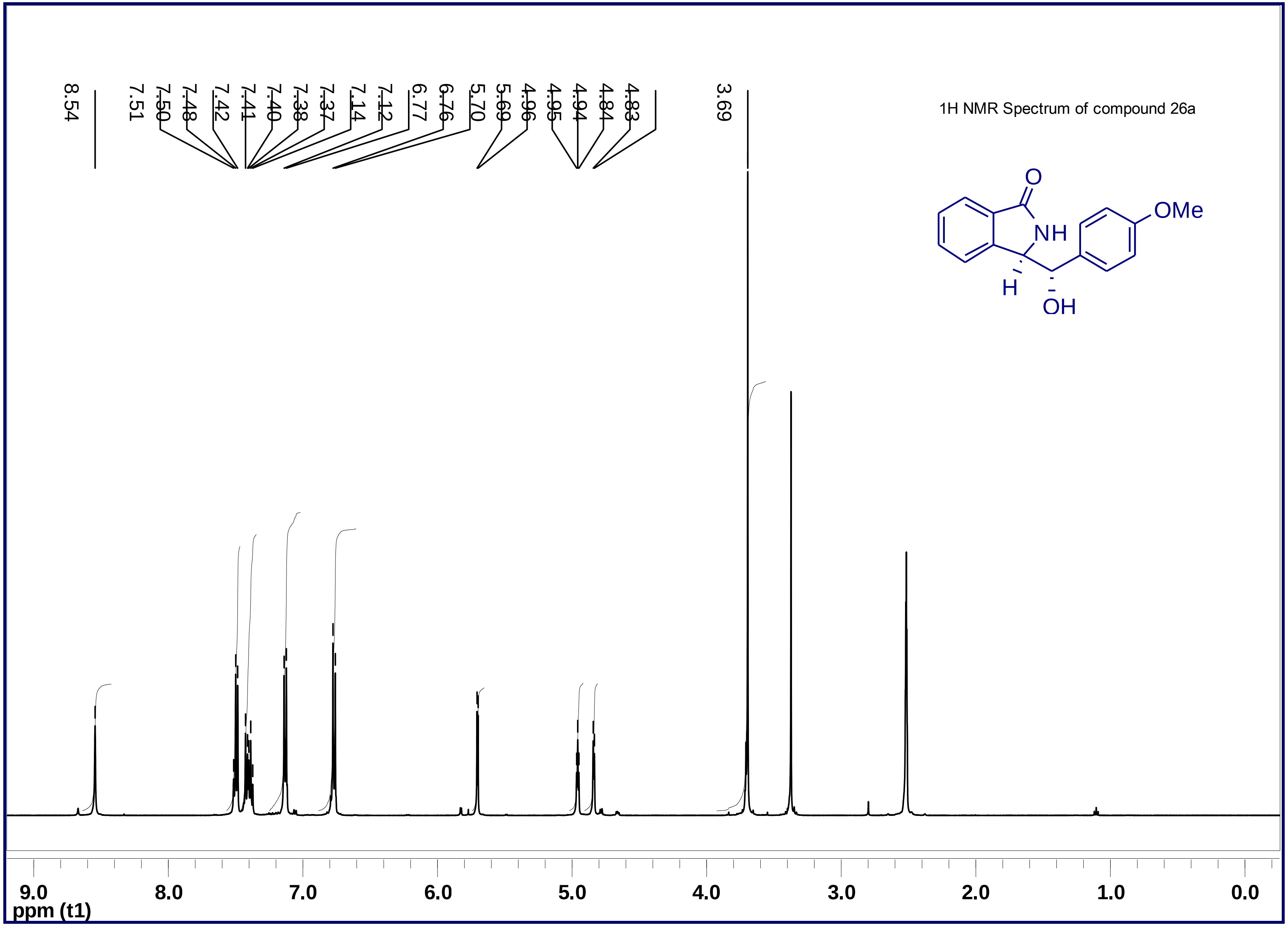
1H NMR Spectrum of compound 26a
8.54
7.51
7.50
7.48
7.42
7.41
7.40
7.38
7.37
7.14
7.12
6.77
6.76
5.70
5.69
4.96
4.95
4.94
4.84
4.83
3.69
O
NH
OMe
H
OH
9.0
8.0
7.0
6.0
5.0
4.0
3.0
2.0
1.0
0.0
ppm (t1)

Expansion - 1H NMR Spectrum of compound 26a

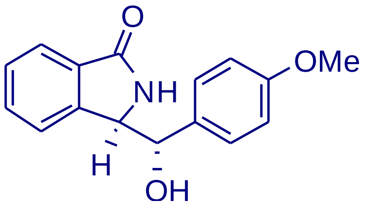

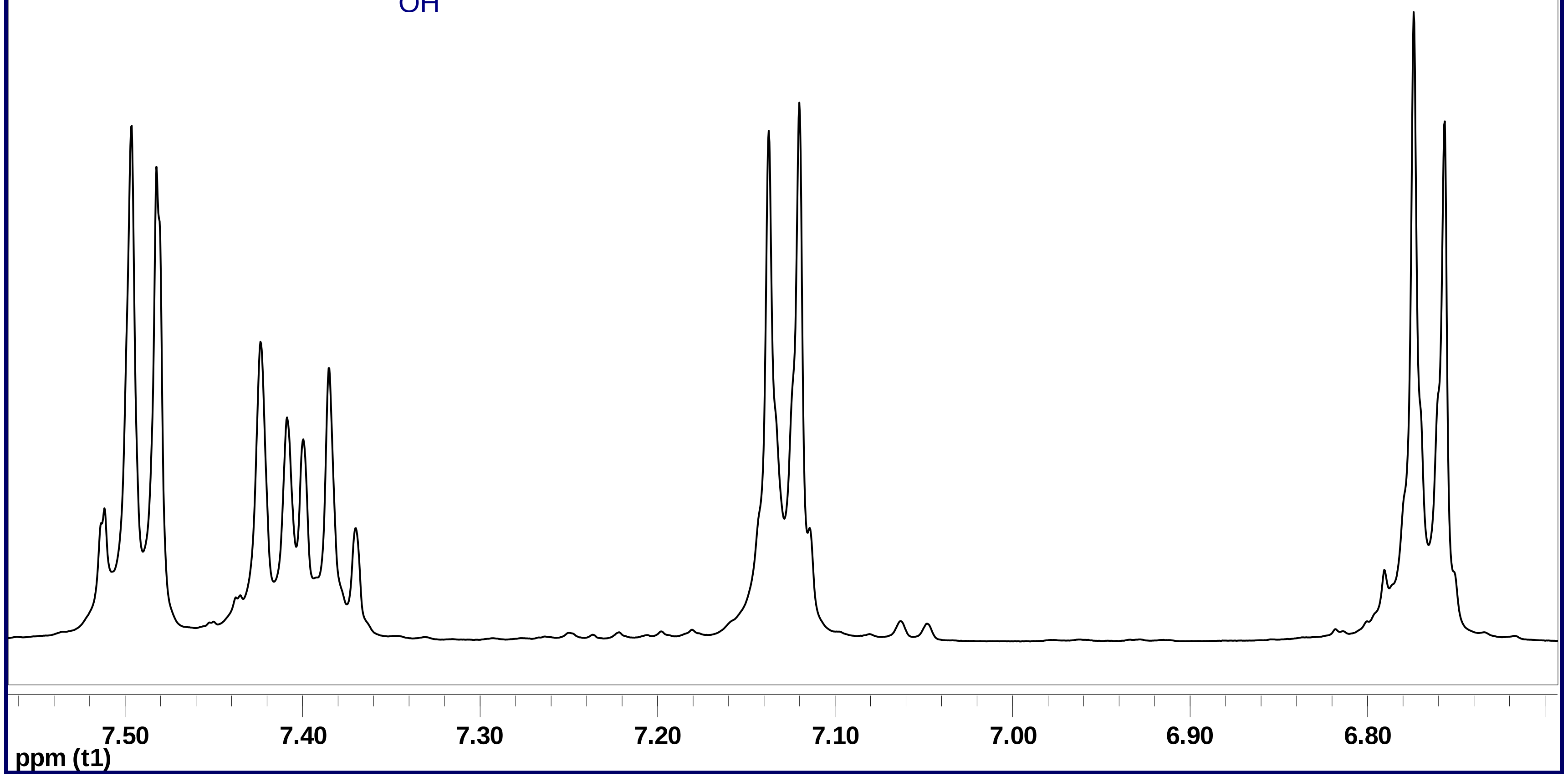

Expansion - 1H NMR Spectrum of compound 26a

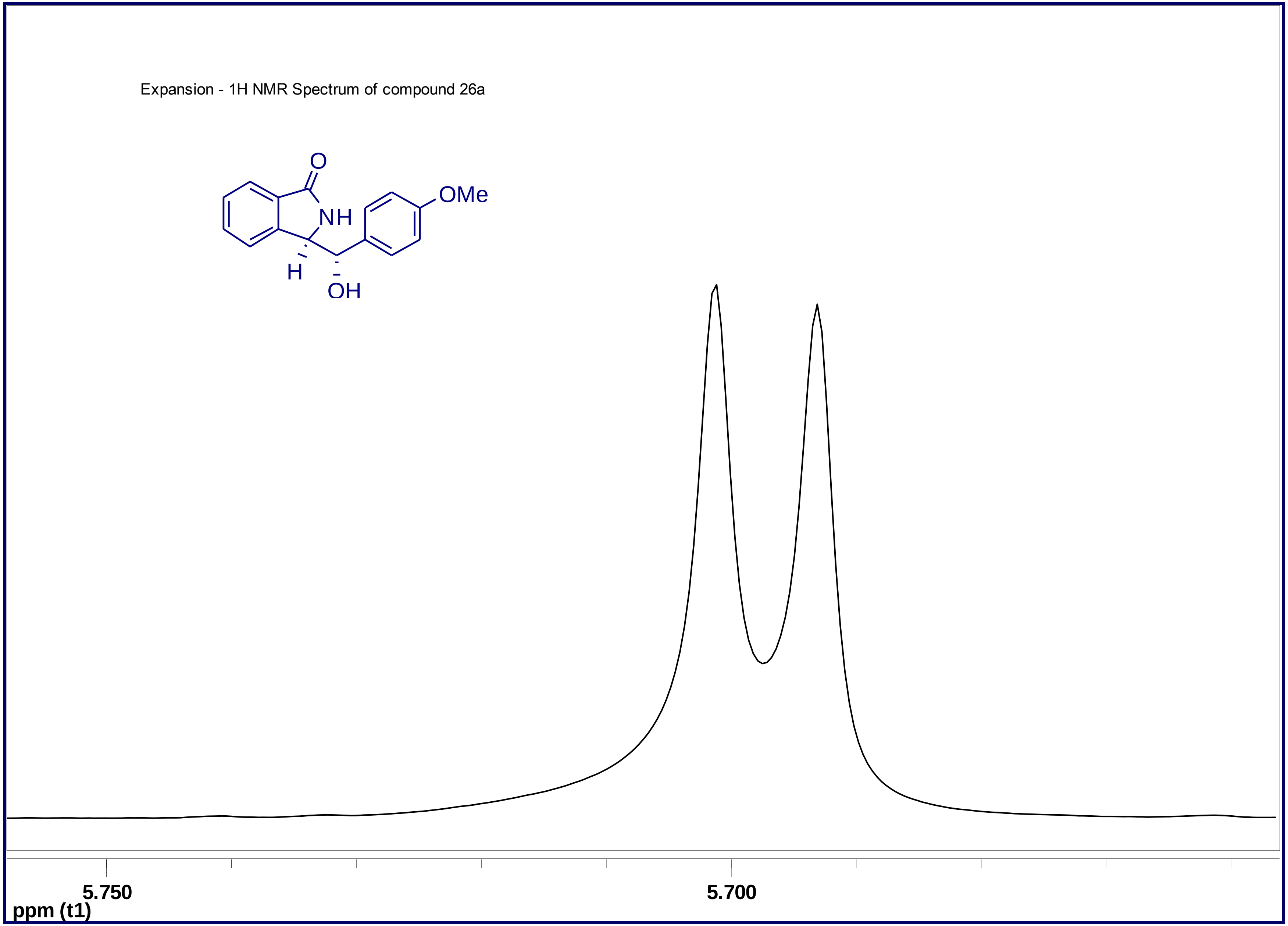

Expansion - 1H NMR Spectrum of compound 26a

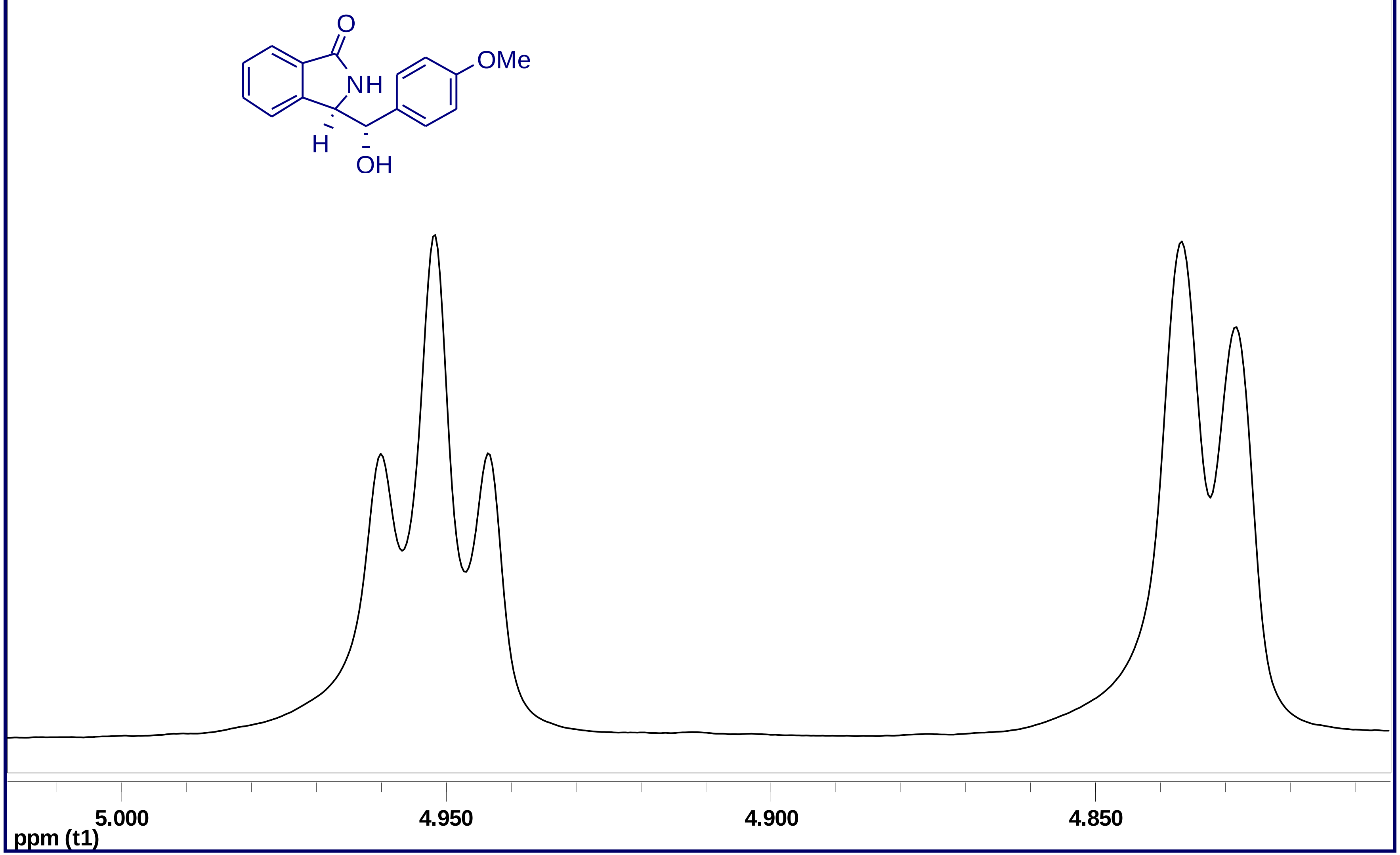

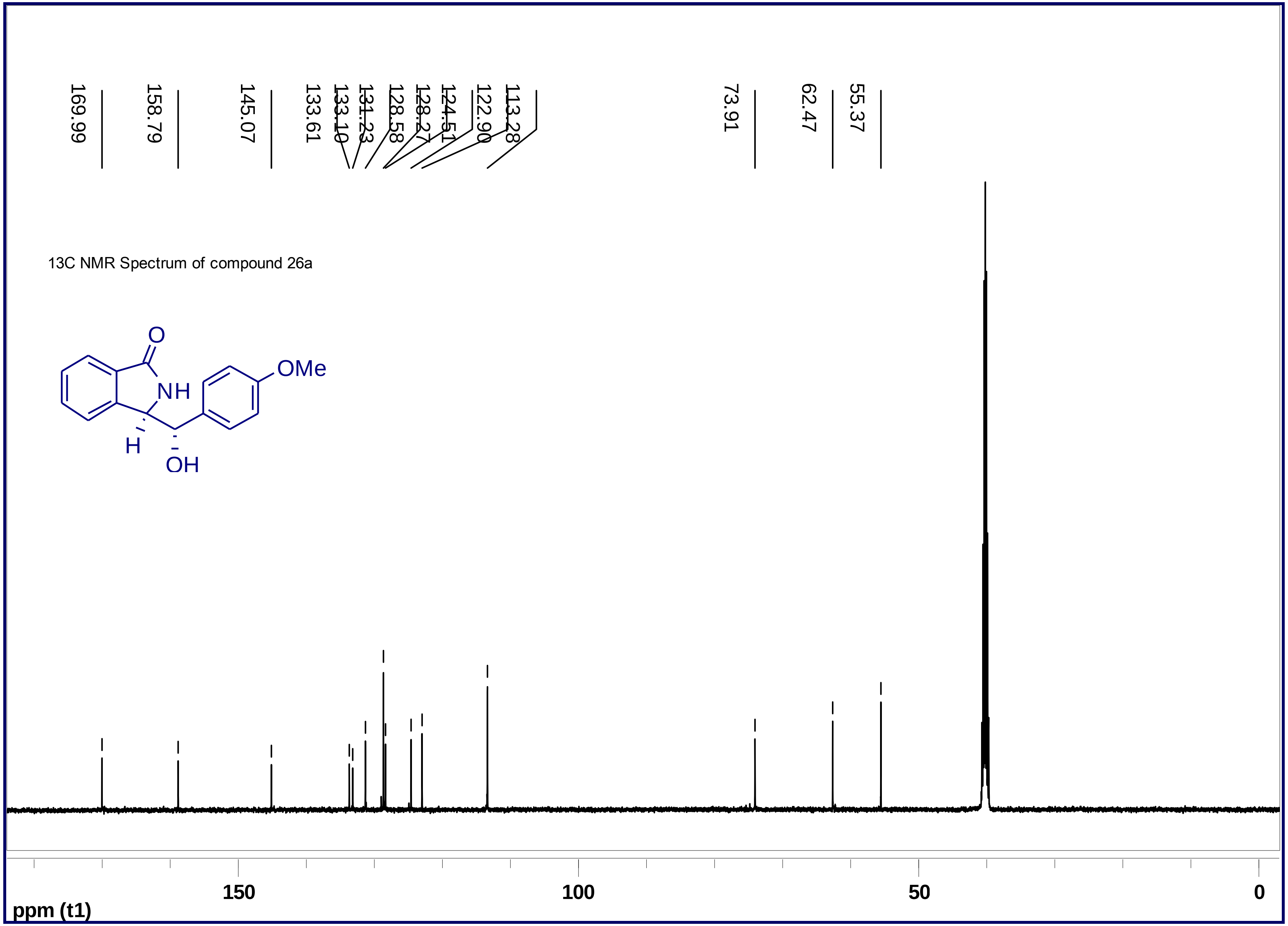

169.99
158.79
145.07
133.61
133.10
131.23
128.58
128.27
124.51
122.90
113.28
73.91
62.47
55.37
13C NMR Spectrum of compound 26a
O
NH
OMe
H
OH
150
100
50
0
ppm (t1)

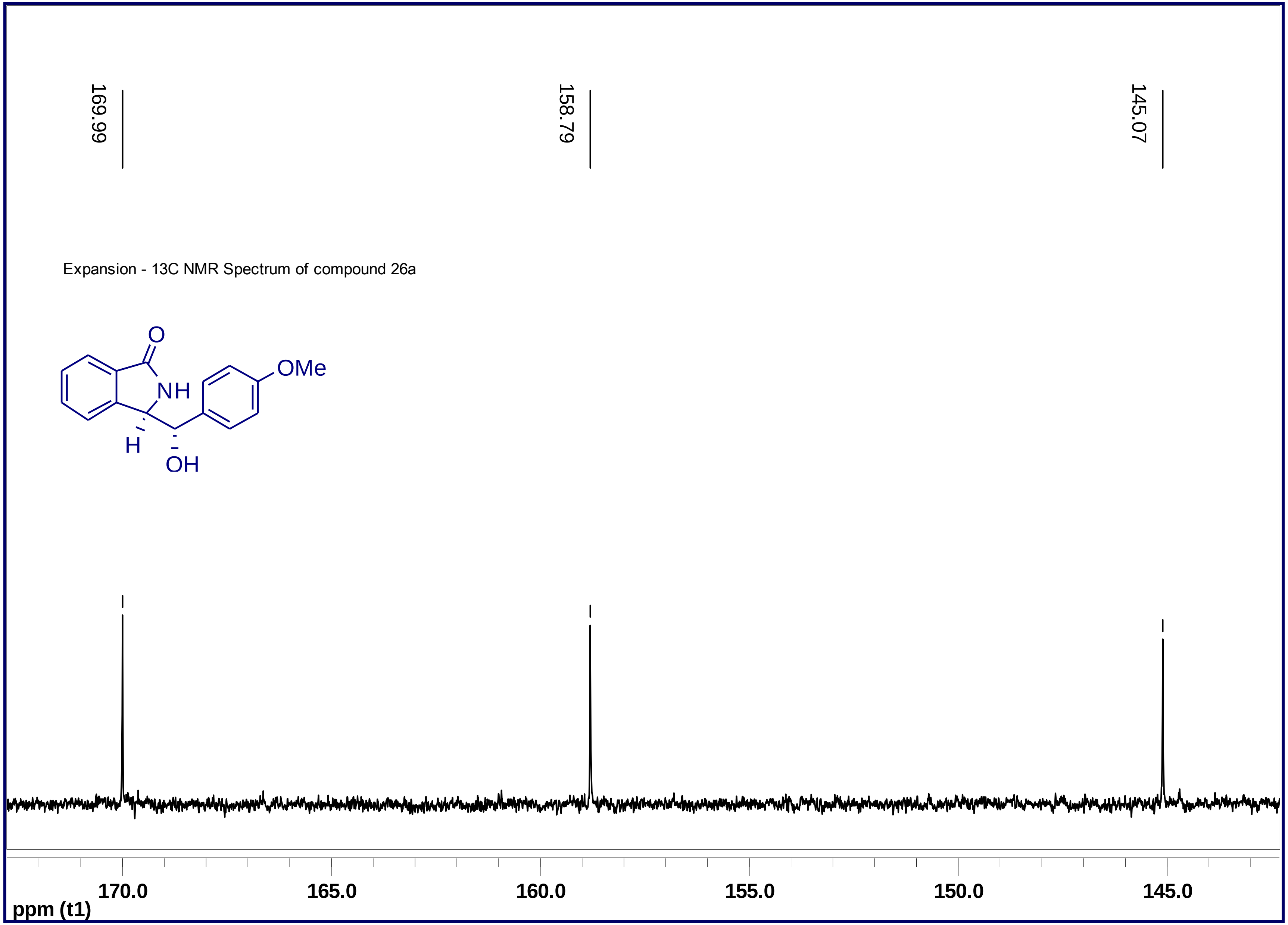
169.99
158.79
145.07
Expansion - 13C NMR Spectrum of compound 26a
O
NH
OMe
H
OH
170.0
165.0
160.0
155.0
150.0
145.0
ppm (t1)

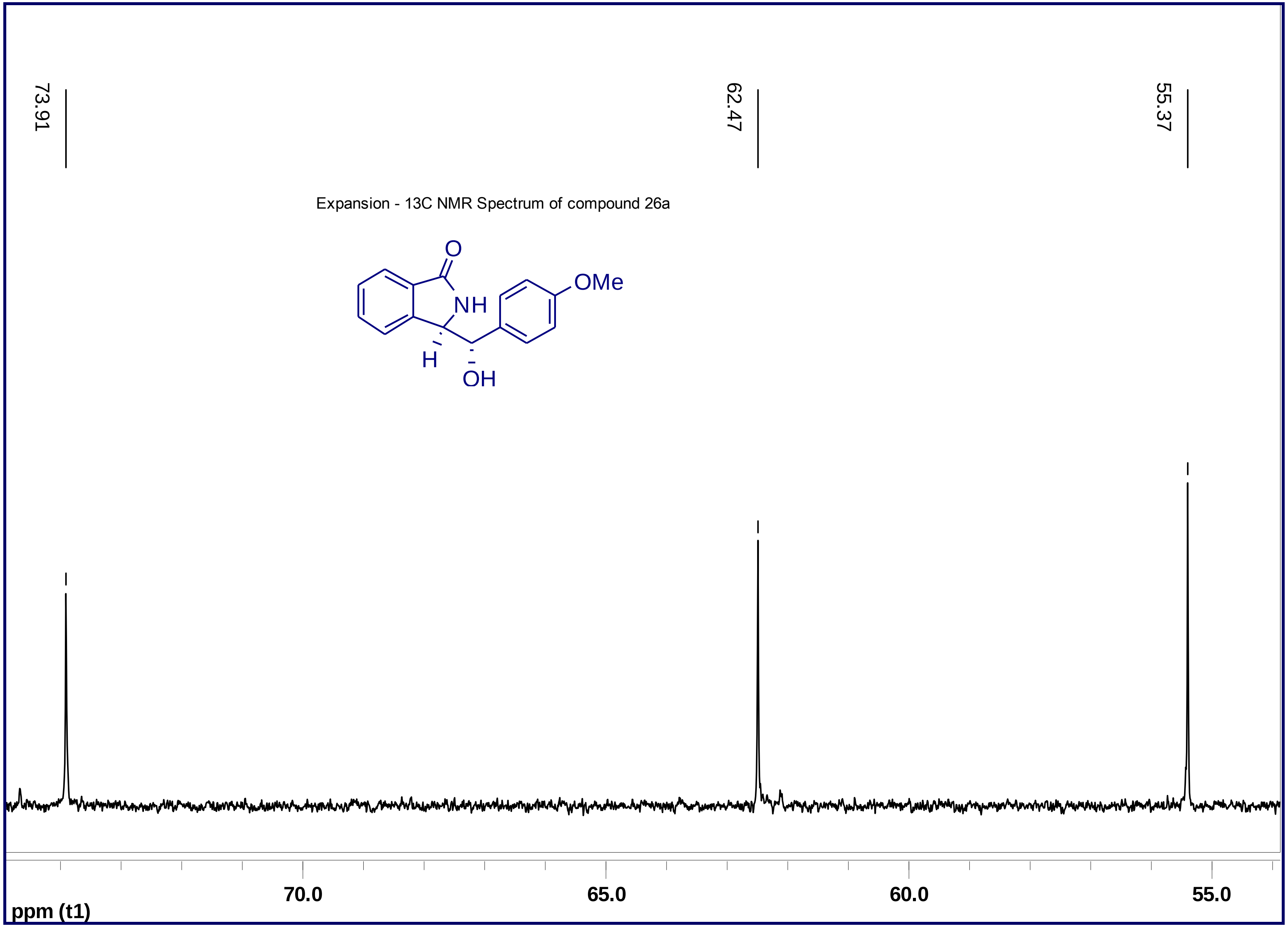

73.91
62.47
55.37
Expansion - 13C NMR Spectrum of compound 26a
O
NH
OMe
H
OH
70.0
65.0
60.0
55.0
ppm (t1)

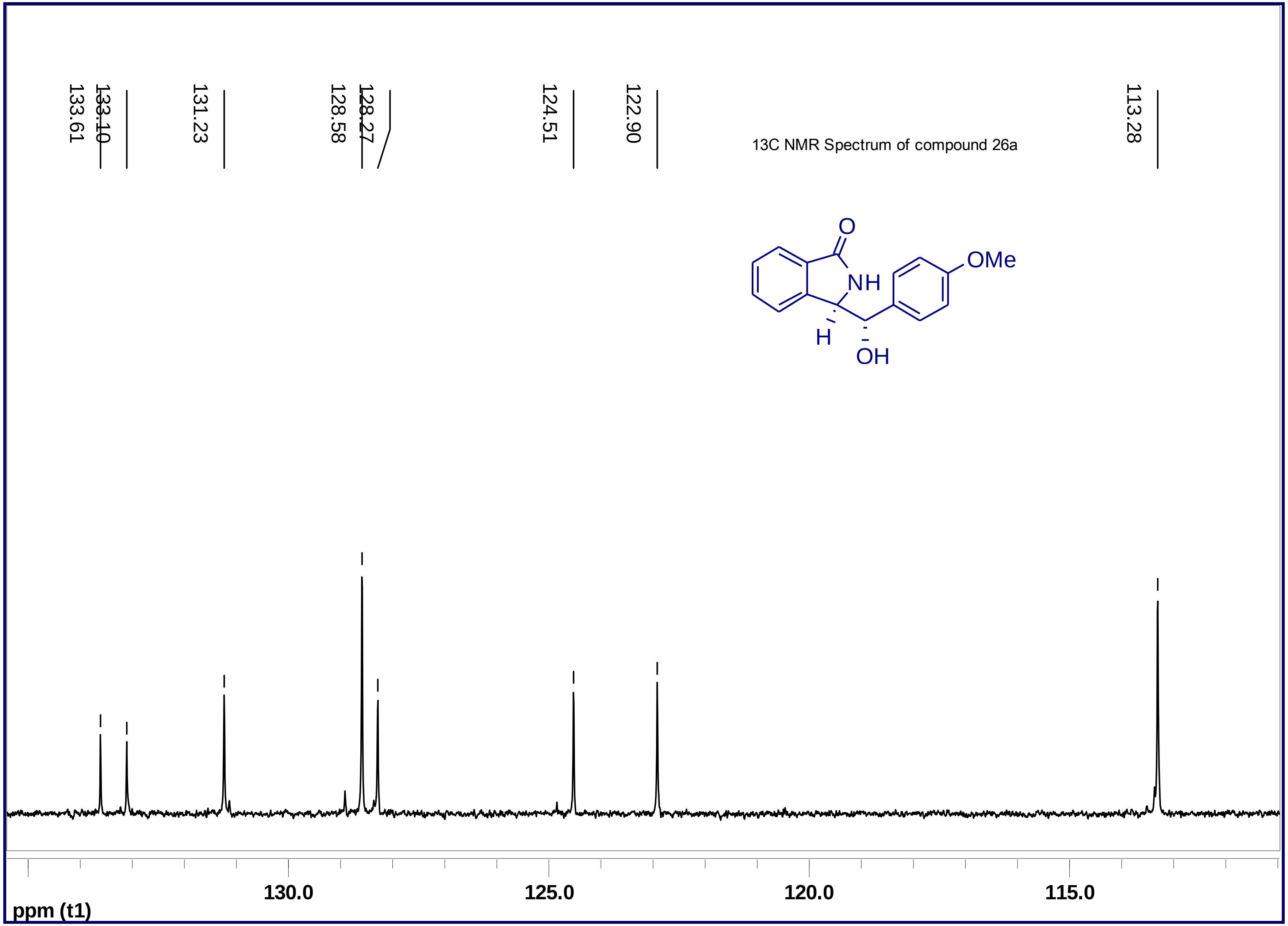

133.61
133.10
131.23
128.58
128.27
124.51
122.90
113.28
13C NMR Spectrum of compound 26a
O
NH
OMe
H
OH
130.0
125.0
120.0
115.0
ppm (t1)

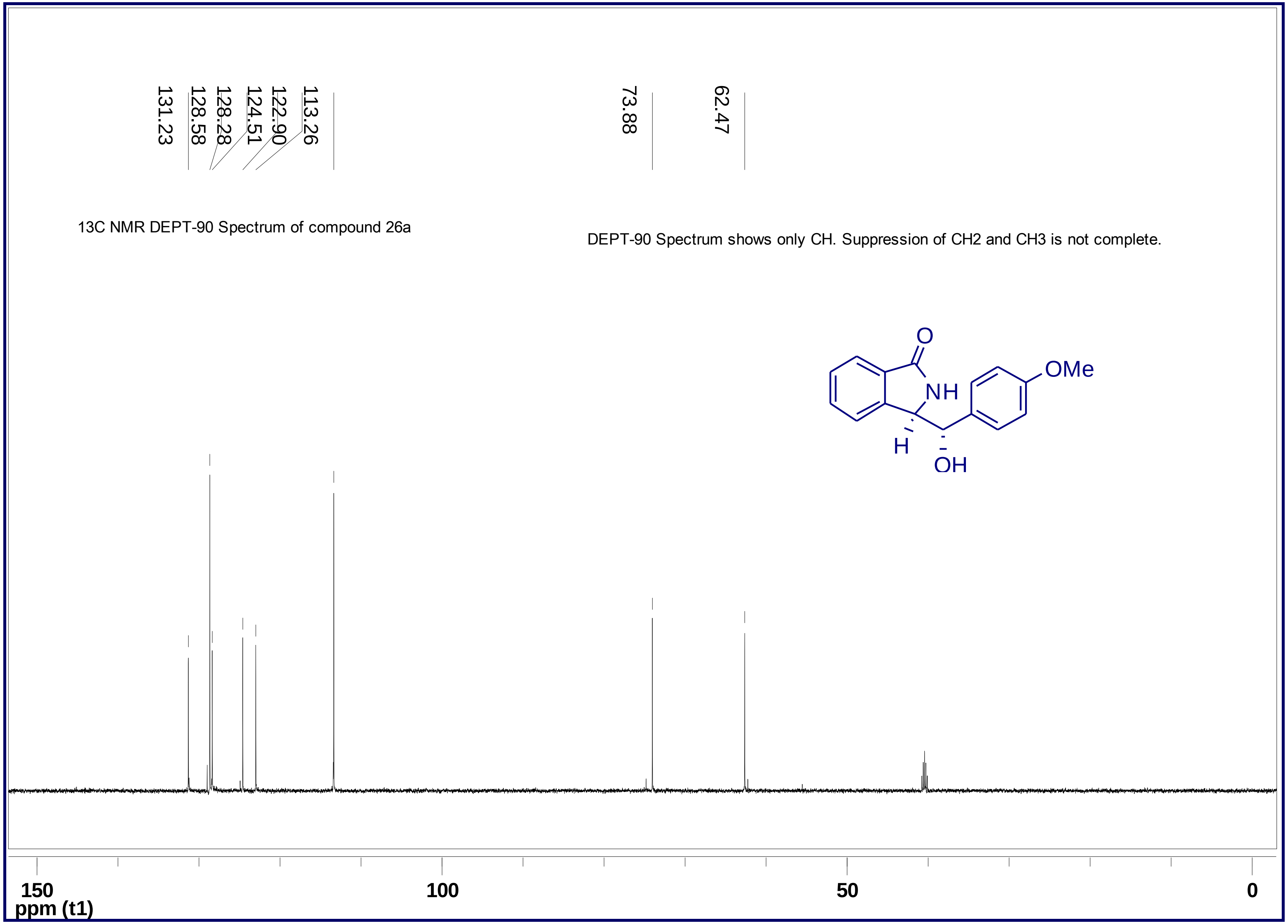
131.23
128.58
128.28
124.51
122.90
113.26
73.88
62.47
13C NMR DEPT-90 Spectrum of compound 26a
DEPT-90 Spectrum shows only CH. Suppression of CH2 and CH3 is not complete.
O
NH
OMe
H
OH
150
100
50
0
ppm (t1)

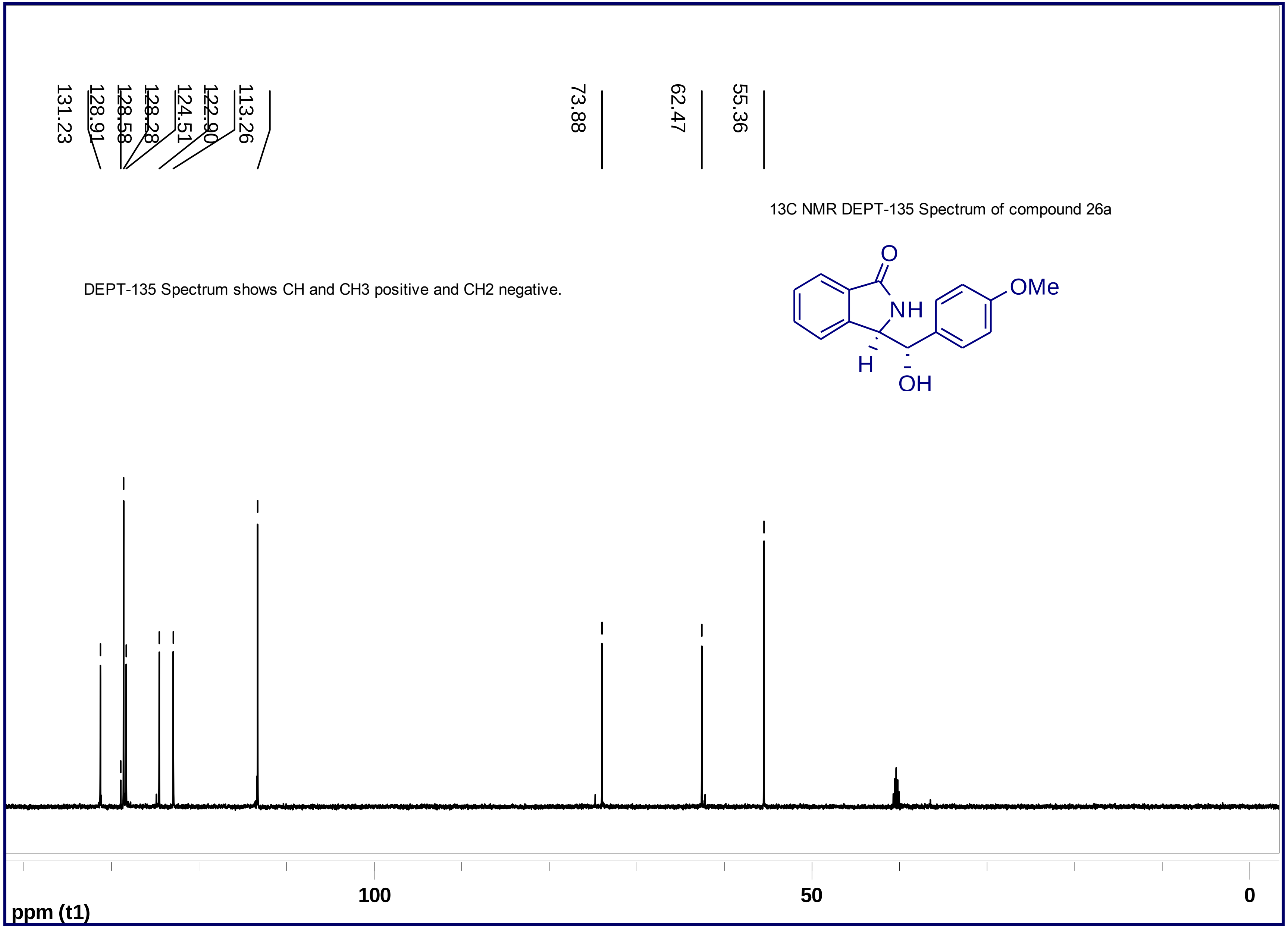
131.23
128.91
128.58
128.28
124.51
122.90
113.26
73.88
62.47
55.36
13C NMR DEPT-135 Spectrum of compound 26a
DEPT-135 Spectrum shows CH and CH3 positive and CH2 negative.
O
NH
OMe
H
OH
100
50
0
ppm (t1)

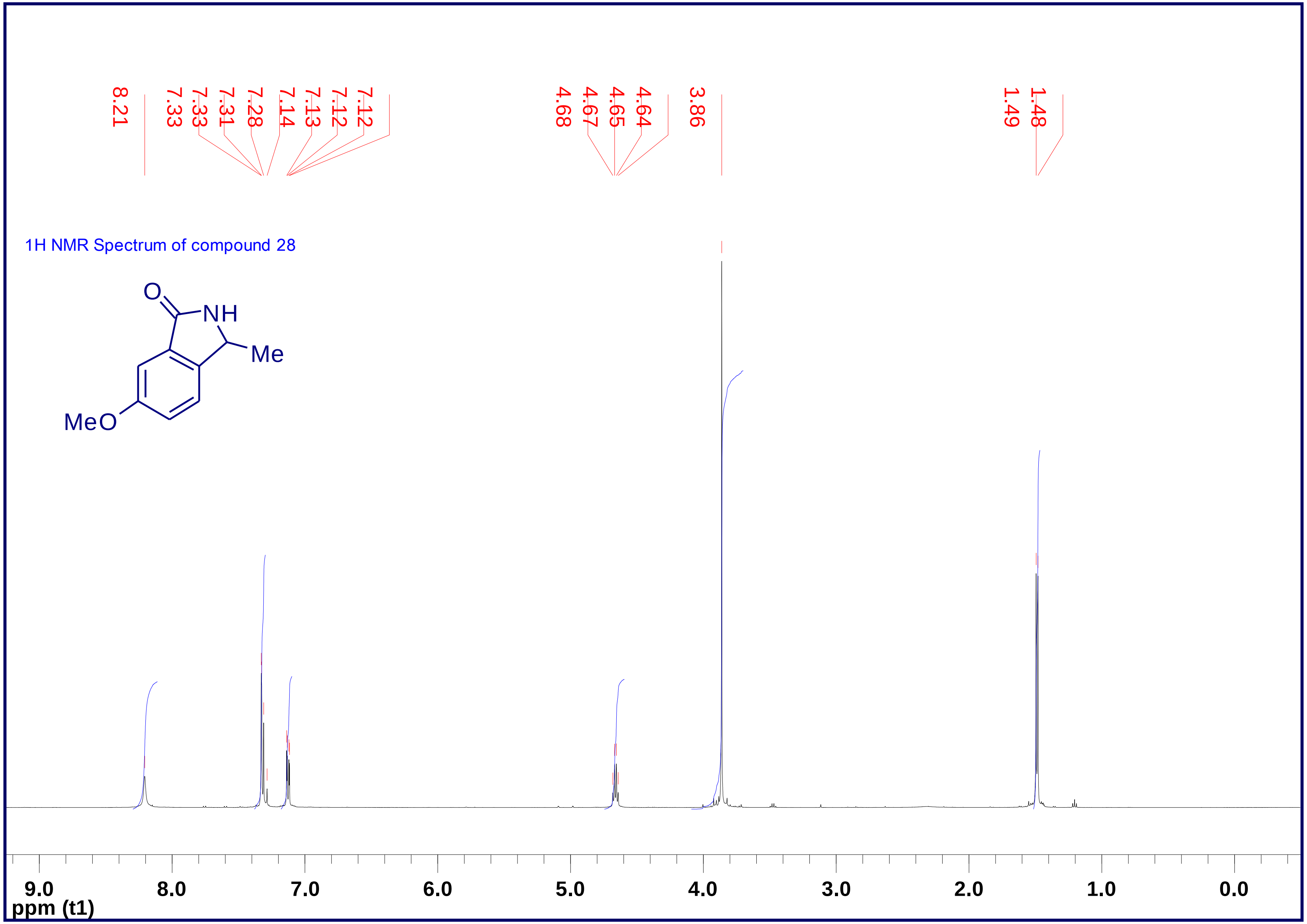
8.21
7.33
7.33
7.31
7.28
7.14
7.13
7.12
7.12
4.68
4.67
4.65
4.64
3.86
1.49
1.48
1H NMR Spectrum of compound 28
O
NH
Me
MeO
9.0
8.0
7.0
6.0
5.0
4.0
3.0
2.0
1.0
0.0
ppm (t1)

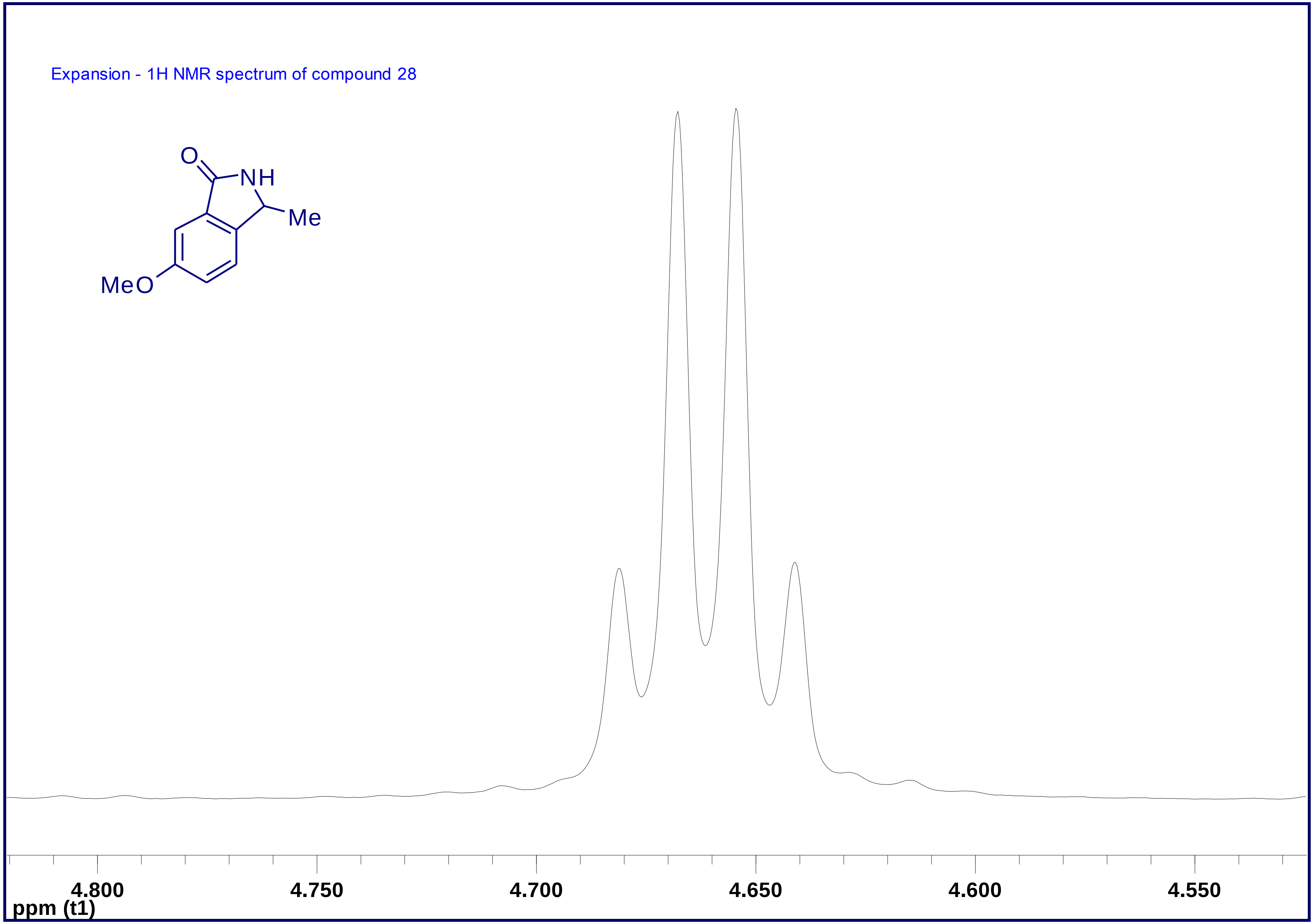
Expansion - 1H NMR spectrum of compound 28
O
NH
Me
MeO
4.800
4.750
4.700
4.650
4.600
4.550
ppm (t1)

Expansion - 1H NMR spectrum of compound 28

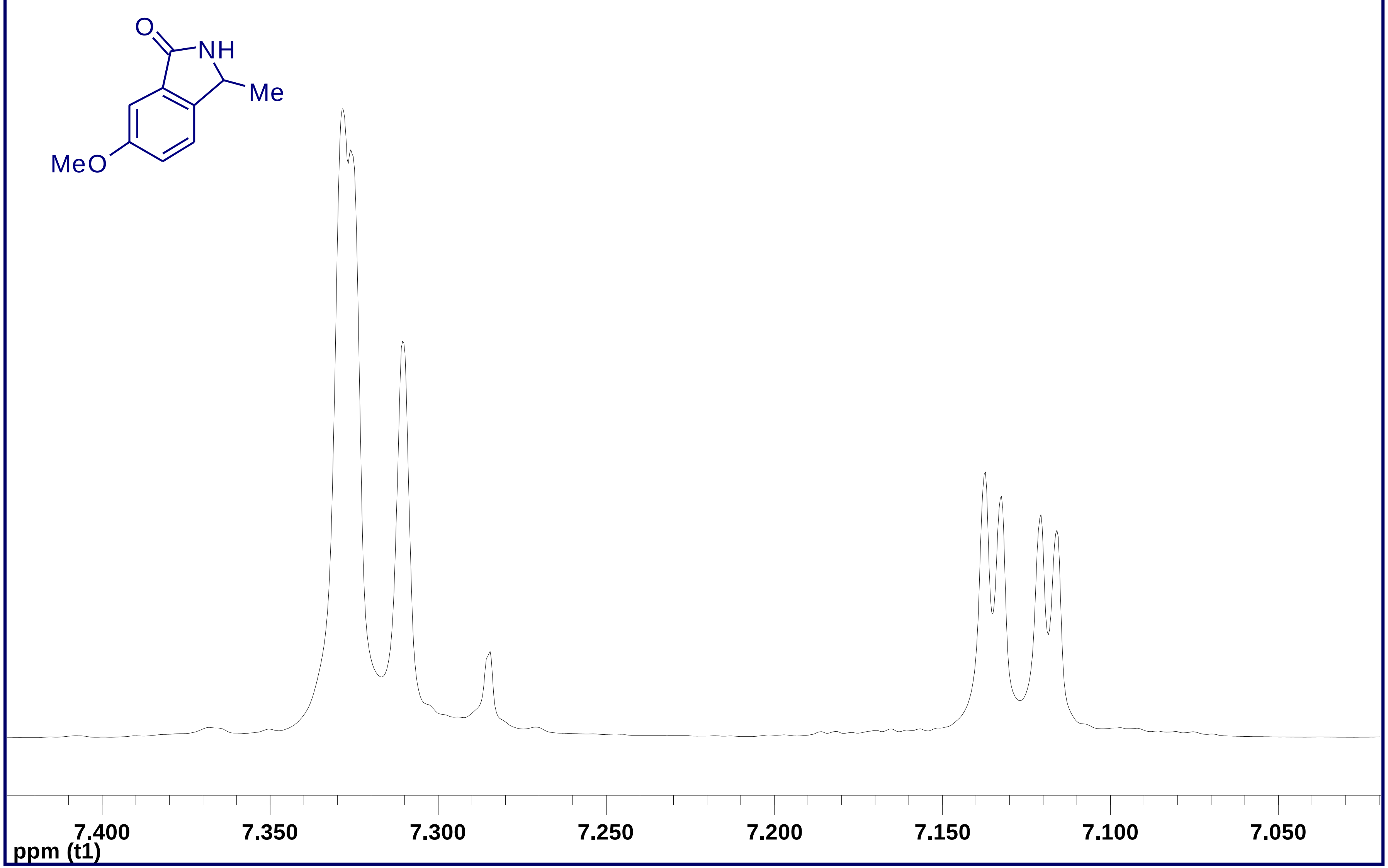

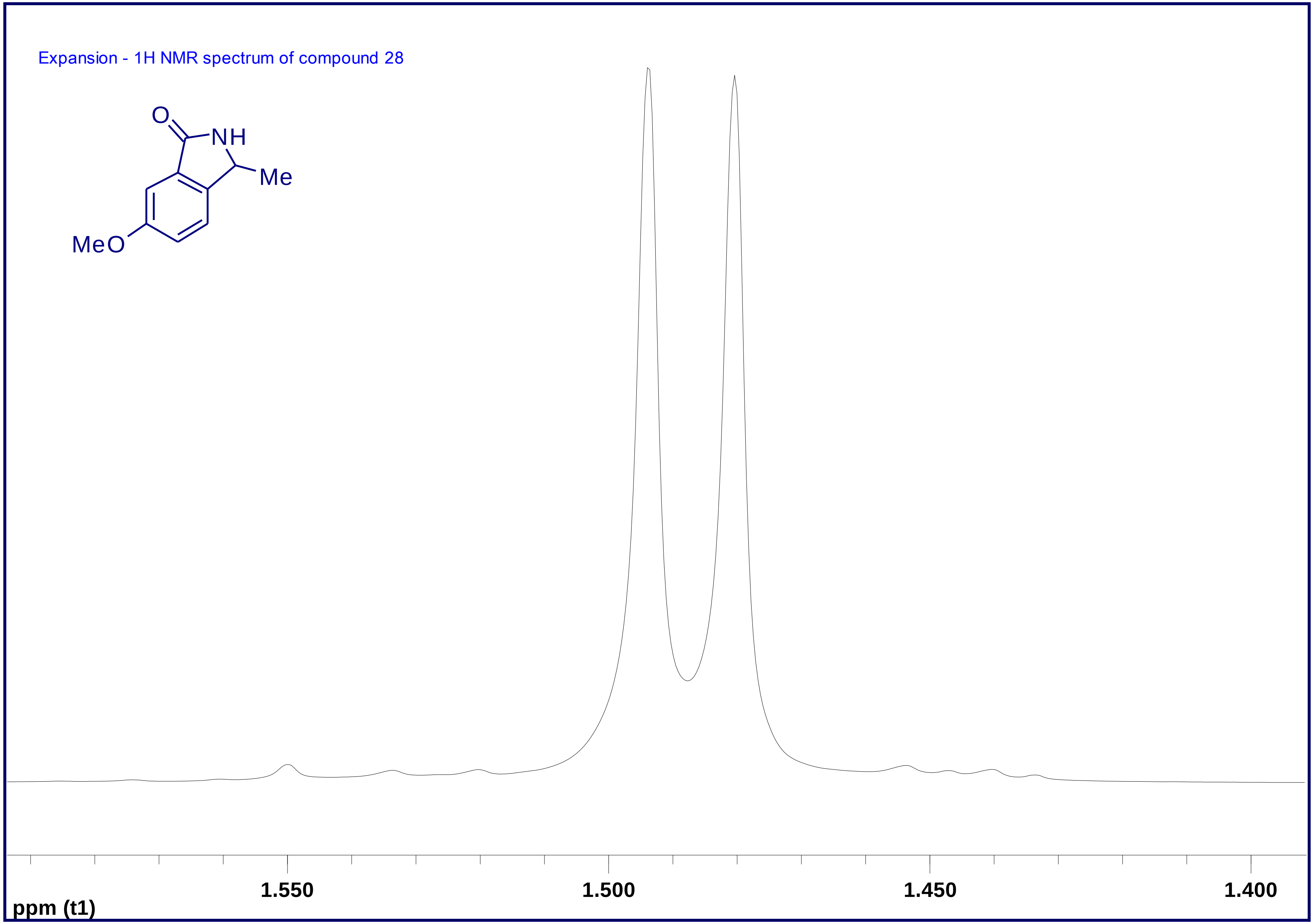

Expansion - 1H NMR spectrum of compound 28
O
NH
Me
MeO
1.550
1.500
1.450
1.400
ppm (t1)

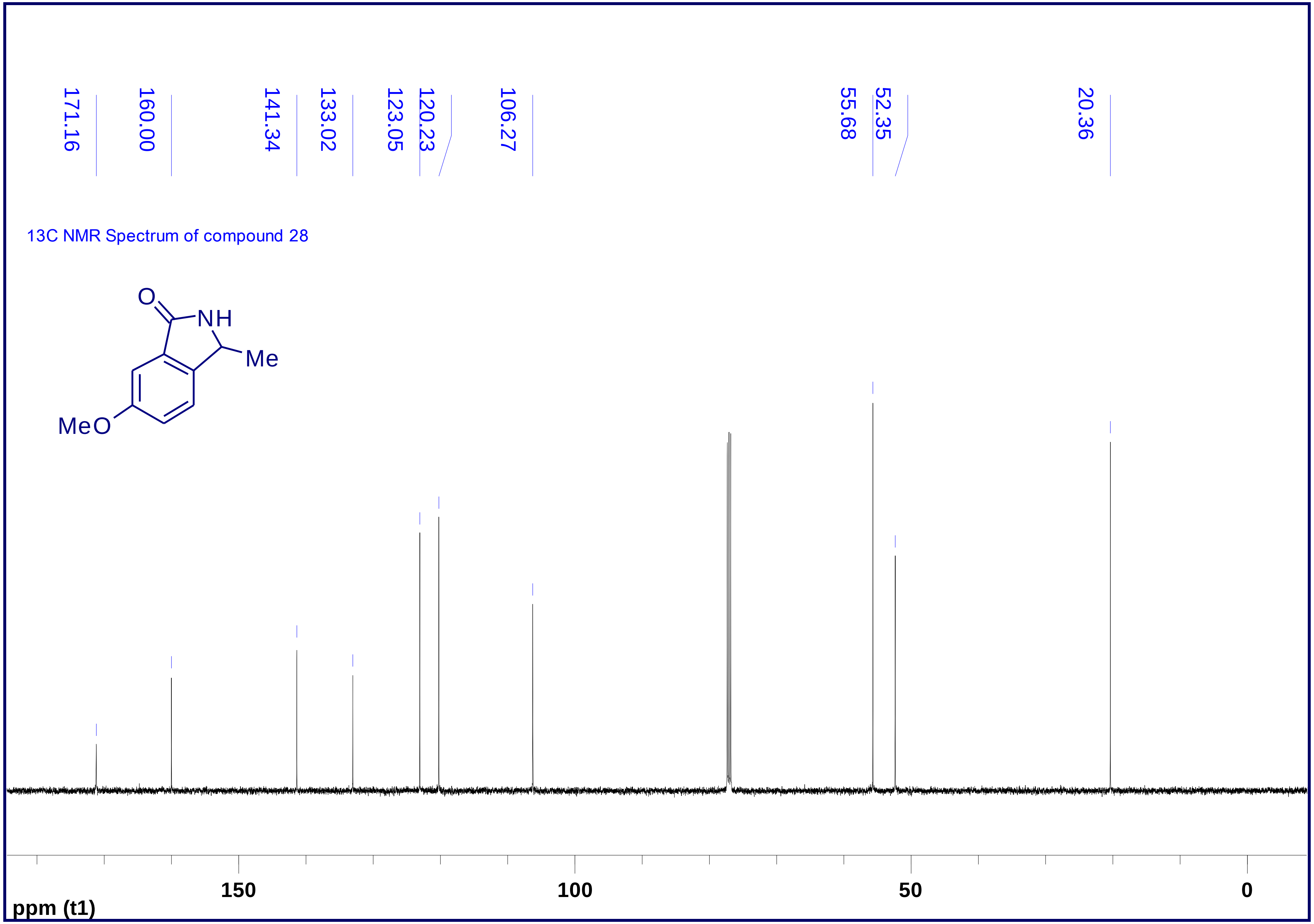

171.16
160.00
141.34
133.02
123.05
120.23
106.27
55.68
52.35
20.36
13C NMR Spectrum of compound 28
O
NH
Me
MeO
150
100
50
0
ppm (t1)

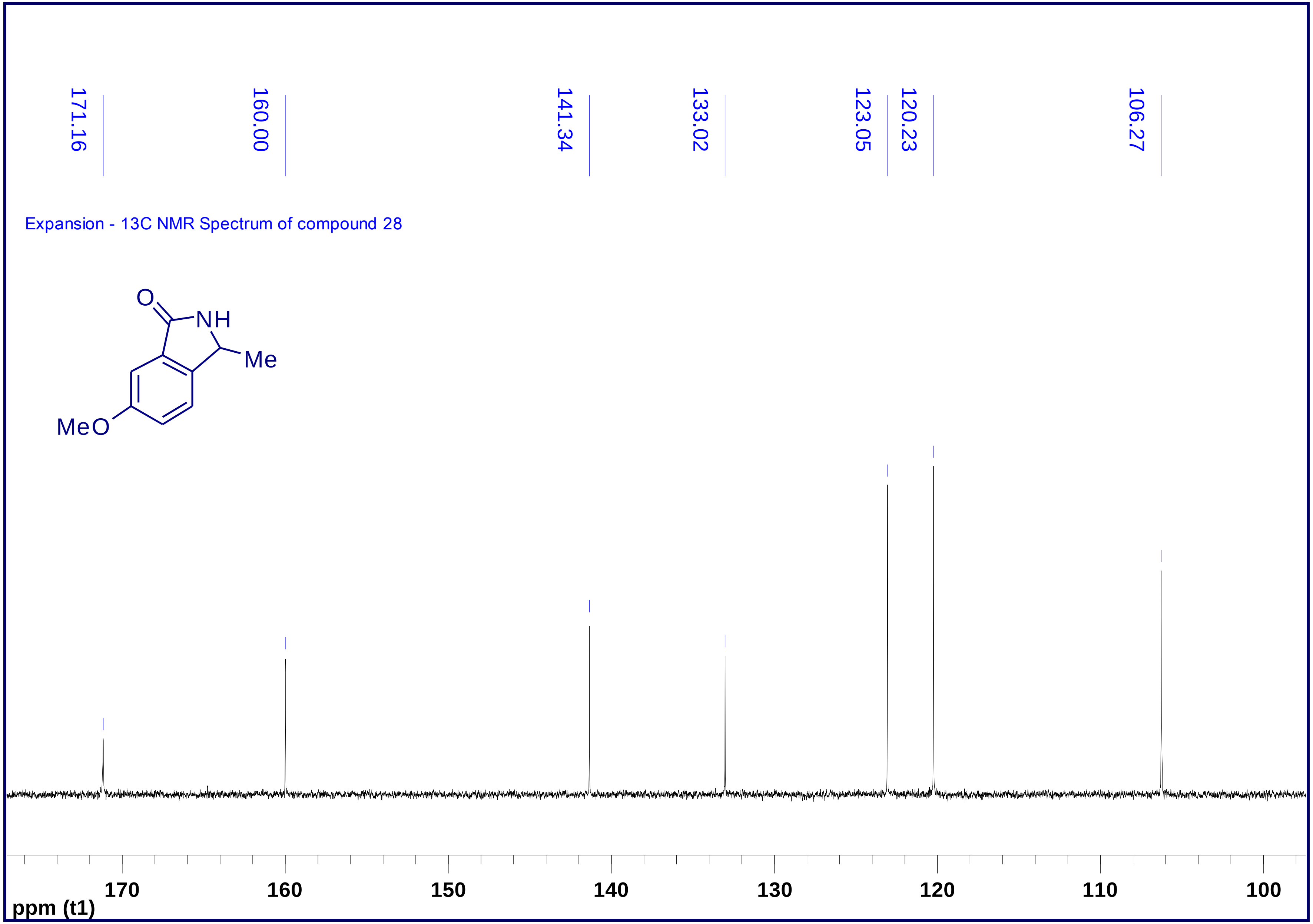
171.16
160.00
141.34
133.02
123.05
120.23
106.27
Expansion - 13C NMR Spectrum of compound 28
O
NH
Me
MeO
170
160
150
140
130
120
110
100
ppm (t1)

13C NMR DEPT-90 Spectrum of compound 28

DEPT-90 Spectrum shows only CH carbons. Suppression of CH2 and CH3 is not complete.

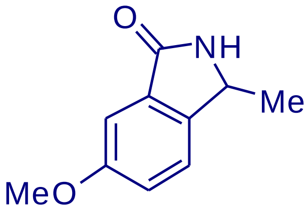

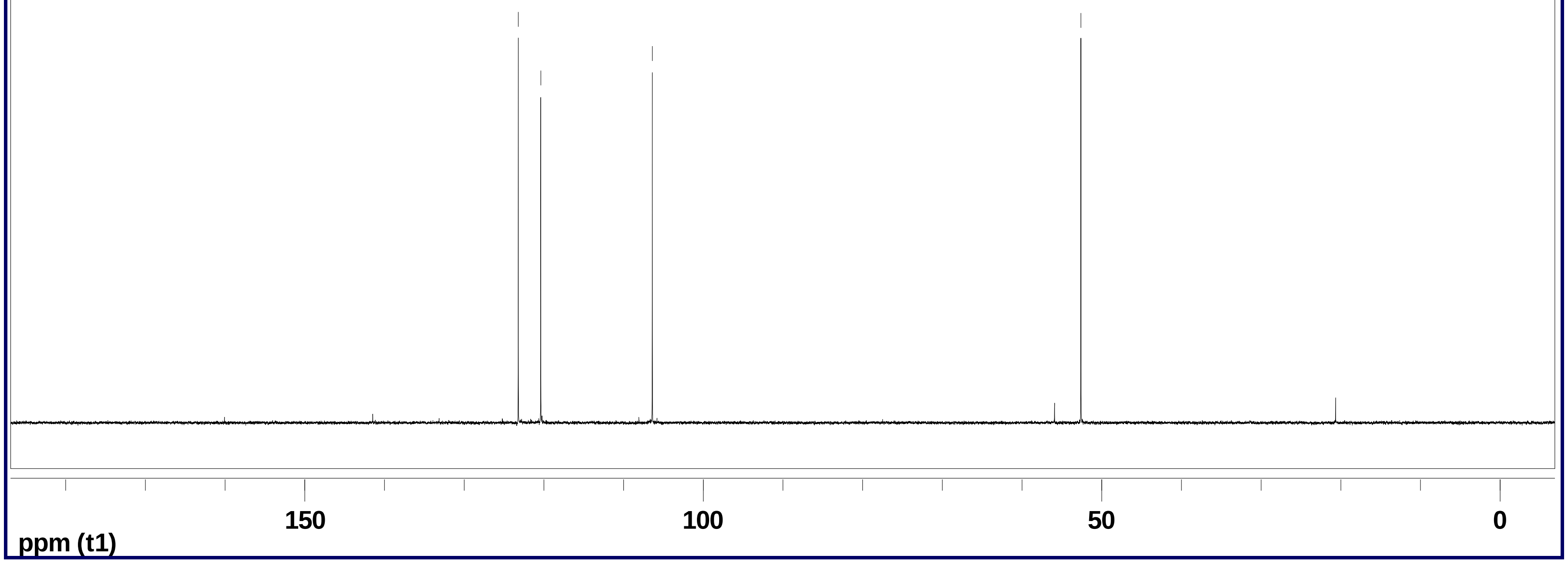

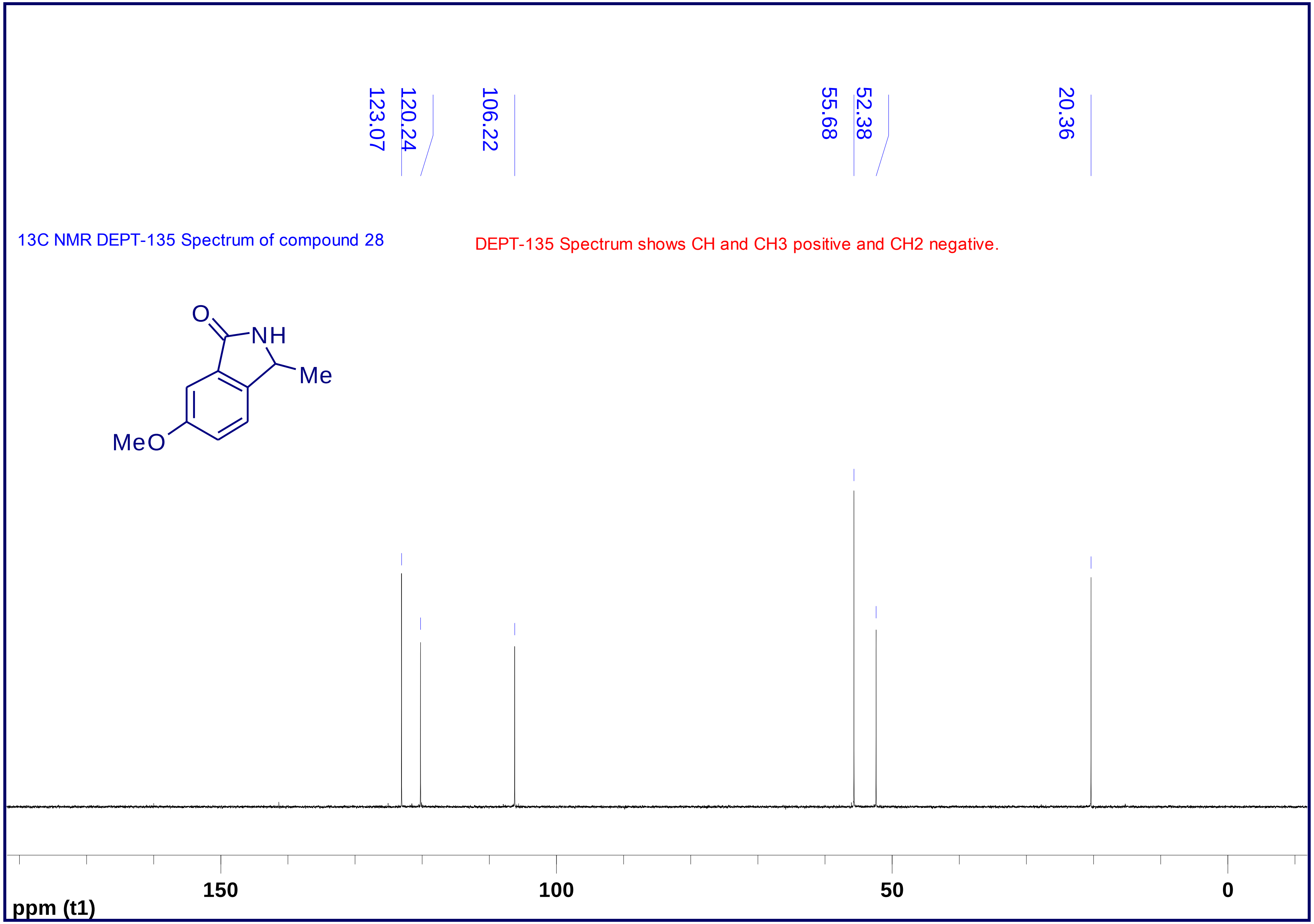
123.07
120.24
106.22
55.68
52.38
20.36
13C NMR DEPT-135 Spectrum of compound 28
DEPT-135 Spectrum shows CH and CH3 positive and CH2 negative.
O
NH
Me
MeO
150
100
50
0
ppm (t1)

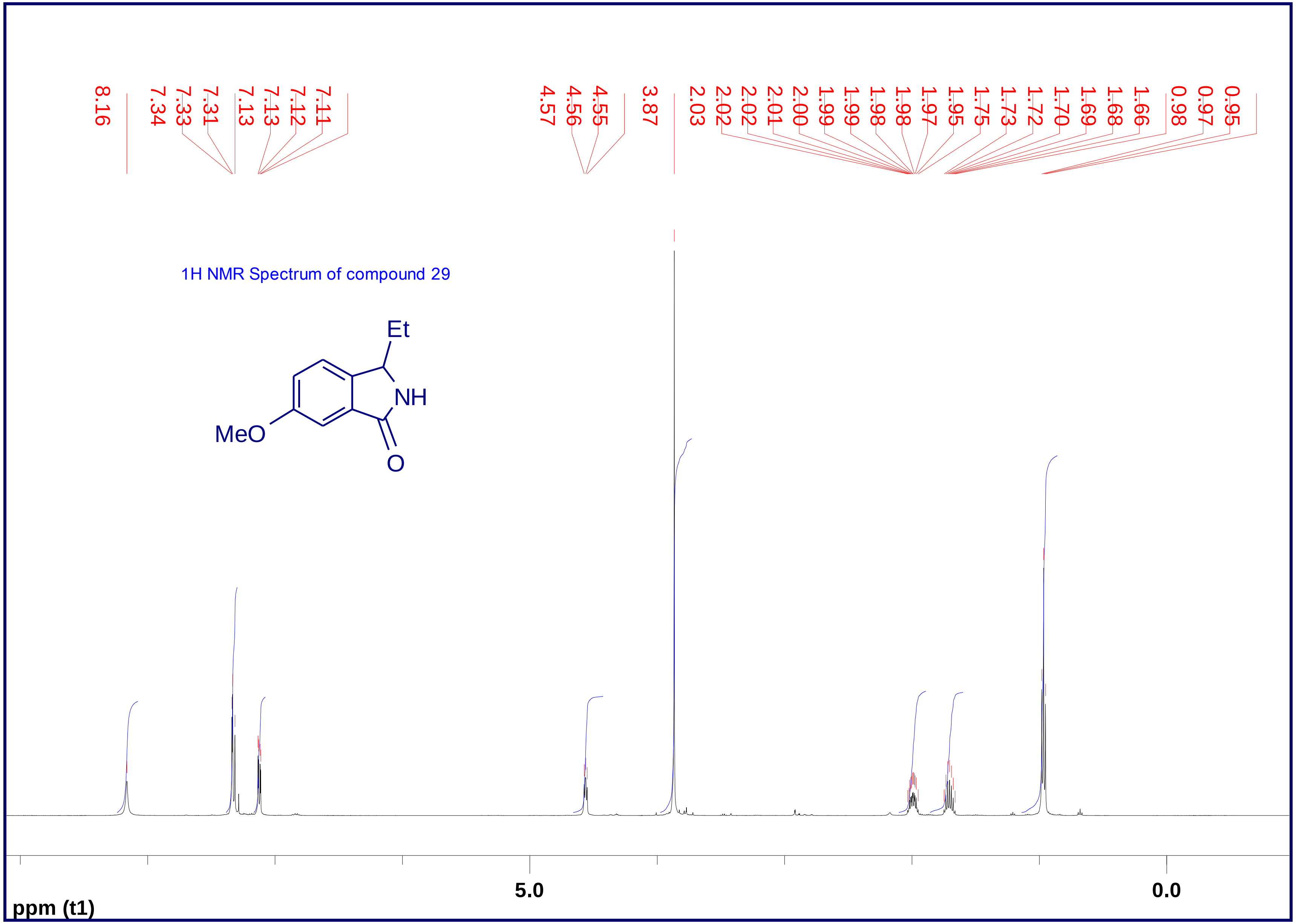
8.16
7.34
7.33
7.31
7.13
7.13
7.12
7.11
4.57
4.56
4.55
3.87
2.03
2.02
2.02
2.01
2.00
1.99
1.99
1.98
1.98
1.97
1.95
1.75
1.73
1.72
1.70
1.69
1.68
1.66
0.98
0.97
0.95
1H NMR Spectrum of compound 29
Et
NH
MeO
O
5.0
0.0
ppm (t1)

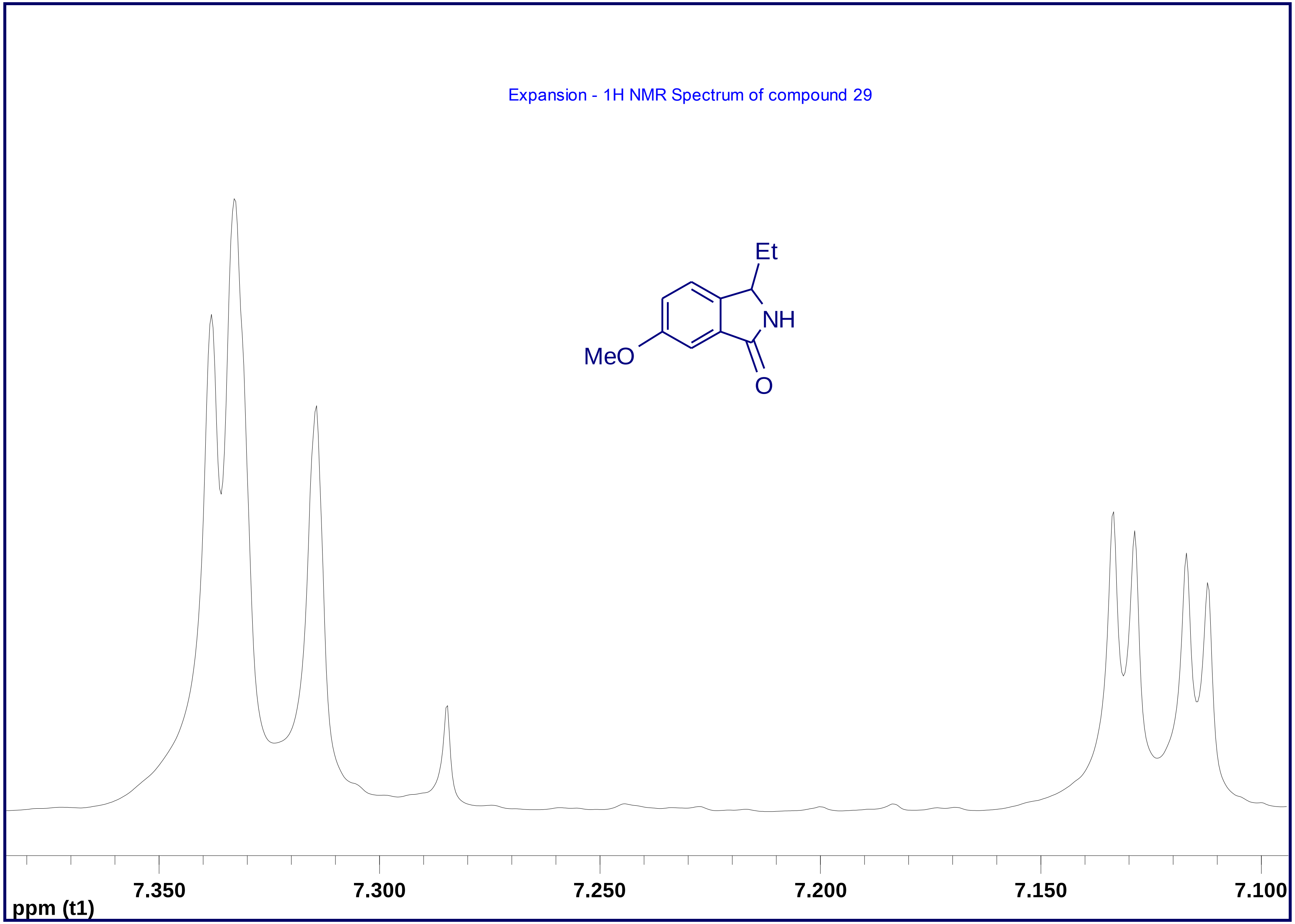
Expansion - 1H NMR Spectrum of compound 29
Et
NH
MeO
O
7.350
7.300
7.250
7.200
7.150
7.100
ppm (t1)

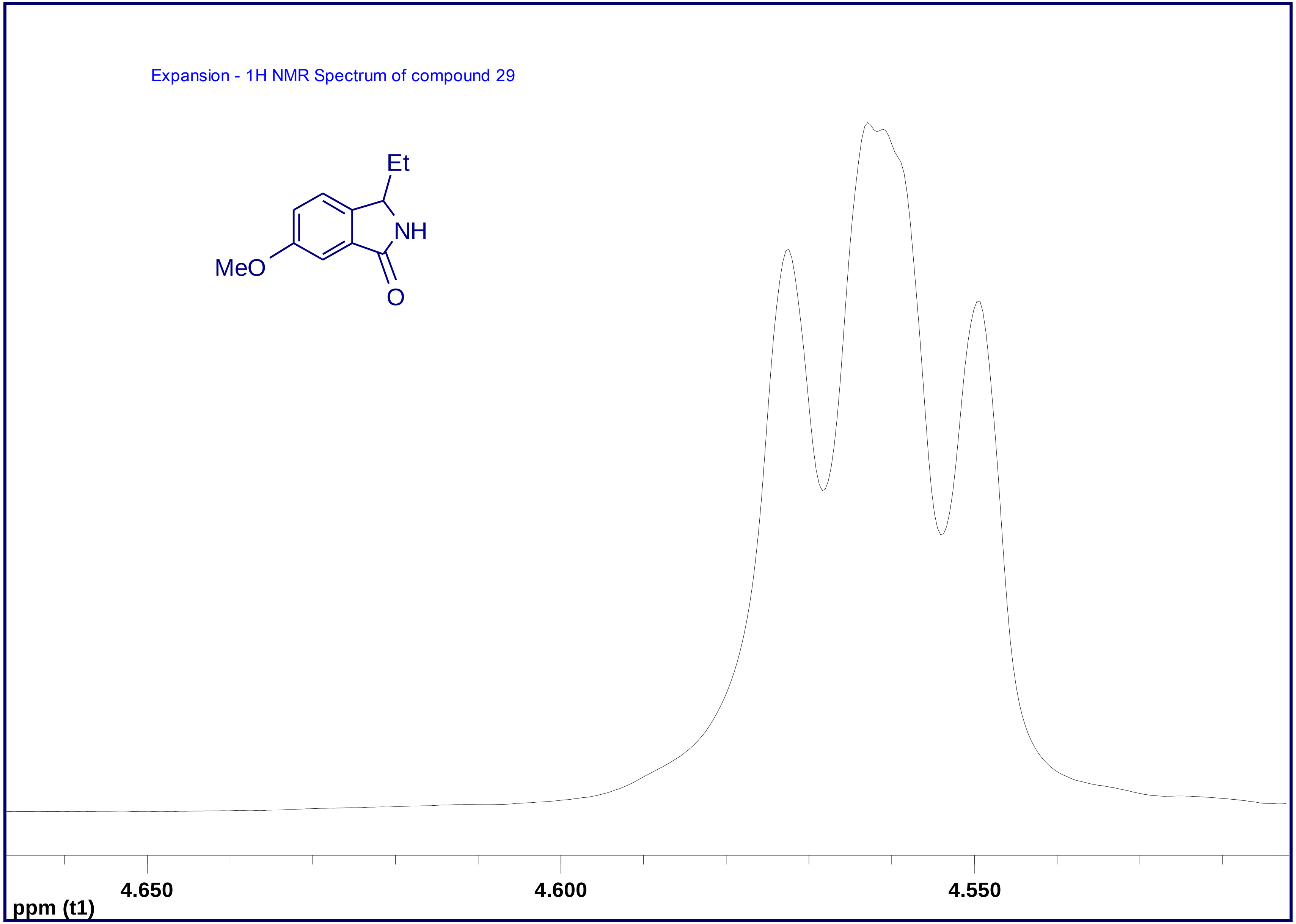
Expansion - 1H NMR Spectrum of compound 29
Et
NH
MeO
O
4.650
4.600
4.550
ppm (t1)

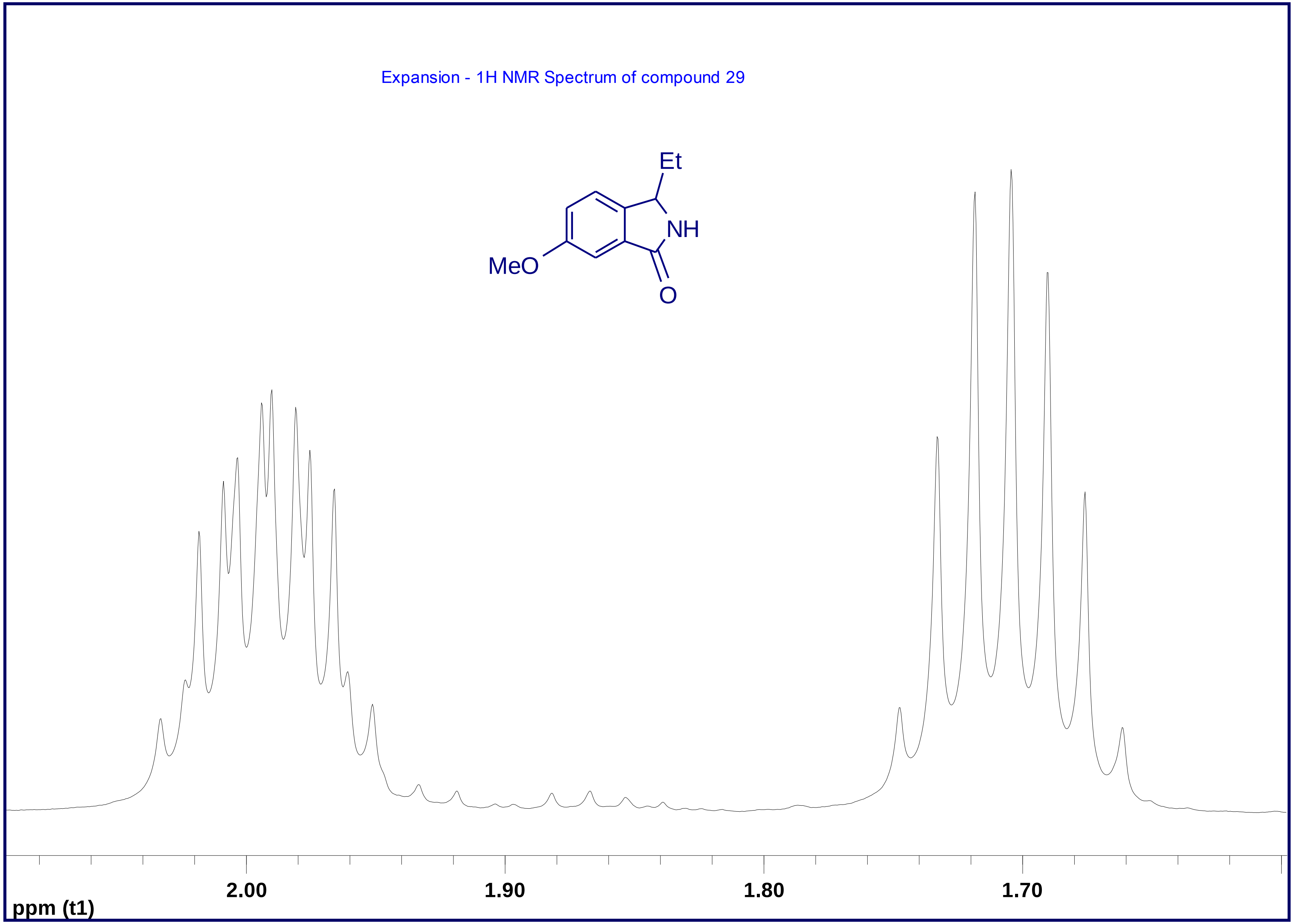

Expansion - 1H NMR Spectrum of compound 29
Et
NH
MeO
O
2.00
1.90
1.80
1.70
ppm (t1)

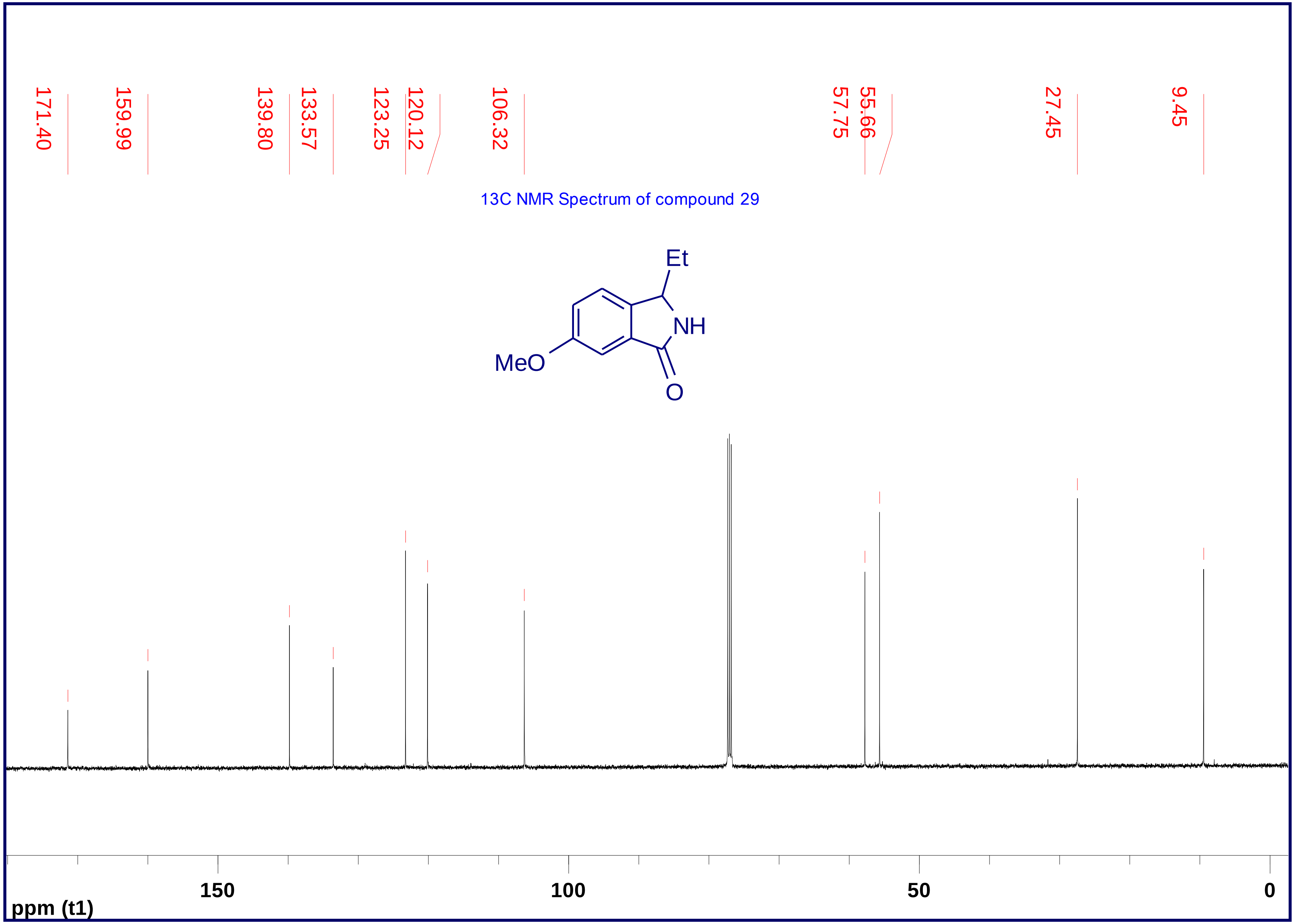

171.40
159.99
139.80
133.57
123.25
120.12
106.32
57.75
55.66
27.45
9.45
13C NMR Spectrum of compound 29
Et
NH
MeO
O
150
100
50
0
ppm (t1)

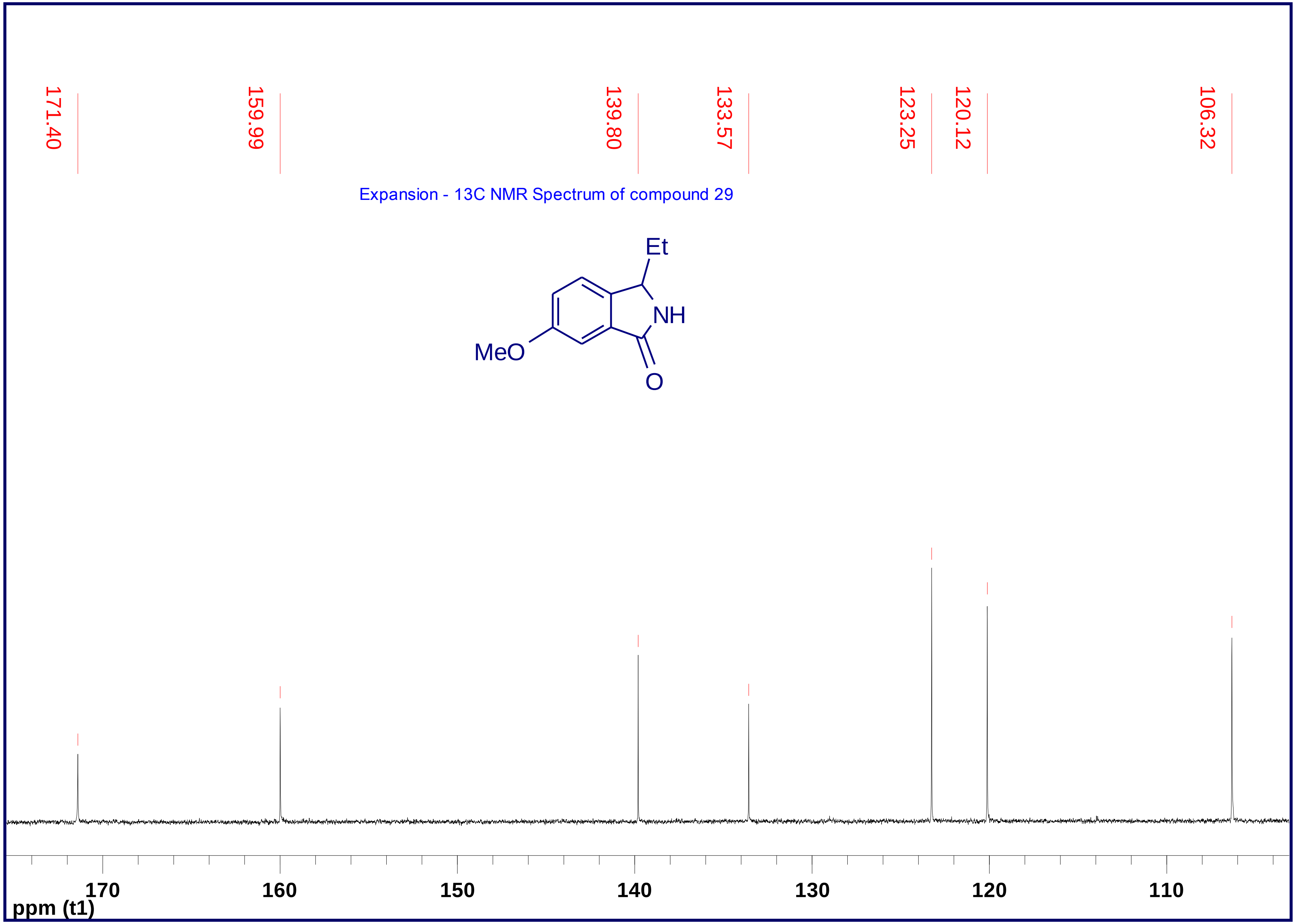
171.40
159.99
139.80
133.57
123.25
120.12
106.32
Expansion - 13C NMR Spectrum of compound 29
Et
NH
MeO
O
170
160
150
140
130
120
110
ppm (t1)

Expansion - 13C NMR Spectrum of compound 29

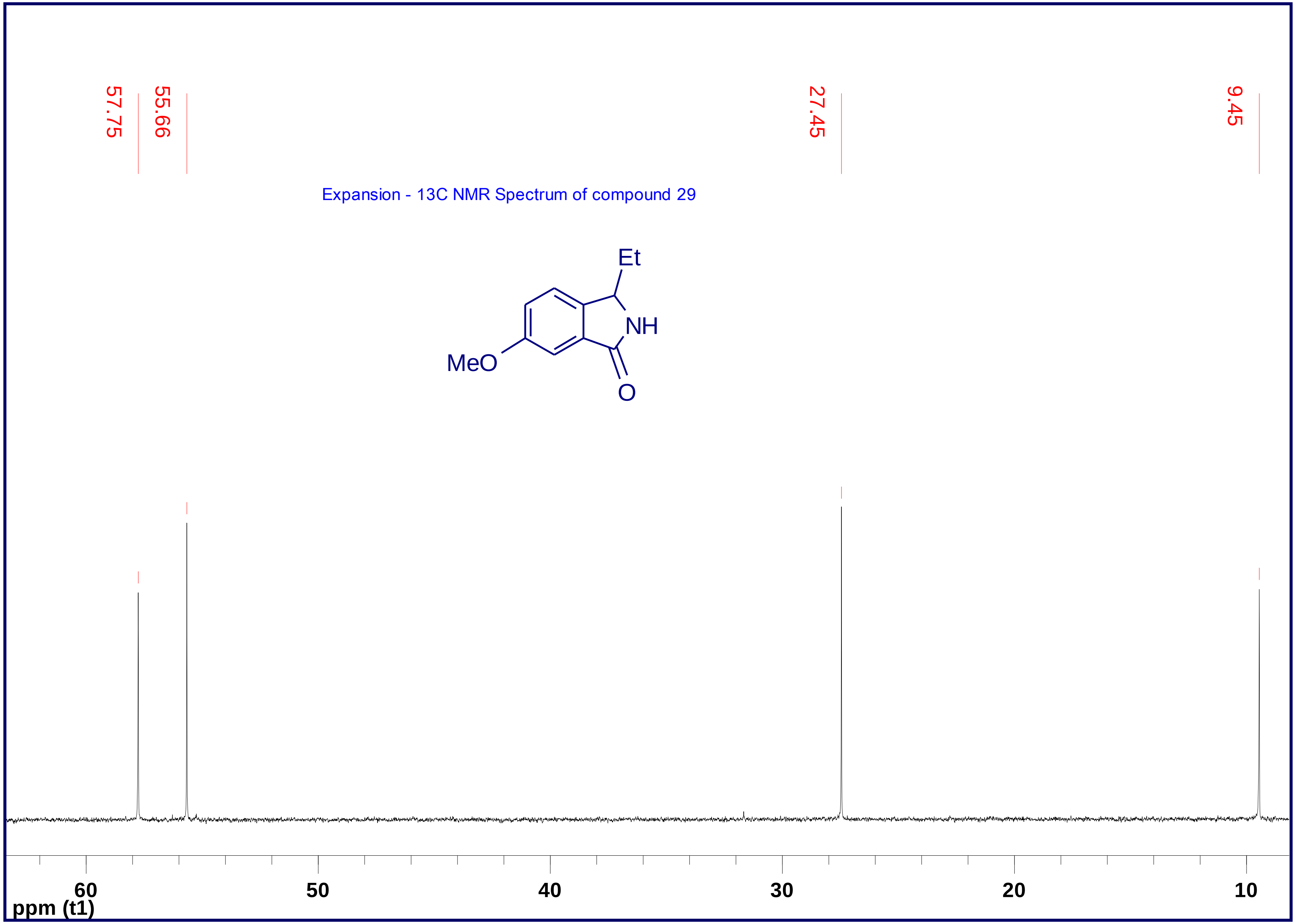

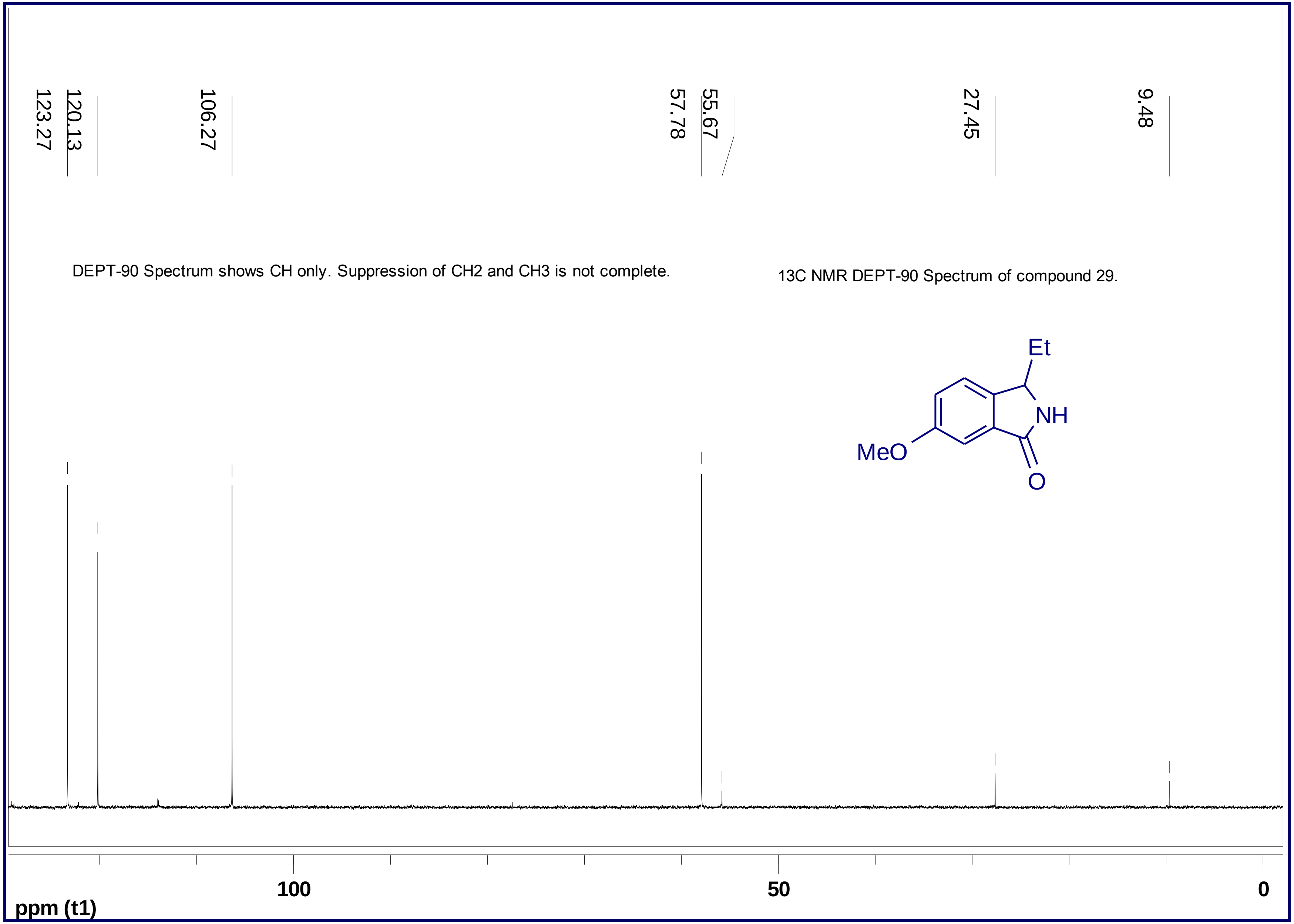
123.27
120.13
106.27
57.78
55.67
27.45
9.48
DEPT-90 Spectrum shows CH only. Suppression of CH2 and CH3 is not complete.
13C NMR DEPT-90 Spectrum of compound 29.
Et
NH
MeO
O
100
50
0
ppm (t1)

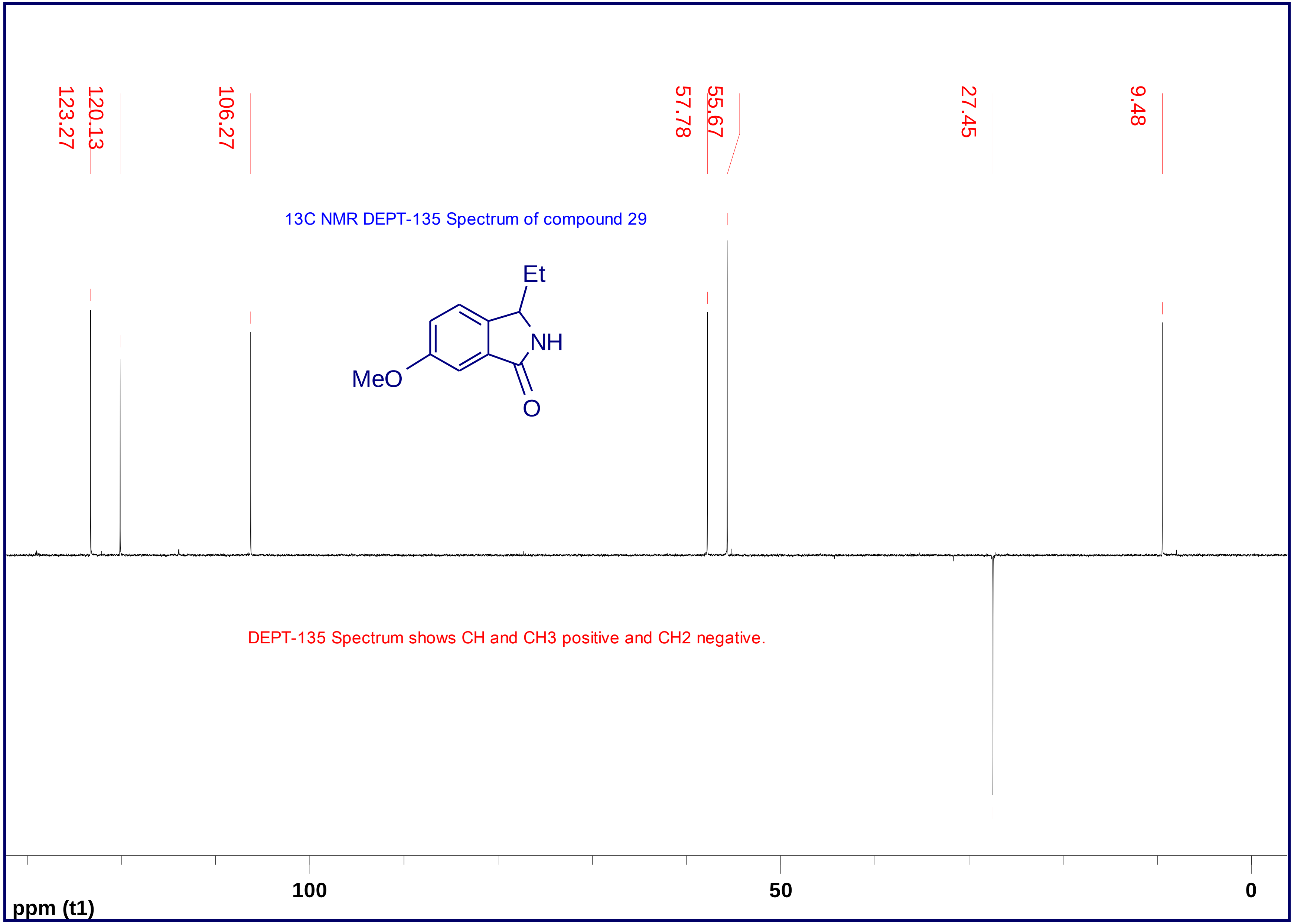
123.27
120.13
106.27
57.78
55.67
27.45
9.48
13C NMR DEPT-135 Spectrum of compound 29
Et
NH
MeO
O
DEPT-135 Spectrum shows CH and CH3 positive and CH2 negative.
100
50
0
ppm (t1)

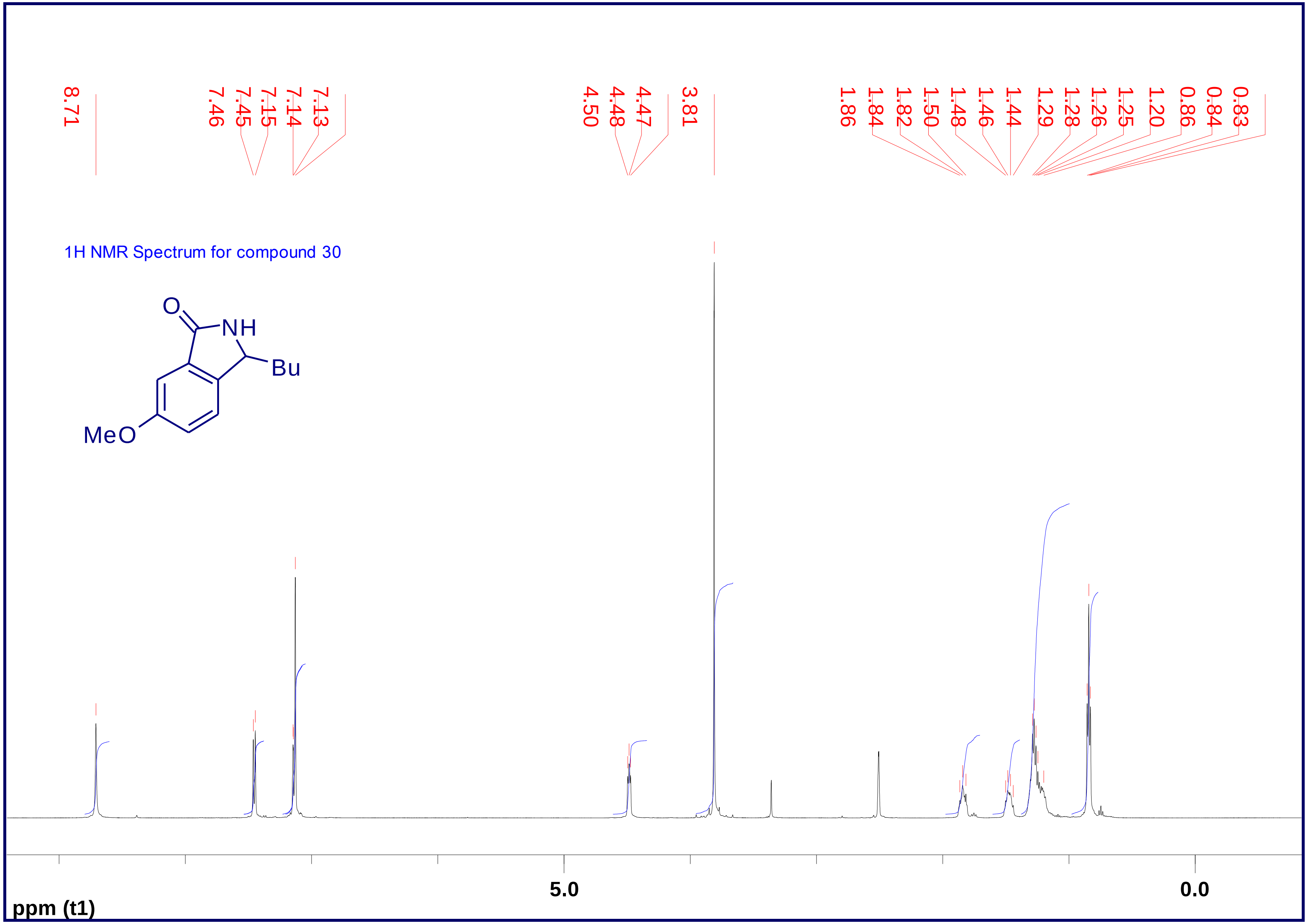
8.71
7.46
7.45
7.15
7.14
7.13
4.50
4.48
4.47
3.81
1.86
1.84
1.82
1.50
1.48
1.46
1.44
1.29
1.28
1.26
1.25
1.20
0.86
0.84
0.83
1H NMR Spectrum for compound 30
O
NH
Bu
MeO
5.0
0.0
ppm (t1)

Expansion - 1H NMR spectrum for compound 30

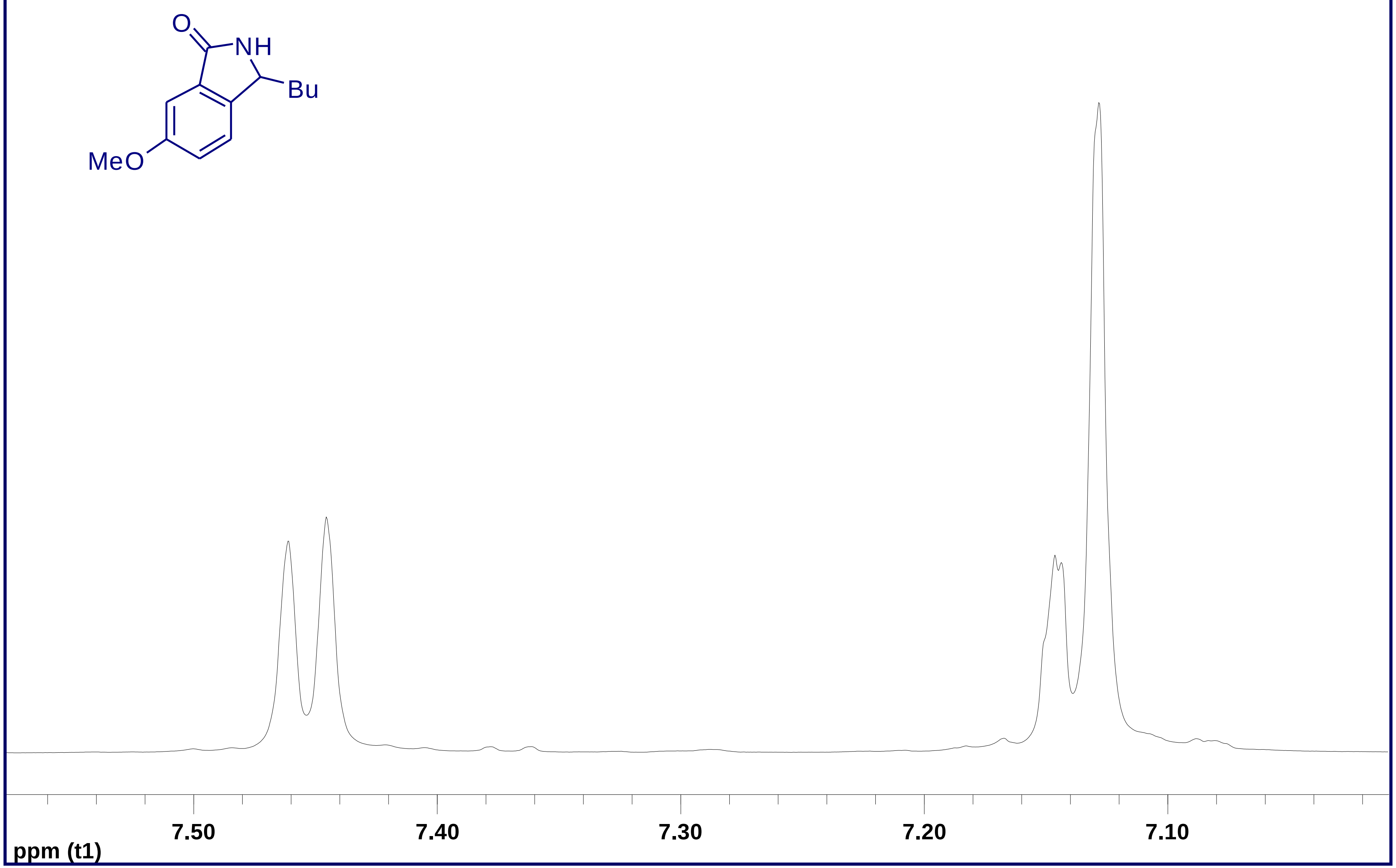

Expansion - 1H NMR spectrum for compound 30

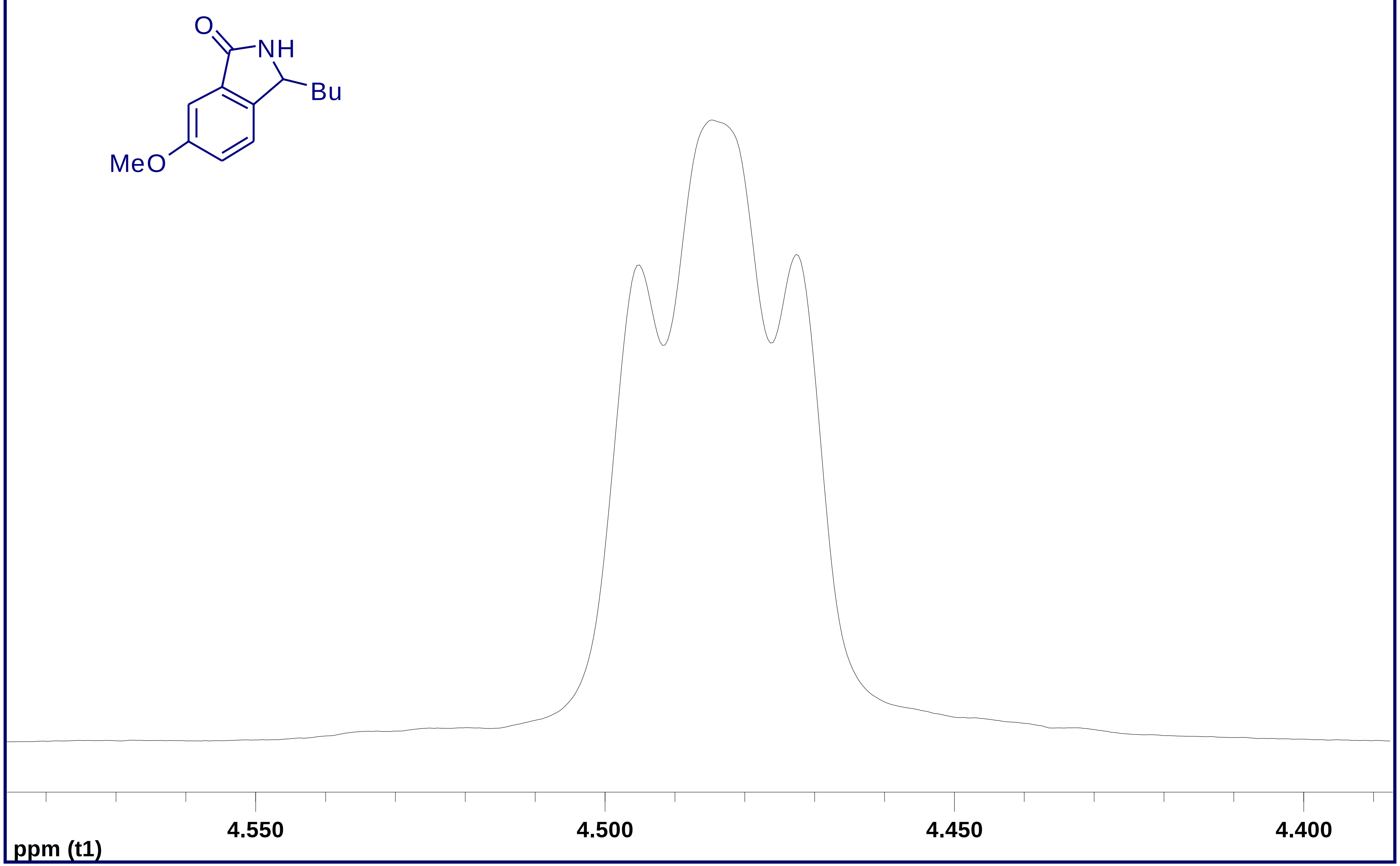

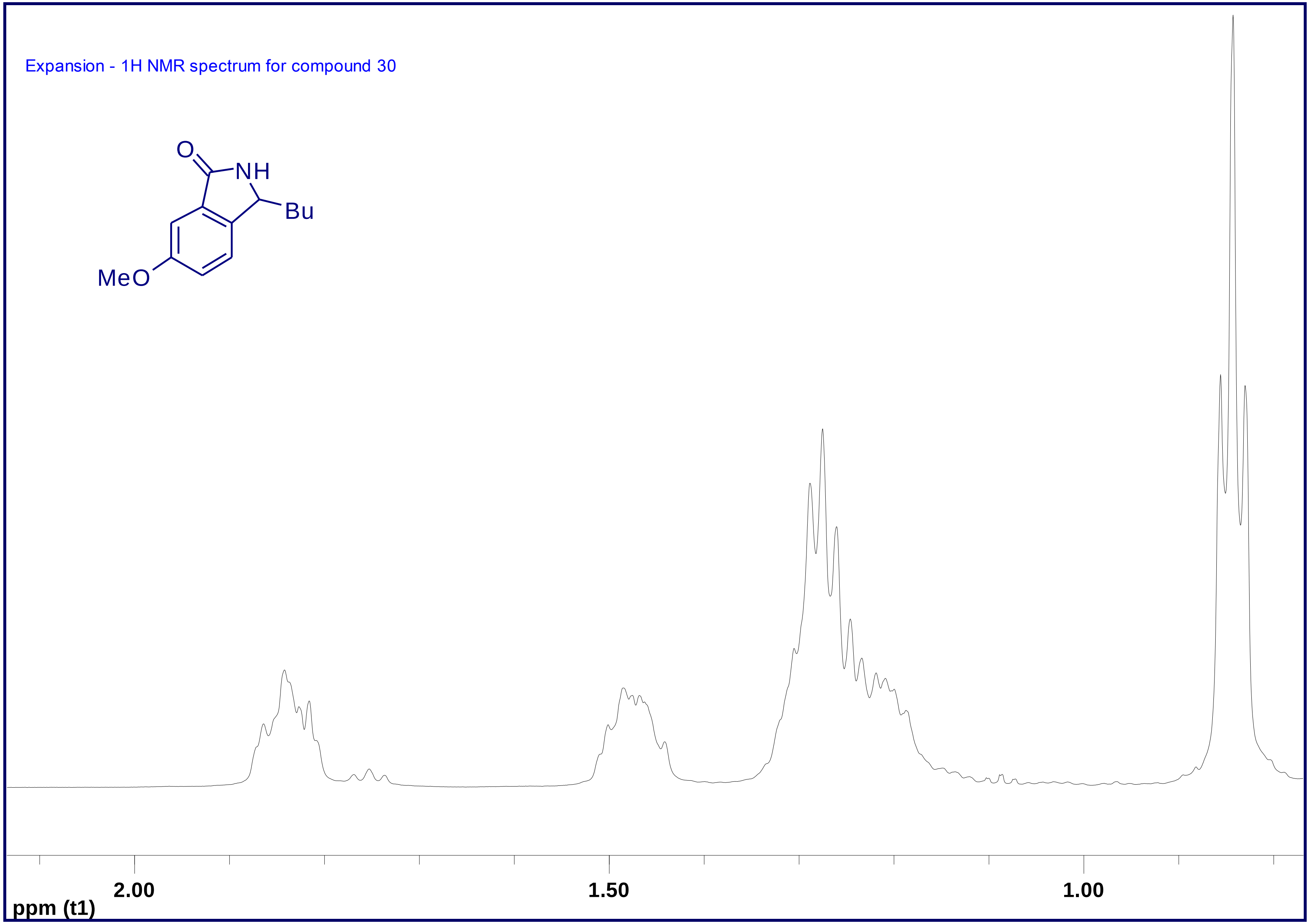
Expansion - 1H NMR spectrum for compound 30
O
NH
Bu
MeO
2.00
1.50
1.00
ppm (t1)

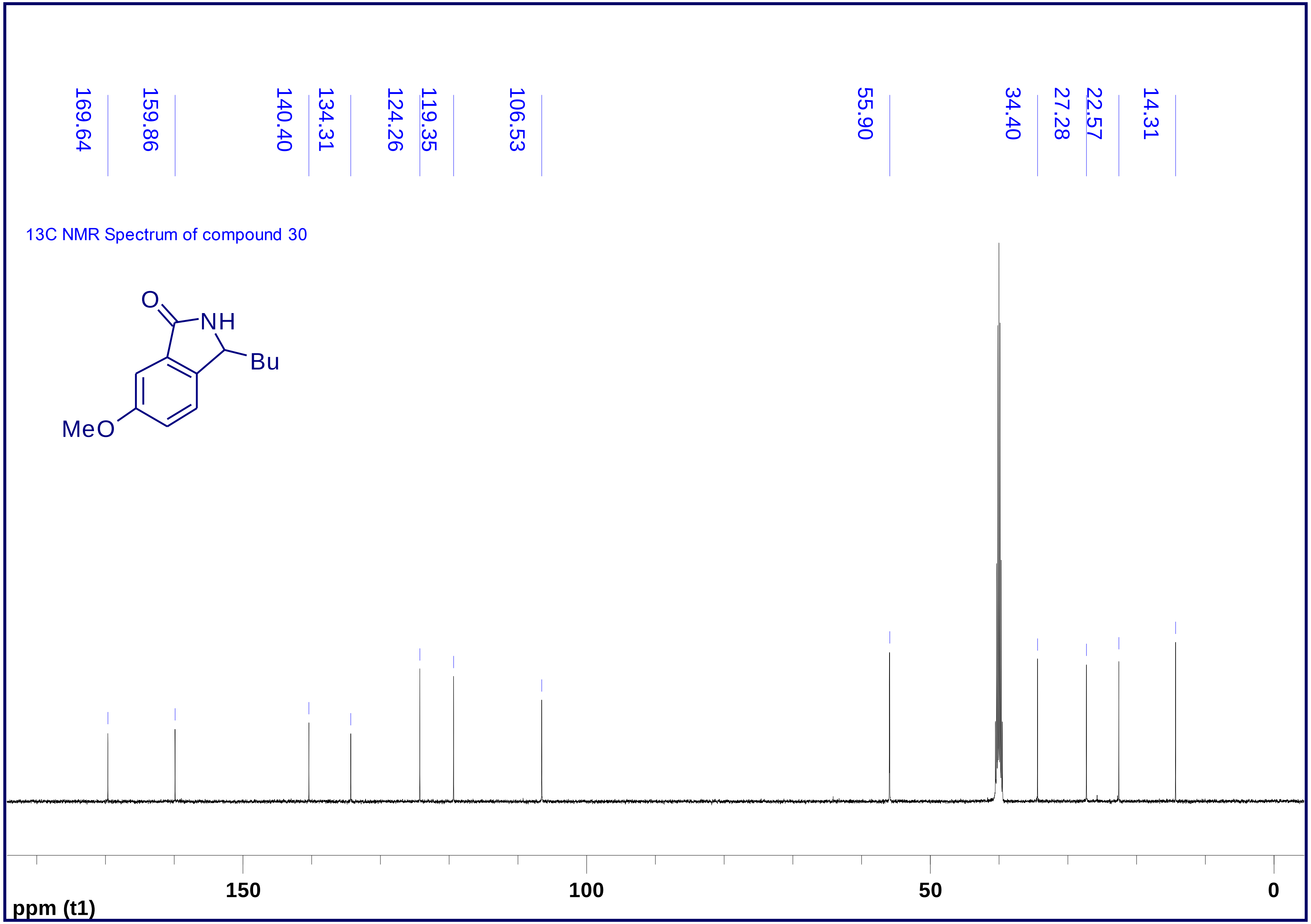
169.64
159.86
140.40
134.31
124.26
119.35
106.53
55.90
34.40
27.28
22.57
14.31
13C NMR Spectrum of compound 30
O
NH
Bu
MeO
150
100
50
0
ppm (t1)

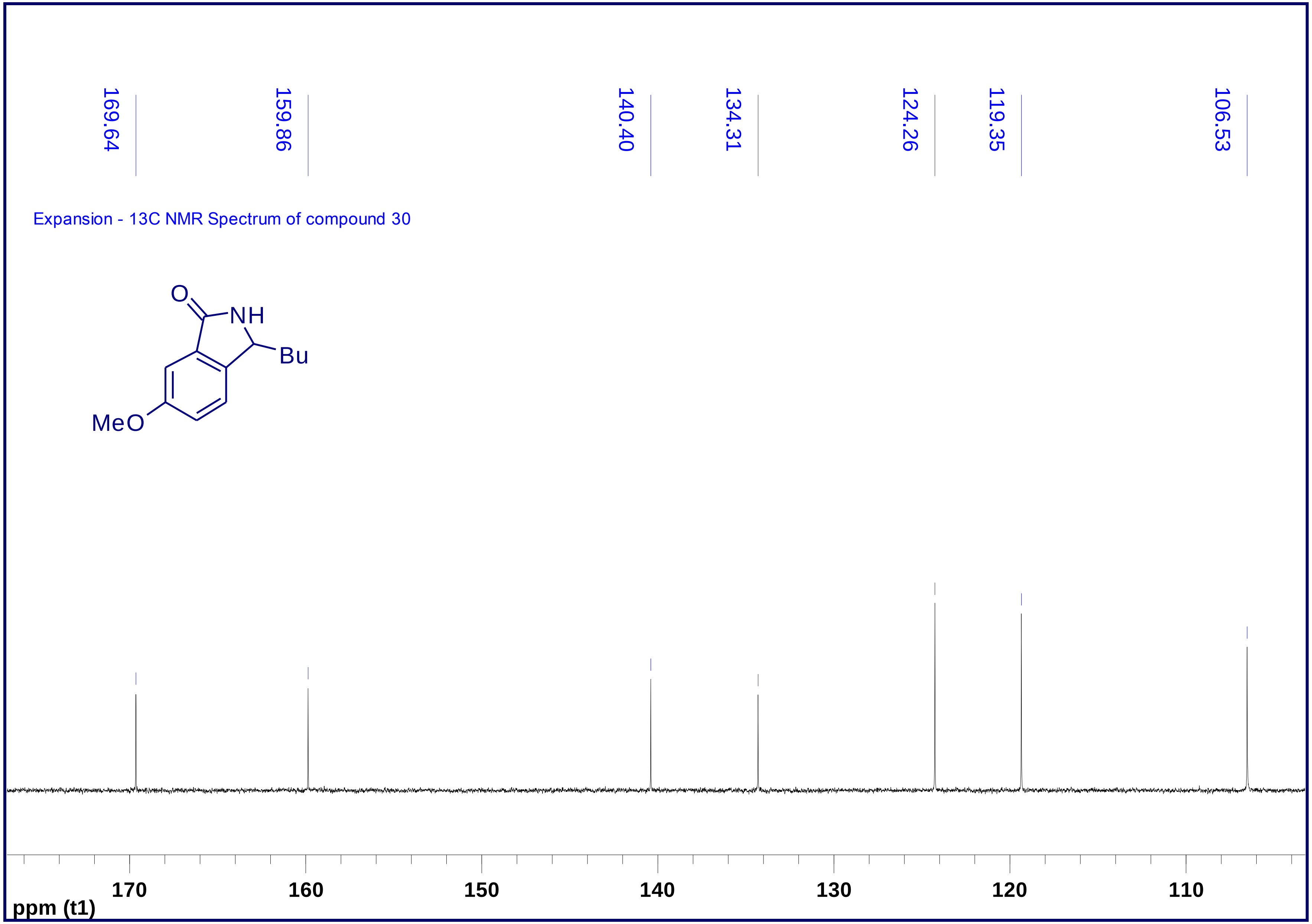

169.64
159.86
140.40
134.31
124.26
119.35
106.53
Expansion - 13C NMR Spectrum of compound 30
O
NH
Bu
MeO
170
160
150
140
130
120
110
ppm (t1)

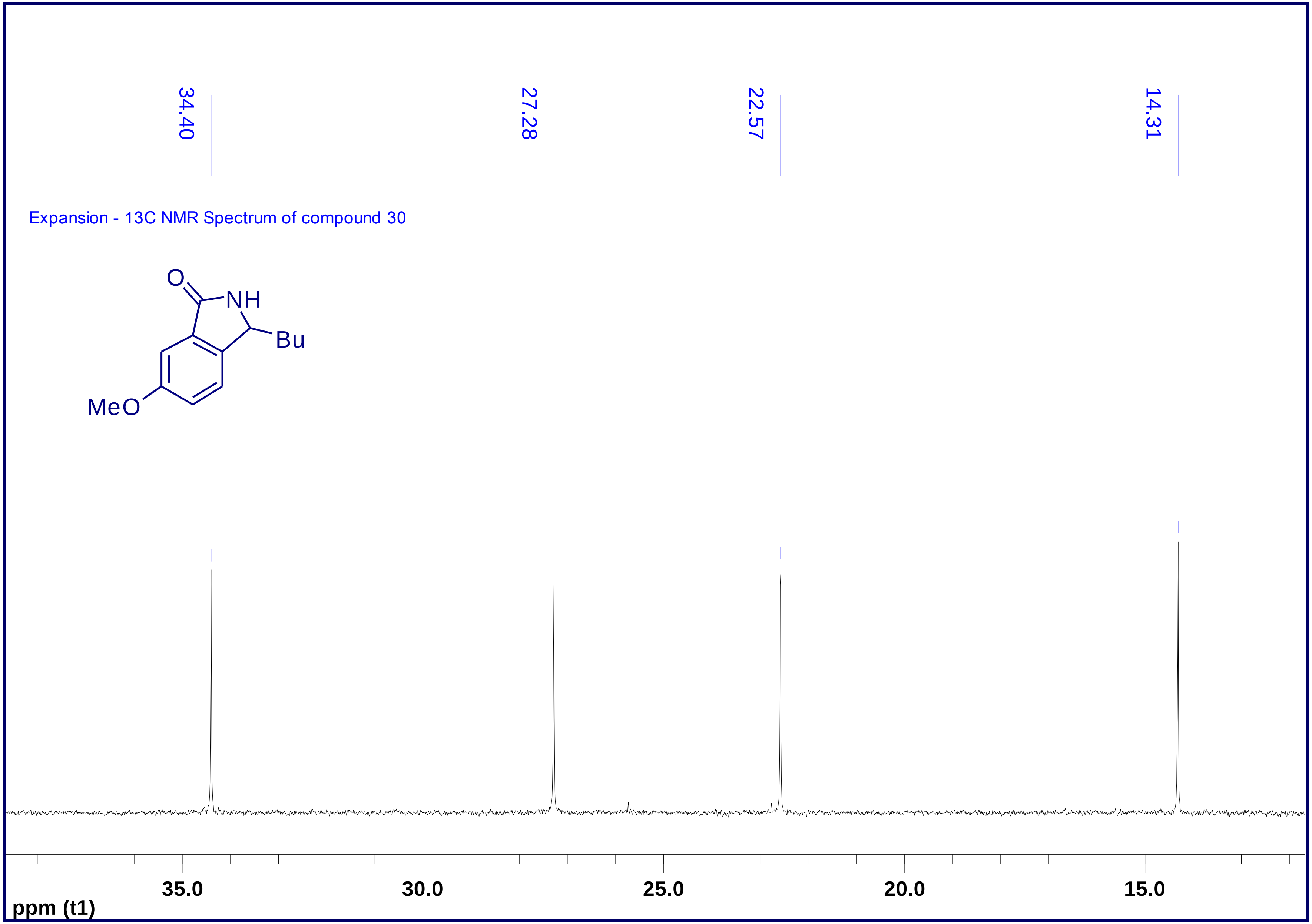
34.40
27.28
22.57
14.31
Expansion - 13C NMR Spectrum of compound 30
O
NH
Bu
MeO
35.0
30.0
25.0
20.0
15.0
ppm (t1)

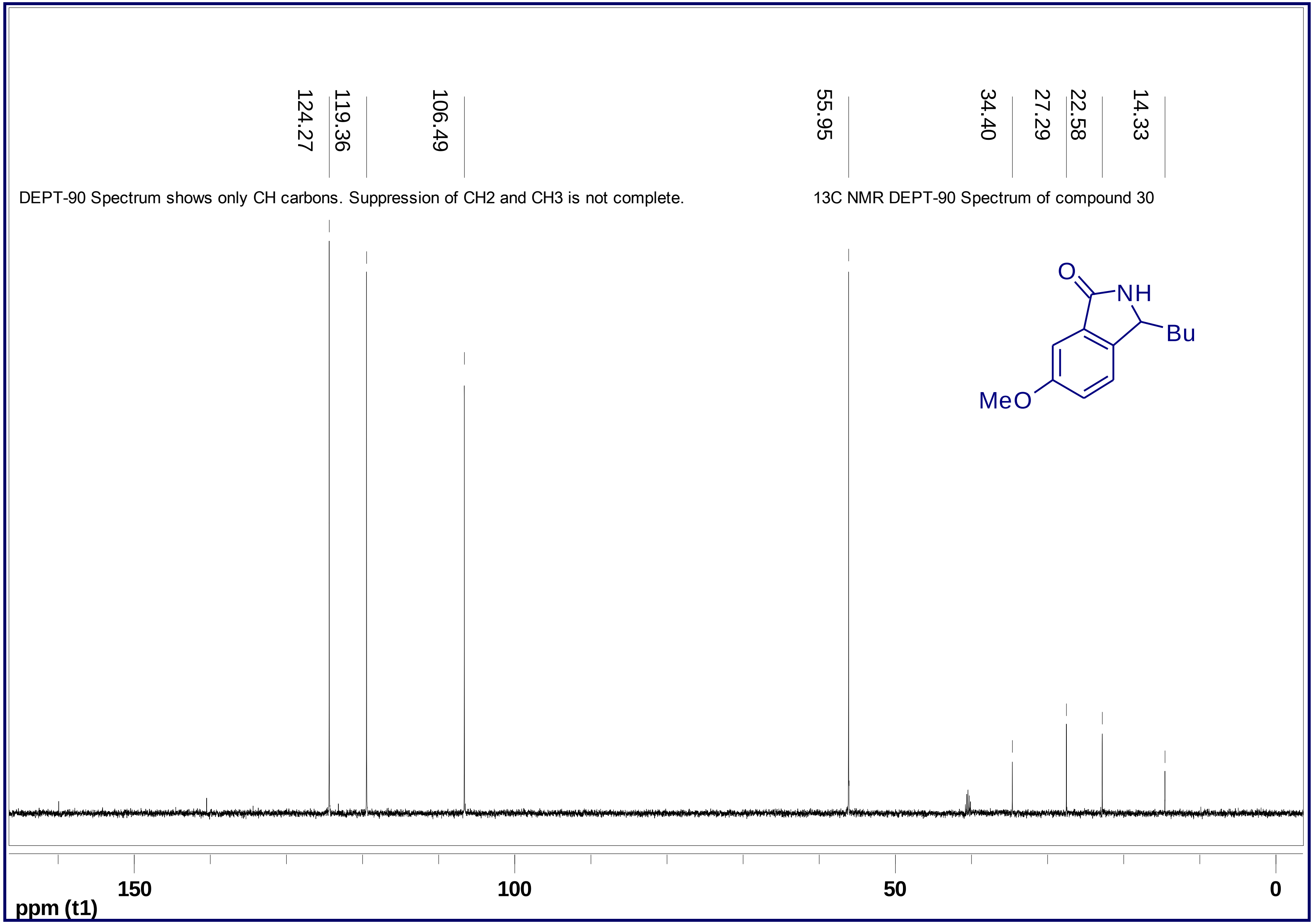
124.27
119.36
106.49
55.95
34.40
27.29
22.58
14.33
DEPT-90 Spectrum shows only CH carbons. Suppression of CH2 and CH3 is not complete.
13C NMR DEPT-90 Spectrum of compound 30
O
NH
Bu
MeO
150
100
50
0
ppm (t1)

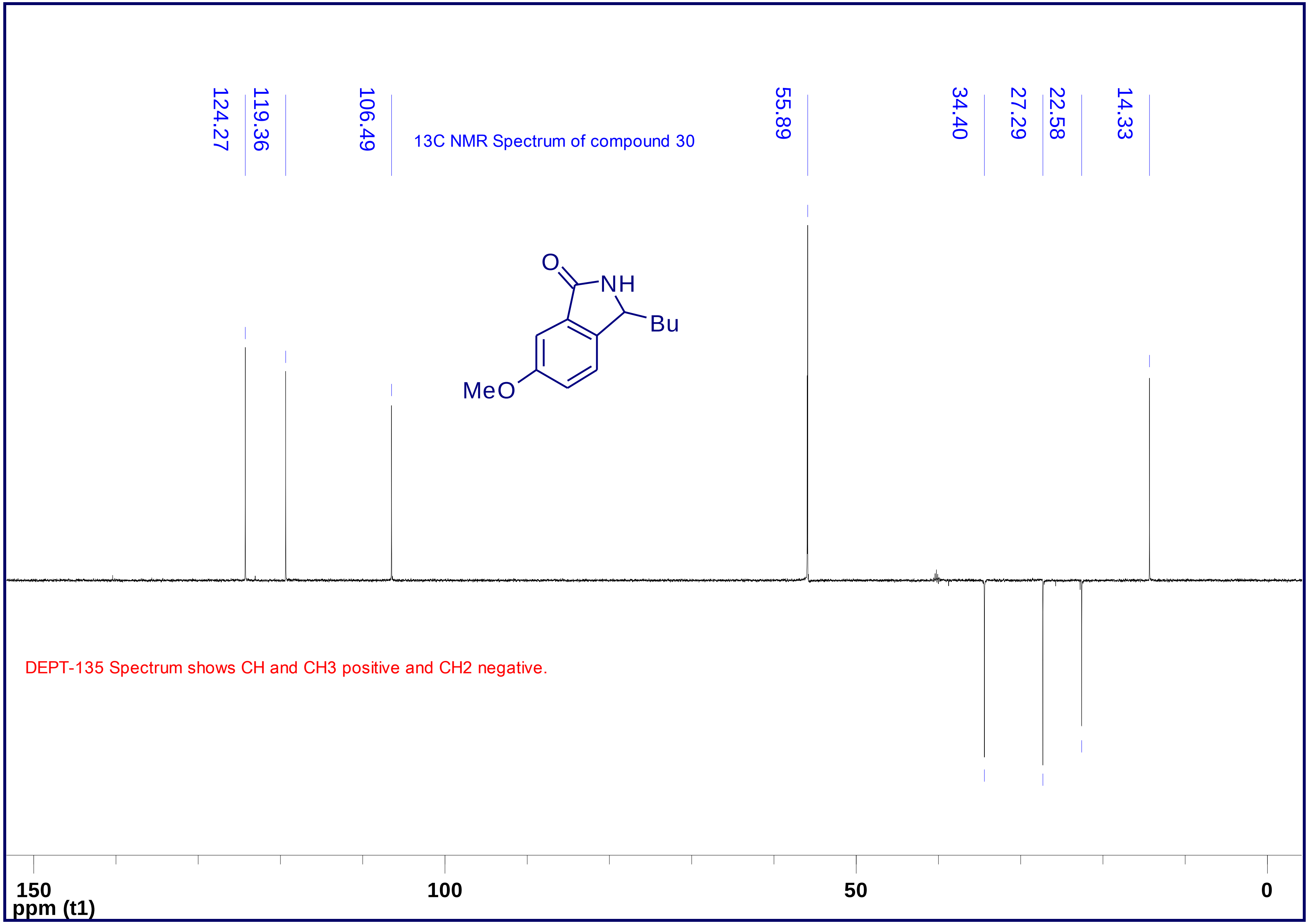

124.27
119.36
106.49
13C NMR Spectrum of compound 30
55.89
34.40
27.29
22.58
14.33
O
NH
Bu
MeO
DEPT-135 Spectrum shows CH and CH3 positive and CH2 negative.
150
100
50
0
ppm (t1)

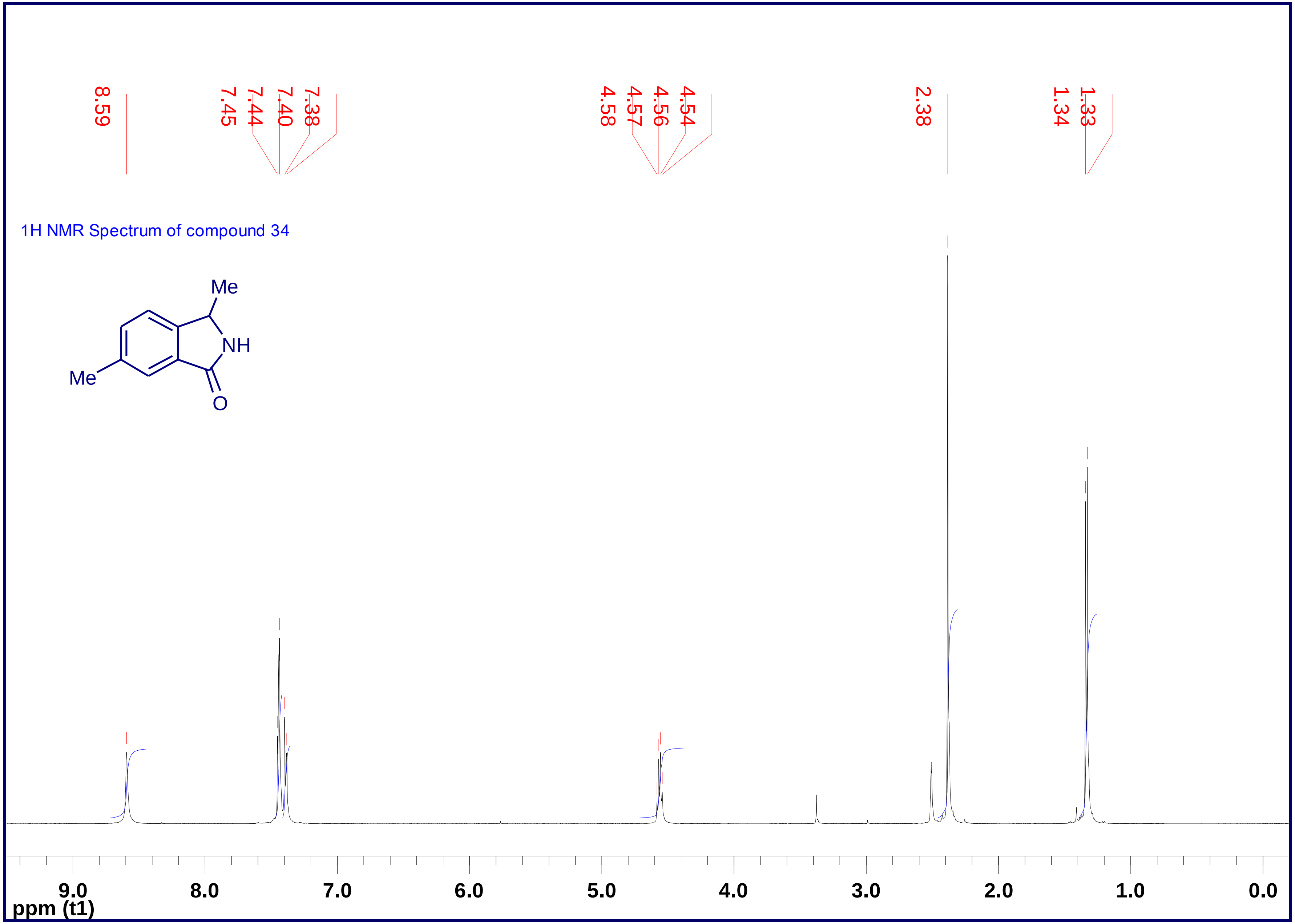

8.59
7.45
7.44
7.40
7.38
4.58
4.57
4.56
4.54
2.38
1.34
1.33
1H NMR Spectrum of compound 34
Me
NH
Me
O
9.0
8.0
7.0
6.0
5.0
4.0
3.0
2.0
1.0
0.0
ppm (t1)

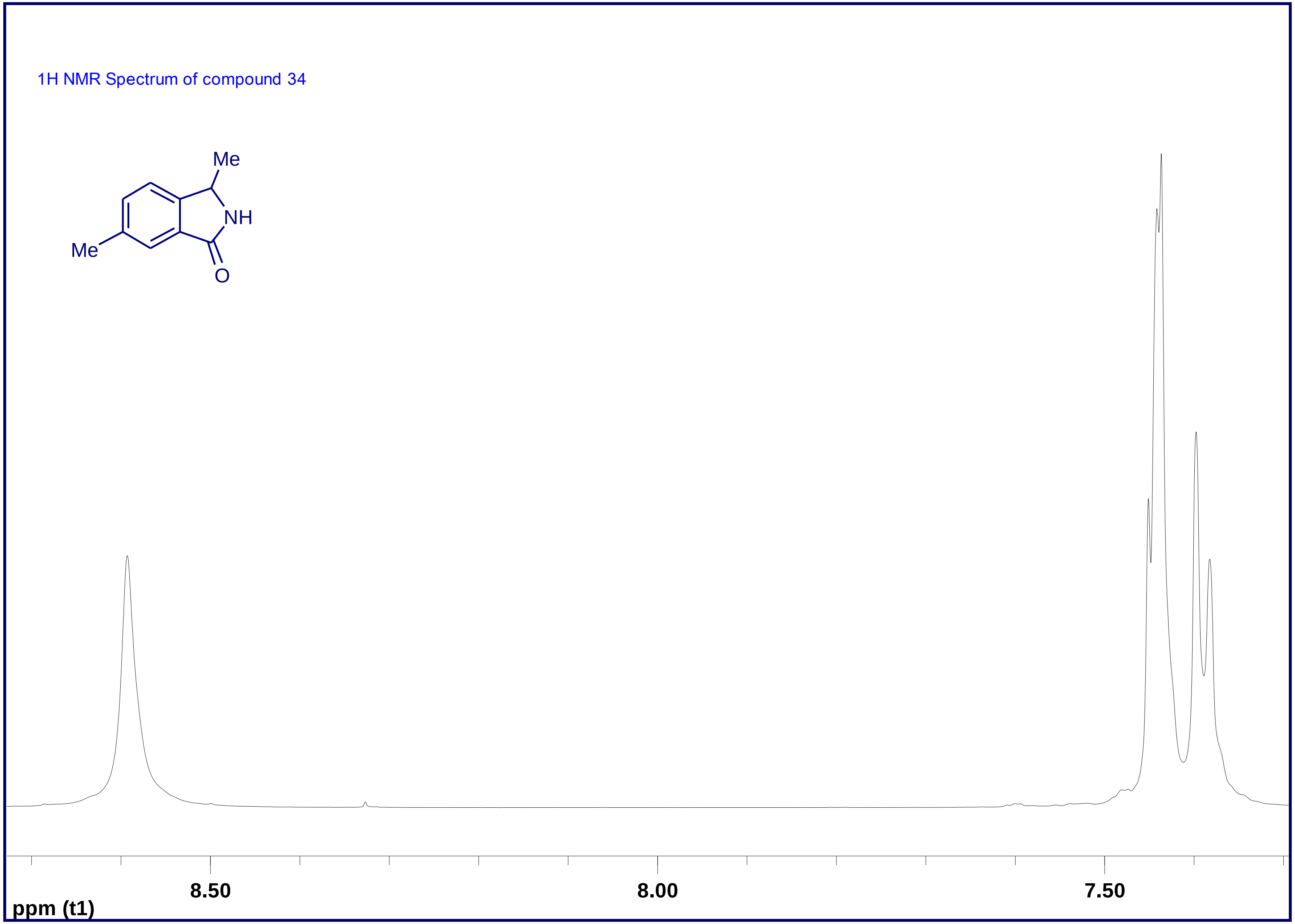
1H NMR Spectrum of compound 34
Me
NH
Me
O
8.50
8.00
7.50
ppm (t1)

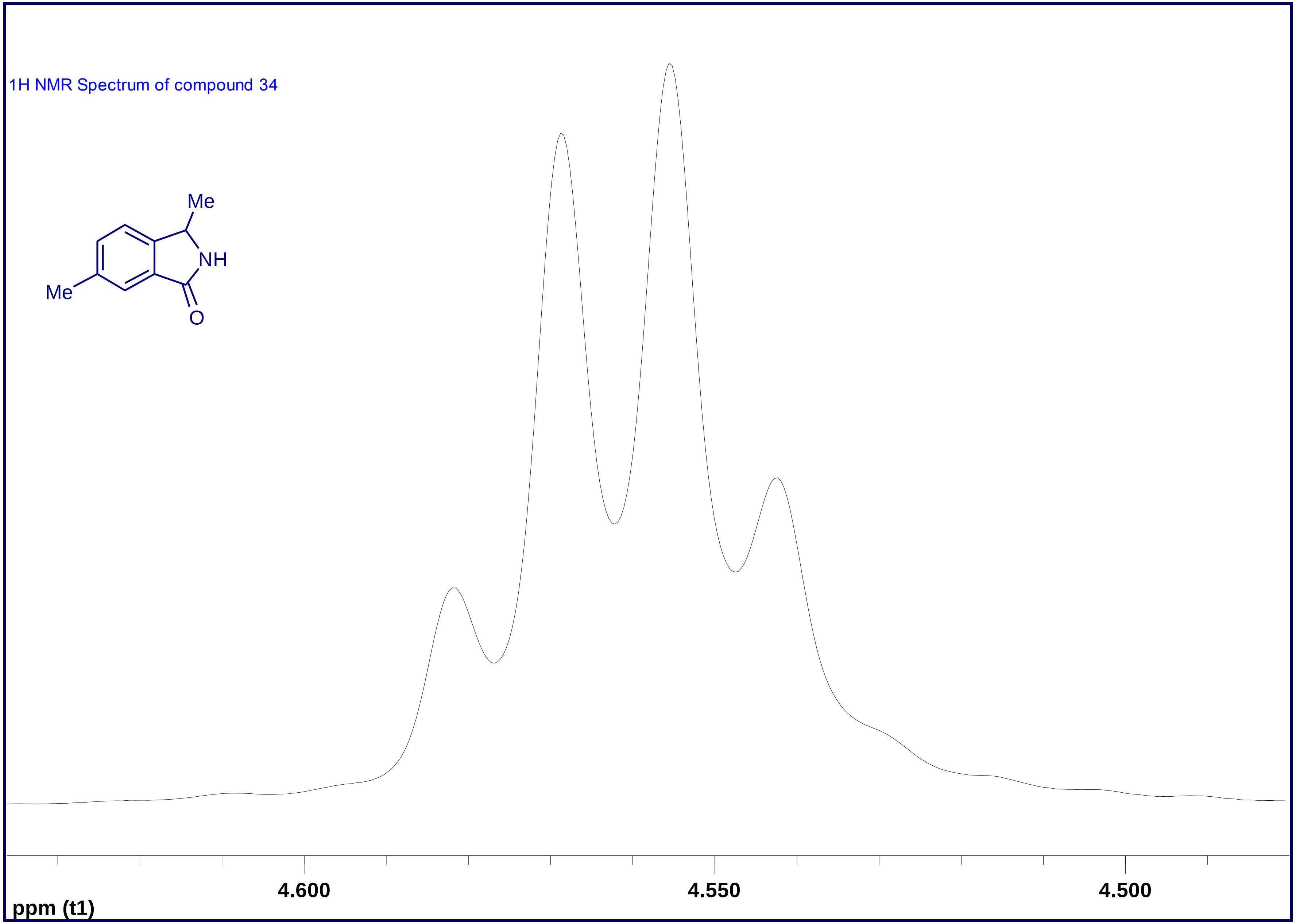

1H NMR Spectrum of compound 34
Me
NH
Me
O
4.600
4.550
4.500
ppm (t1)

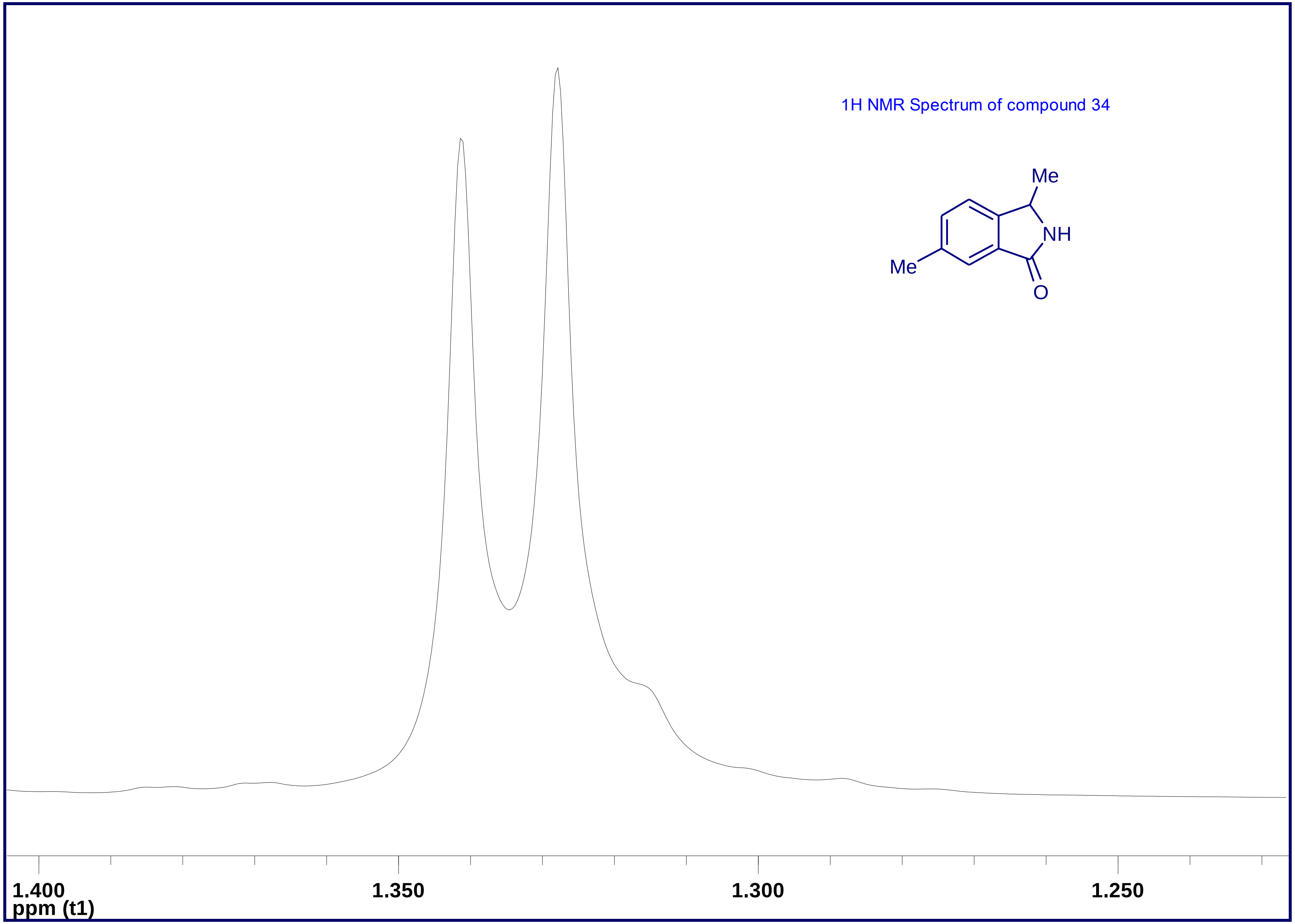
1H NMR Spectrum of compound 34
Me
NH
Me
O
1.400
ppm (t1)
1.350
1.300
1.250

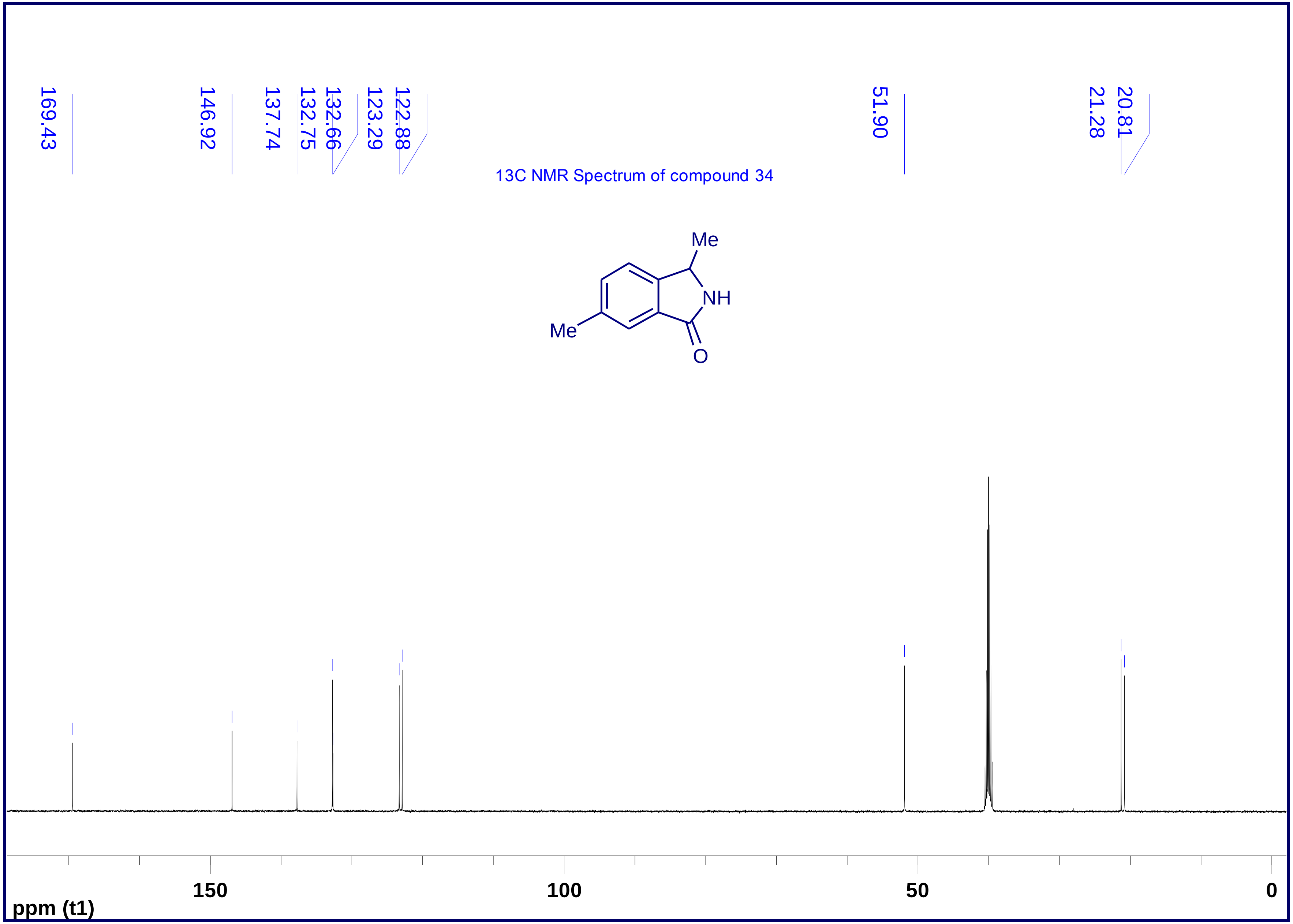
169.43
146.92
137.74
132.75
132.66
123.29
122.88
51.90
21.28
20.81
13C NMR Spectrum of compound 34
Me
NH
Me
O
150
100
50
0
ppm (t1)

Expansion - 13C NMR Spectrum of compound 34

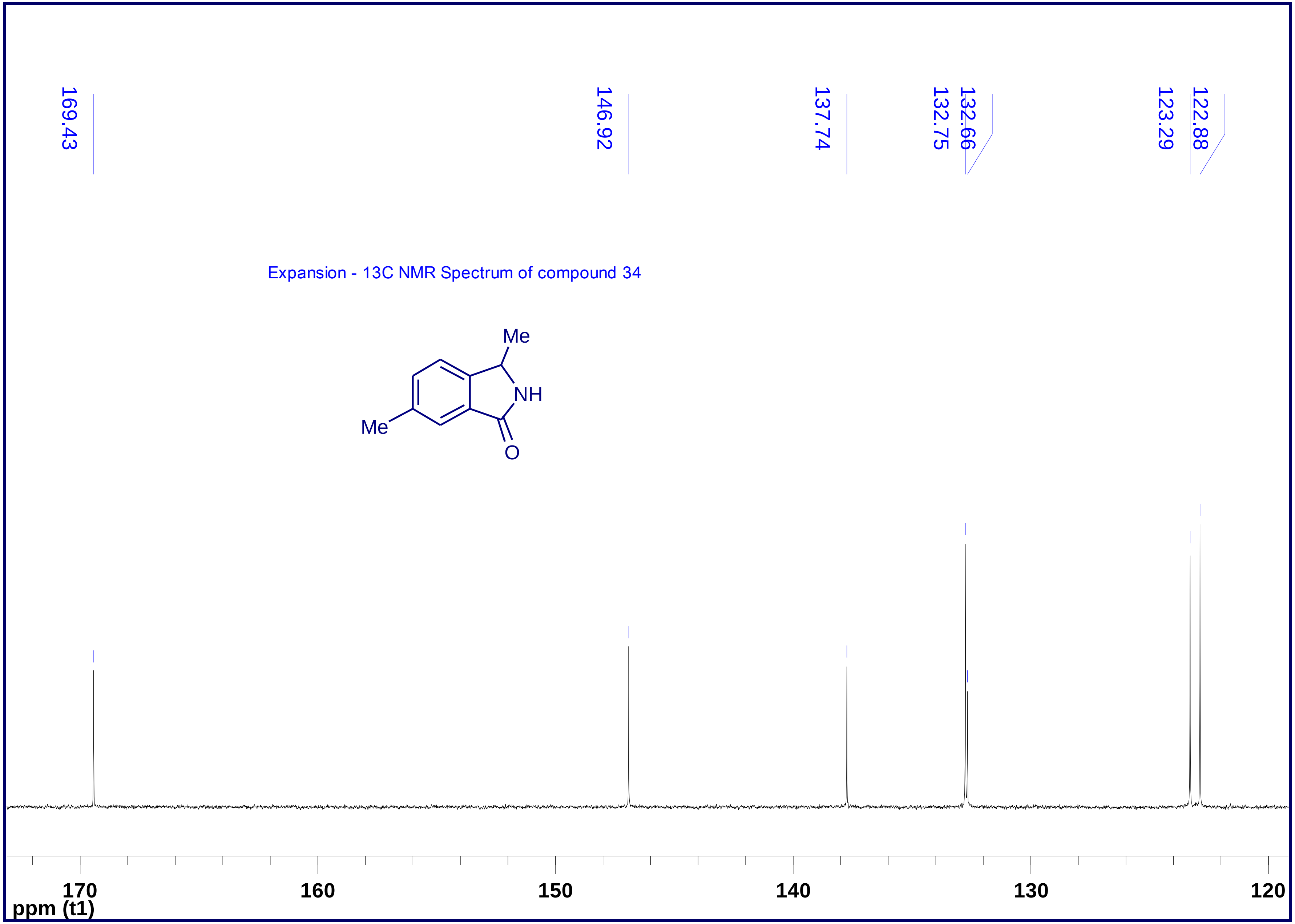

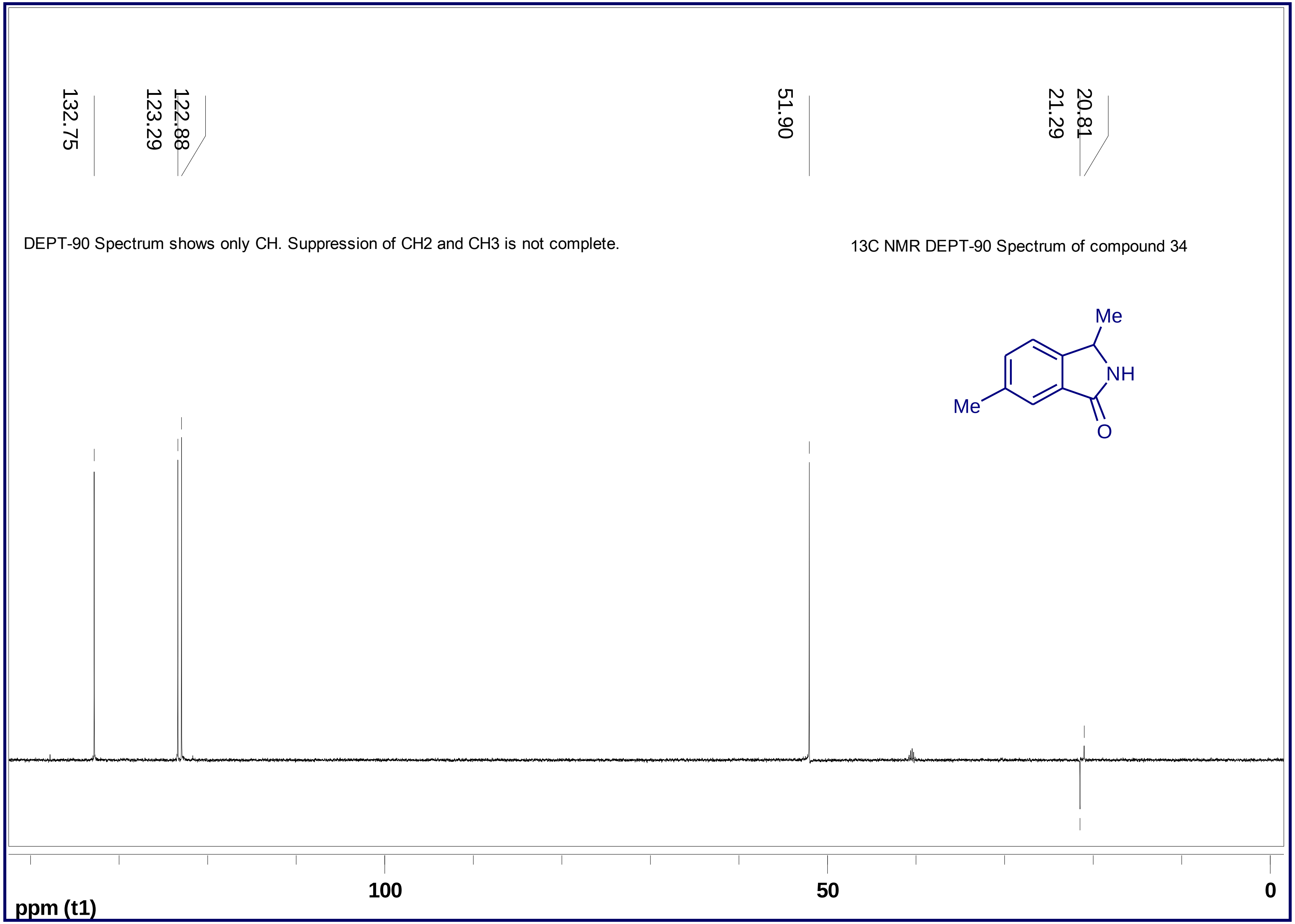
132.75
123.29
122.88
51.90
21.29
20.81
DEPT-90 Spectrum shows only CH. Suppression of CH2 and CH3 is not complete.
13C NMR DEPT-90 Spectrum of compound 34
Me
NH
Me
O
100
50
0
ppm (t1)

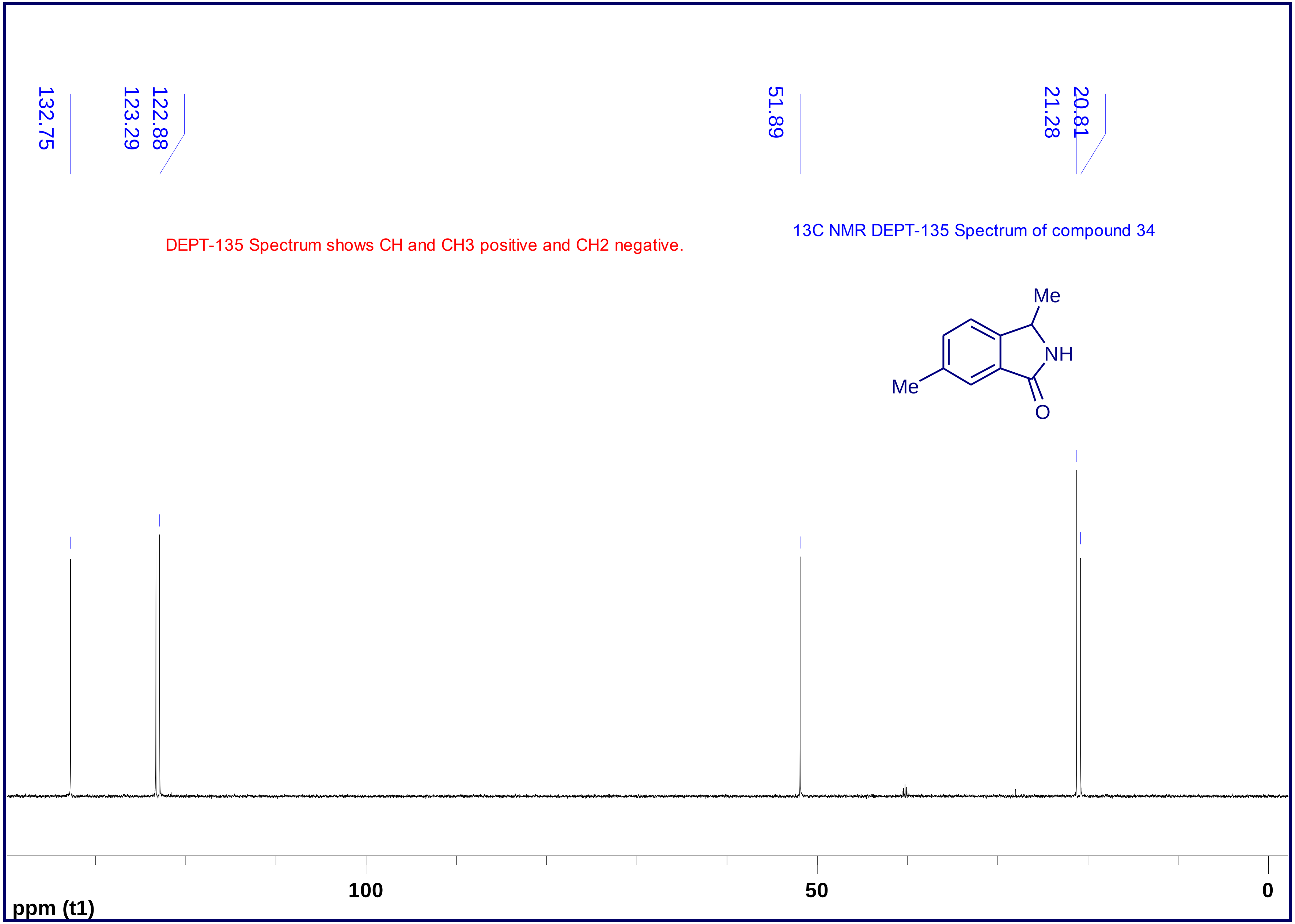

132.75
123.29
122.88
51.89
21.28
20.81
DEPT-135 Spectrum shows CH and CH3 positive and CH2 negative.
13C NMR DEPT-135 Spectrum of compound 34
Me
NH
Me
O
100
50
0
ppm (t1)

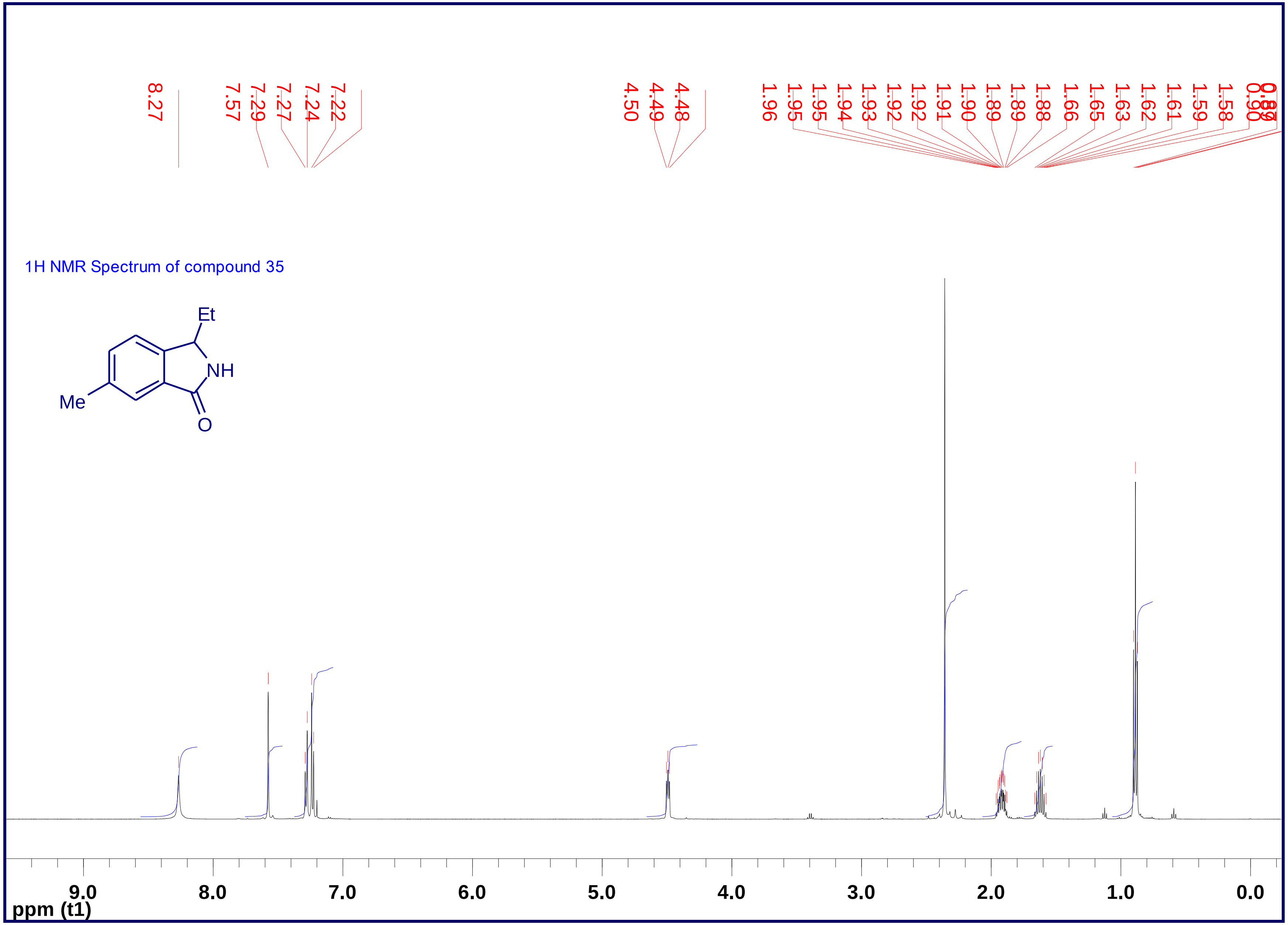
1H NMR Spectrum of compound 35
Et
NH
Me
O
8.27
7.57
7.29
7.27
7.24
7.22
4.50
4.49
4.48
1.96
1.95
1.95
1.94
1.93
1.92
1.92
1.91
1.90
1.89
1.89
1.88
1.66
1.65
1.63
1.62
1.61
1.59
1.58
0.90
9.0
8.0
7.0
6.0
5.0
4.0
3.0
2.0
1.0
0.0
ppm (t1)

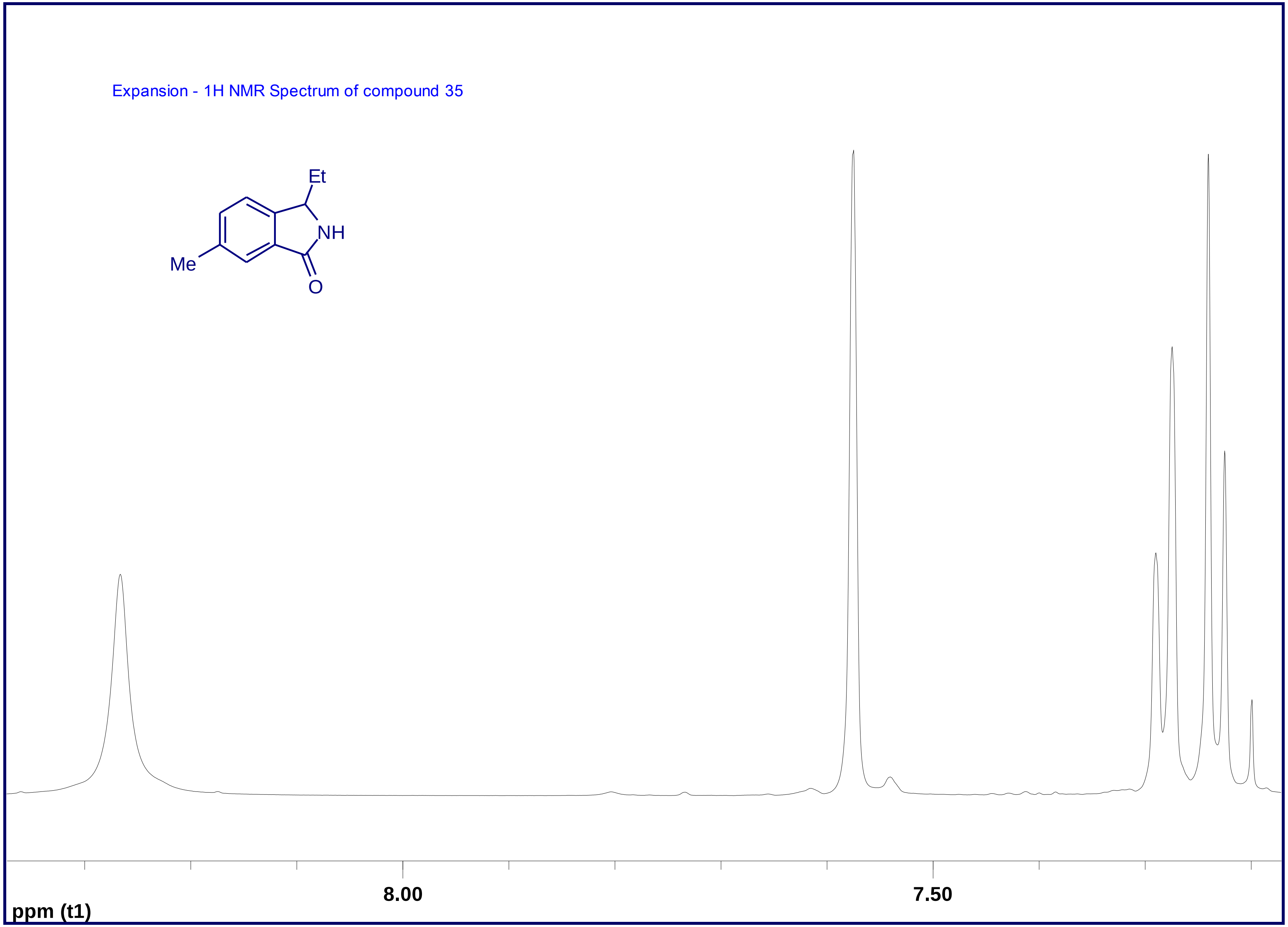
Expansion - 1H NMR Spectrum of compound 35
Et
NH
Me
O
8.00
7.50
ppm (t1)

Expansion - 1H NMR Spectrum of compound 35

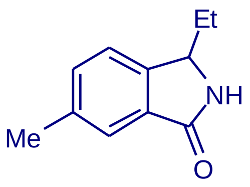

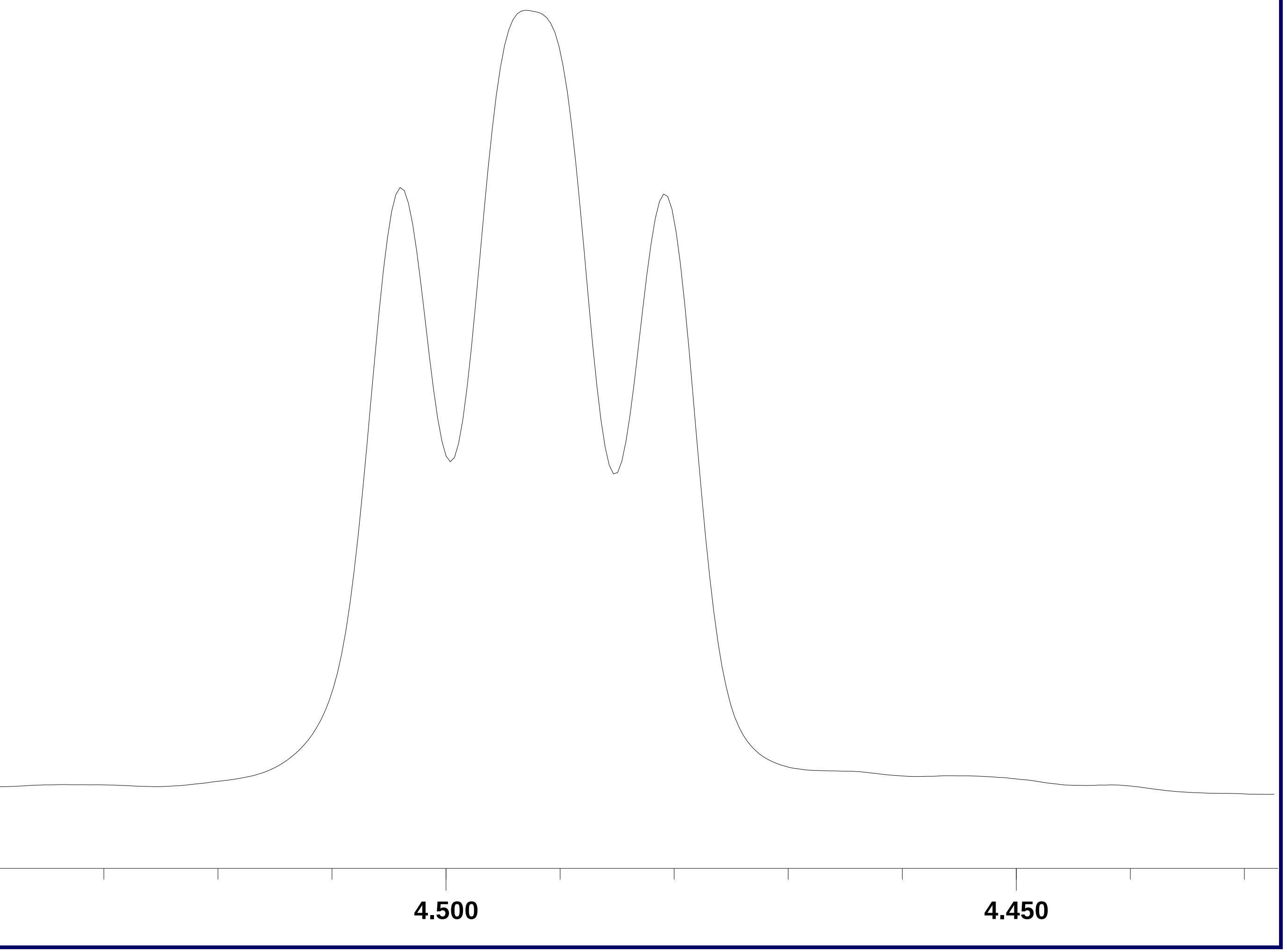

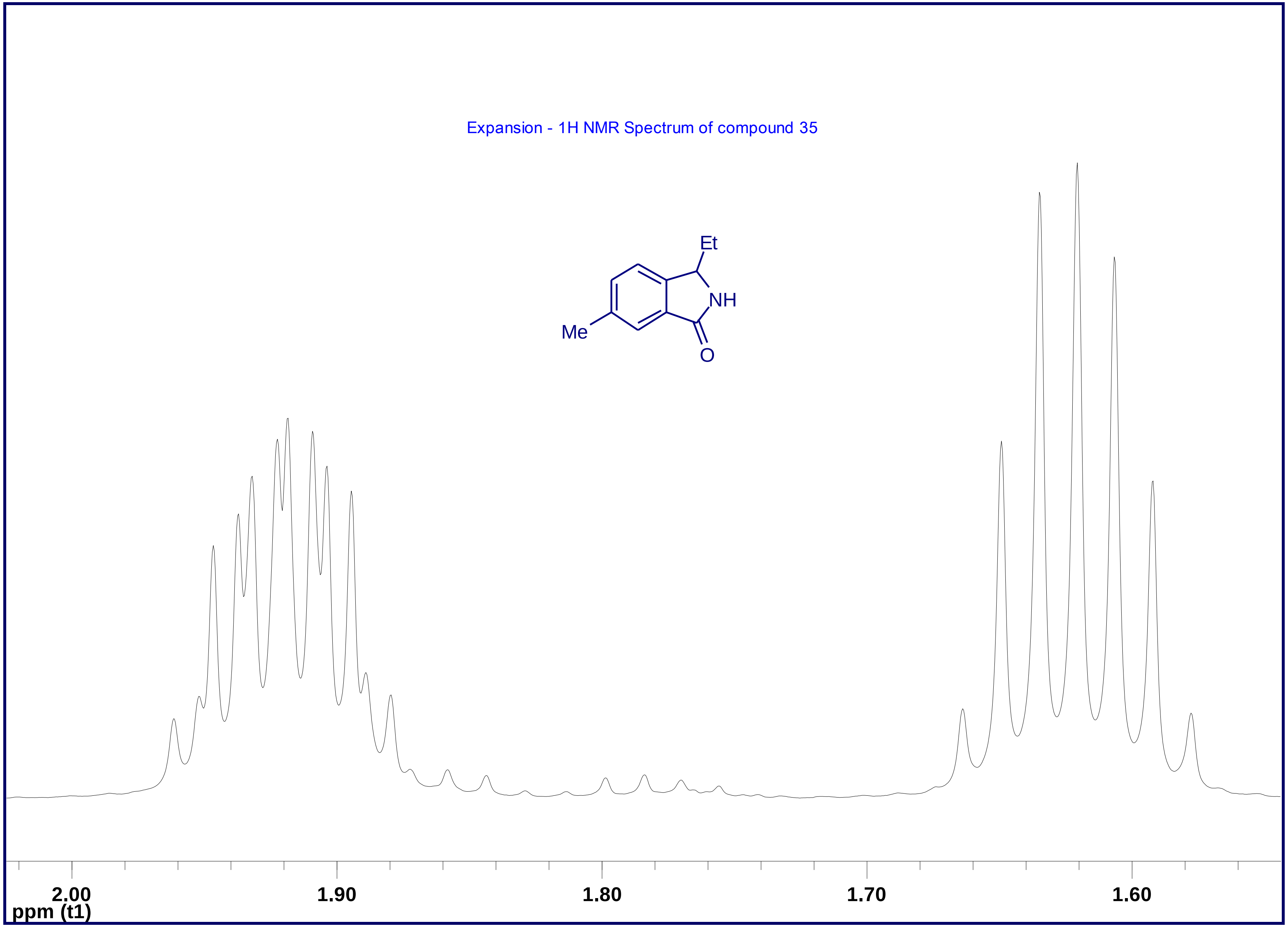
Expansion - 1H NMR Spectrum of compound 35
Et
NH
Me
O
2.00
1.90
1.80
1.70
1.60
ppm (t1)

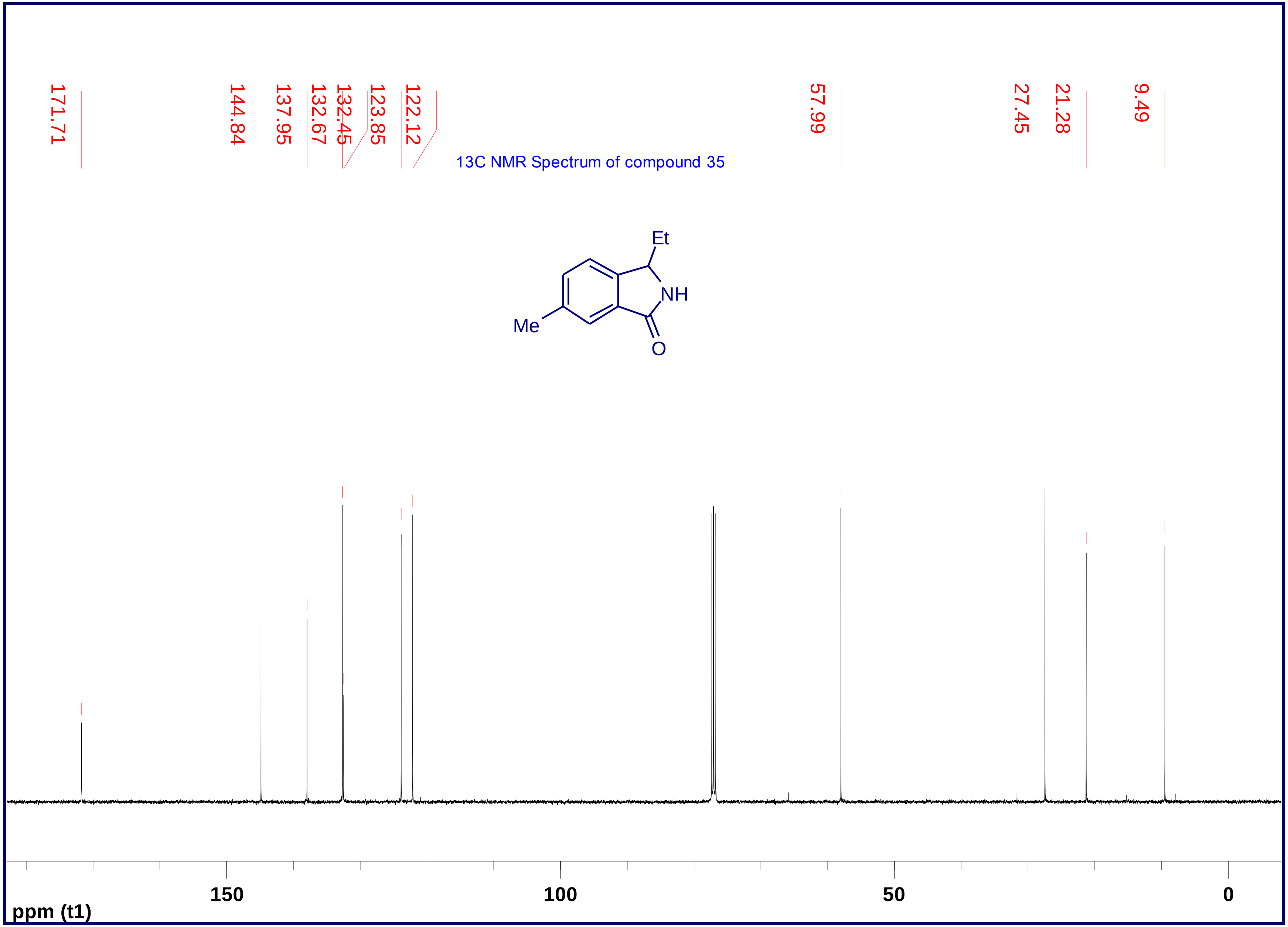

171.71
144.84
137.95
132.67
132.45
123.85
122.12
57.99
27.45
21.28
9.49
13C NMR Spectrum of compound 35
Et
NH
O
Me
150
100
50
0
ppm (t1)

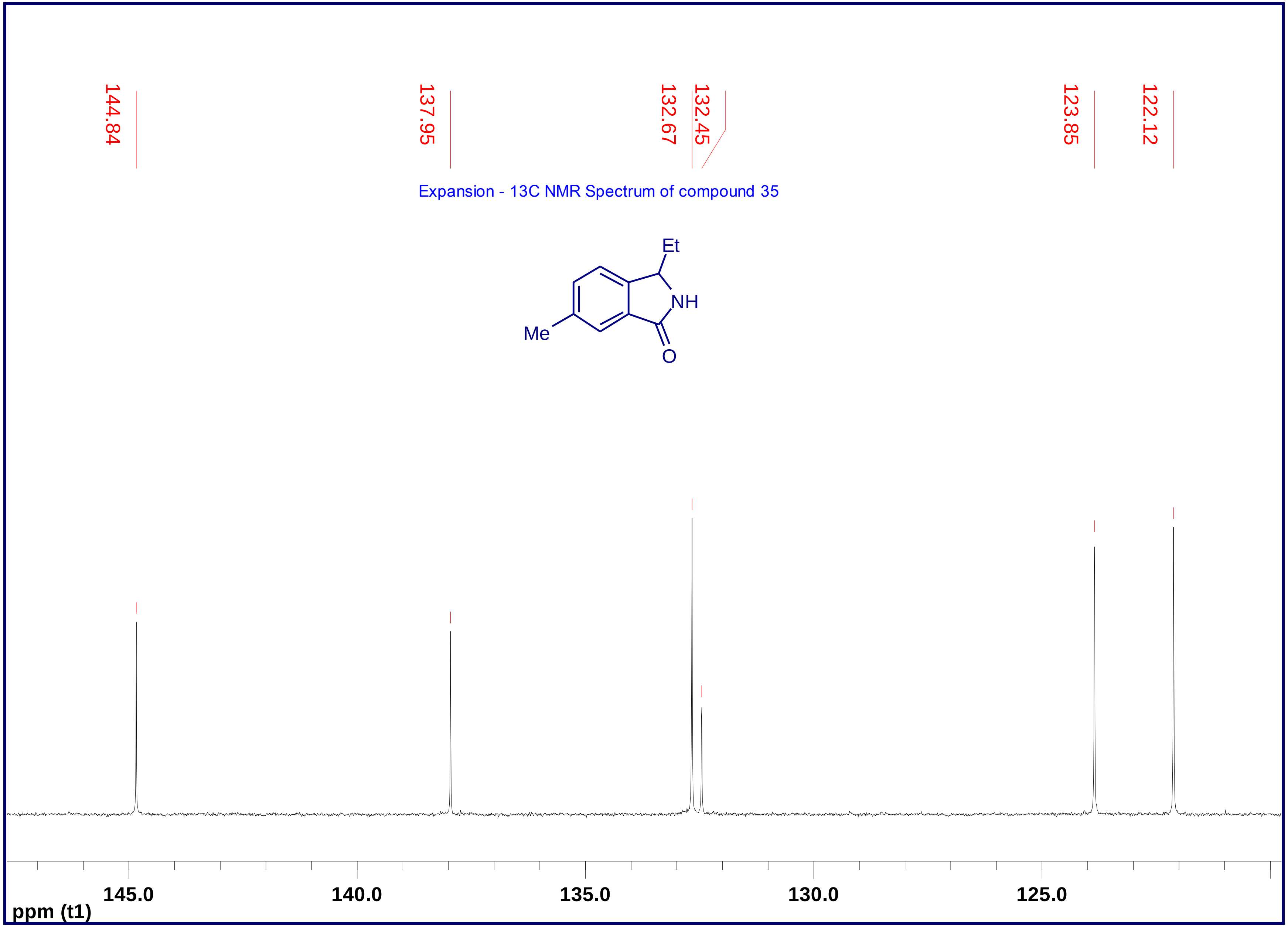

144.84
137.95
132.67
132.45
123.85
122.12
Expansion - 13C NMR Spectrum of compound 35
Et
NH
Me
O
145.0
140.0
135.0
130.0
125.0
ppm (t1)

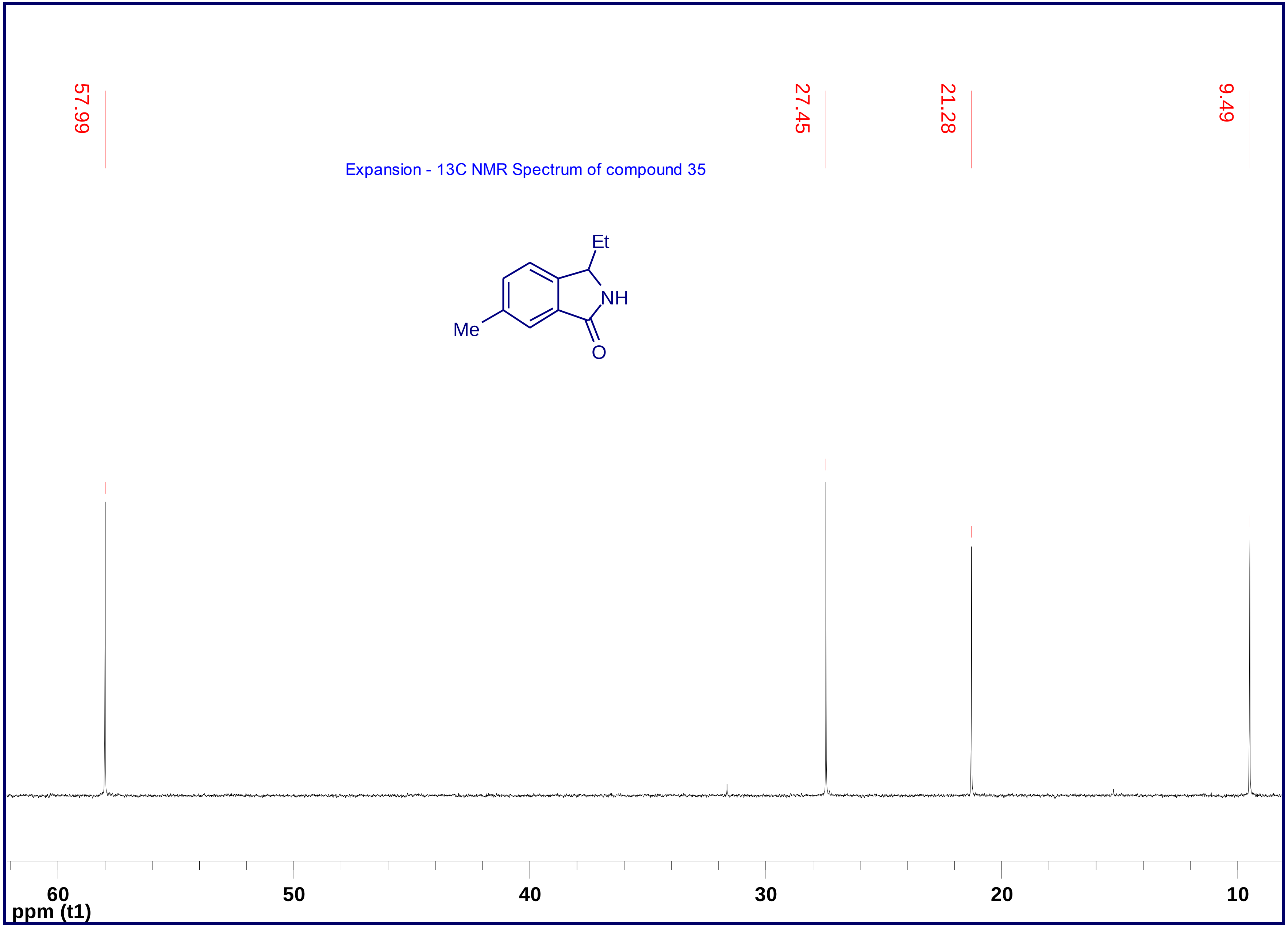

57.99
27.45
21.28
9.49
Expansion - 13C NMR Spectrum of compound 35
Et
NH
Me
O
60
50
40
30
20
10
ppm (t1)

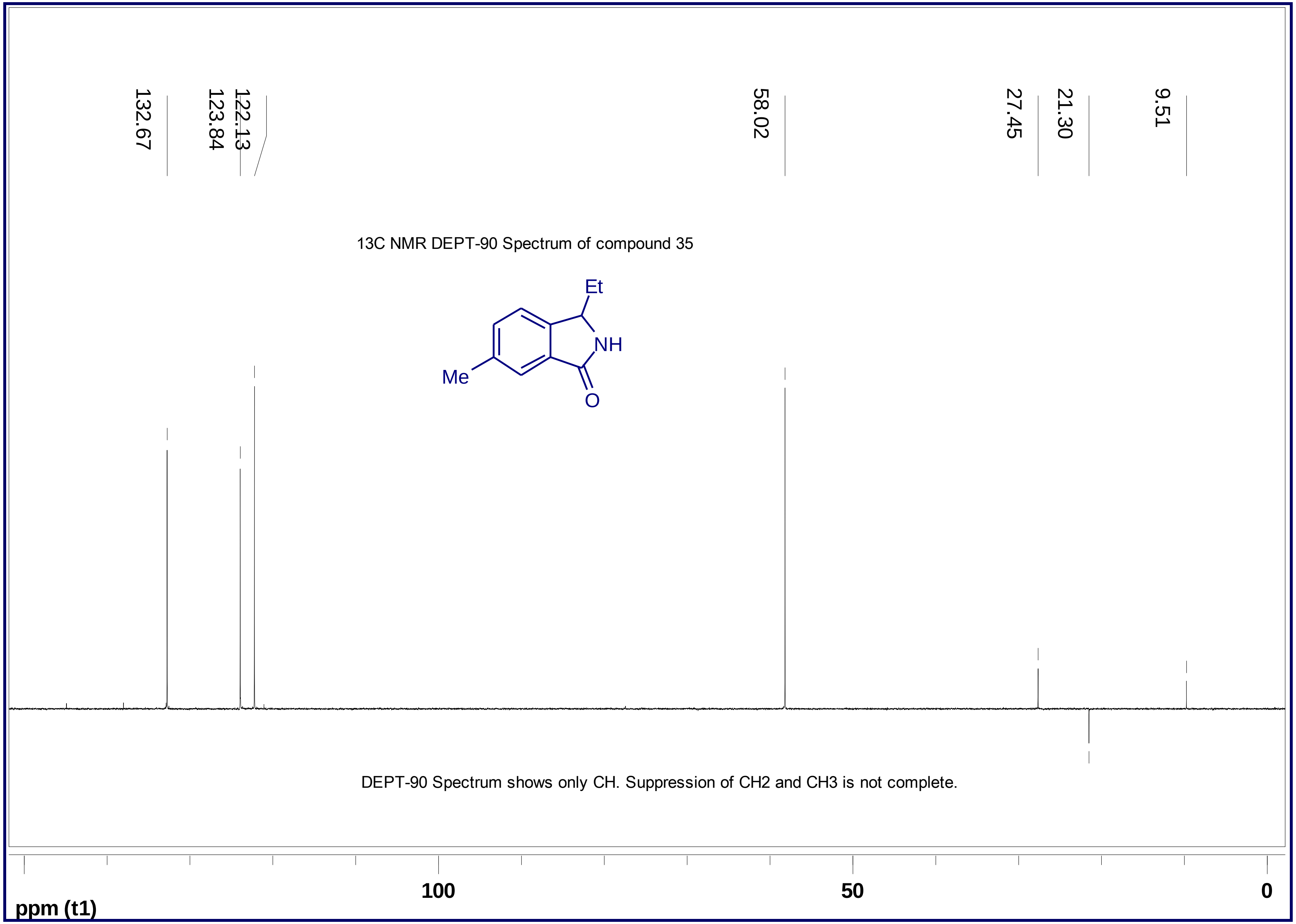
132.67
123.84
122.13
58.02
27.45
21.30
9.51
13C NMR DEPT-90 Spectrum of compound 35
Et
NH
Me
O
DEPT-90 Spectrum shows only CH. Suppression of CH2 and CH3 is not complete.
100
50
0
ppm (t1)

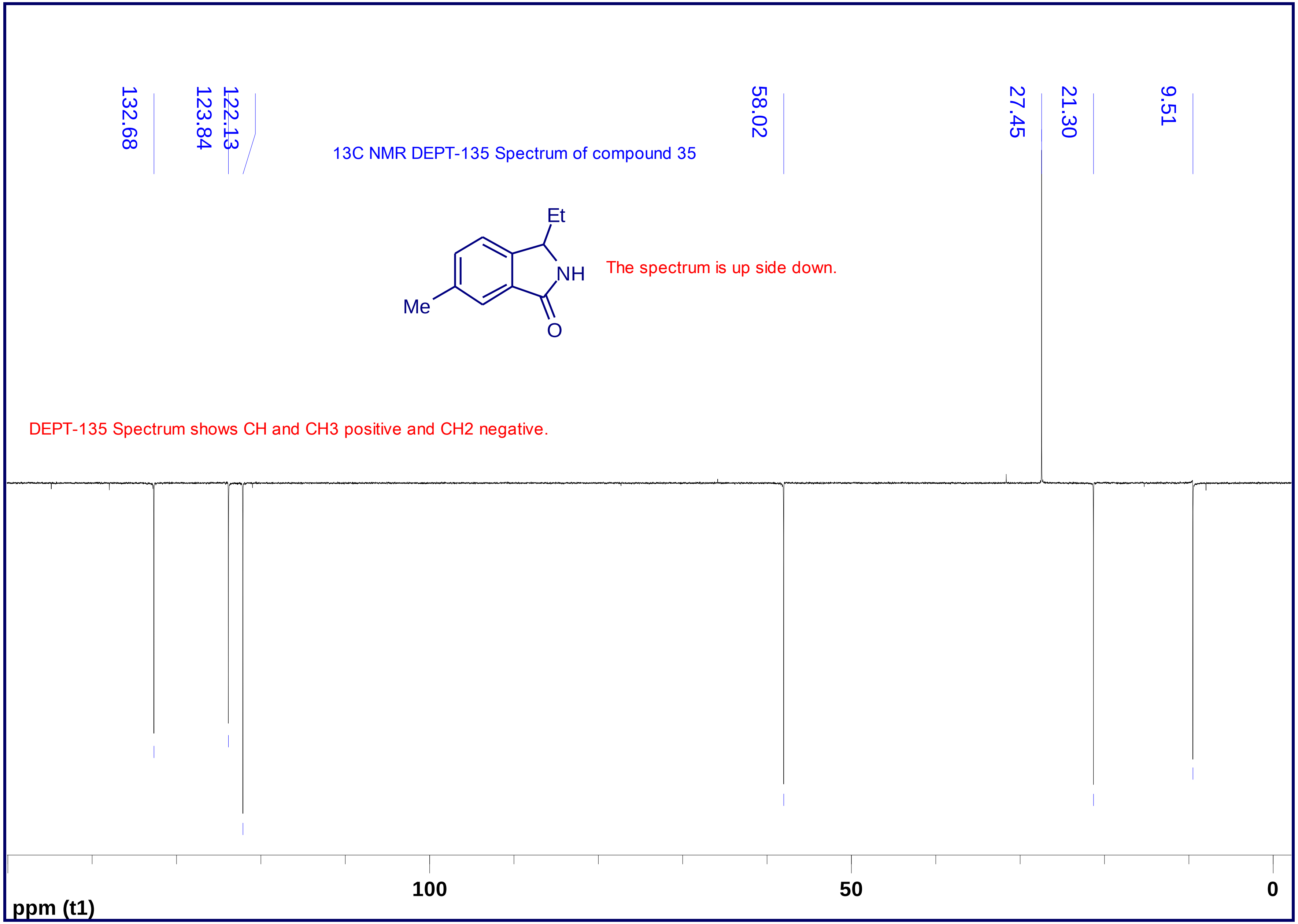

132.68
123.84
122.13
58.02
27.45
21.30
9.51
13C NMR DEPT-135 Spectrum of compound 35
Et
NH
O
Me
The spectrum is up side down.
DEPT-135 Spectrum shows CH and CH3 positive and CH2 negative.
100
50
0
ppm (t1)

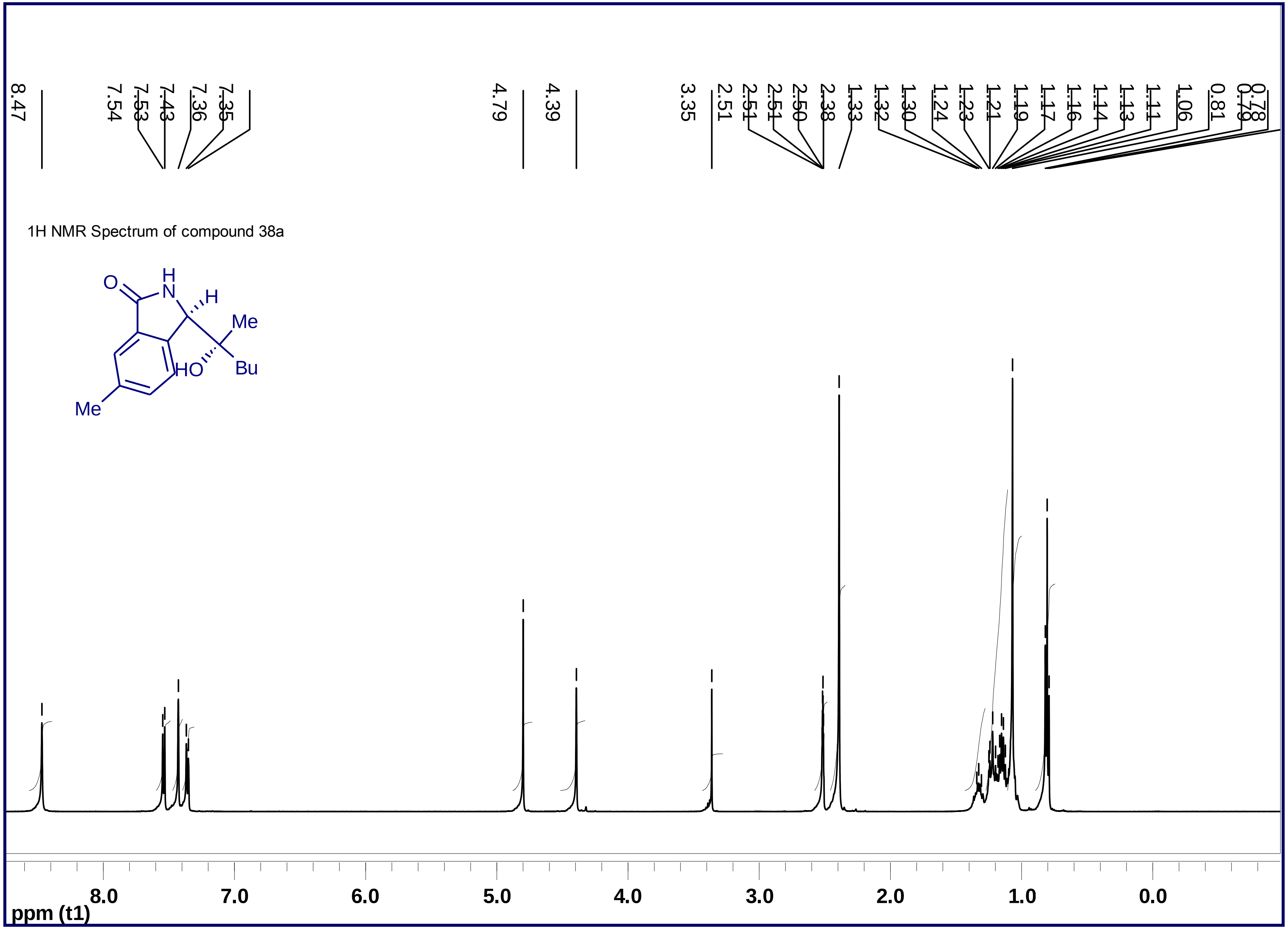
8.47
7.54
7.53
7.43
7.36
7.35
4.79
4.39
3.35
2.51
2.51
2.51
2.50
2.38
1.33
1.32
1.30
1.24
1.23
1.21
1.19
1.17
1.16
1.14
1.13
1.11
1.06
0.81
0.79
0.78
1H NMR Spectrum of compound 38a
O
H
N
H
Me
HO
Bu
Me
8.0
7.0
6.0
5.0
4.0
3.0
2.0
1.0
0.0
ppm (t1)

Expansion - 1H NMR Spectrum of compound 38a

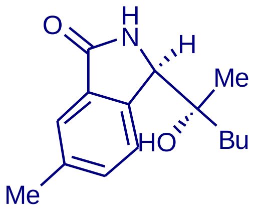

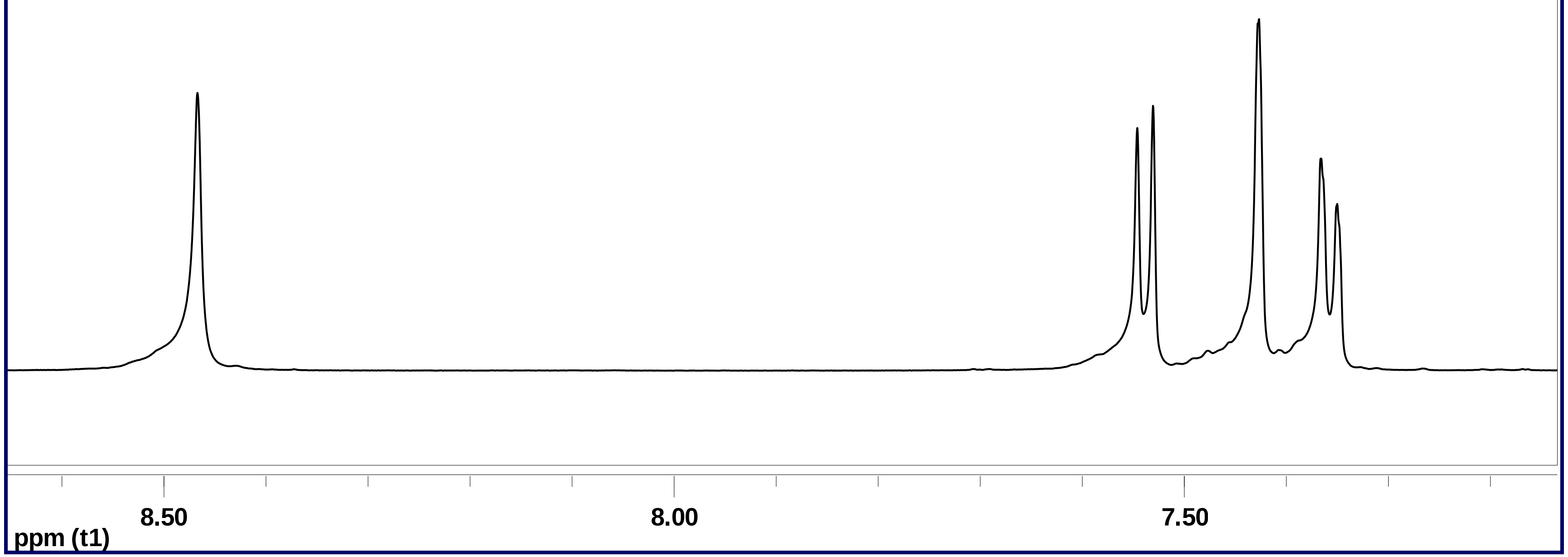

Expansion - 1H NMR Spectrum of compound 38a

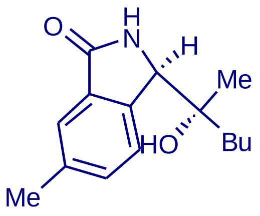

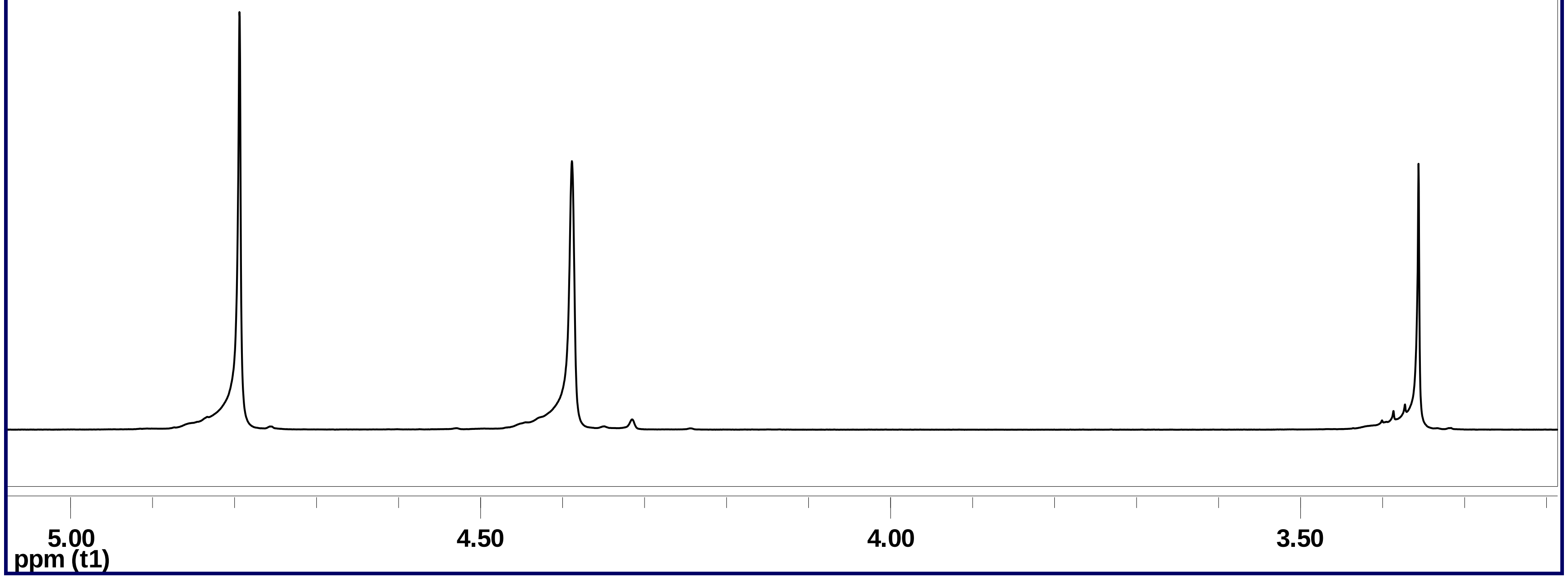

Expansion - 1H NMR Spectrum of compound 38a

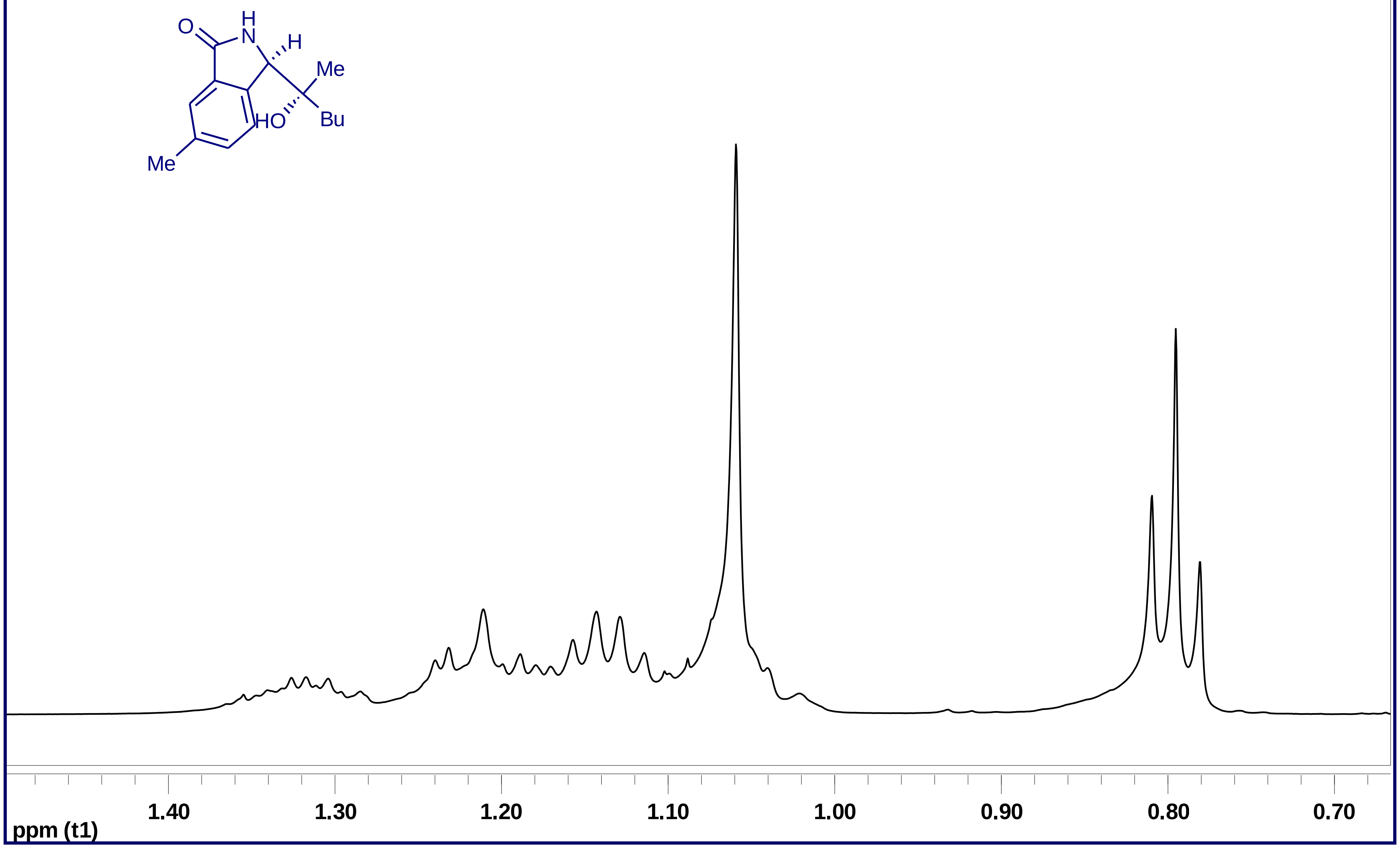

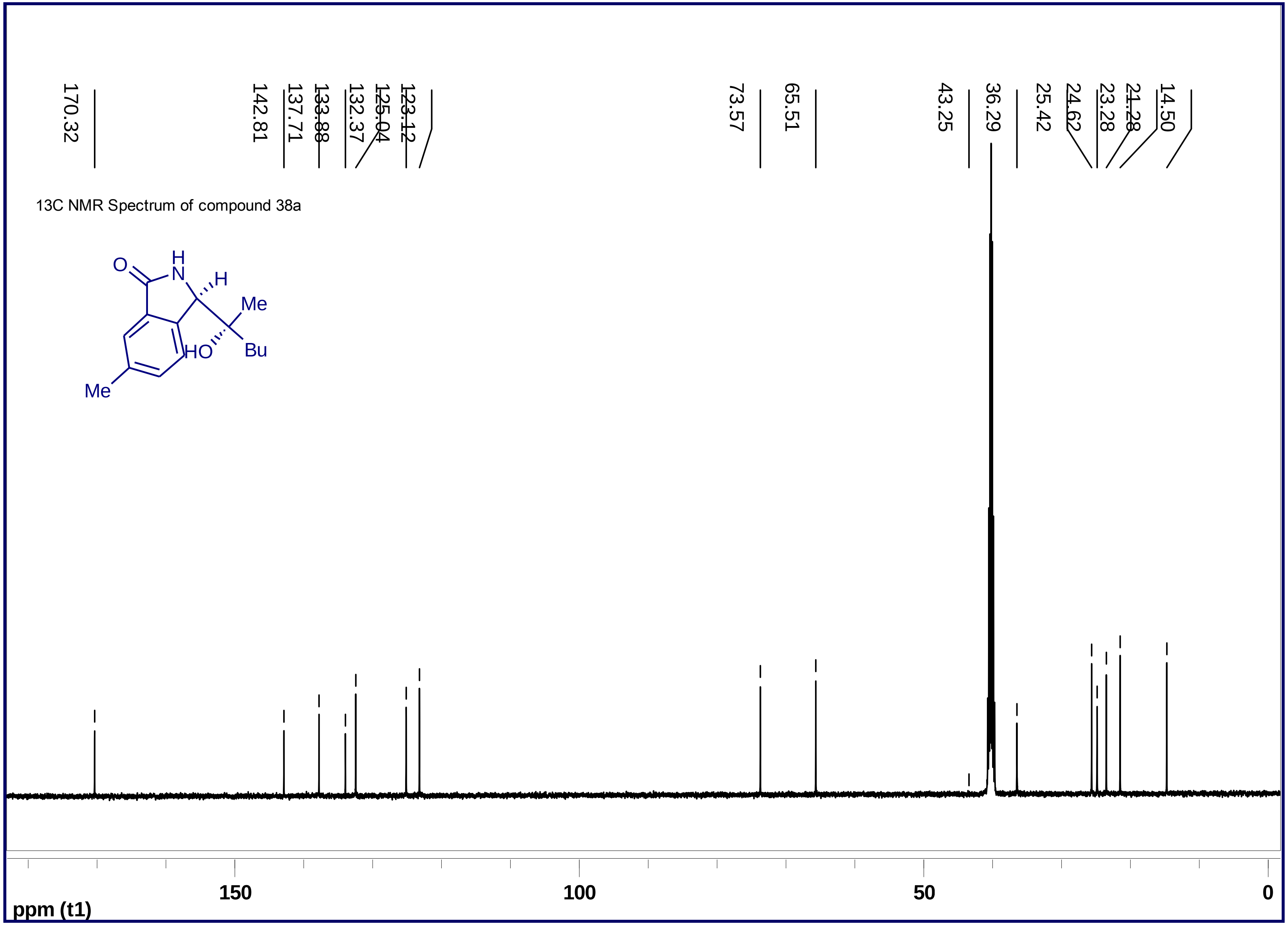

170.32
142.81
137.71
133.88
132.37
125.04
123.12
73.57
65.51
43.25
36.29
25.42
24.62
23.28
21.28
14.50
13C NMR Spectrum of compound 38a
O
H
N
H
Me
HO
Bu
Me
150
100
50
0
ppm (t1)

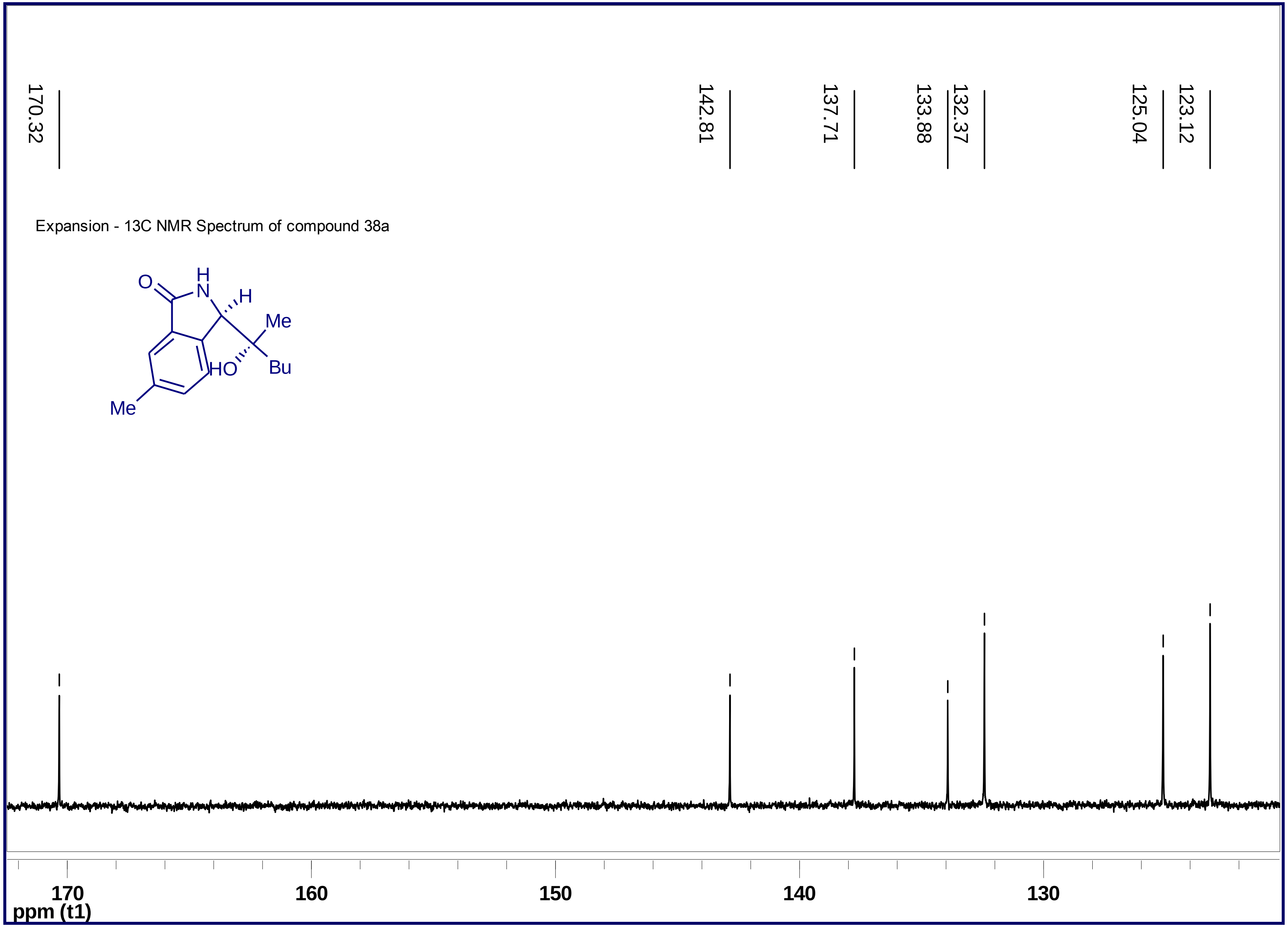
170.32
142.81
137.71
133.88
132.37
125.04
123.12
Expansion - 13C NMR Spectrum of compound 38a
O
H
N
H
Me
HO
Bu
Me
170
160
150
140
130
ppm (t1)

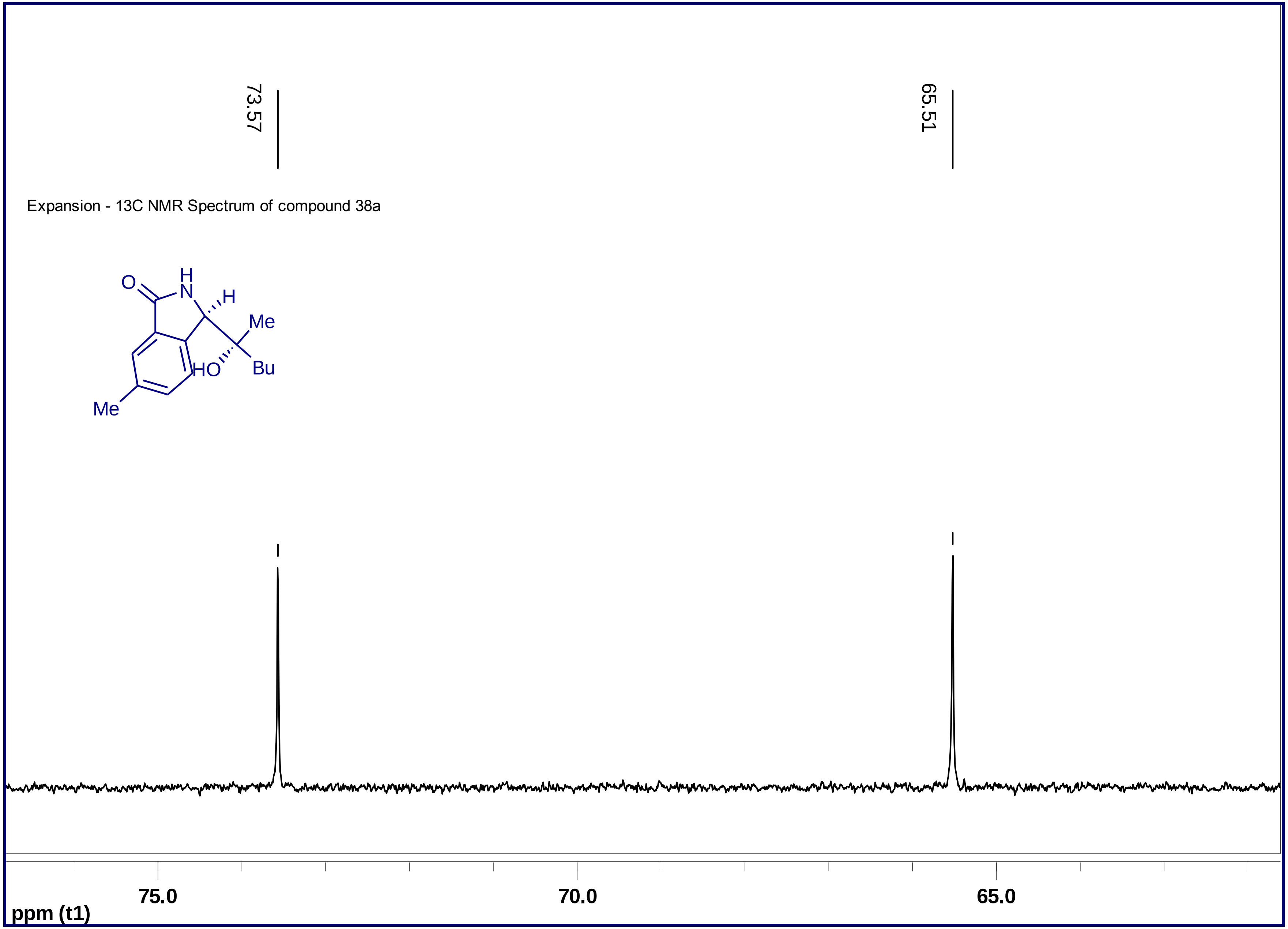

73.57
65.51
Expansion - 13C NMR Spectrum of compound 38a
O
H
N
H
Me
HO
Bu
Me
75.0
70.0
65.0
ppm (t1)

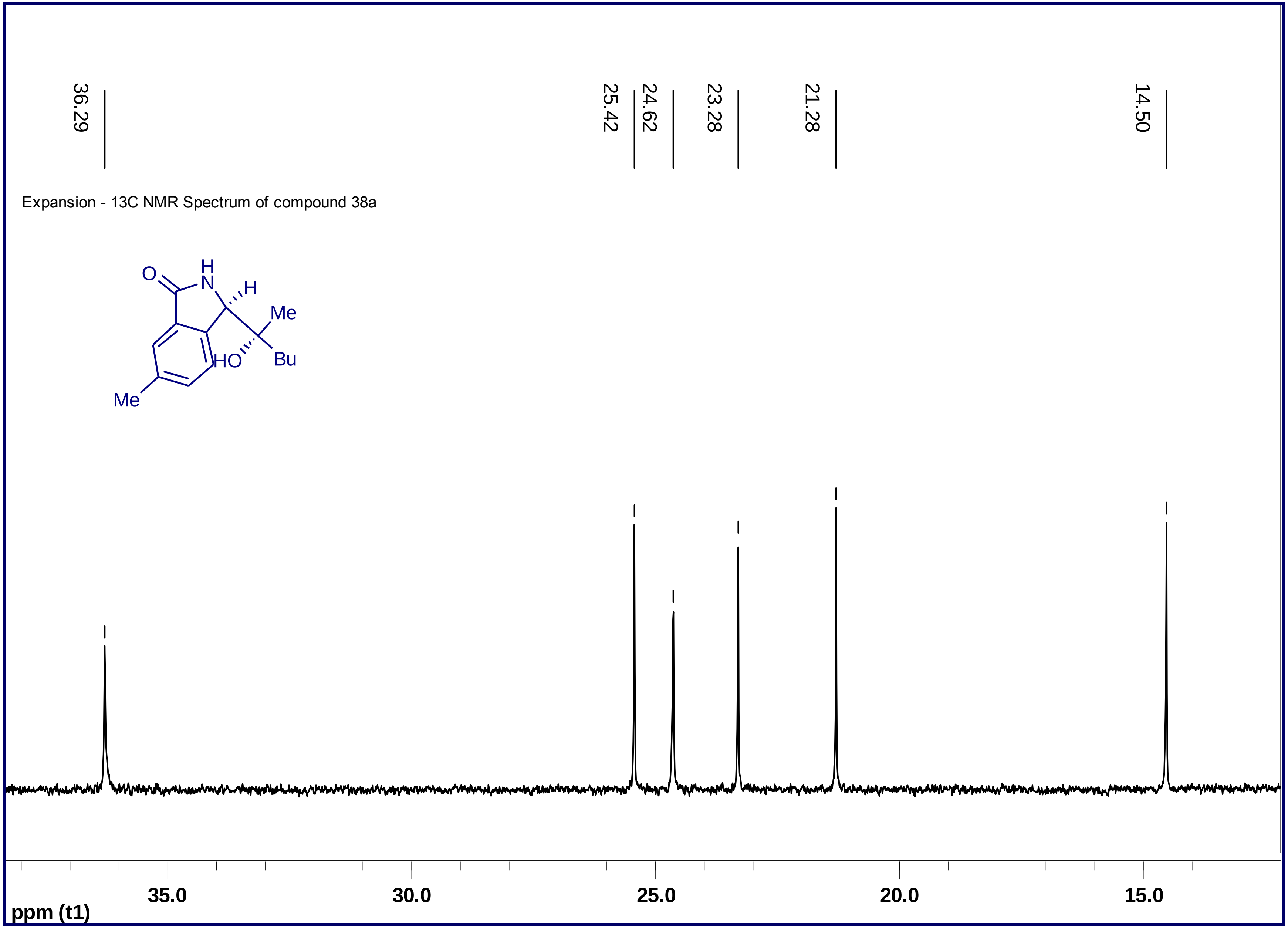
36.29
25.42
24.62
23.28
21.28
14.50
Expansion - 13C NMR Spectrum of compound 38a
O
H
N
H
Me
HO
Bu
Me
35.0
30.0
25.0
20.0
15.0
ppm (t1)

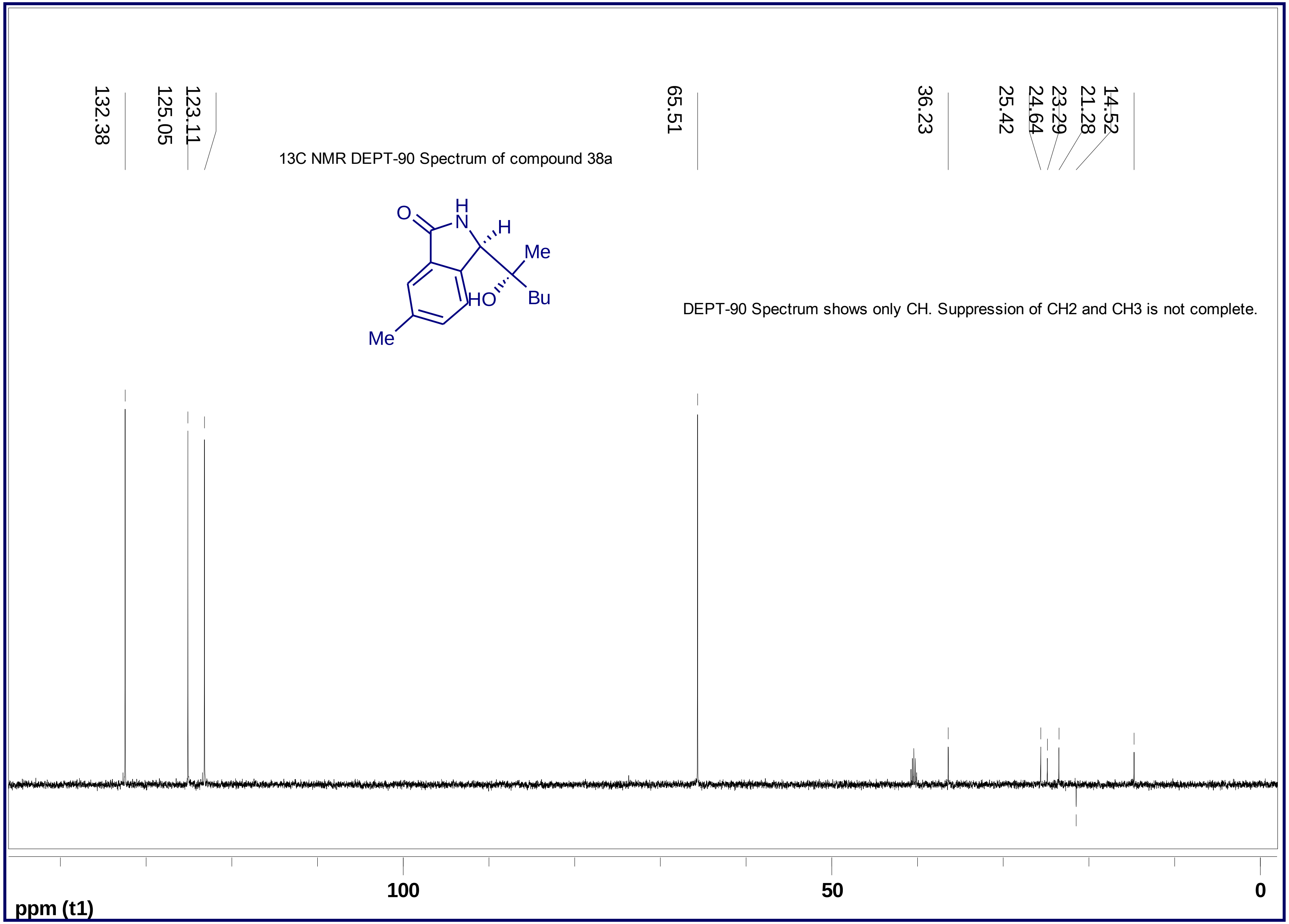
13C NMR DEPT-90 Spectrum of compound 38a
132.38
125.05
123.11
65.51
36.23
25.42
24.64
23.29
21.28
14.52
O
H
N
H
Me
HO
Bu
Me
DEPT-90 Spectrum shows only CH. Suppression of CH2 and CH3 is not complete.
100
50
0
ppm (t1)

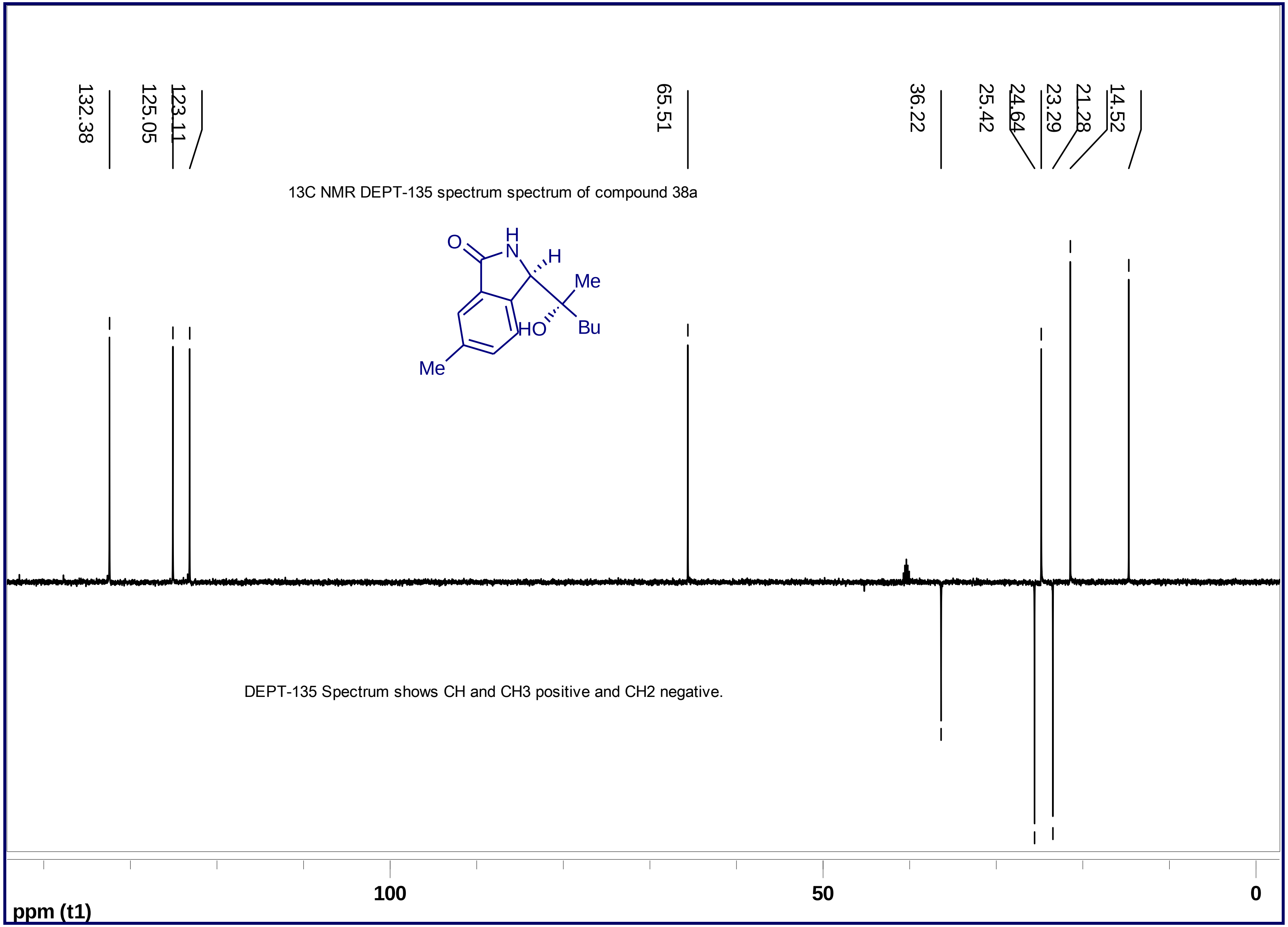
132.38
125.05
123.11
65.51
36.22
25.42
24.64
23.29
21.28
14.52
13C NMR DEPT-135 spectrum spectrum of compound 38a
O
H
N
H
Me
HO
Bu
Me
DEPT-135 Spectrum shows CH and CH3 positive and CH2 negative.
100
50
0
ppm (t1)

Expansion - 13C NMR DEPT-135 spectrum spectrum of compound 38a

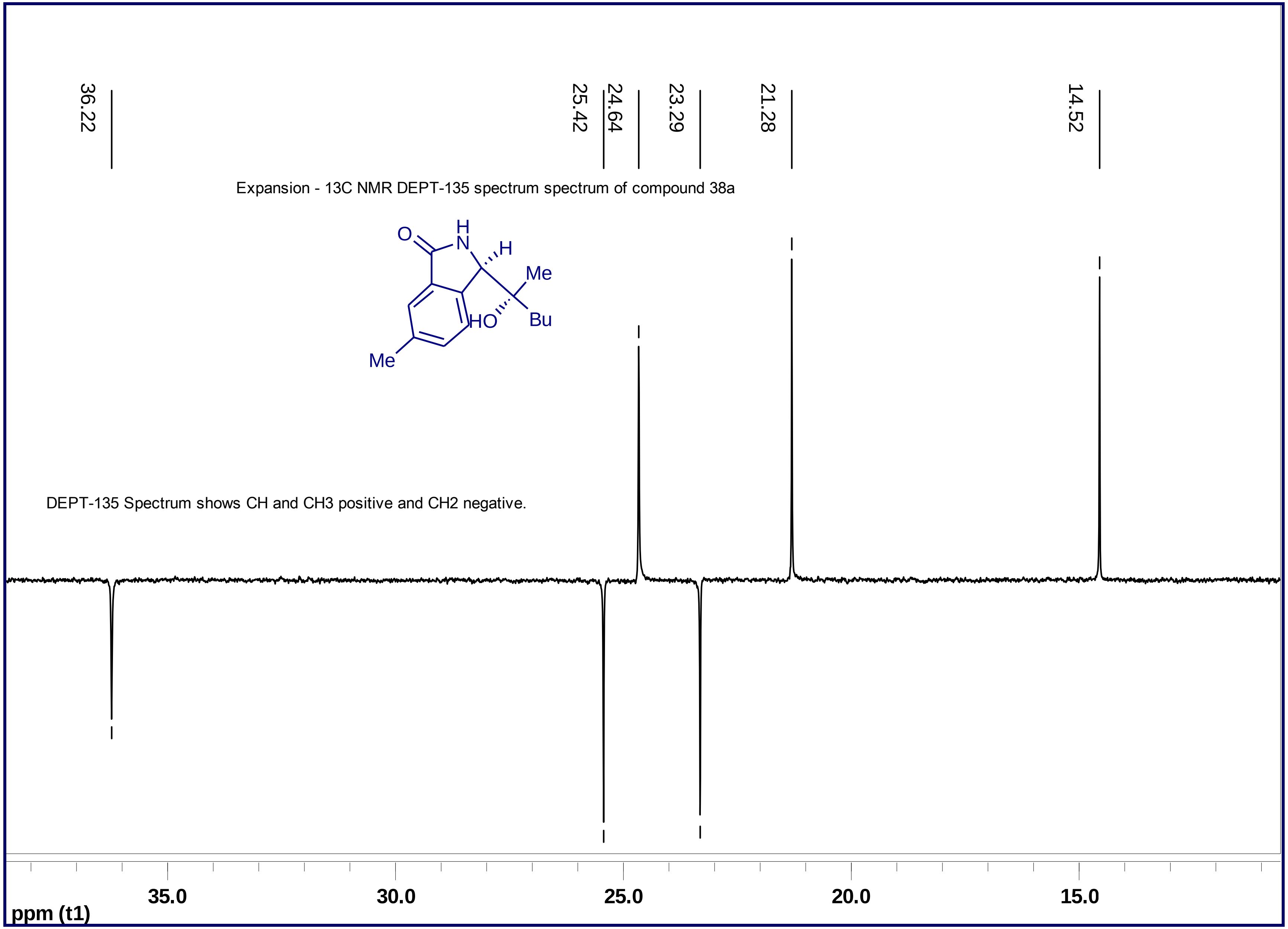

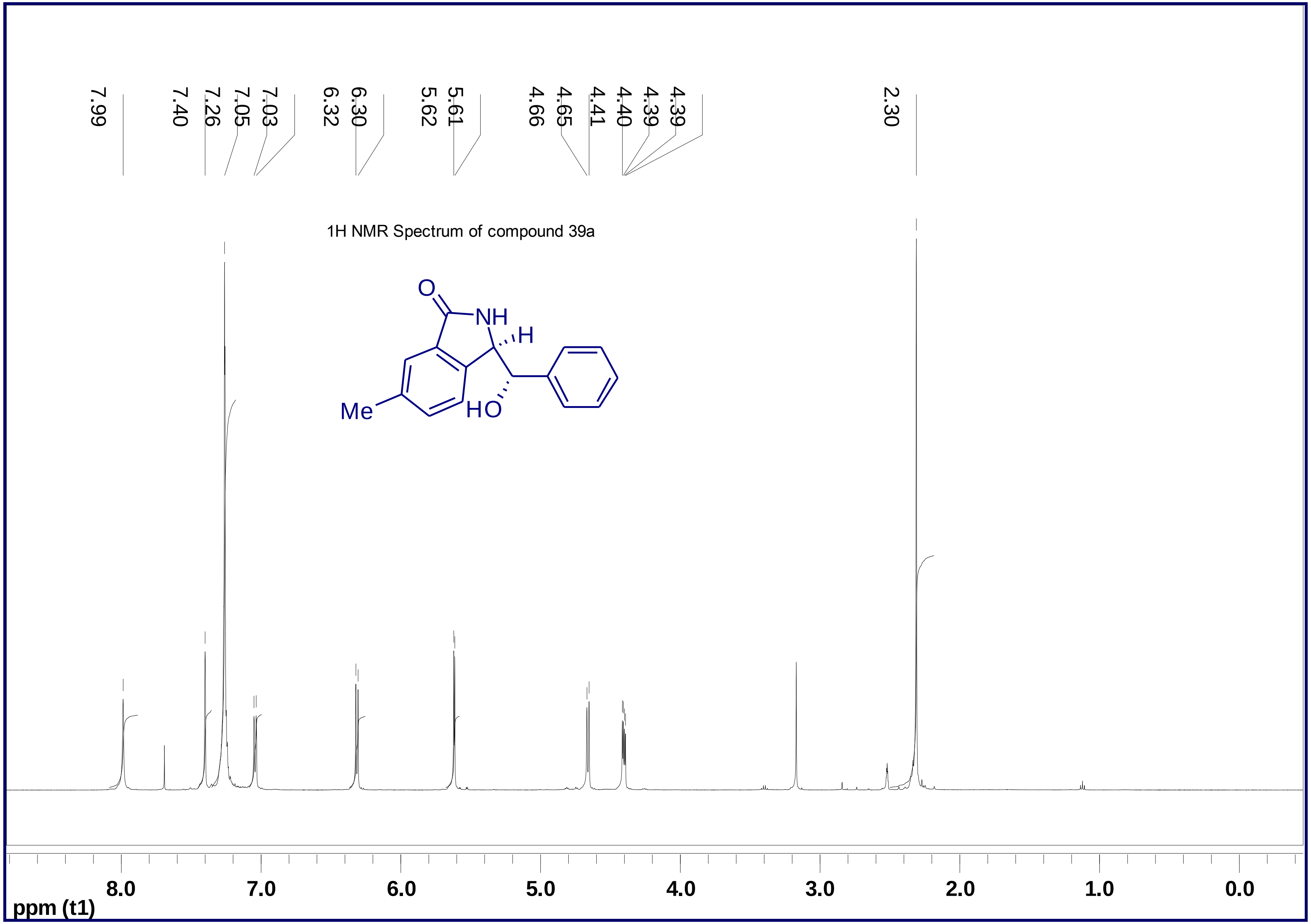
7.99
7.40
7.26
7.05
7.03
6.32
6.30
5.62
5.61
4.66
4.65
4.41
4.40
4.39
4.39
2.30
1H NMR Spectrum of compound 39a
O
NH
H
Me
HO
8.0
7.0
6.0
5.0
4.0
3.0
2.0
1.0
0.0
ppm (t1)

Expansion - 1H NMR Spectrum of compound 39a

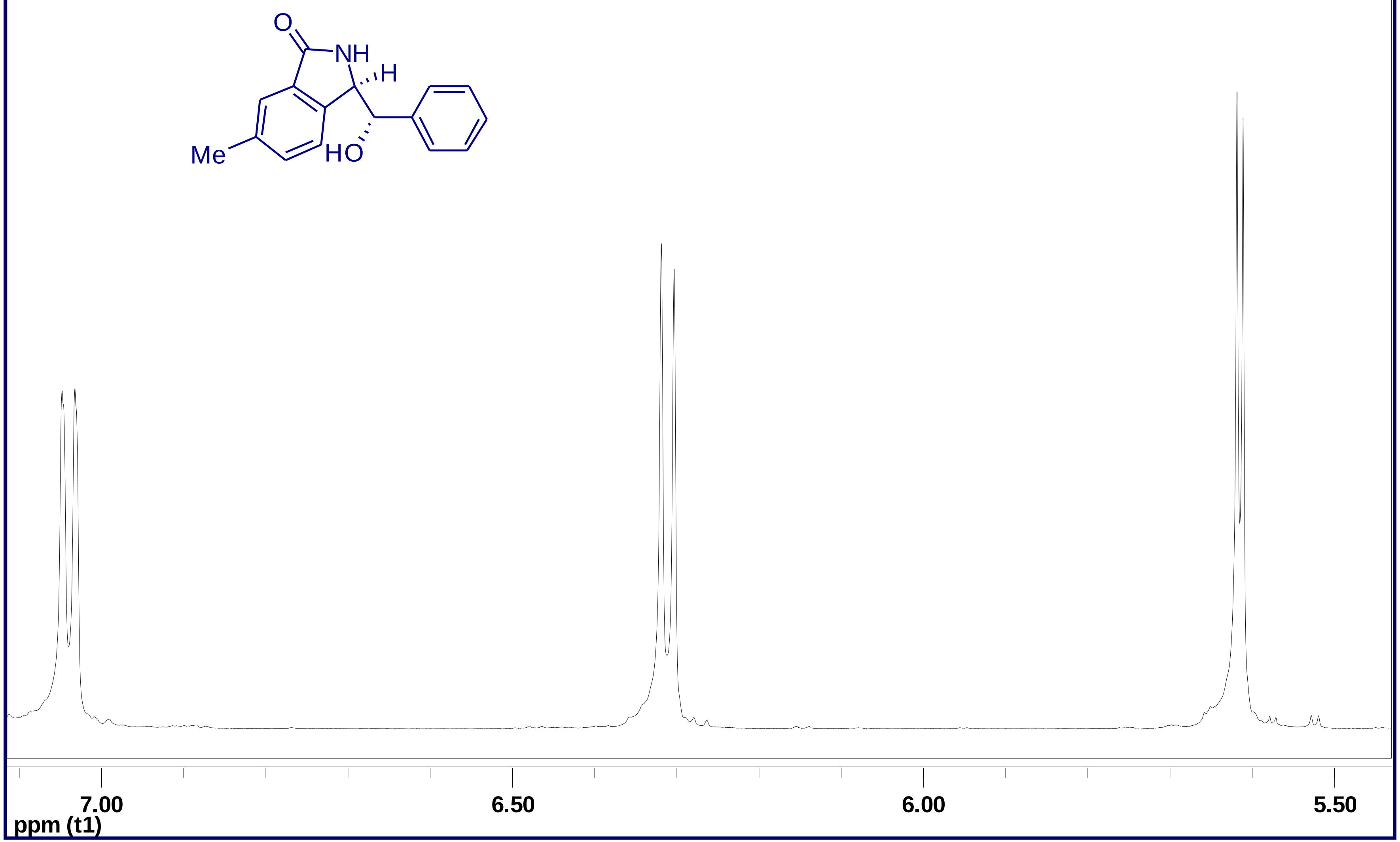

Expansion - 1H NMR Spectrum of compound 39a

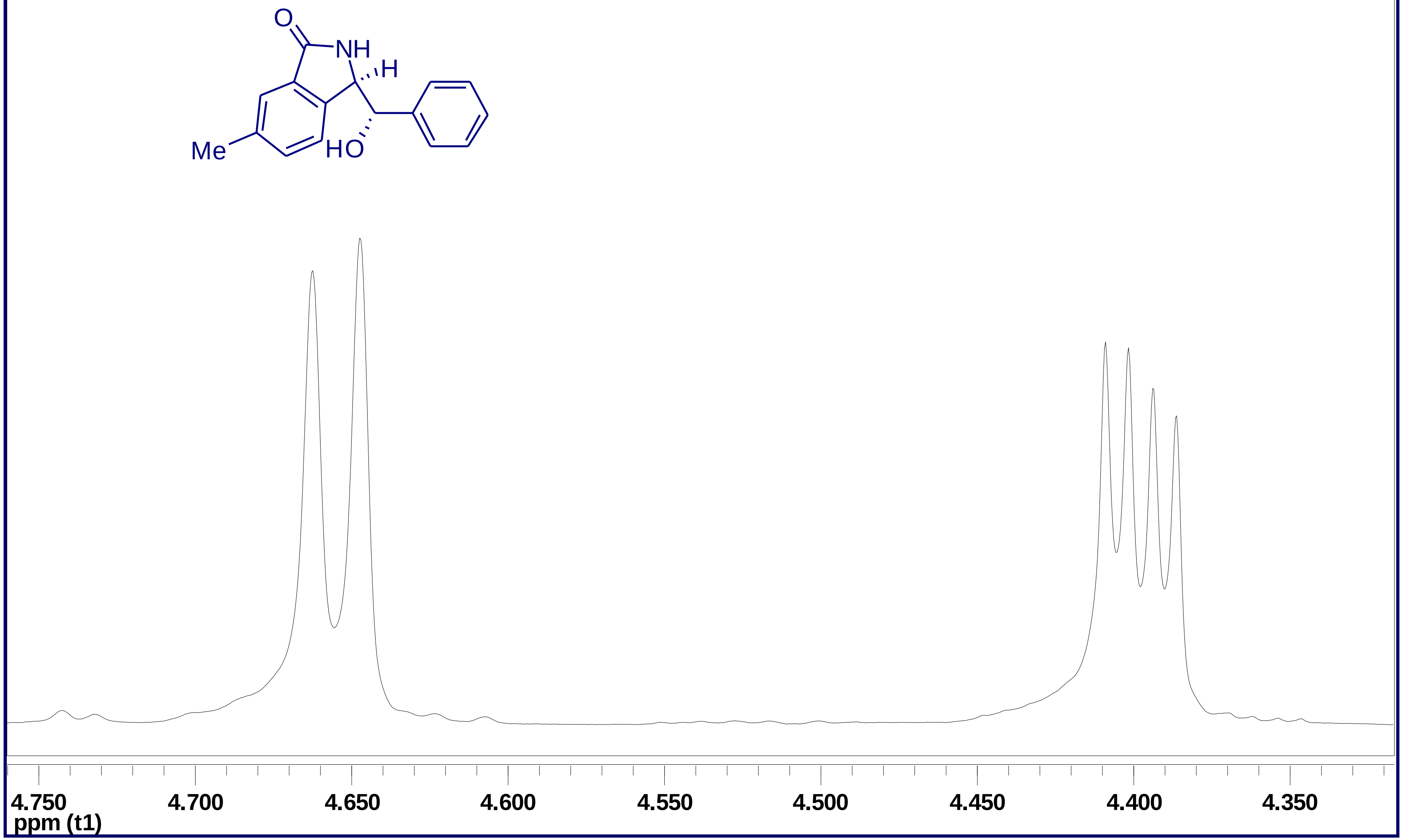

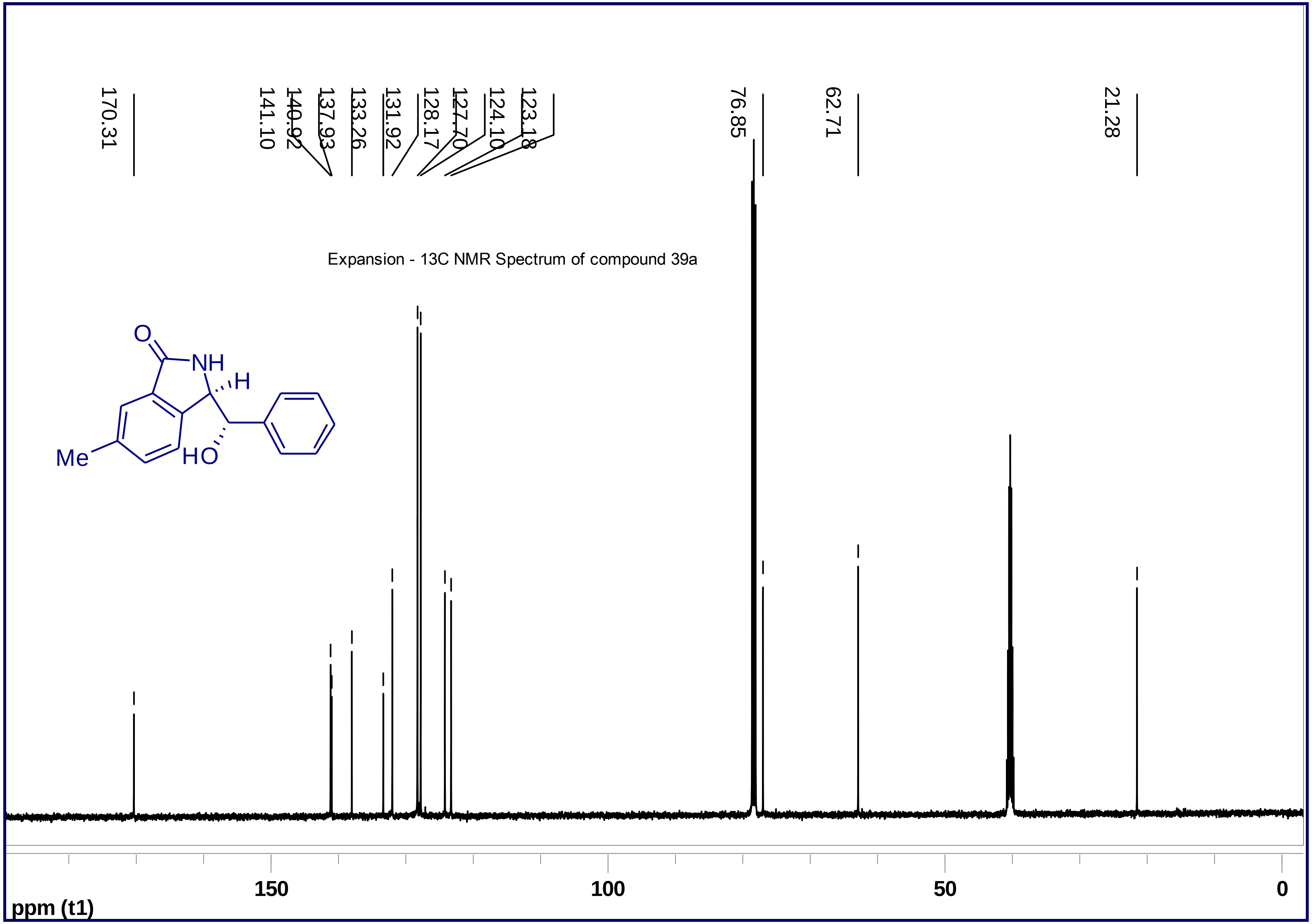

170.31
141.10
140.92
137.93
133.26
131.92
128.17
127.70
124.10
123.18
76.85
62.71
21.28
Expansion - 13C NMR Spectrum of compound 39a
O
NH
H
Me
HO
150
100
50
0
ppm (t1)

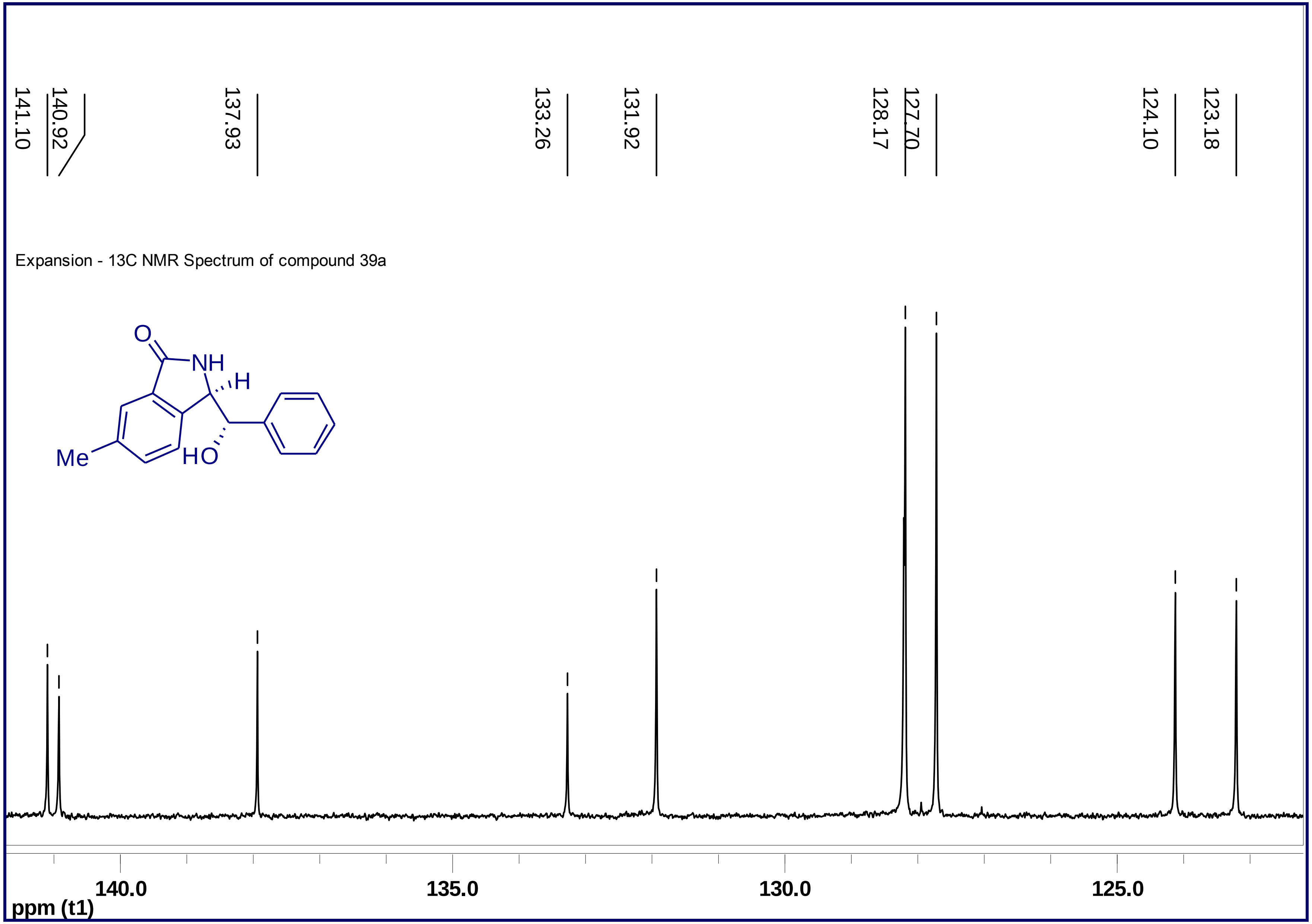

141.10
140.92
137.93
133.26
131.92
128.17
127.70
124.10
123.18
Expansion - 13C NMR Spectrum of compound 39a
O
NH
H
Me
HO
140.0
135.0
130.0
125.0
ppm (t1)

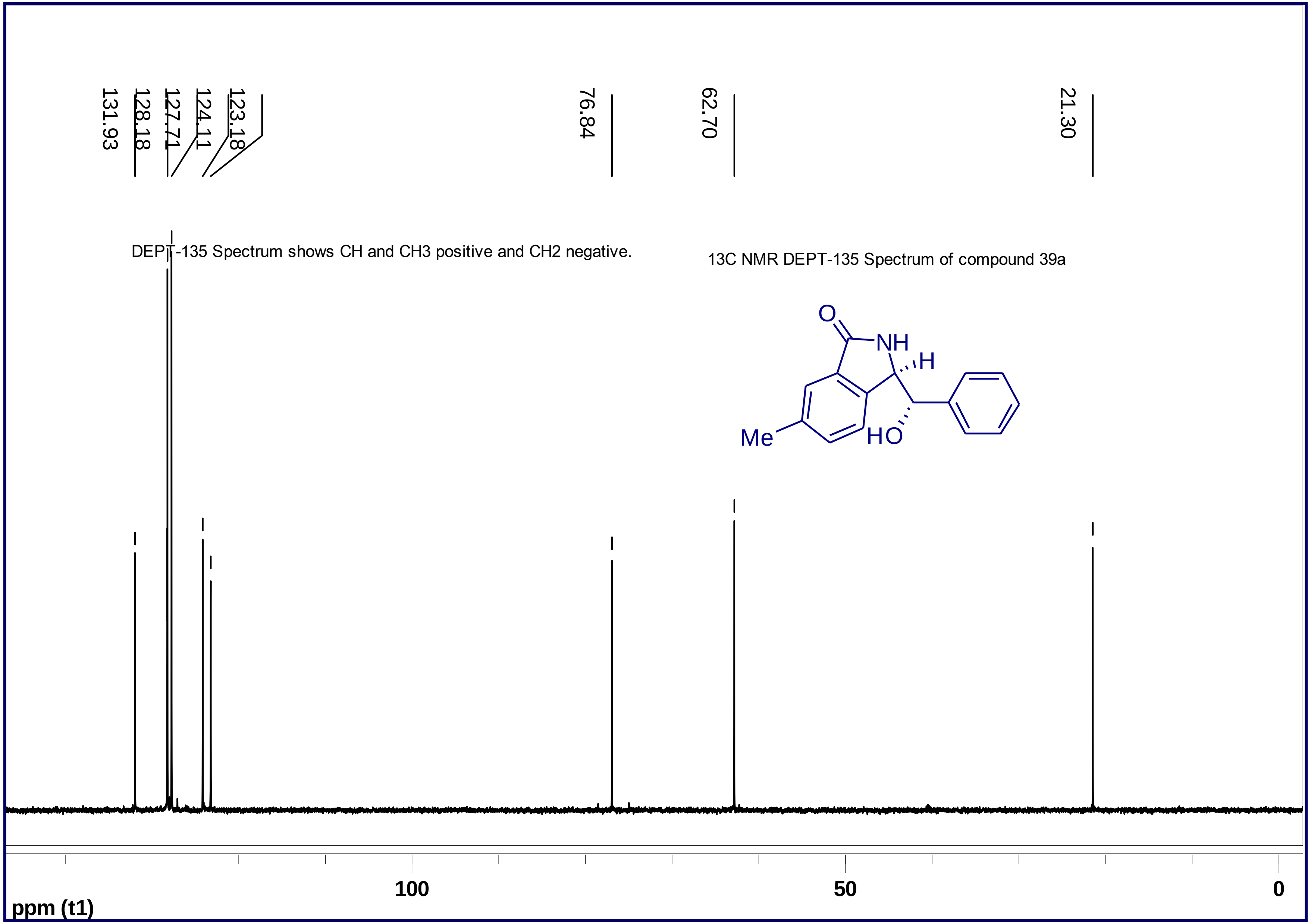

131.93
128.18
127.71
124.11
123.18
76.84
62.70
21.30
DEPT-135 Spectrum shows CH and CH3 positive and CH2 negative.
13C NMR DEPT-135 Spectrum of compound 39a
O
NH
H
Me
HO
100
50
0
ppm (t1)

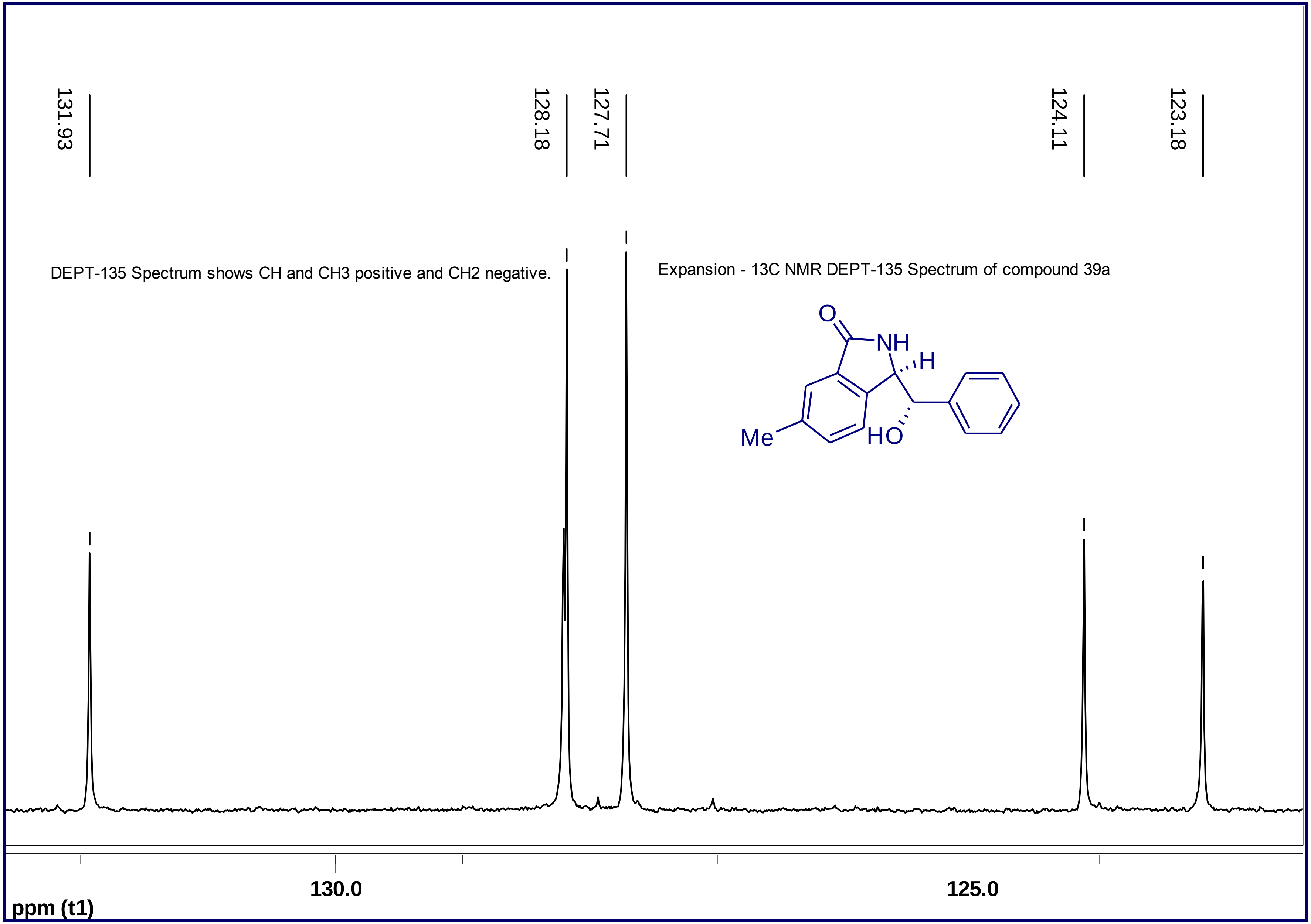
131.93
128.18
127.71
124.11
123.18
DEPT-135 Spectrum shows CH and CH3 positive and CH2 negative.
Expansion - 13C NMR DEPT-135 Spectrum of compound 39a
O
NH
H
Me
HO
130.0
125.0
ppm (t1)

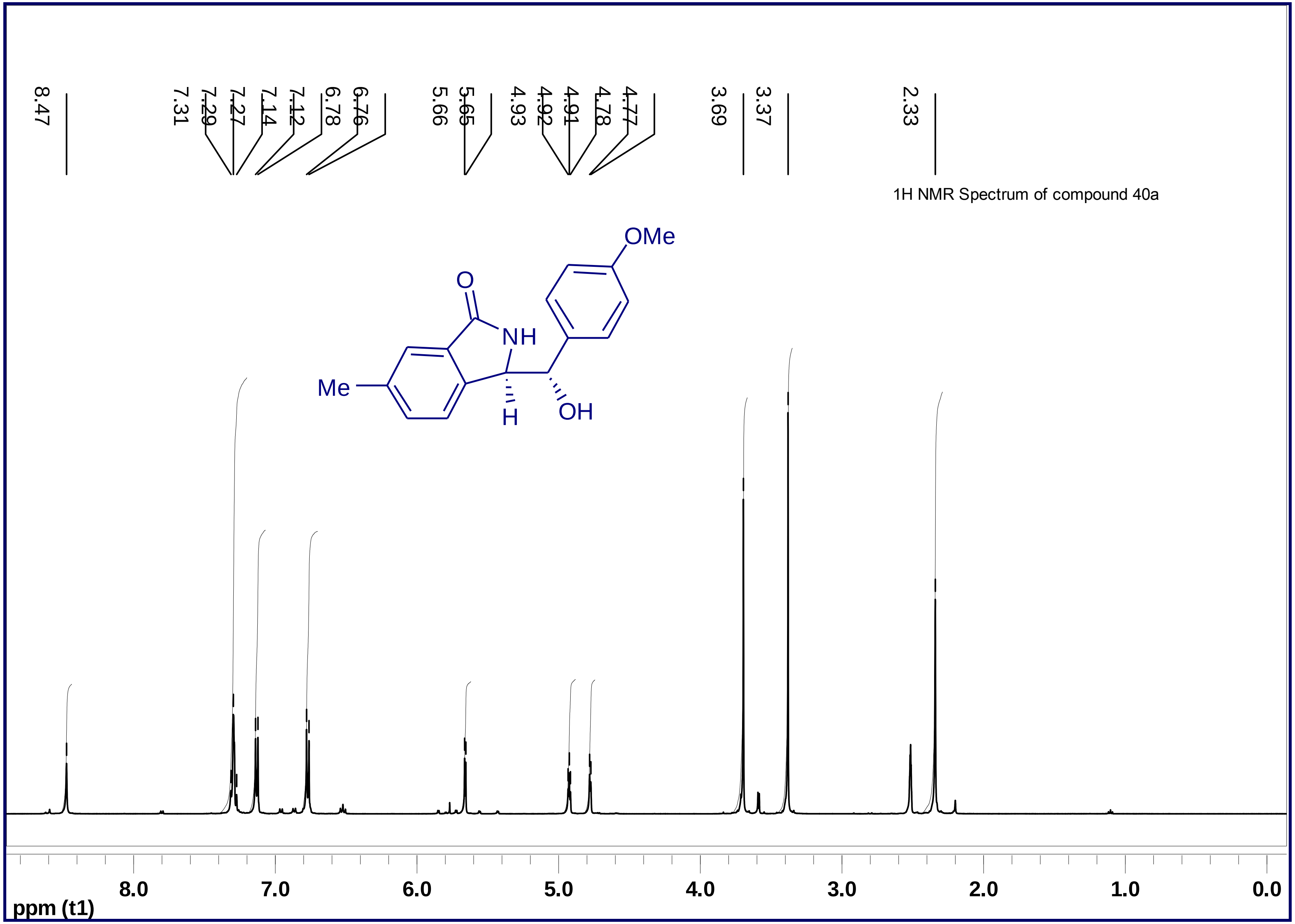
8.47
7.31
7.29
7.27
7.14
7.12
6.78
6.76
5.66
5.65
4.93
4.92
4.91
4.78
4.77
3.69
3.37
2.33
1H NMR Spectrum of compound 40a
OMe
O
NH
Me
H
OH
8.0
7.0
6.0
5.0
4.0
3.0
2.0
1.0
0.0
ppm (t1)

Expansion - 1H NMR Spectrum of compound 40a

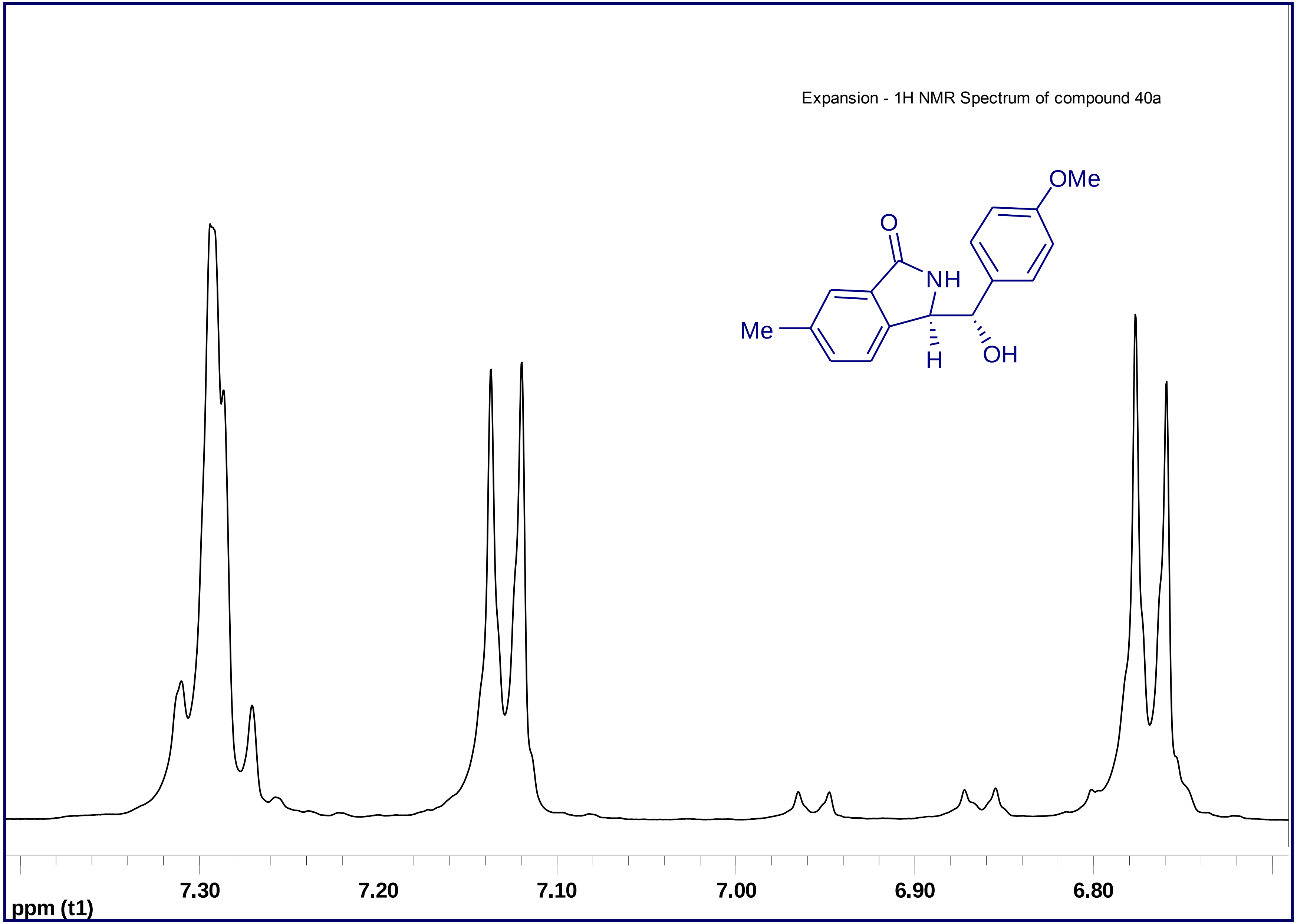

Expansion - 1H NMR Spectrum of compound 40a

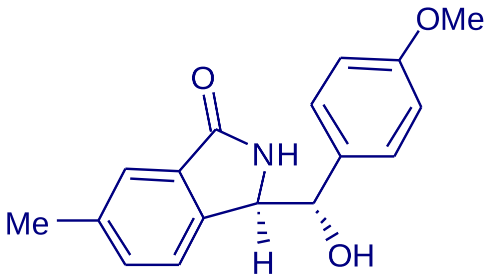

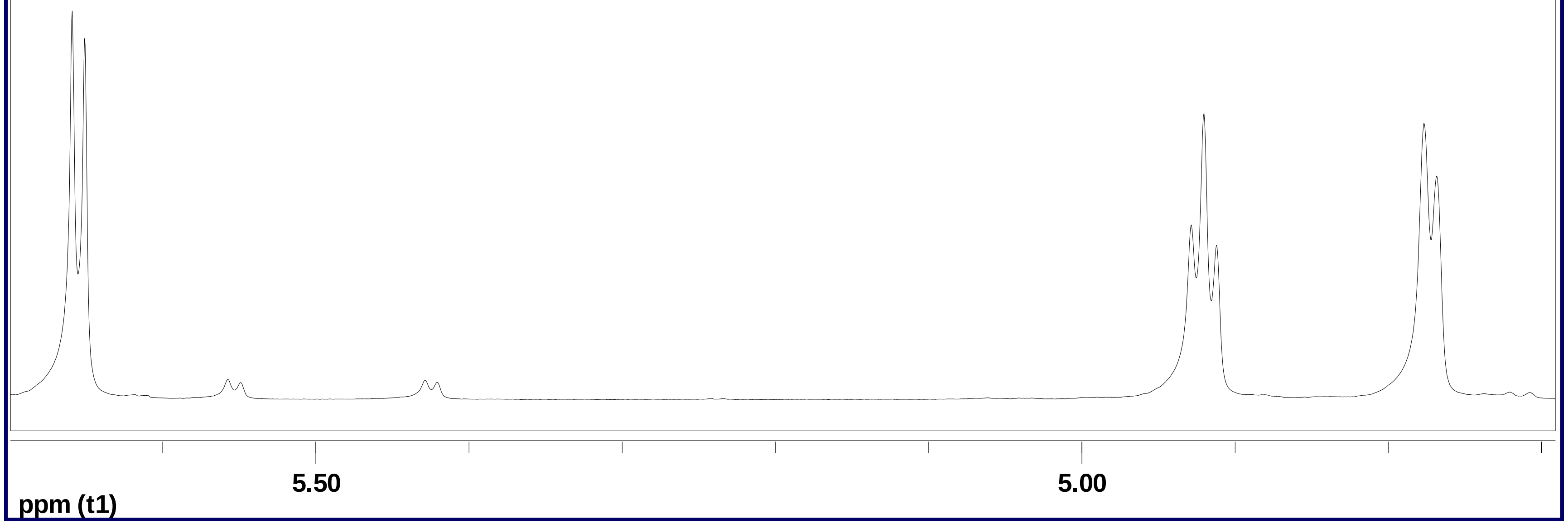

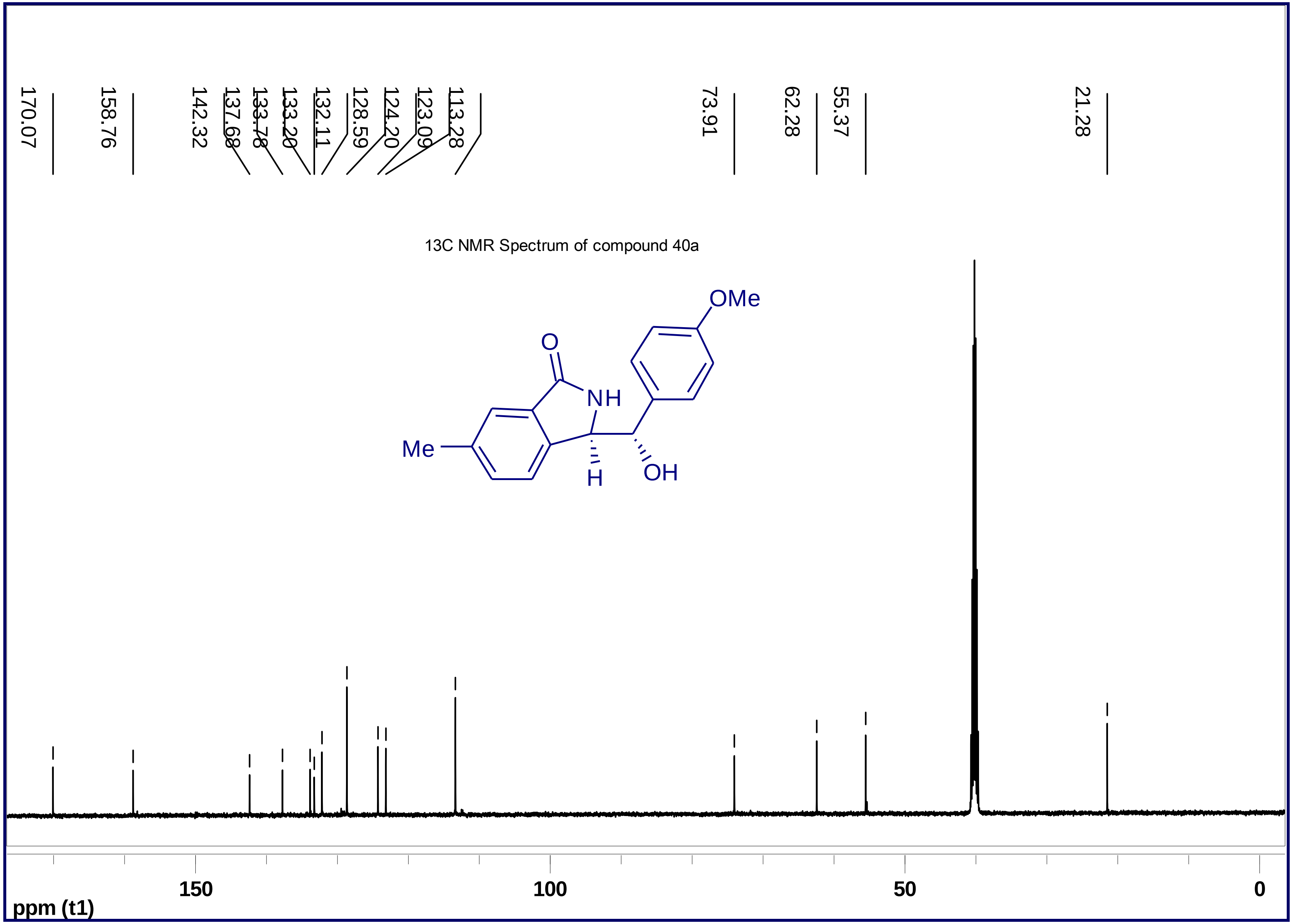
13C NMR Spectrum of compound 40a
170.07
158.76
142.32
137.68
133.78
133.20
132.11
128.59
124.20
123.09
113.28
73.91
62.28
55.37
21.28
OMe
O
NH
Me
H
OH
150
100
50
0
ppm (t1)

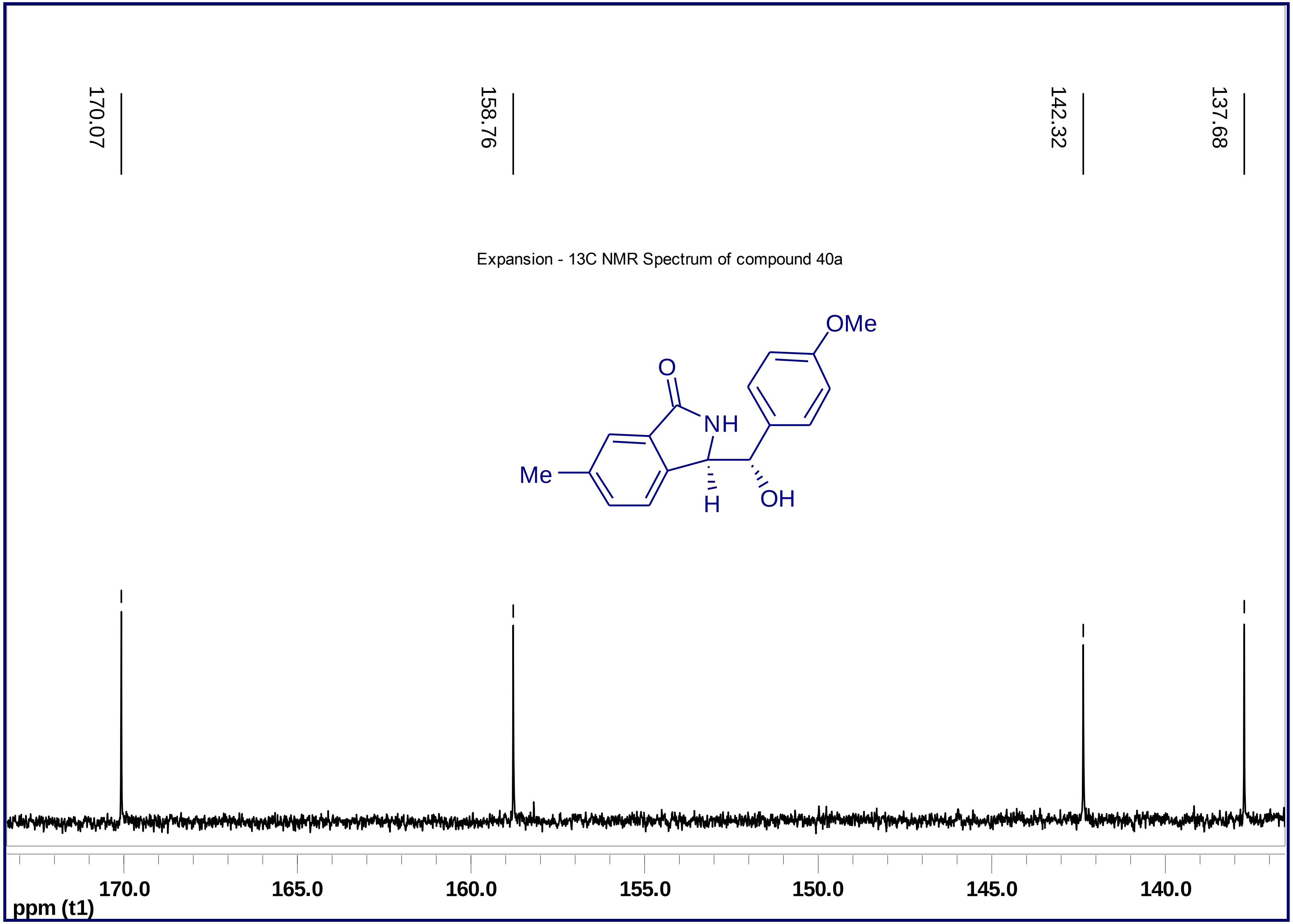
170.07
158.76
142.32
137.68
Expansion - 13C NMR Spectrum of compound 40a
OMe
O
NH
Me
H
OH
170.0
165.0
160.0
155.0
150.0
145.0
140.0
ppm (t1)

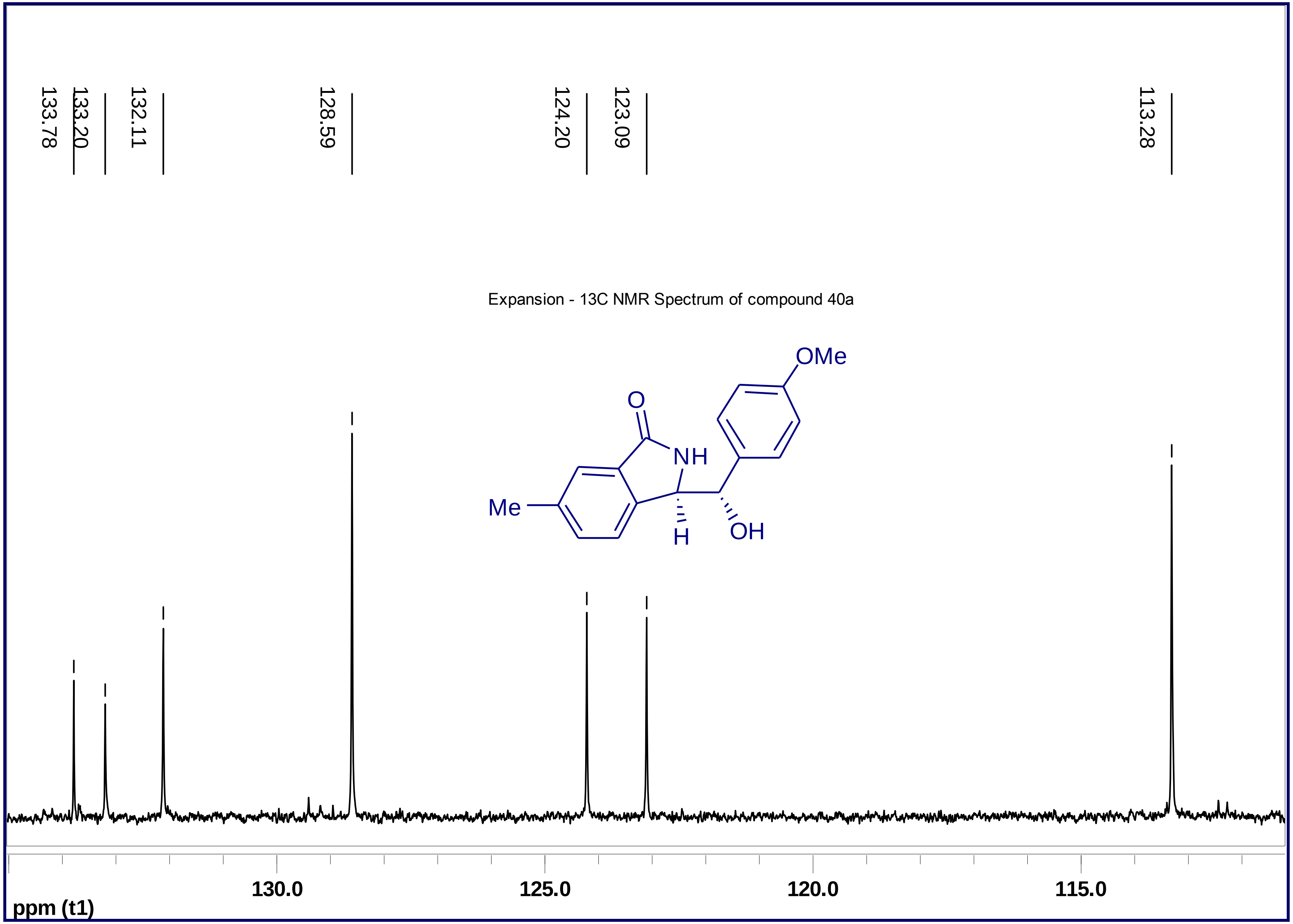
133.78
133.20
132.11
128.59
124.20
123.09
113.28
Expansion - 13C NMR Spectrum of compound 40a
OMe
O
NH
Me
H
OH
130.0
125.0
120.0
115.0
ppm (t1)

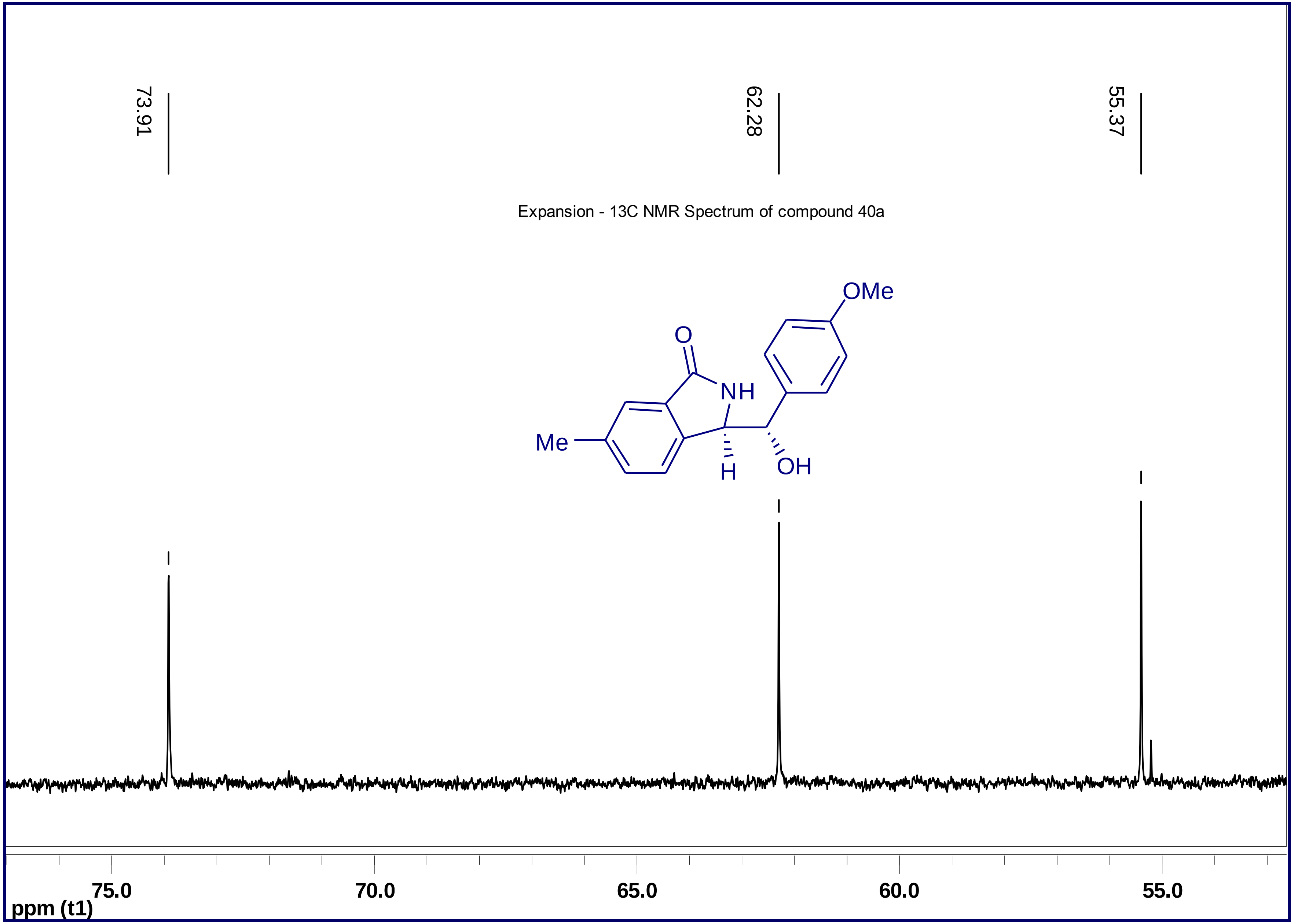
73.91
62.28
55.37
Expansion - 13C NMR Spectrum of compound 40a
OMe
O
NH
Me
H
OH
75.0
70.0
65.0
60.0
55.0
ppm (t1)

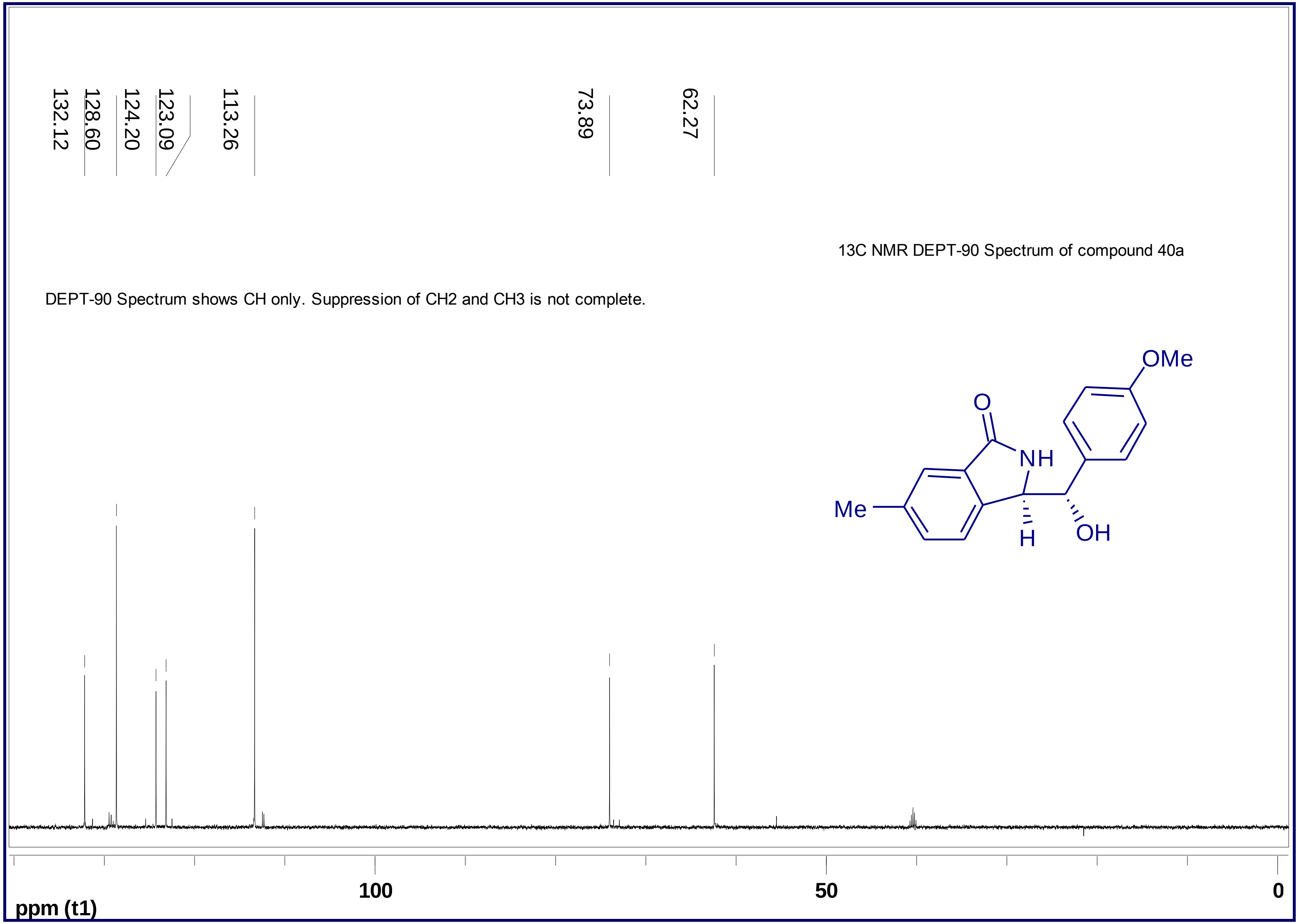

132.12
128.60
124.20
123.09
113.26
73.89
62.27
13C NMR DEPT-90 Spectrum of compound 40a
DEPT-90 Spectrum shows CH only. Suppression of CH2 and CH3 is not complete.
OMe
O
NH
Me
H
OH
100
50
0
ppm (t1)

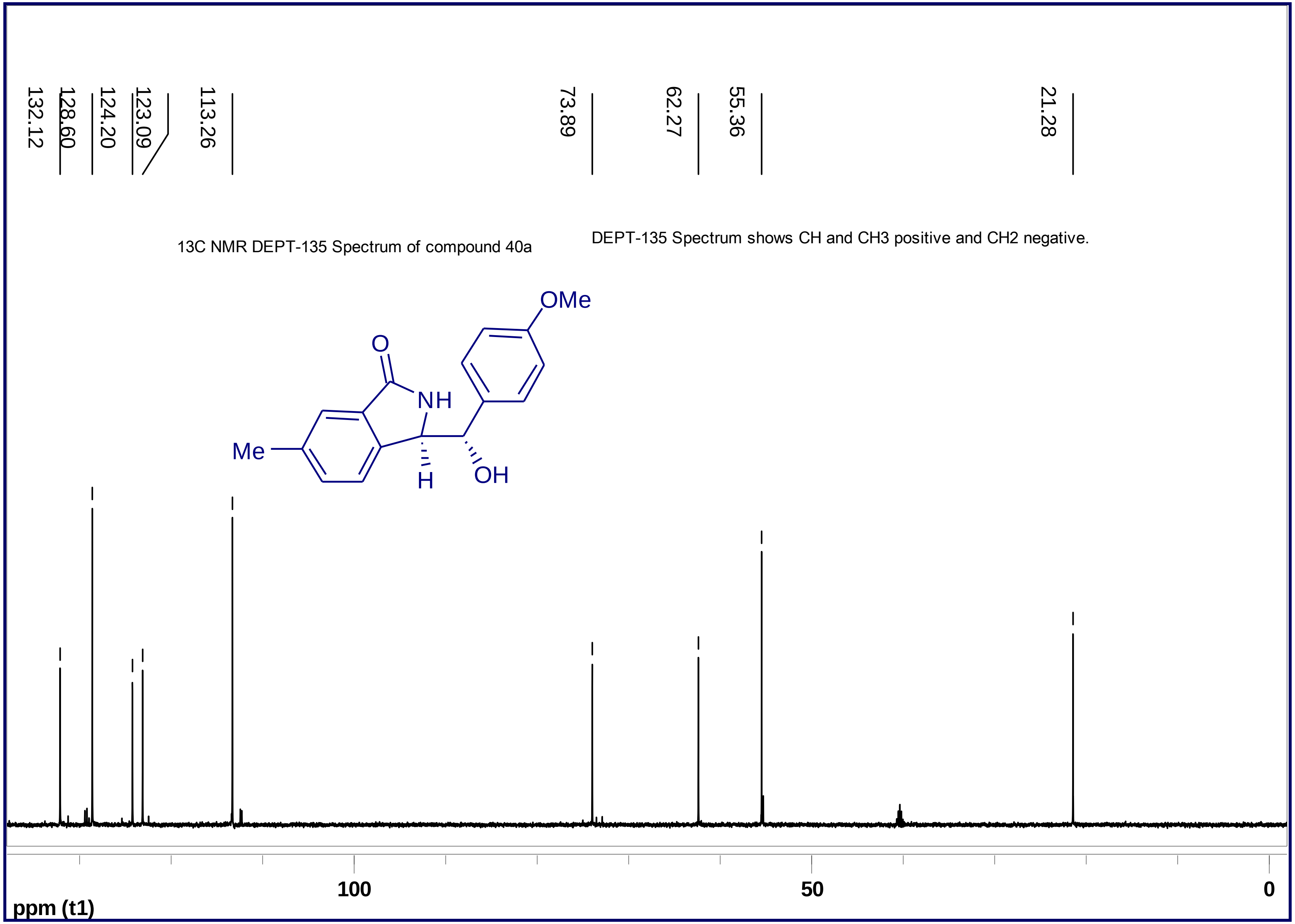
132.12
128.60
124.20
123.09
113.26
73.89
62.27
55.36
21.28
13C NMR DEPT-135 Spectrum of compound 40a
DEPT-135 Spectrum shows CH and CH3 positive and CH2 negative.
OMe
O
NH
Me
H
OH
100
50
0
ppm (t1)

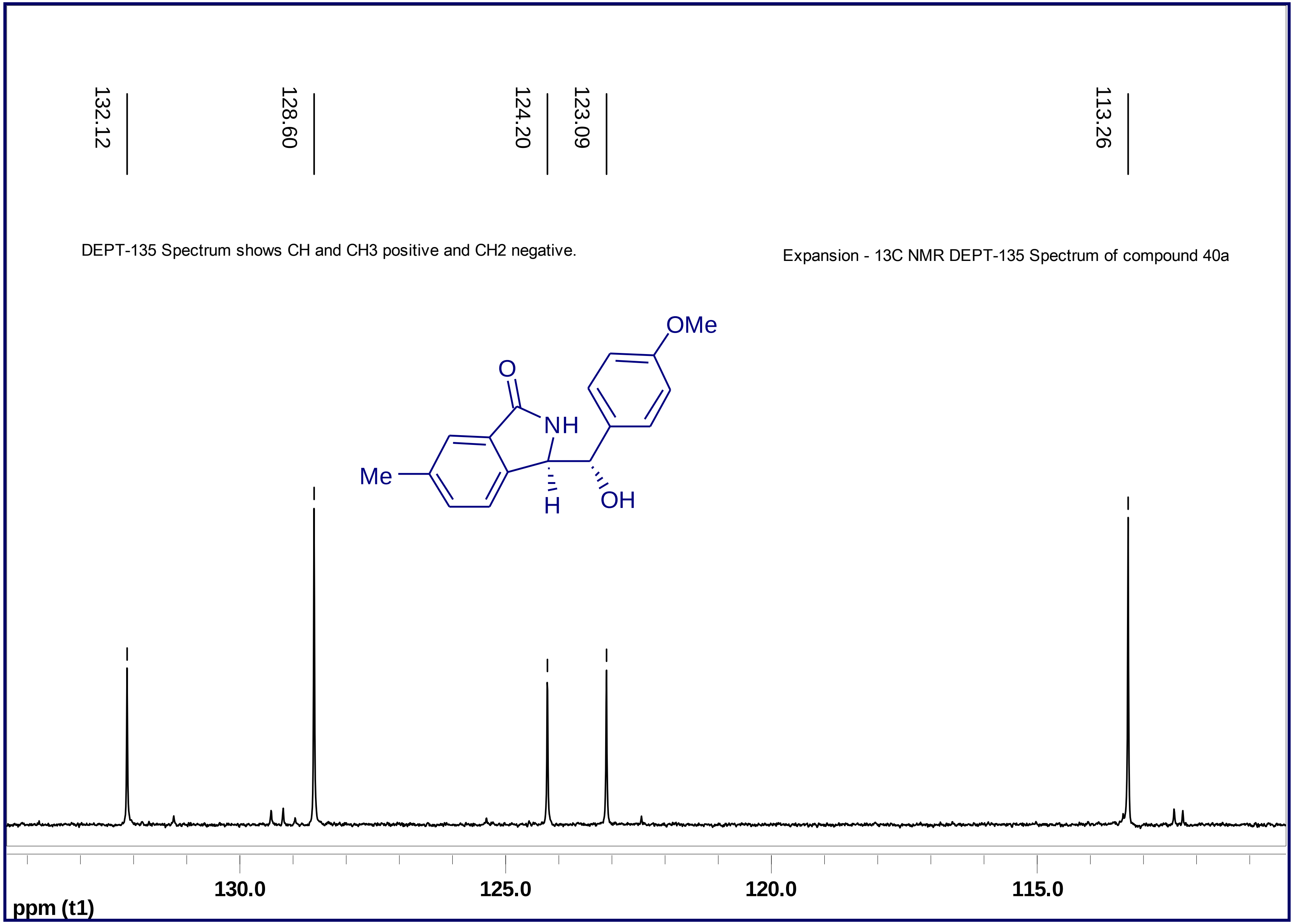

132.12
128.60
124.20
123.09
113.26
DEPT-135 Spectrum shows CH and CH3 positive and CH2 negative.
Expansion - 13C NMR DEPT-135 Spectrum of compound 40a
OMe
O
NH
Me
H
OH
130.0
125.0
120.0
115.0
ppm (t1)

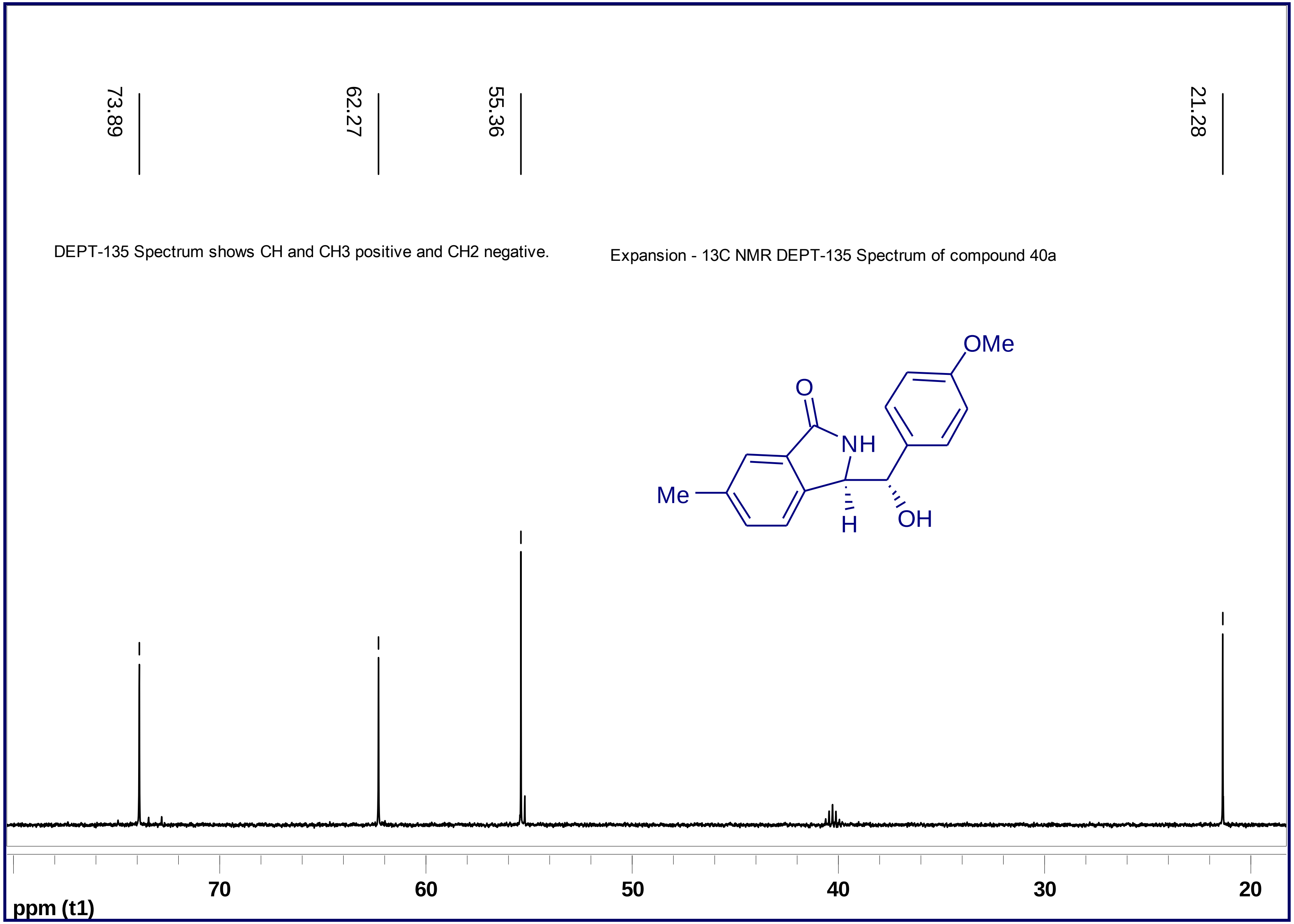

73.89
62.27
55.36
21.28
DEPT-135 Spectrum shows CH and CH3 positive and CH2 negative.
Expansion - 13C NMR DEPT-135 Spectrum of compound 40a
OMe
O
NH
Me
H
OH
70
60
50
40
30
20
ppm (t1)

1H NMR Spectrum of compound 41

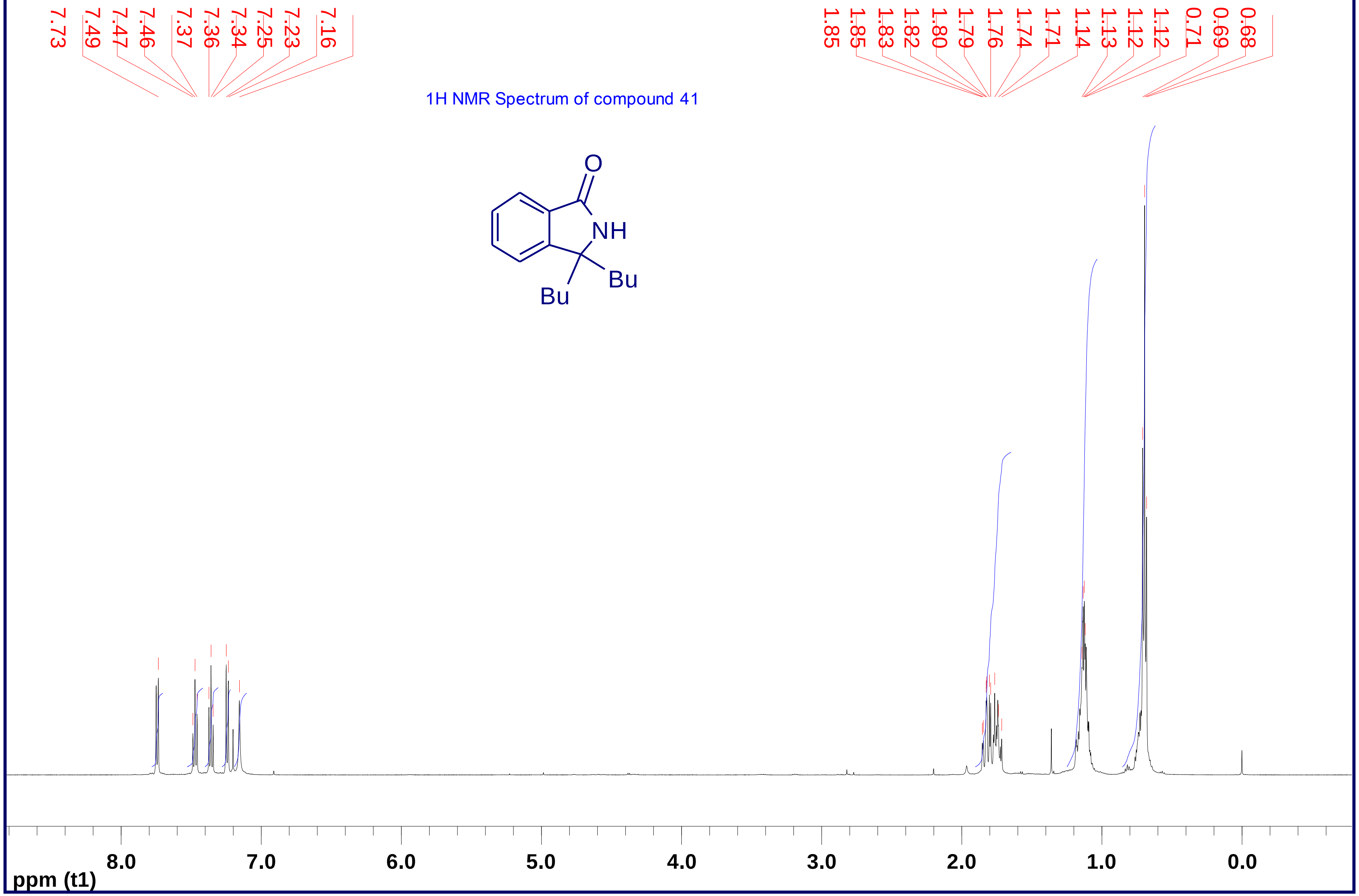

Expansion - 1H NMR Spectrum of compound 41

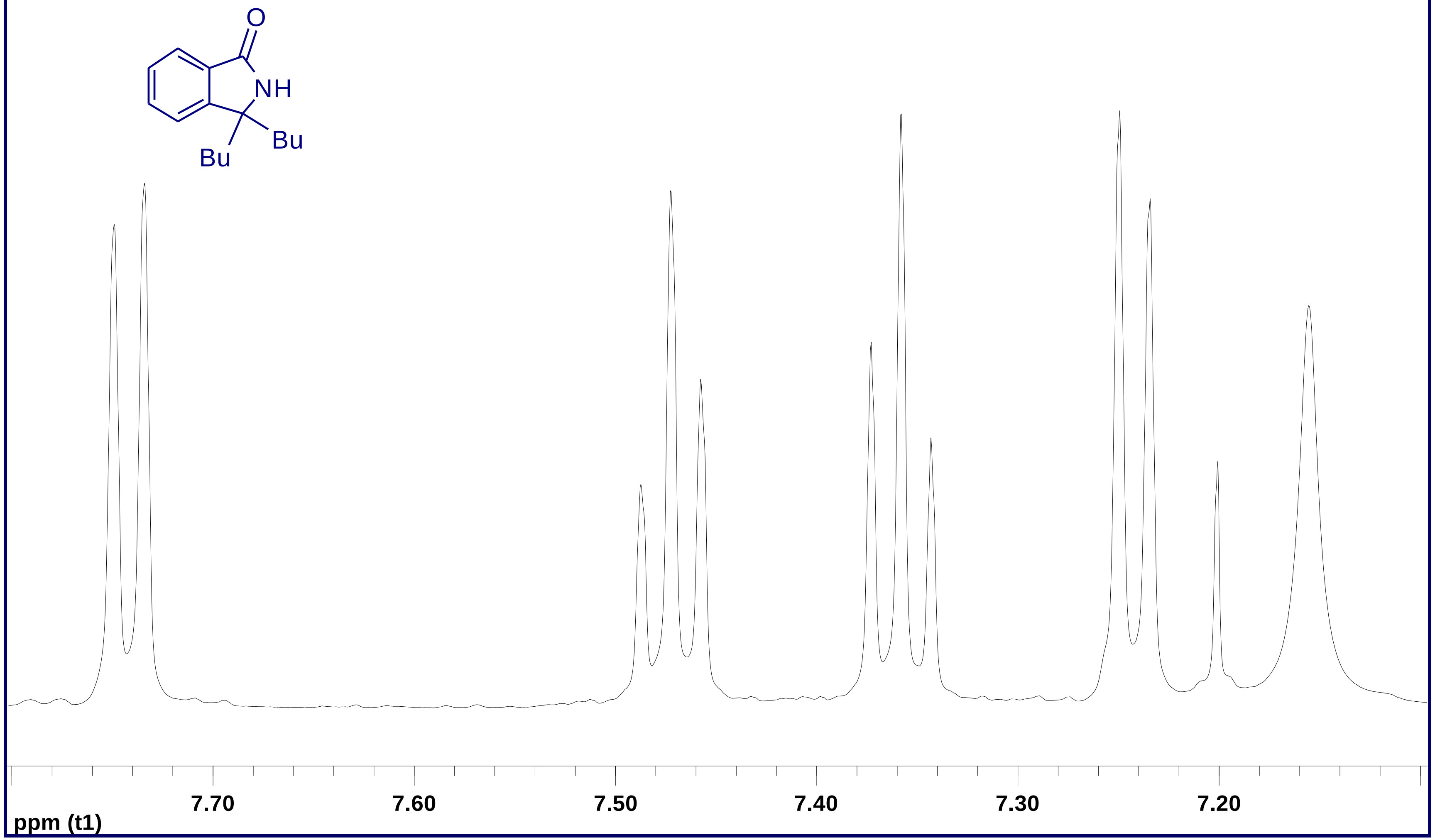

Expansion - 1H NMR Spectrum of compound 41

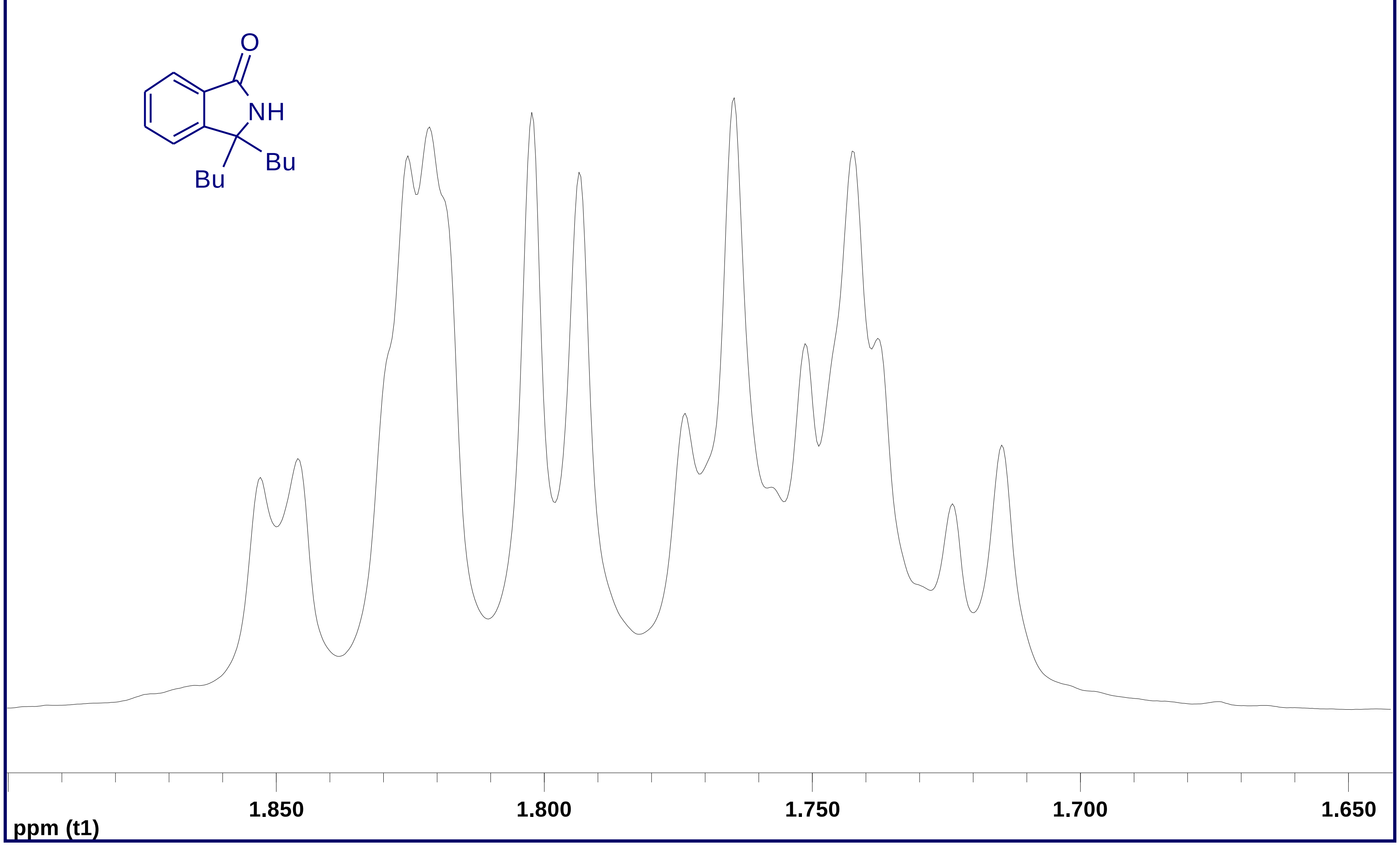

Expansion - 1H NMR Spectrum of compound 41

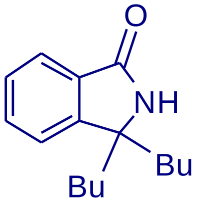

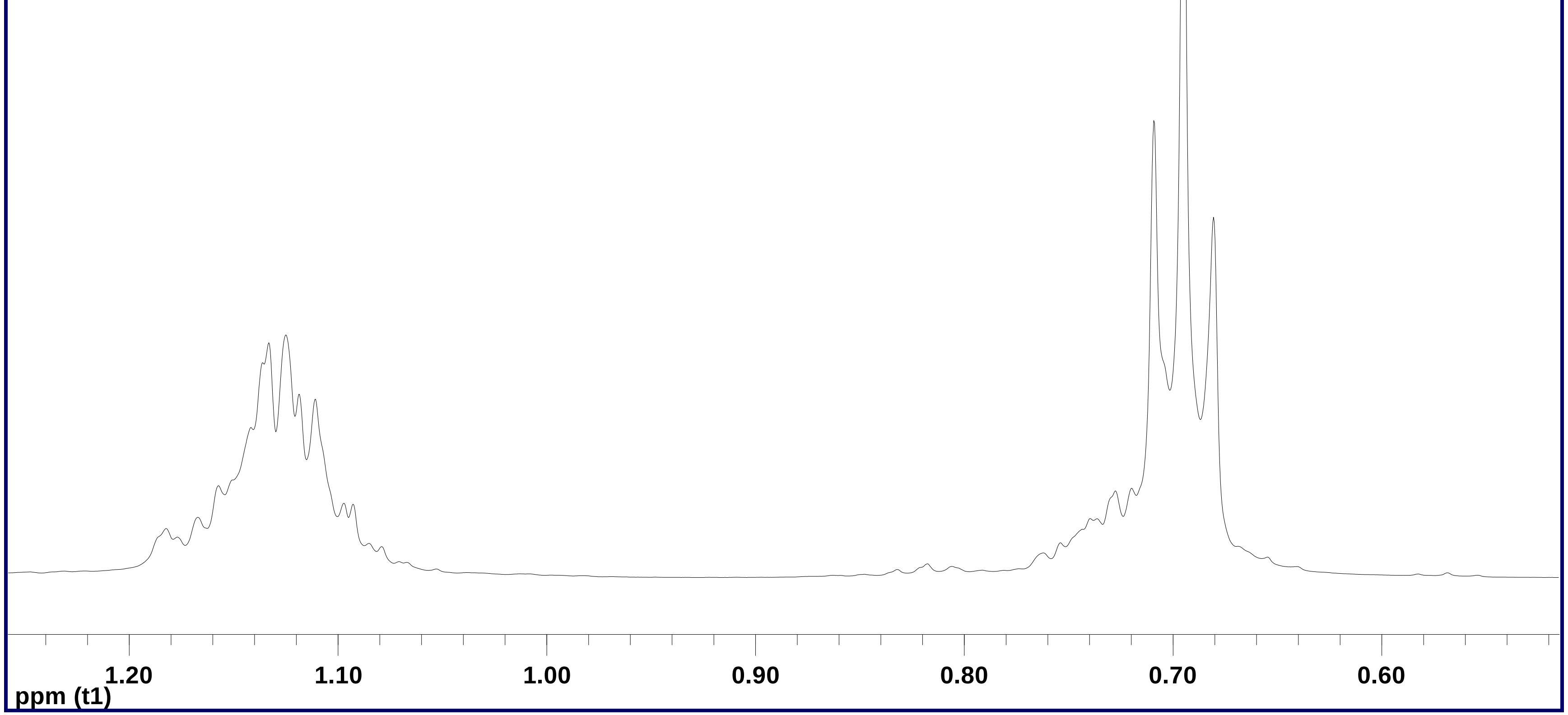

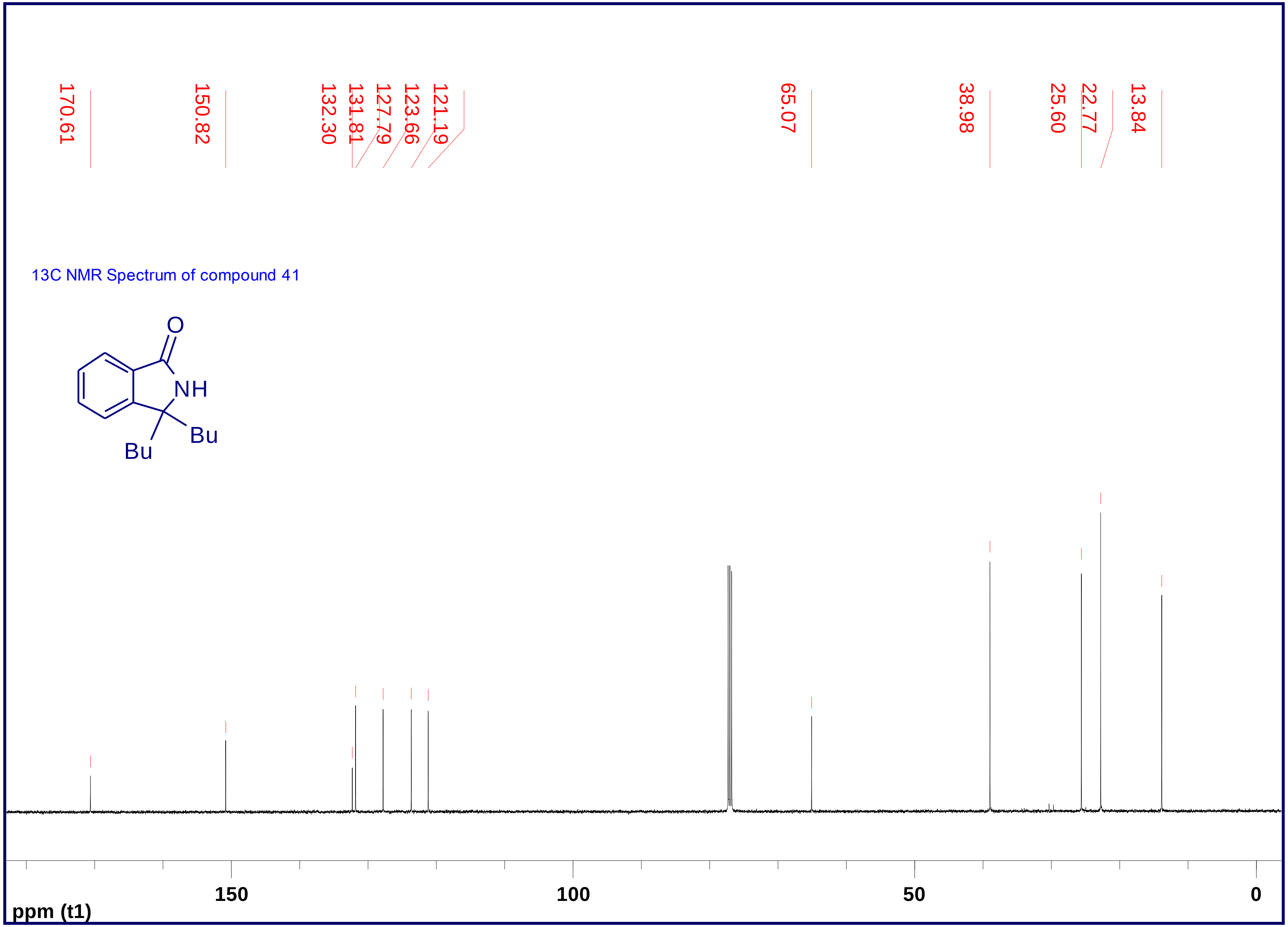
170.61
150.82
132.30
131.81
127.79
123.66
121.19
65.07
38.98
25.60
22.77
13.84
13C NMR Spectrum of compound 41
O
NH
Bu
Bu
150
100
50
0
ppm (t1)

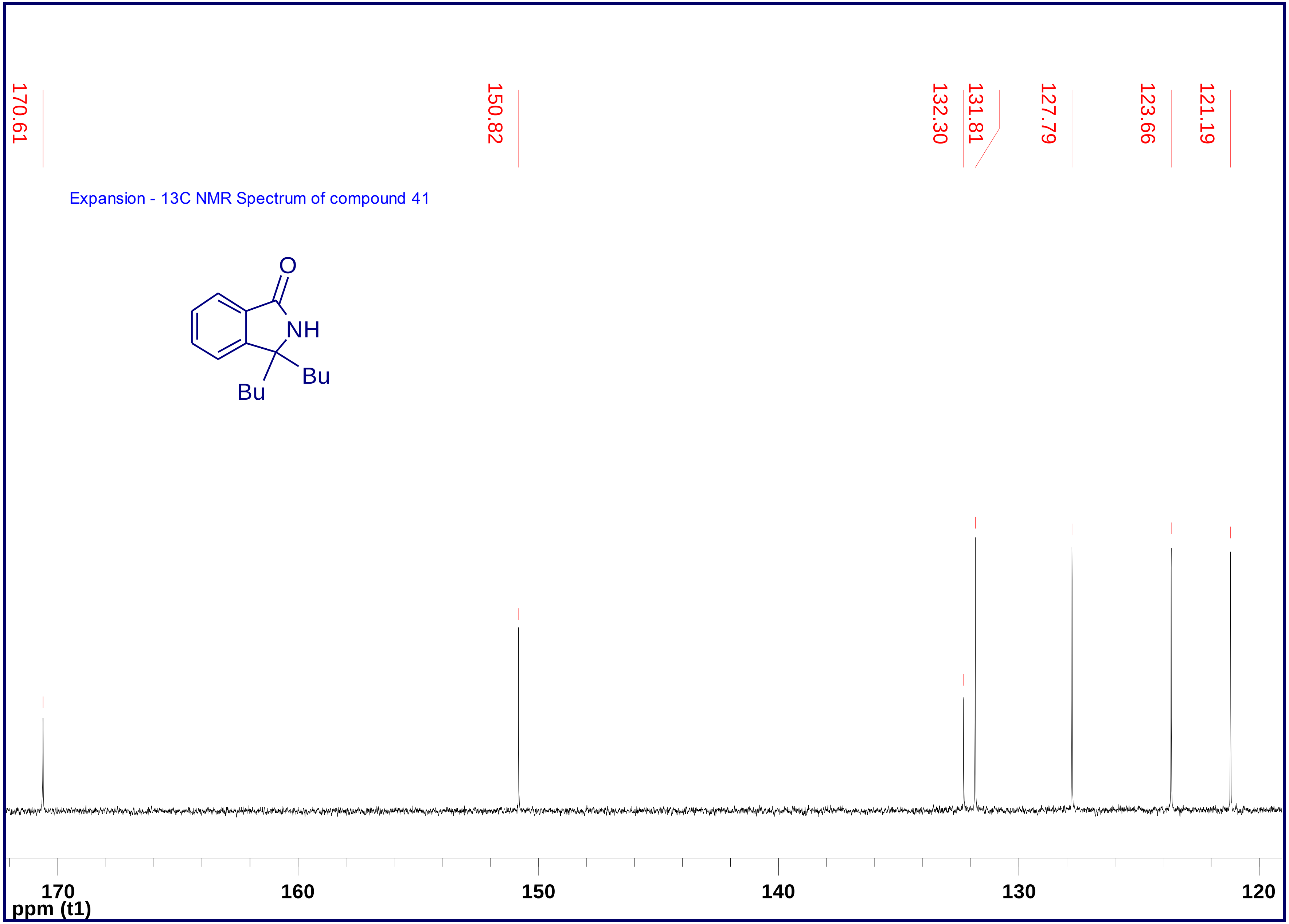
170.61
150.82
132.30
131.81
127.79
123.66
121.19
Expansion - 13C NMR Spectrum of compound 41
O
NH
Bu
Bu
170
160
150
140
130
120
ppm (t1)

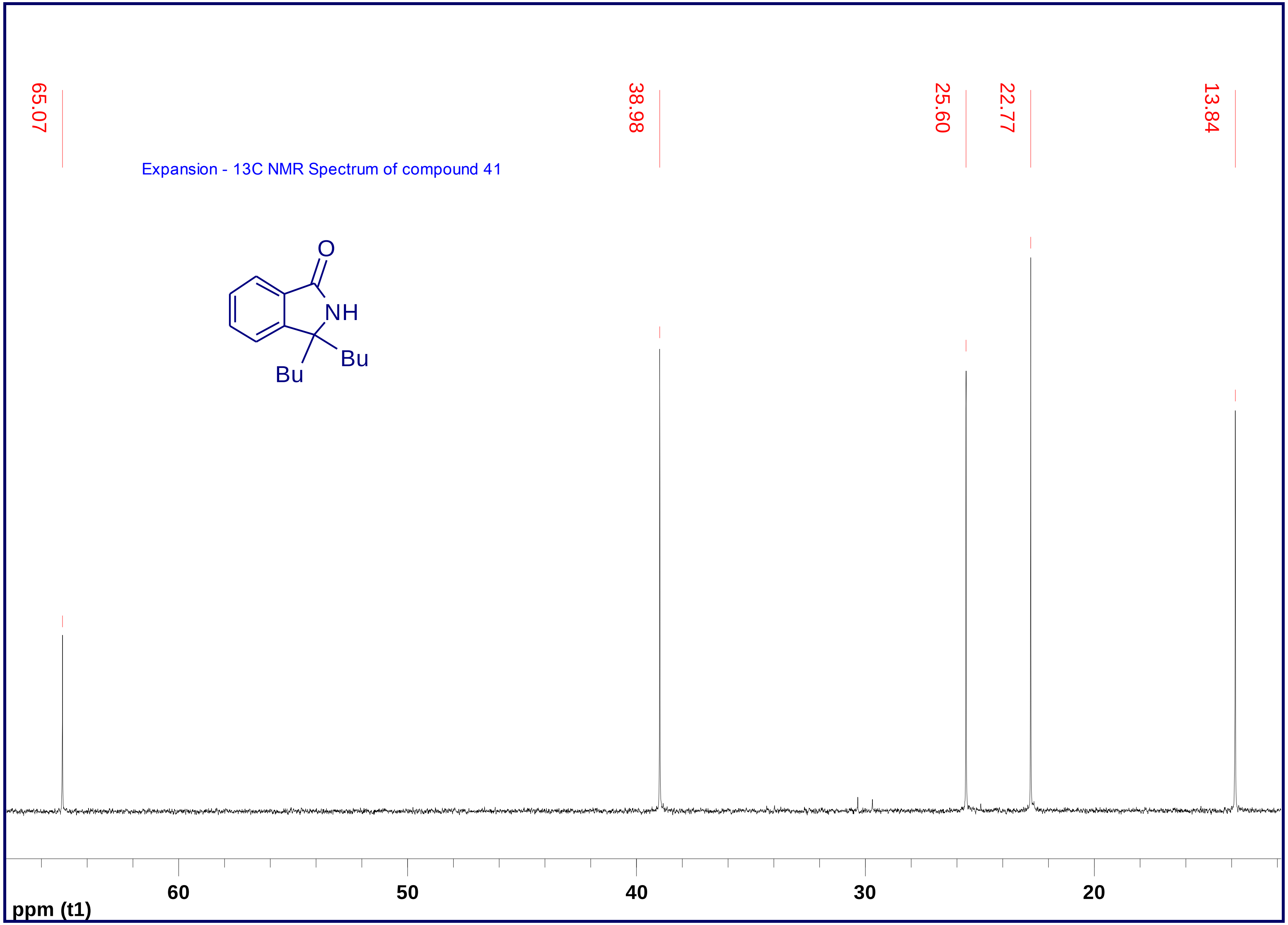

65.07
38.98
25.60
22.77
13.84
Expansion - 13C NMR Spectrum of compound 41
O
NH
Bu
Bu
60
50
40
30
20
ppm (t1)

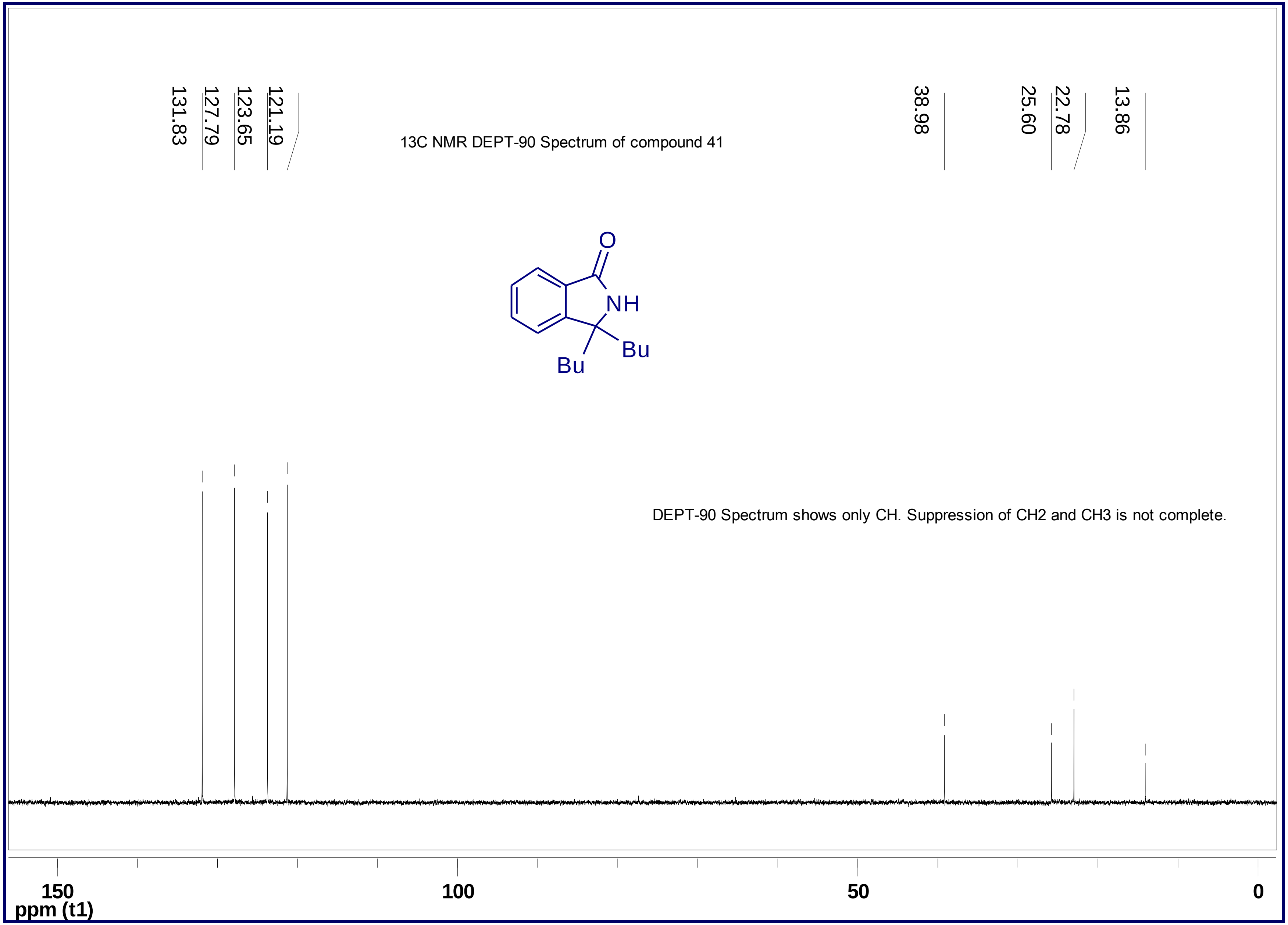
131.83
127.79
123.65
121.19
38.98
25.60
22.78
13.86
13C NMR DEPT-90 Spectrum of compound 41
O
NH
Bu
Bu
DEPT-90 Spectrum shows only CH. Suppression of CH2 and CH3 is not complete.
150
100
50
0
ppm (t1)

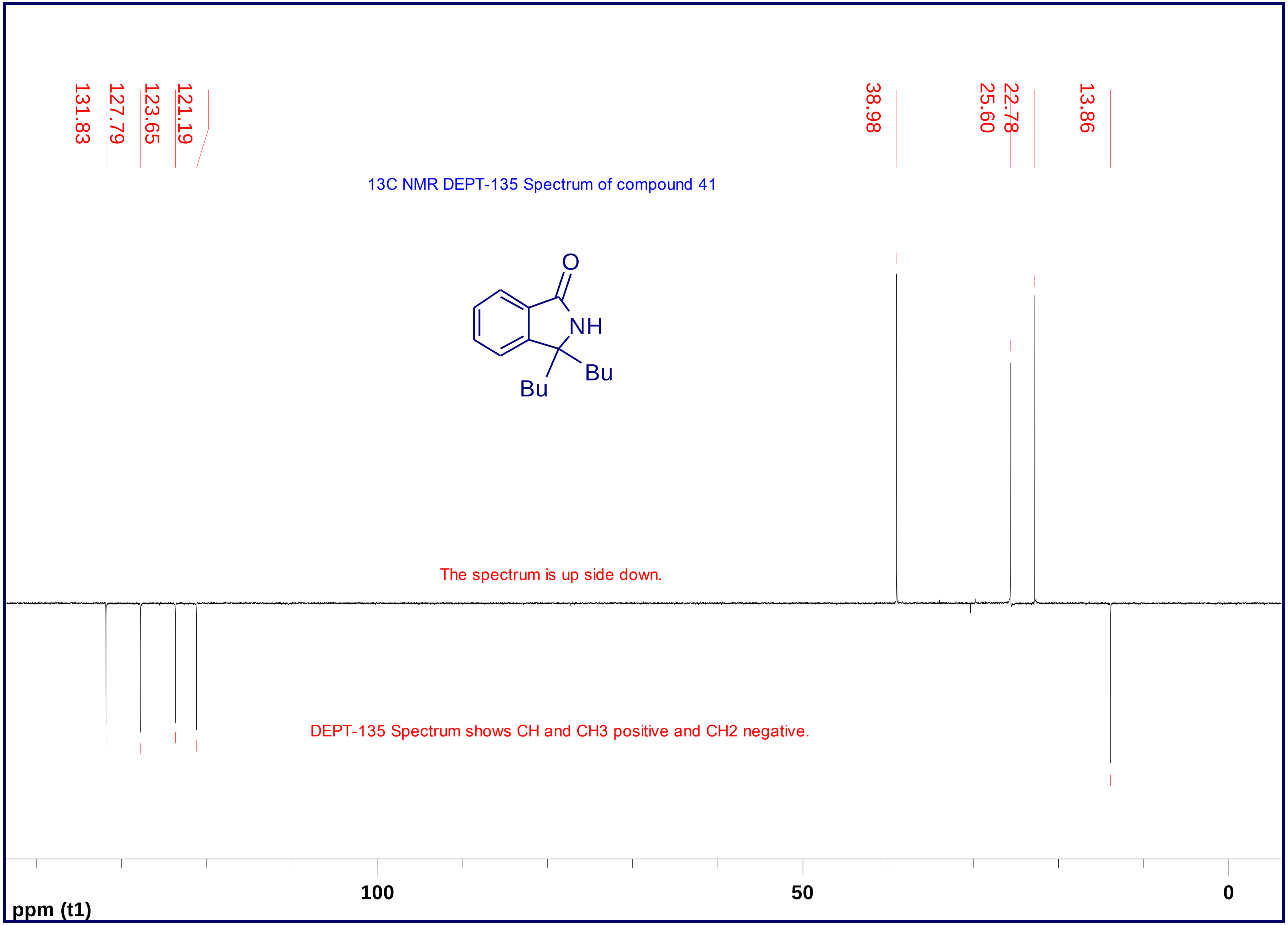

131.83
127.79
123.65
121.19
38.98
25.60
22.78
13.86
13C NMR DEPT-135 Spectrum of compound 41
O
NH
Bu
Bu
The spectrum is up side down.
DEPT-135 Spectrum shows CH and CH3 positive and CH2 negative.
100
50
0
ppm (t1)

Crystal and Structure Refinement Data of Compounds 12 and 15

**Structure**	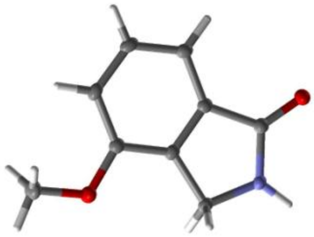	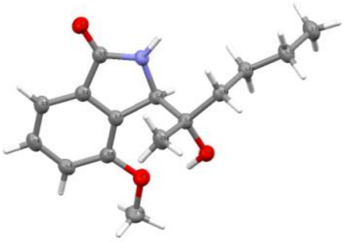
	**12**	**15**
CCDC reference	737411	762624
Empirical formula of crystal unit	$C_9H_9NO_2$	$C_{15}H_{21}NO_3$
Formula weight of crystal unit	163.17	263.33
Temperature/K	150(2)	150(2)
Crystal system	Triclinic	Monoclinic
Space group	P-1	P21/a
a/Å	7.2640(5)	9.7517(5)
b/Å	7.8940(6)	13.5852(7)
c/Å	8.3280(8)	11.0154(4)
α/°	104.845(3)	90
β/°	114.462(4)	95.266(3)
γ/°	103.622(5)	90
V/$Å^3$	387.21(5)	1453.15(12)
Z	2	4
$\rho_{cal/}$Mgm^{-3}	1.400	1.204
μ/mm^{-1}	0.100	0.083
Crystal size/mm^3	0.25 x 0.24 x 0.18	0.30 x 0.06 x 0.06
Reflections collected	2379	5380
Independent	1714	3240
R(int)	0.0301	0.0661
Parameters	110	176
R1	0.0512	0.0725
wR2	0.1220	0.1445

Crystal and Structure Refinement Data of Compounds 16-18

**Structure**	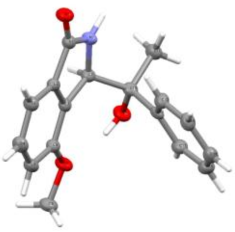 **16**	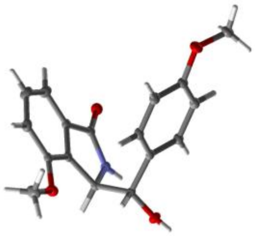 **17**	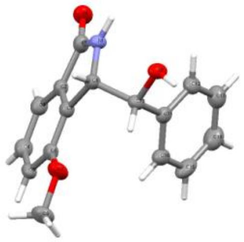 **18**
CCDC reference	766180	737415	762623
Empirical formula of crystal unit	$C_{17}H_{17}NO_3$	$C_{17}H_{17}NO_4$	$C_{18}H_{19}NO_4$
Formula weight of crystal unit	283.32	299.32	313.34
Temperature/K	150(2)	293(2)	150(2)
Crystal system	Triclinic	Centrosymmetric	Triclinic
Space group	P-1	P-1	P-1
a/Å	8.9567(5)	7.5240(3)	7.5698(6)
b/Å	9.0050(3)	9.4100(3)	9.2785(8)
c/Å	9.3788(4)	11.9960(5)	12.2934(10)
α/°	67.768(3)	83.877(2)	78.473(4)
β/°	89.017(3)	77.311(2)	82.365(4)
γ/°	81.593(3)	68.559(2)	70.596(3)
$V/Å^3$	692.09(5)	770.92(5)	795.91(11)
Z	2	2	2
$\rho_{cal/}Mgm^{-3}$	1.360	1.289	1.307
μ/mm^{-1}	0.093	0.092	0.093
Crystal size/mm^3	0.20 x 0.10 x 0.10	0.20 x 0.15 x 0.15	0.30 x 0.30 x 0.10
Reflections collected	4270	5071	4991
Independent	3038	3513	3467
R(int)	0.0345	0.0334	0.0536
Parameters	194	202	238
R1	0.0511	0.0570	0.0774
wR2	0.1271	0.1277	0.1657

Crystal and Structure Refinement Data of Compounds 38 and 39

Structure	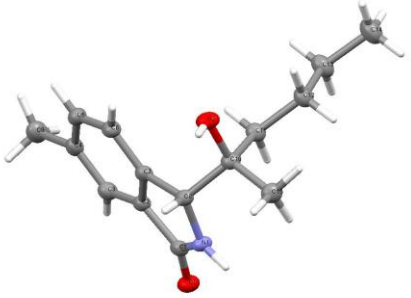 38	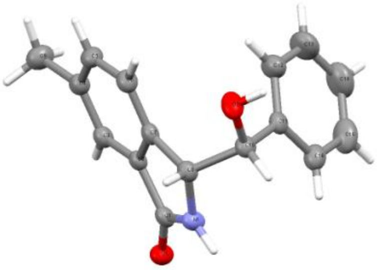 39
CCDC reference	766182	766181
Empirical formula of crystal unit	$C_{36}H_{56}N_2O_4$	$C_{16}H_{15}NO_2$
Formula weight of crystal unit	580.83	253.29
Temperature/K	150(2)	150(2)
Crystal system	Triclinic	Monoclinic
Space group	P-1	C2/c
a/Å	7.1881(4)	23.4249(10)
b/Å	10.5950(4)	7.8424(3)
c/Å	12.5474(5)	15.7782(4)
α/°	112.175(2)	90
β/°	96.371(2)	113.612(2)
γ/°	101.774(2)	90
V/Å^3	847.46(7)	2655.90(17)
Z	1	8
$\rho_{cal/}$Mgm^{-3}	1.138	1.267
μ/mm^{-1}	0.073	0.084
Crystal size/mm^3	0.40 x 0.30 x 0.02	0.15 x 0.15 x 0.10
Reflections collected	5995	8198
Independent	3840	3039
R(int)	0.0434	0.0544
Parameters	195	174
R1	0.0656	0.0504
wR2	0.1716	0.1337
